# RNAi screening identifies a new Toll from shrimp *Litopenaeus vannamei* that restricts WSSV infection through activating Dorsal to induce antimicrobial peptides

**DOI:** 10.1371/journal.ppat.1007109

**Published:** 2018-09-26

**Authors:** Haoyang Li, Bin Yin, Sheng Wang, Qihui Fu, Bang Xiao, Kai Lǚ, Jianguo He, Chaozheng Li

**Affiliations:** 1 School of Marine Sciences, Sun Yat-sen University, Guangzhou, P. R. China; 2 State Key Laboratory for Biocontrol, School of Life Sciences, Sun Yat-sen University, Guangzhou, P. R. China; 3 Institute of Aquatic Economic Animals and Guangdong Province Key Laboratory for Aquatic Economic Animals, Sun Yat-sen University, Guangzhou, P. R. China; 4 Guangdong Provincial Key Laboratory of Marine Resources and Coastal Engineering, Sun Yat-sen University, Guangzhou, P. R. China; 5 South China Sea Resource Exploitation and Protection Collaborative Innovation Center (SCS-REPIC), Sun Yat-sen University, Guangzhou, P. R. China; University of Tennessee Health Science Center, UNITED STATES

## Abstract

The function of Toll pathway defense against bacterial infection has been well established in shrimp, however how this pathway responds to viral infection is still largely unknown. In this study, we report the Toll4-Dorsal-AMPs cascade restricts the white spot syndrome virus (WSSV) infection of shrimp. A total of nine Tolls from *Litopenaeus vannamei* namely Toll1-9 are identified, and RNAi screening *in vivo* reveals the Toll4 is important for shrimp to oppose WSSV infection. Knockdown of Toll4 results in elevated viral loads and renders shrimp more susceptible to WSSV. Furthermore, Toll4 could be a one of upstream pattern recognition receptor (PRR) to detect WSSV, and thereby leading to nuclear translocation and phosphorylation of Dorsal, the known NF-κB transcription factor of the canonical Toll pathway. More importantly, silencing of Toll4 and Dorsal contributes to impaired expression of a specific set of antimicrobial peptides (AMPs) such as anti-LPS-factor (ALF) and lysozyme (LYZ) family, which exert potent anti-WSSV activity. Two AMPs of ALF1 and LYZ1 as representatives are demonstrated to have the ability to interact with several WSSV structural proteins to inhibit viral infection. Taken together, we therefore identify that the Toll4-Dorsal pathway mediates strong resistance to WSSV infection by inducing some specific AMPs.

## Author summary

The TLR pathway mediated antiviral immune response is well identified in mammals, yet, Toll pathway governing this protection in invertebrates remains unknown. In the present study, we uncover that a shrimp Toll4 from a total of nine Tolls in *L*. *vannamei* confers resistance to WSSV thought inducing the NF-κB transcription factor Dorsal to inspire the production of some antimicrobial peptides (AMPs) with antiviral activity. The anti-LPS-factor (ALF) and lysozyme (LYZ) family are identified as the Toll4-Dorsal pathway targeted genes with the ability to interact with viral structural proteins in response to WSSV infection. These results suggest that the Toll receptor induces the expression of AMPs with antiviral activity could be a general antiviral mechanism in invertebrates and Toll pathway established antiviral defense could be conserved during evolution.

## Introduction

Multicellular organisms have evolved for the ability to protect themselves from a wide variety of pathogens such as viruses. In invertebrates including shrimps that lacking immunoglobulin-based adaptive immune system, this protection is thus provided through the action of an innate immune system. The innate immune response is generally initiated via the detection of pathogen-associated molecular patterns (PAMPs), some evolutionarily conserved structures or motifs shared by broad classes of invading organisms, by a wide diversity of host pattern recognition receptors (PRRs) [1]. One important class of PRRs is the Toll receptor superfamily, comprising invertebrate Tolls and vertebrate Toll-like receptors (TLRs), and is now considered to be the primary sensor of pathogens in all metazoans [2].

Mammalian TLRs play universal and pivotal roles in host defenses mainly via the innate immune system, but also the immunoglobulin-based adaptive immune system that are devoid in invertebrate [3]. In human, the function of TLR signaling pathway is clearly clarified, and the ten TLRs can directly recognize and bind to a number of diverse molecular structures, including lipids (e.g., TLR4: LPS via MD2; TLR1/2/6: lipoproteins), proteins (e.g., TLR5: flagellin; TLR2 and TLR4: HMGB1), and nucleic acids (e.g., TLR3: dsRNA; TLR7/8: ssRNA; and TLR9: unmethylated CpG motifs in bacterial, viral, and fungal DNA) [4]. It is generally accepted that TLRs require ligand‐induced dimerization or crosslinking to initiate intracellular signaling event [4]. Ligand binding is likely to lead to a conformational rearrangement of the cytoplasmic TIR domains, thereby creating a docking site to which TIR domain containing adaptors MyD88/TIRAP/TRIF/TRAM can be recruited [2, 4]. The stimulation of TLRs then results in activation of the NF-κB (nuclear factor κB) transcription factors that drive the transcription of proinflammatory cytokines, or/ and trigger IRF (interferon regulatory factor) transcription factors that induce transcription of type I interferon cytokines, both of which ultimately confer immune response against infection [2, 4].

*Drosophila* genome encodes nine Toll receptor genes (Toll1 to Toll9), but these Tolls are different and elusive in the context of function, ligand sensing and intracellular signaling compared to those of mammals. *Drosophila* Toll1 (or simply, Toll) generally binds an endogenous cytokine Spätzle (Spz) rather than microbe motif [5]. In contrast to that mammalian TLRs function mainly in immunity, *Drosophila* Toll1 functions not only in developmental processes [6, 7], but also in innate immunity response to bacterial, fungal, and viral infections [8–10]. *Drosophila* Toll7 can directly interact with vesicular stomatitis virus (VSV) virion at the plasma membrane perhaps in a manner similar to mammalian TLRs, and induces antiviral autophagy independent of the canonical Toll signaling pathway (MyD88/Tube/Pelle/Dorsal or Dif cascade) [11]. Besides, *Drosophila* Toll2 (18-wheeler) may play a minor role in the antibacterial response [12], and Toll5 and Toll9 can trigger the production of the antifungal gene Drosomycin [13]. By now, only the *Drosophila* Toll1 is definitely identified as upstream receptor of Dorsal or Dif, two transcription factors homologous to *Human* NF-κB [14]. However, the function and intracellular signaling routes of the remaining Tolls in response to infection are not well characterized.

Cultured shrimps frequently suffer from many DNA and RNA viruses, among which white spot syndrome virus (WSSV) has been considered as the most serious threat to shrimp aquaculture industries and caused serious economic losses every year [15, 16]. WSSV is a large and enveloped dsDNA virus, which is highly pathogenic and especially virulent on penaeid shrimp [15, 17]. There is growing interest in research of the interplay between WSSV and many aspects of shrimp host [18, 19], but the precise function of Toll receptors and Toll pathway related genes participating in viral infection remains to be determined. Until now, a large number of Tolls have been identified in multiple shrimps including *Litopenaeus vannamei* [20, 21], *Fenneropenaeus chinensis* [22], *Penaeus monodon* [23–25], *Marsupenaeus japonicas* [26], *Macrobrachium rosenbergi* [27], *Cherax quadricarinatus* [28] and *Procambarus clarkii* [29, 30]. In *L*. *vannamei*, three Tolls of the Toll1, Toll2 and Toll3 are up-regulated after WSSV challenge, whereas their functions during WSSV infection are not well characterized [21]. Some shrimp Tolls from *C*. *quadricarinatus*, *P*. *clarkii* and *M*. *rosenbergii* have been shown to induce some AMPs expression in response to WSSV infection, which indicate these AMPs could play an antiviral role [28, 29, 31]. Besides, WSSV infection can contribute to activation of many *P*. *monodon* Toll pathway genes, which suggest that the whole pathway play a crucial role in the immune response during WSSV infection [32]. In a recent study, the Toll3 from *L*. *vannamei* is demonstrated to have the ability to activate the expression of interferon regulatory factor (IRF) and its downstream Vago4/5, suggesting it could play a critical role in host antiviral immunity independent of the canonical Toll signaling pathway [33]. Overall, some shrimp Tolls have been shown to participate in innate immune responses against viral infection, but their antiviral functions remain largely elusive and their underlying antiviral mechanism needs further investigation clearly.

Herein, we clone and identify a total of nine Tolls (Toll1-9) from *L*. *vannamei*, and RNAi screening demonstrates Toll4 as a critical antiviral factor against WSSV *in vivo*. Mechanismly, Toll4 senses WSSV infection and leads to activation of Dorsal to converge on the production of some specific AMPs such as ALFs and LYZs, which confer host defense against WSSV by targeting its structural proteins. These data provide evidence that a new identified Toll4 senses WSSV and initiates an antiviral response in shrimp.

## Results

### Cloning, sequence analysis and phylogenetic tree of shrimp nine Tolls

**Fig 1:**
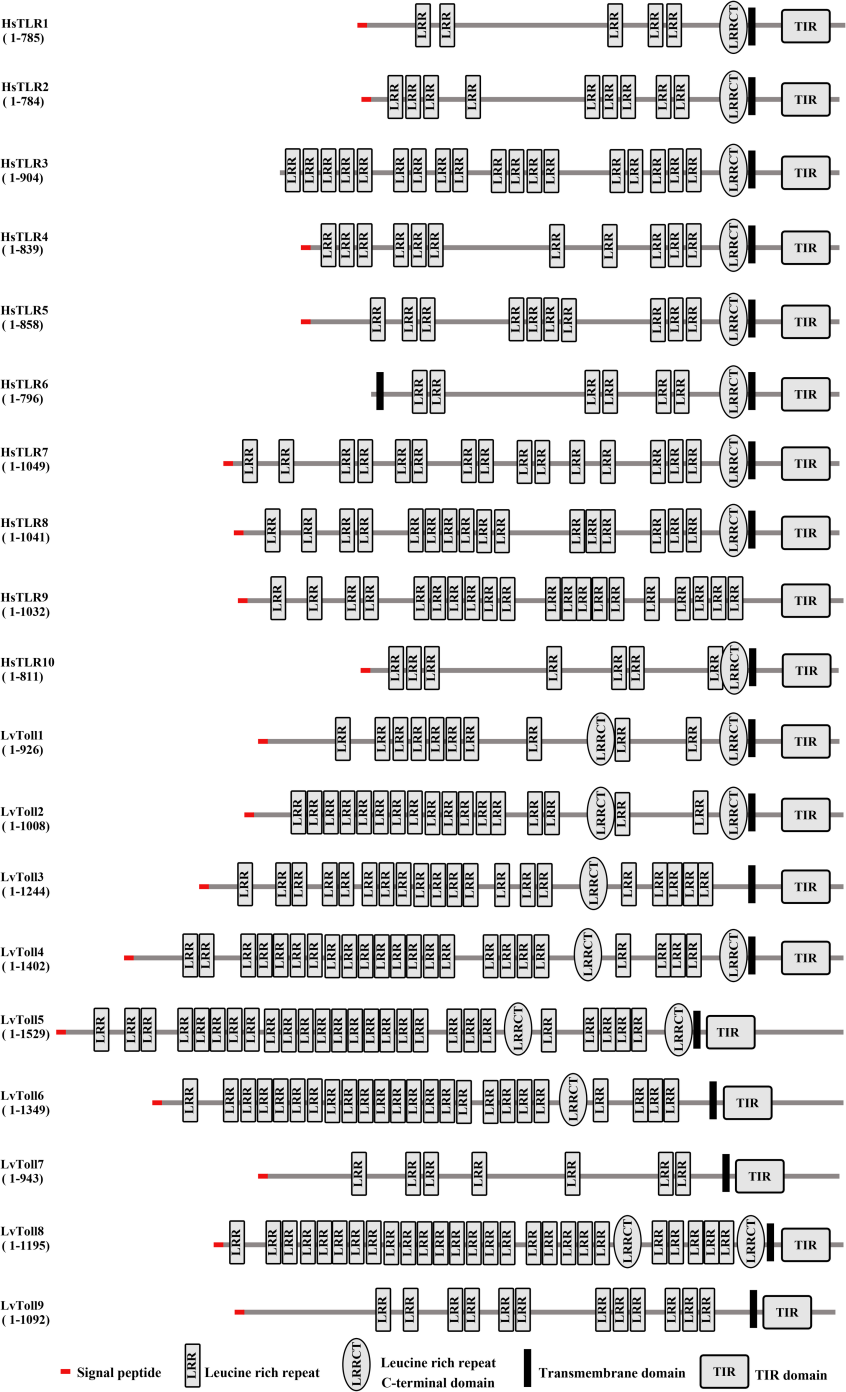
Schematic representations of the domain topology of *L*. *vannamei* Tolls (LvToll1–9) and human TLRs (HsTLR1–10) according to SMART analysis.

In order to identify all candidate Toll genes from shrimp (*L*. *vannamei*), we carried out protein homology search by local TBLASTN program against our transcriptome data from whole bodies of *L*. *vannamei* (all tissues pooled) [34] and other *L*. *vannamei* transcriptome data from NCBI [35–38]. A total of nine individual and putative Toll homologs from *L*. *vannamei* were obtained, of which the Toll1, Toll2 and Toll3 were perfectly identical to previous reported LvToll1, LvToll2 and LvToll3, respectively [21]. We next cloned the full-length cDNA sequences of other Tolls by using rapid amplification cDNA ends (RACE)-PCR method, and we subsequently designated these Tolls from Toll1 to Toll9 according to the time order of their being cloned. Their sequences of Toll1-9 in FASTA format were available in Supplement Data S1. Functional domain analysis indicated that each of the nine Tolls adopted a typical domain organization characteristic of Toll family gene including an N-terminal signal peptide, an extracellular domain, a single transmembrane region and an intracellular TIR domain in the C-terminal (Fig 1). These Tolls varied considerably in the number of extracellular LRRs from 7 (Toll7) to 28 (Toll5) (Fig 1), and the intracellular TIR domains were not conserved among each other except for the two pairs of Toll1/2 and Toll3/Toll8 bearing sequence identities of greater than or equal to 70% (S1 Fig). The significant differences both in number of extracellular LRRs and sequence identities of intracellular TIR domains may suggest that these Tolls are able to respond to various extracellular stresses (pathogens or ligands) and exploit distinct intracellular signaling routes. Phylogenetic tree analysis showed that most of Tolls from invertebrates including the Tolls from *L*. *vannamei* Toll1 to Toll6, and Toll8, as well as the Tolls from insects and other species were clustered together. However, *L*. *vannamei* Toll7 and Toll9 and *Drosophila* Toll9 were clustered with vertebrates TLRs, which were separated with several groups such as TLR1 to TLR10, TLR12 and TLR15 (S2 Fig). Taken together, we cloned and identified nine Tolls from *L*. *vannamei*, among which six Tolls namely the Toll4, Toll5, Toll6, Toll7, Toll8 and Toll9 are firstly cloned and identified in *L*. *vannamei*.

### Toll4 and the canonical Toll pathway components restrict WSSV infection in shrimp

**Fig 2:**
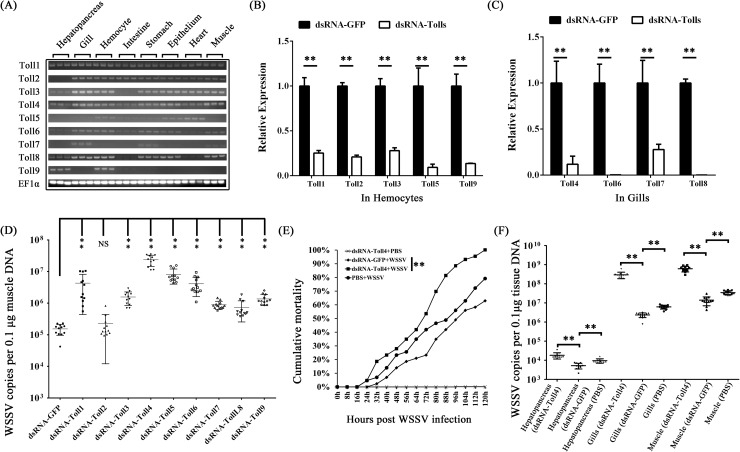
RNAi screening identifies Toll4 as an antiviral factor against WSSV. (A) Tissue distribution of *L*. *vannamei* Tolls (Toll1-9) was analyzed by Semi-RT-PCR, and EF1α was used as a control. (B-C) Knockdown efficiencies of Toll1, Toll2, Toll3, Toll5 and Toll9 in hemocytes (B), and silencing efficiencies of Toll4, Toll6, Toll7 and Toll8 in gills (B) were checked by qRT-PCR. The GFP dsRNA treated shrimp was set as a control. (D) RNAi screening identified shrimp Tolls (LvToll1-9) as potential antiviral (anti-WSSV) factors. WSSV was inoculated at 48 h post each Toll silencing. The viral load was assessed at 48 h post-infection through absolute qPCR. The experiment was repeated three times with similar results. One dot represented 1 shrimp and the horizontal line represented the median of the results. (E) Silencing of Toll4 enhanced shrimps susceptibility to WSSV infection. WSSV was inoculated at 48 h post Toll4 silencing, and the death of shrimp was recorded at every 8 h for cumulative mortality rates analysis. The experiment was repeated three times, and similar results were obtained. The data were analyzed statistically by the Kaplan–Meier plot (log-rank χ^2^ test) (** *p* < 0.01). (F) Silencing of Toll4 enhanced WSSV replication in multiply shrimp tissues. WSSV was inoculated at 48 h post Toll4 silencing, and the viral load was assessed at 48 hpi through absolute qPCR. The experiment was performed three times with similar results. All the data from B, C, D and F were analyzed statistically by student’s T test (** *p* < 0.01; NS, not significant).

To determine whether any of the shrimp Tolls are involved in antiviral defense against WSSV, we generated double-stranded RNA (dsRNA) against each of the nine Tolls and silenced them *in vivo* via dsRNA mediated RNA interference (RNAi). We firstly addressed the tissue distribution of shrimp nine Tolls transcripts by Semi-quantitative reverse transcription PCR (Semi-qRT-PCR). The results showed that both Toll1 and Toll4 could be detected in all the examined tissues and were highly expressed in gill, hemocyte and intestine, whereas other Tolls were abundant in only a few specific tissues (Fig 2A). According to the tissue distribution of each Tolls, the gill tissue was chosen to check the knockdown efficiencies for Toll4, Toll6, Toll7 and Toll8 (Fig 2C), while hemocyte was as the target tissue to evaluate silencing efficiencies for Toll1, Toll2, Toll3 Toll5 and Toll9 (Fig 2B). Efficient silencing for each Toll receptor was confirmed by quantitative reverse transcription PCR (qRT-PCR) (Fig 2B and 2C). Next, we challenged RNAi-treated shrimps with WSSV and subsequently analyzed viral genome copies (WSSV DNA) by absolute quantitative PCR (absolute q-PCR). We observed a greater number of each Toll except for Toll2 silenced shrimps exhibited higher quantities of viral titers in muscle when compared to control shrimps at 48 hours post infection (hpi) (Fig 2D). Of note, shrimps with silencing of Toll4 had the highest WSSV titers, and the average of viral DNA burden was approximately 150 times higher than that of the control shrimps (GFP dsRNA injected shrimps following WSSV infection) (Fig 2D). Therefore, we focused our special attention on the Toll4 mediated mechanism underlying resistance to WSSV in this study. To further confirm the above screening result, with *in vivo* RNAi again, a higher lethality was observed in the silencing of Toll4 shrimps followed by WSSV infection when compared to that of the dsRNA-GFP treated control group (Fig 2E). To investigate whether the increased lethality rates of Toll4 silenced shrimps was due to decreased resistance or tolerance to WSSV, we analyzed the viral levels by absolute q-PCR in several tissues including hepatopancreas, gill and muscle at 48 hpi. We observed that shrimps with knockdown of Toll4 had elevated viral replication levels in all the three tissues than dsRNA-GFP treated control shrimps (Fig 2F). Of note, dsRNA-GFP group had higher cumulative mortality rate and viral loads than the PBS group (Fig 2E and 2F), which supported the conclusion of existence of nucleic acids induced antiviral immunity in shrimps [39–43].The obviously lower viral levels observed in hepatopancreas than those of gill and muscle could be explained by that only the connective tissue and myoepithelial cells of the hepatopancreas sheath are infected by WSSV [44, 45]. Collectively, these data strongly suggest Toll4 as a critical antiviral factor against WSSV *in vivo*.

**Fig 3:**
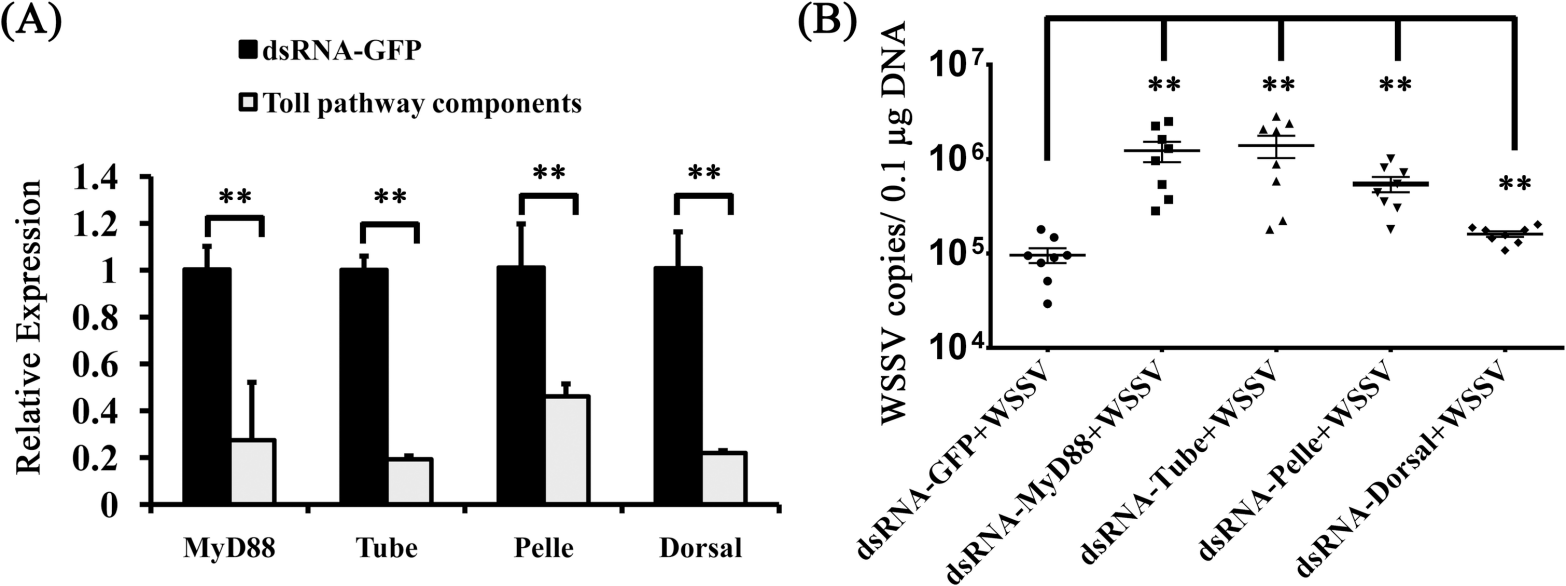
Increased viral replication levels in the canonical Toll pathway components silenced shrimps. (A) Knockdown efficiencies of the canonical Toll pathway components including MyD88, Tube, Pelle and Dorsal in gills were checked by qRT-PCR. The GFP dsRNA treated shrimp was set as a control. (B) Silencing of the canonical Toll pathway components resulted in enhanced WSSV replication levels in gill tissues. WSSV was inoculated at 48 h post each component silencing, and the viral load was assessed at 48 hpi through absolute qPCR. The experiment was performed three times with similar results. All the data were analyzed statistically by student’s T test (** *p* < 0.01).

Because the Toll4 can confer protection against WSSV, we then explored whether the canonical Toll pathway components including MyD88, Tube, Pelle and Dorsal are involved in antiviral responses. We injected shrimps with dsRNA of each component to depress their expression, which were confirmed by quantitative RT-PCR (Fig 3A). We observed that knockdown of these critical Toll pathway components had notable impact on WSSV replication *in vivo* (Fig 3B), suggesting that the canonical Toll pathway plays an important role against WSSV. In summary, these data show that Toll4 and the canonical Toll pathway components are crucial to oppose WSSV infection.

### Toll4 regulates antimicrobial peptides (AMPs) production upon WSSV infection in shrimp

**Fig 4:**
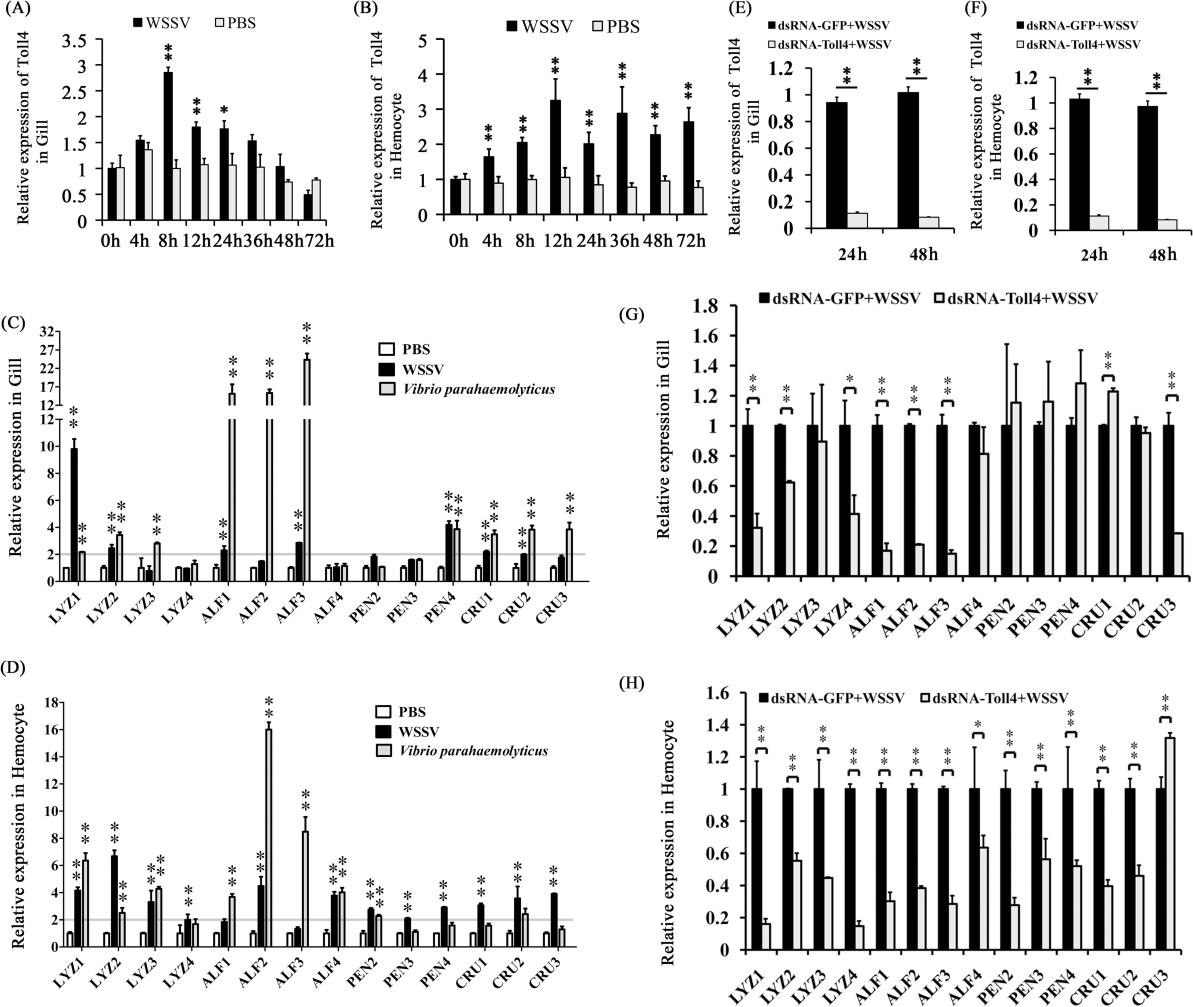
AMPs expression levels in Toll4 silenced shrimps upon WSSV infection. (A-B) Expression profiles of Toll4 after WSSV infection in gill (A) and hemocyte (B) were assessed by qRT-PCR. (C-D) AMPs expression patterns responding to the challenge of WSSV, *V*. *parahaemolyticus* (as an activator for canonical Toll pathway) and PBS (as a negative control) in gill (C) and hemocyte (D) was detected by qRT-PCR. The horizontal line indicated 2-fold induction threshold. (E-F) Knockdown efficiencies of Toll4 in gill (E) and hemocyte (F) was confirmed by qRT-PCR at 24 and 48 h post dsRNA injection. (G-H) Toll4-knockdown shrimps had impaired AMPs expression levels upon WSSV infection both in gill (G) and hemocyte (H). All experiments were performed three times, and similar results were observed. All the data were analyzed statistically by student’s T test (* *p* < 0.05; ** *p* < 0.01).

Mammalian TLRs opposed viral infection mainly through inducing the type I interferon (IFN) expression [46], whereas *Drosophila* Toll restricted viral infection via inspiring some specific AMPs production [10]. To address whether the involvement of Toll4 in regulating AMPs synthesis, we firstly studied the response of Toll4 to viral infection and measured the transcriptional changes of Toll4 after WSSV challenge in the two immune related tissues gill and hemocyte by quantitative RT-PCR. The results showed that Toll4 in gill was remarkably up-regulated from 8 hpi to 24 hpi compared to the control shrimps injected only PBS (Fig 4A), and Toll4 in hemocytes maintained an elevated expression level in the whole period of infection (Fig 4B). To test whether some known shrimp AMPs can respond to WSSV infection, we detected the transcriptional levels of fourteen shrimp AMPs consisting of four different AMP families of anti-lipopolysaccharide (LPS) factor (ALF), lysozyme (LYZ), penaeidin (PEN) and crustin (CRU), by quantitative RT-PCR at 6 hpi in gill and hemocyte tissues (Fig 4C and 4D). These sequences of fourteen shrimp AMPs in FASTA format were available in Supplement Data S2. *Vibrio parahaemolyticus* is a pathogen of shrimp has been verified as an activator of the shrimp Dorsal-AMPs pathway [47]. Thus it was used here as a control to reveal the levels of AMPs expression in bacterial infection and in WSSV infection. PBS controls were used for baseline levels of AMPs expression. We found that, except for ALF4, PEN2 and PEN3 with slight a up-regulation less than 2-fold in gills, both WSSV and *V*. *parahaemolyticus* infection induced considerably increased expression levels of the most of AMPs over 2-fold than those of control shrimps in the both tissues (Fig 4C and 4D). Moreover, we used the RNAi to knockdown the expression of Toll4 *in vivo* once again, and the silencing efficiencies of Toll4 in gill and hemocyte at 24 h and 48 h post dsRNA injection were further confirmed by quantitative RT-PCR (Fig 4E and 4F). After 48 h post dsRNA injection, we infected the RNAi treated shrimps by injection of WSSV, and probed the expression levels of the fourteen AMPs at 6 hpi. The results showed that all of these AMPs, except for CRU3 with a slight up-regulation, were dramatically down-regulated compared to the control in hemocytes (Fig 4H). In the gills of WSSV infected shrimps, RNAi of Toll4 led to the down-regulated secretion of LYZ1, LYZ2, LYZ4, ALF1, ALF2, ALF3 and CRU3, almost of which belonged to LYZ and ALF families (Fig 4G). These results suggest that Toll4 induced AMPs expression may vary in gill and hemocyte tissues under WSSV infection. Even so, most of the tested AMPs from LYZ and ALF families in both hemocytes and gills showed an identical down-regulation pattern in Toll4 silenced shrimps under WSSV infection. In summary, we conclude that WSSV infection in shrimp could activate Toll4 expression and perhaps through Toll4 to induce the production of a specific set of AMP genes including LYZ1, LYZ2, LYZ4, ALF1, ALF2 and ALF3 both in gill and hemocyte tissues.

### Dorsal is activated following exposure to WSSV challenge

**Fig 5:**
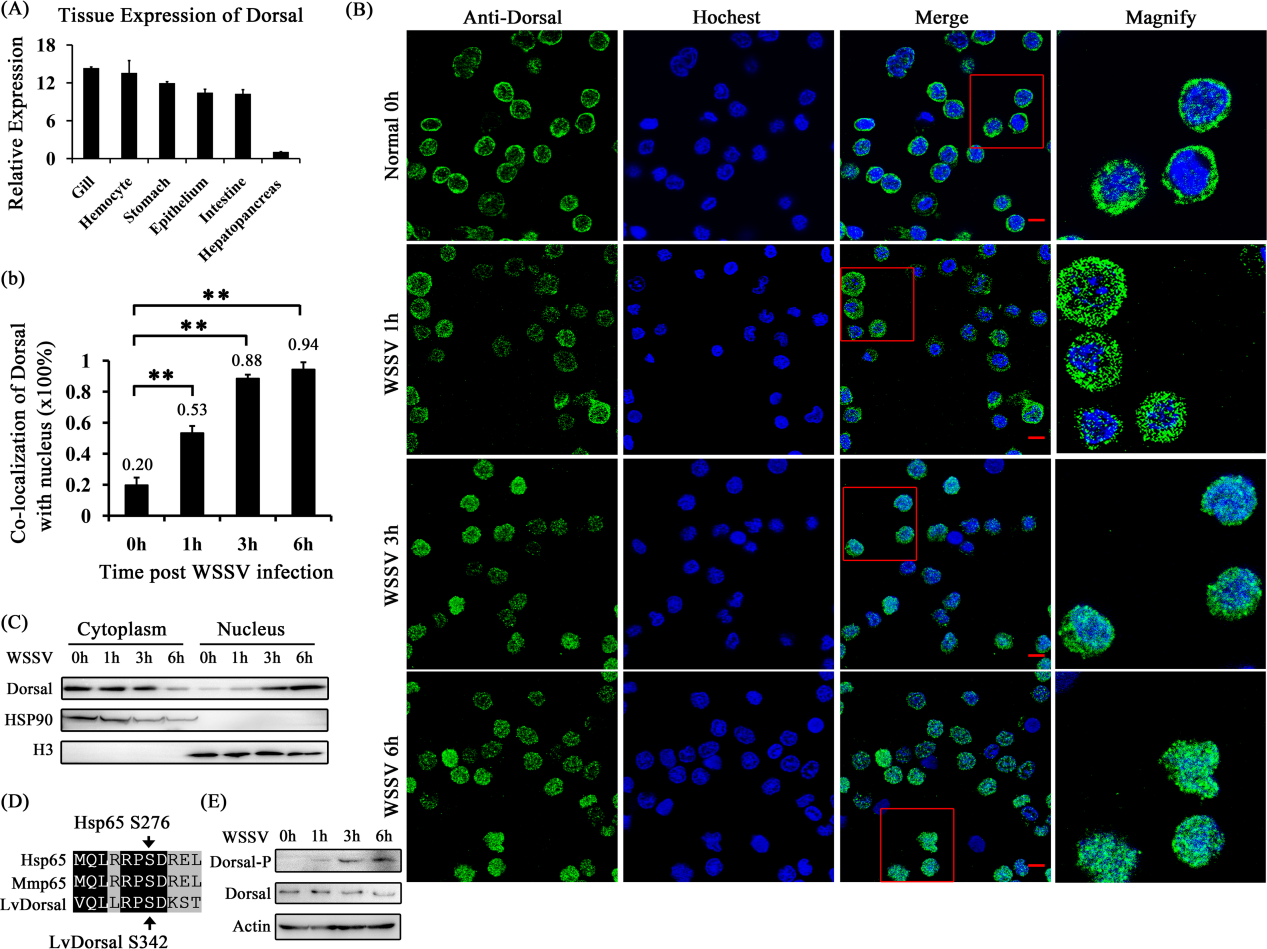
Dorsal nuclear translocation and phosphorylation are induced by WSSV infection. (A) Tissue distribution of Dorsal was analyzed by qRT-PCR. (B) Dorsal nuclear translocation in hemocytes was detected at 1 h, 3 h and 6 h post WSSV infection, and the WSSV untreated hemocytes (0 h) as a control. The hemocytes were collected at 0 h, 1 h, 3 h and 6 h post WSSV infection, deposited onto a glass slide and subjected to immunocytochemical staning by a prepared anti-Dorsal specific antibody, and finally visualized on a confocal laser scanning microscope. (b) Co-localization of Dorsal and Hochest-stained nucleus in hemocytes was calculated by WCIF ImageJ software and analyzed statistically by student’s T test (** *p* < 0.01). (C) The subcellular distribution of Dorsal in hemocytes was detected by western blotting. (D) Dorsal contained a putative phosphorylation site (Ser342) in a conserved region across species. (E) Dorsal was phosphorylated at Ser342 after WSSV infection and analyzed by western blotting with human anti-NF-κB p65 (phospho S276) antibody. All experiments were performed three times, and similar results were obtained.

Since we observed that Toll4 responded to WSSV infection and ultimately led to the induction of a specific set of AMP genes, we explored whether Dorsal, the NF-κB transcription factor known to be downstream of the canonical Toll pathway [48], is activated during viral infection. We firstly detected the tissue distribution of shrimp Dorsal, and found that Dorsal showed high expression levels in gill, hemocyte, stomach, intestine and epithelium, but low in hepatopancreas (Fig 5A). We then investigated the effect of WSSV infection on Dorsal nuclear translocation by immunofluorescence staining in shrimp hemocytes. To gain more information on nuclear translocation of Dorsal upon WSSV infection, we firstly detected the dynamics of Dorsal translocation at several times, and we observed that shrimp hemocytes exhibited gradually and significantly increased nuclear translocation levels of Dorsal (53%, 88% and 94%) compared to the control hemocytes treated with PBS (Normal 0 h, 20%) at 1 h, 3 h and 6 h after WSSV challenge, respectively (Fig 5B and b). To further confirm the above results, we probed Dorsal translocation from the cytoplasm to the nucleus upon WSSV infection by using an *L*. *vannamei* Dorsal specific antibody prepared in our previous study [49]. In good agreement with the results of immunofluorescence staining, we were able to detect more nuclear import of Dorsal in shrimp hemocytes along with the times of WSSV infection from 0 h to 6 h, while at 6 hpi only a slight signal of Dorsal can be detected in the cytoplasm (Fig 5C). In addition to nuclear translocation, the activation of Dorsal could be manifested by phosphorylation on some specific amino acids. We found that shrimp Dorsal contains a considerable conserved region, which displays a significant degree of sequence similarity to a comparison region of its mammalian counterparts. In this region, human p65 NF-κB factor contains a Ser276, corresponding to Ser342 of shrimp Dorsal, which can be phosphorylated upon many stresses including viral infection [50] (Fig 5D). We therefore hypothesize that the Ser342 of Dorsal could also be phosphorylated after WSSV infection, and we detected Dorsal phosphorylation by using the human p65 Ser276 phosphorylation antibody. The results showed that a strong phosphorylation signal of Dorsal was observed at 3 hpi and 6 hpi (Fig 5E), correlating well with Dorsal enrichment in the nucleus at 3 hpi and 6 hpi, respectively. Taken together, WSSV infection did induce shrimp Dorsal translocated from the cytoplasm to the nucleus, and phosphorylation on Ser342.

### Dorsal regulates Toll4 dependent AMP genes expression after WSSV infection

**Fig 6:**
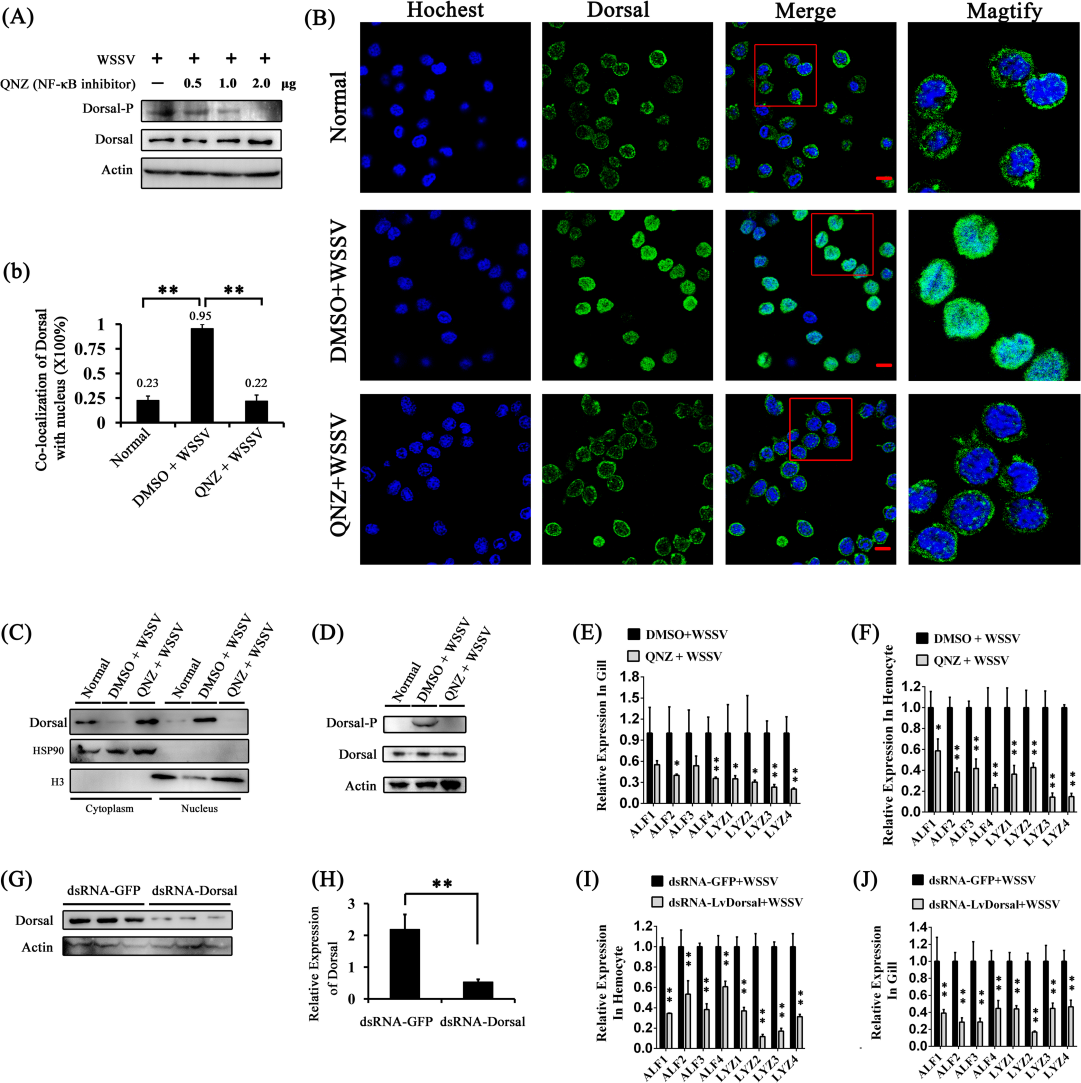
Dorsal regulates the same AMPs modulated by Toll4 upon WSSV infection. (A) Expression of Dorsal-P and Dorsal in QNZ-treated hemocytes at 6 h post WSSV infection were detected by western blotting. (B) Dorsal nuclear translocation in response to WSSV infection was inhibited by QNZ. Each shrimp was injected with 2 μg QNZ (EVP4593), followed by shrimp was intraperitoneal injected with WSSV. The hemocytes were collected at 6 h post WSSV infection, and then subjected to immunofluorescence staining. (b) Co-localization of Dorsal and nucleus in hemocytes corresponding to Fig 6B was calculated by WCIF ImageJ software and analyzed statistically by student’s T test (** *p* < 0.01). (C-D) The subcellular distribution (C) and phosphorylation level (D) of Dorsal in QNZ-treated hemocytes was detected at 6 h post WSSV infection by western blotting. The DMSO and WSSV untreated hemocytes were used as controls, respectively. (E-F) AMPs expression levels in hemocyte (E) and gill (F) of QNZ-treated shrimp at 6 h post WSSV infection were detected by qRT-PCR. (G-H) Silencing efficiencies for Dorsal protein levels (G) and mRNA levels (H) were affirmed by western blotting and qRT-PCR. (I-J) AMPs expression levels in hemocyte (I) and gill (J) of Dorsal silenced shrimp at 6 h post WSSV infection were assessed by qRT-PCR. All experiments were performed three times, and similar results were obtained. All the data from b, E, F, H, I and J were analyzed statistically by student’s T test (* *p* < 0.05; ** *p* < 0.01).

Because Dorsal can be activated upon WSSV infection, we reason that this activation of Dorsal could lead to trigger the expression of some AMPs. To address this, we detected the expression levels of Toll4 dependent AMP genes of ALF1-4 and LYZ1-4 *in vivo* either when Dorsal activity was suppressed by NF-кB inhibitor or when Dorsal expression was silenced by RNAi, respectively. Firstly, we measured whether a NF-κB inhibitor QNZ (EVP4593) can work for Dorsal activity suppression. We observed that the 2 ug/per shrimp was able to suppress Dorsal Ser342 phosphorylation efficiently *in vivo* and this quality was used in the following analysis (Fig 6A). In order to confirm whether Dorsal regulated AMPs expression, we injected the shrimp with the NF-κB inhibitor QNZ prior to the WSSV challenge, and investigated Dorsal translocation, phosphorylation and AMPs expression. We analyzed the effect of QNZ on Dorsal nuclear translocation in hemocytes under WSSV infection by immunofluorescence staining and western blotting (WB) analysis. The results showed that QNZ significantly suppressed Dorsal translocated from the cytoplasm to the nucleus (Fig 6B and b), which was consistent with the WB analysis of Dorsal cytoplasmic and nuclear localization (Fig 6C). Expectedly, Dorsal Ser342 phosphorylation was efficiently suppressed by QNZ, although shrimps hemocytes were under WSSV challenge (Fig 6D). Further, in the NF-κB inhibitor-injected shrimp the WSSV challenge failed to up-regulate Toll4 dependent AMPs expression both in hemocytes and gills, respectively (Fig 6E and 6F). On the other hand, we carried out RNAi to knockdown Dorsal *in vivo*, WB and qRT-PCR analysis confirmed efficient silencing of its protein levels (Fig 6G) and mRNA levels (Fig 6H), respectively. We observed that in the RNAi treated shrimps Toll4 dependent AMPs of ALF and LYZ families were marginally down-regulated after WSSV infection compared to GFP dsRNA treated control shrimps both in hemocytes and gills (Fig 6I and 6J). These results persuasively confirm that Dorsal nuclear translocation and phosphorylation are functionally related to the increased expression of Toll4 dependent AMPs under WSSV infection *in vivo*.

To further explore the potential mechanism by Dorsal to regulate expression of AMPs, ALF1 and LYZ1 as a representative was chosen and their 5' flanking regulatory regions (Supplement Data S4) were obtained by Genome Walking method. By JASPER tool prediction, we found the presence of a putative NF-κB binding site in ALF1 and LYZ1 promoter, respectively (S3A Fig). Luciferase reporter assays demonstrated that over-expression of *L*. *vannamei* Dorsal was able to activate the promoter activities of ALF1 and LYZ1 in a Dorsal-concentration dependent manner in *Drosophila* S2 cells (S3B Fig). In addition, EMSA experiments indicated that Dorsal can interact with the putative NF-κB binding sites in ALF1 and LYZ1 promoters (S3C Fig). Taken together, these evidences strongly suggest *L*. *vannamei* Dorsal is able to regulate the transcription of AMPs perhaps via interacting with the conserved NF-κB binding sites in their promoters such as ALF1 and LYZ1.

### WSSV but not other pathogens-induced Dorsal activation is partially dependent on Toll4 in shrimp

**Fig 7:**
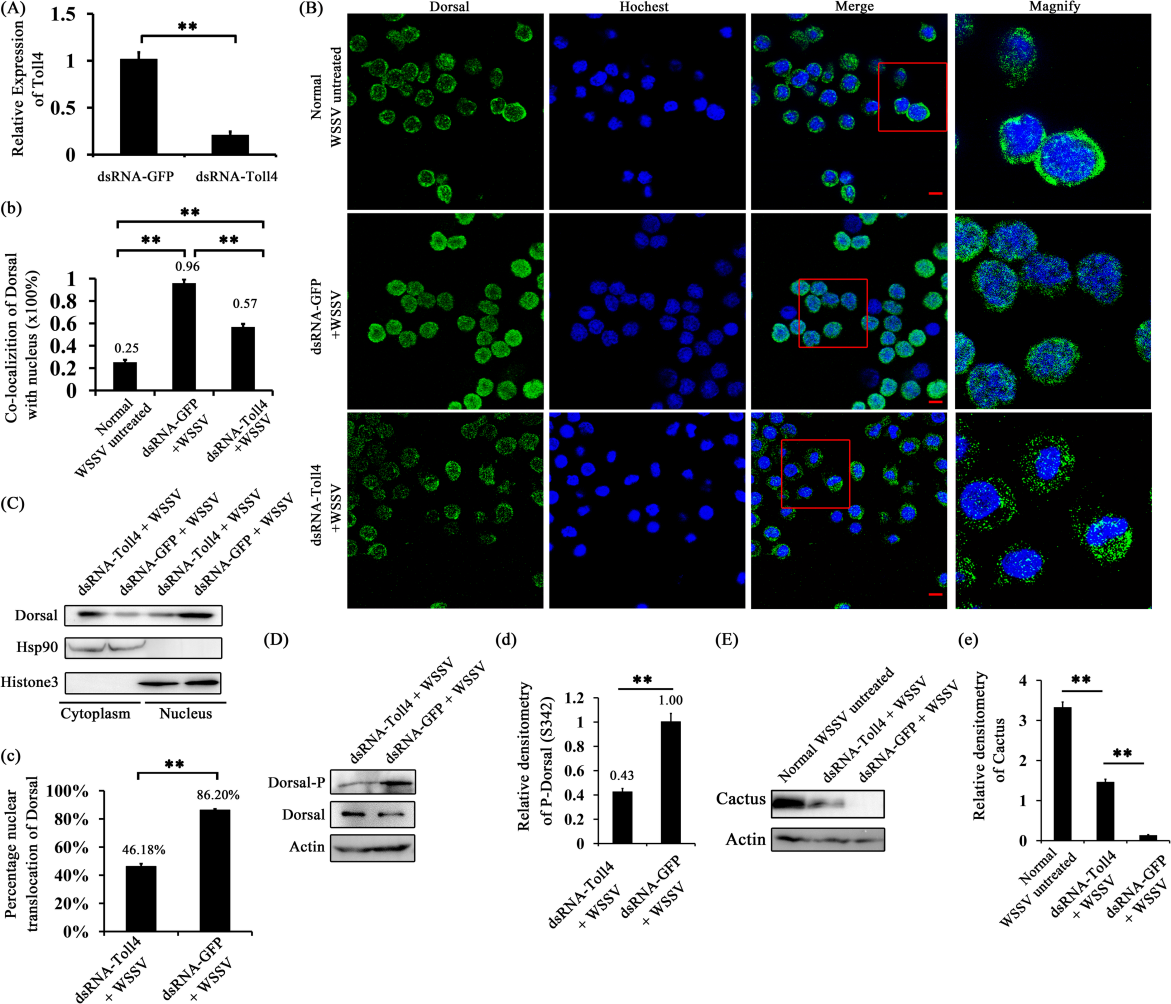
Toll4 regulates Dorsal activation in response to WSSV infection in shrimp. (A) Silencing efficiency for Toll4 in hemocytes was detected by qRT-PCR. (B) Dorsal nuclear translocation in Toll4 silenced hemocytes at 6 h post WSSV infection. (b) Statistical analysis of co-localization of Dorsal and nucleus in hemocytes by WCIF ImageJ software. (C) The subcellular distribution of Dorsal in Toll4 silenced hemocytes was detected at 6 h post WSSV infection by western blotting. The GFP dsRNA treated hemocytes was used as a control. (D) Dorsal phosphorylation level (Ser342) in Toll4 dsRNA and GFP dsRNA treated hemocytes was probed by western blotting. (E) Cactus protein levels were detected by western blotting using prepared anti-Cactus specific antibody in Toll4 dsRNA and GFP dsRNA treated hemocytes at 6 h post WSSV infection. The dsRNA untreated and WSSV non-challenged hemocytes were used as control. (c, d, e) Statistical analysis of the Dorsal nuclear translocation proportion, Dorsal phosphorylation levels and Cactus protein levels in Toll4 dsRNA and GFP dsRNA treated hemocytes at 6 h post WSSV infection, respectively. All experiments were performed three times, and similar results were observed. All the data from b, c, d and e were analyzed statistically by student’s T test (** *p* < 0.01).

**Fig 8:**
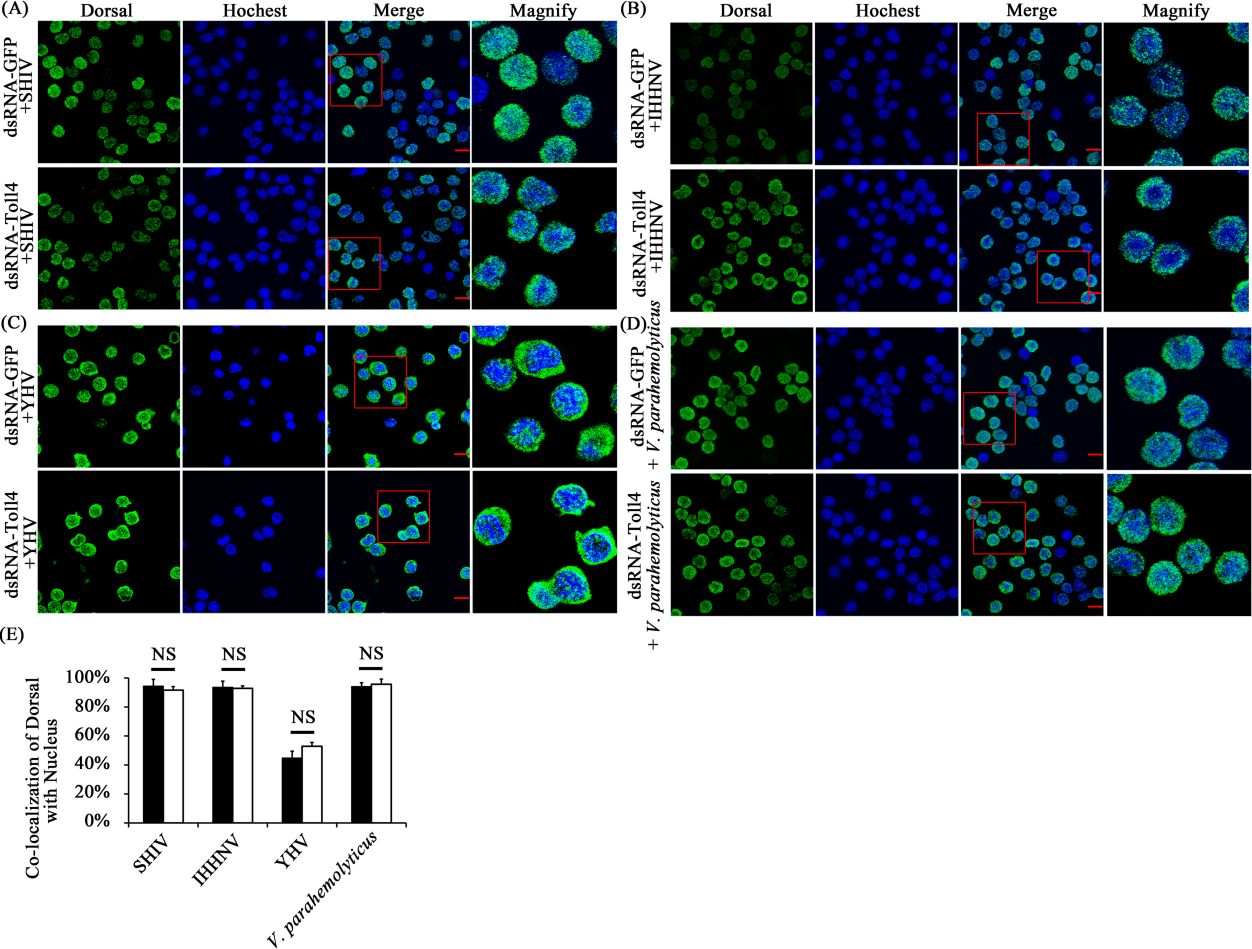
Knockdown of Toll4 doesn’t affect the translocation of Dorsal in response to other shrimp pathogens. (A-D) The immunocytochemical staning of Dorsal in Toll4 silenced hemocytes with challenges of SHIV (A), IHHNV (B), YHV (C) and *V*. *parahaemolyticus* (D) at 6 hpi as described earlier. The GFP dsRNA treated hemocytes following infection were used as a control. (E) Co-localization of Dorsal and Hochest-stained nucleus in hemocytes were calculated by WCIF ImageJ software and analyzed statistically by student’s T test (NS, not significant). All experiments were performed three times, and similar results were obtained.

Because both Toll4 and Dorsal induced the same AMPs expression under WSSV infection, we tested whether WSSV mediated Dorsal activation is dependent on Toll4. To assess this, RNAi was performed to evaluate the effects of Toll4 on Dorsal nuclear translocation and phosphorylation under WSSV infection *in vivo*. Efficient silencing of Toll4 mRNA was confirmed by quantitative RT-PCR (Fig 7A). At 6 hours after WSSV infection, but not the negative control (WSSV untreated, Fig 7B top panel), we were able to observe more nuclear imports of Dorsal in hemocytes of GFP dsRNA treated shrimps (96%) than those of Toll4 silenced shrimps (57%) (Fig 7B and b, *p* < 0.01). We confirmed these results by measuring Dorsal localization and phosphorylation after WSSV infection in hemocytes of GFP dsRNA and Toll4 dsRNA treated shrimps using WB analysis. A decreased percentage of nuclear translocation of Dorsal (46.18%) was observed in Toll4 silenced shrimps under WSSV infection compared to that of dsRNA GFP treated shrimps (86.20%) (Fig 7C and c, *p* < 0.01). Moreover, knockdown of Toll4 markedly reduced the WSSV induced phosphorylation of Dorsal when compared to GFP dsRNA treated shrimps (Fig 7D and d, *p* < 0.01). We next appraised the protein levels of shrimp Cactus, an inhibitor of shrimp Dorsal, by WB analysis using an *L*. *vannamei* Cactus specific antibody [49]. The results demonstrated that a very strong signal and a weak signal were detected in the negative control (WSSV untreated) and Toll4 dsRNA treated shrimps, respectively, but we failed to detect any signal of Cactus in the GFP dsRNA treated shrimps (Fig 7E and e, *p* < 0.01), which further confirmed that WSSV induced Dorsal activation was partially blocked in Toll4 silenced shrimps. To test whether Toll4 mediated Dorsal activation is pathogen specific, we tested its requirement to other shrimp pathogens including DNA viruses (infectious hypodermal and hematopoietic necrosis virus, IHHNV, and shrimp hemocyte iridescent virus, SHIV), RNA virus (yellow head virus, YHV) and bacteria (*V*. *parahaemolyticus*). The results showed that all the four types of pathogens could induce Dorsal activation with different degrees of nucleus translocations, but it seemed to be not relevant in the context of the silencing of Toll4 (Fig 8). The prominent nuclear translocation of Dorsal in hemocytes after *V*. *parahaemolyticus* challenge is in good concurrence with previous reports that G^-^ bacterial infection can induce the activation of Dorsal and its translocation [47, 51]. These analyses with different pathogens strongly suggest that Toll4 mediated Dorsal activation is WSSV specific, which indicate that Toll4 could play a vital role in recognizing WSSV infection. Taken together, our data suggest that Toll4 is a key factor for sensing WSSV and mediating downstream Dorsal activation, and thereby inducing some specific AMPs production.

### AMPs regulated by Toll4-Dorsal pathway oppose WSSV infection

**Fig 9:**
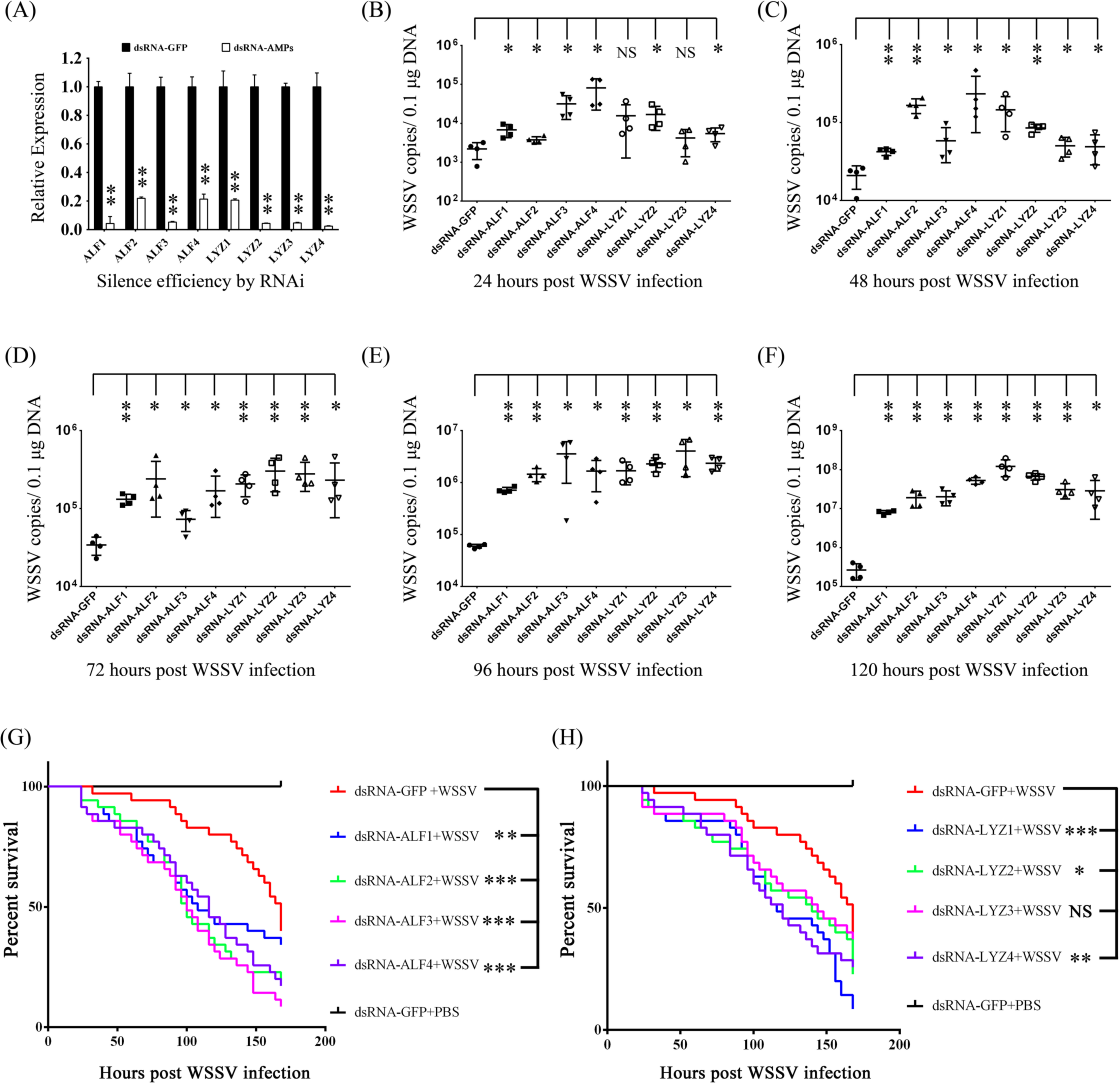
The function of Toll4-Dorsal cascade regulated AMPs in WSSV infection. (A) Effective knockdown for ALF1-4 and LYZ1-4 in hemocytes by dsRNA was confirmed by qRT-PCR. (B-F) Silencing of AMPs enhanced WSSV infection in shrimp. WSSV was inoculated at 48 h post each AMP silencing. The viral loads in gills were assessed at 24 h (B), 48 h (C), 72 h (D), 96 h (E) and 120 h (F) post-infection via absolute qPCR. (G-H) Survival of WSSV challenged AMP-silenced shrimp and GFP dsRNA treated shrimp. All experiments were performed three times, and similar results were obtained. All the data from B, C, D, E and F were analyzed statistically by student’s T test (NS, not significant; * *p* < 0.05; ** *p* < 0.01). Survival rates from G and H were analyzed statistically by the Kaplan–Meier plot (log-rank χ^2^ test) (* *p* < 0.05, ** *p* < 0.01, *** *p* < 0.001, NS, not significant).

The induction of AMPs as a response to pathogenic infection is a crucial defense mechanism of innate immunity in invertebrates including insects and shrimps. Our results above have revealed that after WSSV infection Toll4 and Dorsal induced the same AMPs expression, raising the hypothesis of these AMPs playing antiviral role against WSSV. We silenced the eight AMPs including ALF1-4 and LYZ1-4 regulated by both Toll4 and Dorsal through dsRNA treatment. Silence efficiencies were confirmed by quantitative RT-PCR (Fig 9A). To gain more information about the effects of AMPs on viral replication, a timeframe of 24 h, 48 h, 72 h, 96 h and 120 h after WSSV infection was selected to investigate the viral loads in gills of each AMP silenced shrimp. Compared with GFP dsRNA inoculated shrimps, shrimps in which ALF1, ALF2, ALF3, ALF4, LYZ1, LYZ2, LYZ3 or LYZ4 were silenced had higher viral burden (Fig 9B, 9C, 9D, 9E and 9F) at the whole timeframe. To further dissect the function of these AMPs during WSSV infection, a parallel experiment was performed to explore the survival phenotype of each AMP silenced shrimps followed by WSSV infection. Experimental shrimps were challenged with WSSV at 48 h post dsRNA injection, and the survival rate was recorded over a period of 168 h after the challenge. We observed that in each AMP-knockdown group except LYZ3-knockdown group, survival rate was lower than that of control group and shrimps were more susceptible to WSSV infection (Fig 9G and 9H). Notably, the survival rate of LYZ3-knockdown group showed no significant difference, but had the trend of lower (p = 0.3853) compared to that of control group (Fig 9H). In summary, our data convincingly demonstrate that Toll4-Dorsal pathway regulated AMPs are involved in WSSV restriction in shrimp.

### AMPs exhibit antiviral activity by interacting with WSSV structural proteins

**Fig 10:**
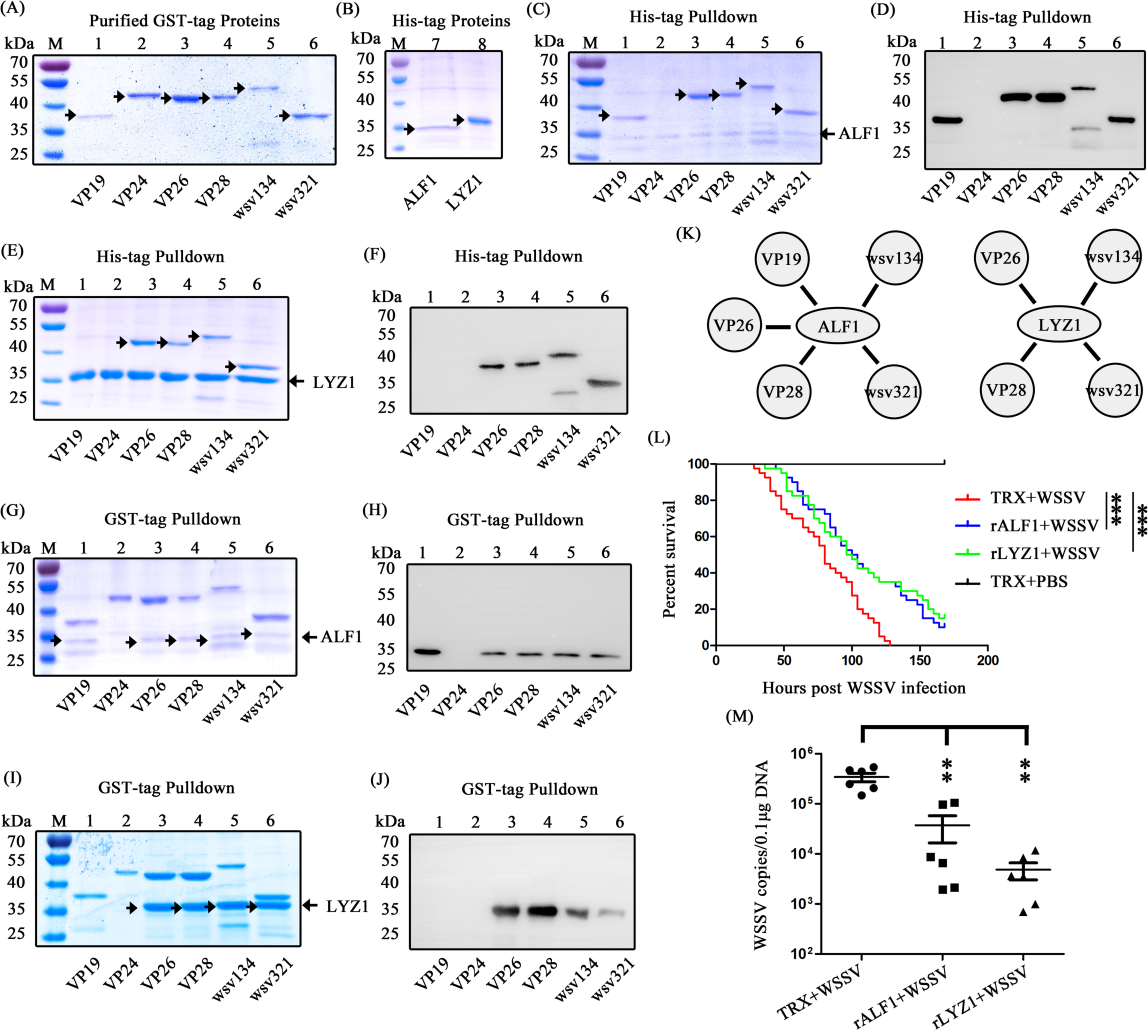
AMPs interact with WSSV structural proteins to inhibit viral infection. (A) Purified GST tagged WSSV structural proteins of VP19, VP24, VP26, VP28, wsv134 and wsv321. (B) Purified His tagged ALF1 and LYZ1. (C) His tagged ALF1 interacted with GST-VP19, VP26, VP28, wsv134 and wsv321 was obtained in His pull-down assay and visualized by coomassie blue staining. (D) His-ALF1 interacted with GST-VP19, VP26, VP28, wsv134 and wsv321 in His pull-down assay was confirmed by western blotting with anti-GST antibody. (E) His tagged LYZ1 interacted with GST tagged VP26, VP28, wsv134 and wsv321 was obtained in His pull-down assay and visualized by coomassie blue staining. (F) His pull-down assay with His-LYZ was confirmed by western blotting with anti-GST antibody. (G) GST tagged VP19, VP24, VP26, VP28, wsv134 and wsv321 were used to pull down His-ALF1, and visualized by coomassie blue staining. (H) GST pull-down assay of GST-VP19, VP26, VP28, wsv134 and wsv321 interacting with His-ALF1 was confirmed by western blotting with anti-His antibody. (I) GST tagged VP19, VP24, VP26, VP28, wsv134 and wsv321 were used to pull down His-LYZ1, and visualized by coomassie blue staining. (J) GST pull-down assay of VP26, VP28, wsv134 and wsv321 interacting with His-LYZ1 was confirmed by western blotting with anti-His antibody. (K) Schematic illustration of the ALF1 or LYZ1 interacting with WSSV structural proteins. The experiments were repeated three times. (L) Survival of viral infected shrimps by co-incubation of WSSV with ALF1 or LYZ1 or TRX (as a control). Survival rates from L were analyzed statistically by the Kaplan–Meier plot (log-rank χ^2^ test) (*** *p* < 0.001). (M) Co-incubation of WSSV with ALF1 or LYZ1 inhibited viral replication in shrimps. The viral loads in gills were assessed at 96 h post-infection via absolute qPCR. All experiments were performed three times, and similar results were obtained. The data from M were analyzed statistically by student’s T test (** *p* < 0.01).

Some AMPs have been reported to play a vital role in combating viral pathogens via directly acting on the viral virion [52, 53]. To decipher the molecular basis underlying AMPs against WSSV, we performed an *in vitro* pull-down assay between AMPs and WSSV structural proteins in order to elucidate how these AMPs act on WSSV. In this study, we paid our attention to ALF1 and LYZ1 as a representative one of ALF and LYZ families, respectively. Several envelope and tegument proteins of WSSV such as VP24, wsv134 (WSSV189) and wsv339 (WSSV395) have been proved to be the target of AMPs such as ALFs [54–56]. In addition, VP19, VP28, VP24 and VP26 are abundant in WSSV virion [15, 57], and they are always be targeted by other antiviral effectors such as Lectins [58–60]. So, four envelope proteins (VP19, VP28, wsv134 and wsv321 (VP16)) and two tegument proteins (VP24 and VP26) for the potential target of AMPs were used in the *in vitro* pull-down assay to explore the potential interaction between these structural proteins and ALF1 or LYZ1. The six WSSV structural proteins with GST tag and the two AMPs ALF1 and LYZ1 with His tag were expressed and purified (Fig 10A and 10B). In the His tagged ALF1 pull-down assay with six WSSV structural proteins (GST tag), we observed that ALF1 precipitated VP19, VP26, VP28, wsv134 and wsv321 by SDS–PAGE with coomassie blue staining (Fig 10C, lanes 1, 3, 4, 5 and 6, respectively). However, His tagged ALF1 did not interact with GST tagged VP24, which indicates that the interaction of between ALF1 and other four structural proteins is specific, but not related to the His and GST tags. We further confirmed this result by western blotting with GST tag antibody, which is in good agreement with that of coomassie blue staining (Fig 10D). In the His tagged LYZ1 pull-down assay, we found that VP26, VP28, wsv134 and wsv321 were enriched (Fig 10E, lanes 3, 4, 5 and 6, respectively), and an identical result was observed by western blotting (Fig 10F). To further identify the above results, six WSSV structural proteins with GST tag were used in a GST pull-down assay with purified His tagged ALF1 or LYZ1 followed by SDS-PAGE with coomassie blue staining and western blotting with His antibody, respectively. As shown in Fig 10G and 10H, VP19, VP26, VP28, wsv134 and wsv321 interacted with ALF1 (arrows) in GST pull-down assay, which further confirmed the results of pull-down assay with His tagged ALF. In a similar manner, Fig 10I and 10J showed that VP26, VP28, wsv134 and wsv321 strongly interacted with LYZ1. Thus, the results strongly suggest that ALF1 and LYZ1 were able to interact with WSSV structural proteins, to be specific, ALF1 interacted with VP19, VP26, VP28, wsv134 and wsv321, and LYZ1 interacted with VP26, VP28, wsv134 and wsv321 (Fig 10K). To explore whether other members of ALF and LYZ family also exhibit the ability to interact with WSSV structural proteins, we additionally expressed and purified ALF3 and LYZ2 His-tag fused proteins (S4A Fig). In the His pull-down assay, we observed that VP24 and VP26 was precipitated by ALF3, and VP24, VP26, VP28 and wsv134 was precipitated by LYZ2, respectively (Figs S4B and 4C). The results demonstrated that it could be a general action by which Toll4-Dorsal cascade targeted ALFs and LYZs to interact with WSSV structural proteins.

To further confirm the antiviral activities of ALF and LYZ family proteins, we performed a viral infection experiment by prior co-incubation of WSSV with recombinant ALF1 (rALF1) and LYZ1 (rLYZ1). As shown in Fig 10L and 10M, the shrimps had reduced viral loads and showed more resistance against WSSV in the recombinant proteins co-incubation treated groups compared to TRX control group. Taken together, our results provide some substantial evidences to demonstrate that Toll-Dorsal pathway targeted AMPs exhibit antiviral activity by interacting with WSSV structural proteins.

## Discussion

Accumulating evidence indicates that shrimp Tolls participate in host defense against WSSV infection; however, the underlying mechanism of the Toll receptor mediated antiviral functions has been poorly understood. Herein, we have identified an antiviral role for a new Toll from *L*. *vannamei*, the Toll4, in response to WSSV infection *in vivo*. Toll4 silenced shrimps demonstrate significantly elevated viral replication and mortality after WSSV challenge. Shrimps with knockdown of genes in some core components of the canonical Toll pathway such as MyD88, Tube, Pelle and Dorsal have remarkably increased WSSV titers. Furthermore, Toll4 appears to be specific to sense WSSV infection to trigger Dorsal, which lead to induce a specific set of AMPs with the ability of interacting with viral structural proteins that confer resistance to viral infection. Our results have now demonstrated that the Toll4-Dorsal-AMPs cascade is involved in the control of WSSV infection in shrimp.

Of note, a total of nine Tolls (Toll1-9) have been identified from *L*. *vannamei*, but we can’t identify their corresponding orthologs to TLRs in mammals. As phylogenetic tree analysis shown, we find that nearly all of Tolls from invertebrates are clustered together, while vertebrates TLRs can separate into several groups such as TLR1 to TLR10, TLR12 and TLR15 (S2 Fig). Such results could be due to the following aspects: (i) Invertebrate Tolls display little conservation with vertebrate TLRs; (ii) The number of discovered Tolls from invertebrates is limited. As a result, we can’t draw a conclusion that Toll4 from *L*. *vannamei* is the ortholog of TLR4 in mammals. Therefore, the Tolls are named as Toll1 to Toll9 according to the time order of their being identified, like *Drosophila* Toll1 to Toll9 [61–65].

The Toll pathway is essential to establish an innate immune response to defend against a wide range of pathogens including virus. The importance of this pathway in the innate control of viral infections in insects is best demonstrated by that mutants in some core components such as Dif and Toll of *Drosophila* with increased susceptibility to infection [10]. The *Drosophila* Toll pathway has also been shown to play an universal antiviral role against multiply viruses by oral infection such as *Drosophila* C virus, Cricket paralysis virus, Flock house virus, and Nora virus [66]. In addition, several reports show that the Toll pathway has an antiviral role in innate immunity of mosquitoes [67–69]. Our results indicate that the increased lethality rates observed in the Toll4 silenced shrimps are associated with higher WSSV loads. Thus, the Toll4 is involved in resistance to WSSV and it is a major antiviral factor in shrimp. Moreover, several Tolls have been proved to confer antiviral immunity in other shrimps. For example, a Toll4 from *P*. *clarkii* [29] and a Toll from *M*. *rosenbergii* [31] are important for the innate immune responses against WSSV, although the exact antiviral mechanism is not elucidated. We also provide evidence indicating the key antiviral role of the canonical Toll pathway by that silencing of the core components such as MyD88, Tube, Pelle and Dorsal results in increased WSSV titers. These data may suggest that the function of Toll pathway in the control of viral infections could be conserved through evolution. This is consistent with previous studies showing Toll pathway antiviral effect in other Arthropods including crayfish [28], *Drosophila* [10, 66], mosquitoes [70] and honeybees [67].

In general, the canonical Toll pathway of *Drosophila* mediated immune response relies on the activation of the NF-κB transcription factors Dorsal or Dif, however whether it is true for shrimp is still largely unknown. Firstly, we demonstrate that Toll4 and Dorsal are involved in regulating the same AMPs after WSSV infection. In addition, our data show that detection of WSSV infection by Toll4 triggers transcriptional activity of Dorsal, but knockdown of Toll4 was not sufficient to restrain the activation of Dorsal in response to WSSV infection. This may be owing to inability to absolutely suppress Toll4 expression by RNAi method. But we cannot exclude the possibility that the activation of Dorsal in response to WSSV infection may integrate signals from other upstream receptors. In other words, there could be more than one upstream receptor, in addition to Toll4, involved in the response to WSSV infection responsible for the activation of Dorsal. Of note, Dorsal has the ability to bind with the promoters of some WSSV genes such as the Immediate Early gene 1 (IE1) and regulate their transcriptional expression in insect cells background or *in virto* [71]. Thus, it is thought that Dorsal is required for WSSV gene expression and genome replication [72], more experiment evidence *in vivo* needs to support this. By RNAi method, we observe that shrimps with knockdown of Dorsal have elevated viral loads than those of the GFP control group, suggesting that Dorsal is important for host to limit viral replication. Besides, the report of WSSV encoding two MicroRNAs with the ability to suppress shrimp Dorsal also supports that Dorsal is a key restrict factor against viral infection [73]. However, knockdown of Dorsal results in lower viral loads than those of MyD88, Tube and Pelle silenced groups, which may be explained by that Dorsal locates the lower levels at MyD88/Tube/Pelle/Dorsal cascade of the canonical Toll pathway.

Some reports have showed that Tolls from *Drosophila* and shrimps inducing antiviral innate immunity are independent of activation of the transcription factor NF-κB (Dorsal or Dif), as shown by the fact that *Drosophila* Toll7 activates antiviral autophagy not involvement of Dorsal or Dif [11], as well as by the fact that shrimp Toll3 initiates a IRF-Vago dependent antiviral route [33]. Therefore, whether other Tolls are responsible for activation of Dorsal in response to WSSV infection, and how they confer resistance to WSSV infection deserve to be further studied. In addition, in the present study, we identified a total of nine Tolls, and silencing of each of Toll except for the Toll2 contributes to increased WSSV loads compared to the control shrimps. This observation is reminiscent of that the lack of immunoglobulin-based adaptive immune system and classical IFN mediated antiviral defense maybe require some invertebrates including shrimps and *Drosophila* to be more heavily dependent on the Tolls or other receptors for antiviral immunity. Identification of the target genes of the other Tolls after viral infection will be important to understand how they contribute to resistance to viruses.

There are significant differences in the Toll and TLR receptors initiated activation by ligand recognition in invertebrate and vertebrate. In general, TLR receptors in mammals are able to detect microbial infection through directly binding to PAMP [2], but *Drosophila* Toll1 instead interacts with the endogenous cytokine-like factor Spätzle, the product of a proteolytic cascade induced upon upstream recognition of fungal and bacterial PAMPs [68]. Notably, Toll7 in *Drosophila* can bind to vesicular stomatitis viruses at the plasma membrane and therefore has been considered as a specific and bona fide PRR for sensing this virus [11]. Interestingly, several Tolls from *L*. *vananmei* are able to detect some PAMPs directly, as shown by the fact that Toll1 and Toll3 can interact with CpG ODN 2395 *in vitro* [74]. Surprisingly, another study demonstrates that three Tolls from *M*. *japonicas*, two of them homologous to the above Toll1 and Toll3 from *L*. *vananmei* [74], can directly bind to both PGN and LPS [51]. These data suggest that one type of shrimp Toll could recognize more than one PAMP. In the present study, we observed that Dorsal activation and translocation to the nucleus is dependent on Toll4 in response to WSSV infection, but not other tested pathogens, which indicates that Toll4 could also be a specific PRR to detect WSSV in a manner similar to *Drosophila* Toll7. Unravelling how Toll4 senses WSSV in the future will be important to understand antiviral immunity in shrimp.

The production of antimicrobial peptides (AMPs) is commonly considered to be an evolutionarily conserved mechanism of the innate immune response and has been extensively studied in vertebrates and other non-vertebrate organisms including shrimps. Some shrimp Tolls are able to resist bacterial infection via regulating a wide range of AMPs expression [28, 29, 75], which inspires us to suppose that shrimp Tolls can also regulate some specific AMPs synthesis to oppose WSSV. In fact, our examinations of AMPs expression in WSSV-infected shrimp show an increase in expression of AMPs comparable to that found during a *V*. *parahaemolyticus* infection of shrimp. Moreover, the decreased expression of the same AMPs in Toll4 and Dorsal-silenced shrimp does show that Toll4-Dorsal pathway indeed devotes to induce AMPs transcription upon WSSV infection. By RNAi, we detect survival rates and viral titers in single AMPs silenced shrimps and find that each single AMP except for LYZ3 provides effective resistance to viral infection. Although evidence exists that some AMPs can respond to WSSV infection, it was not known how these shrimp AMPs affected viruses. Previous reports show that shrimp ALF can protect against WSSV infection via interfering with viral replication *in vitro* and *in vivo* in crayfish *Pacifastacus leniusculus* [76] and CqALF can disrupt WSSV envelope integrity that leads to the decrease of WSSV infectivity in the red claw crayfish *Cherax quadricarinatus* [77]. Furthermore, an ALF isoform 3 from *P*. *monodon* has performed its anti-WSSV action by binding to several viral structural proteins such as wsv131 (WSSV186), wsv134 (WSSV189) and wsv339 (WSSV395) [78]. On the other hand, lysozyme is a key effector of the innate immune system and kills bacteria by catalytic hydrolysis of cell wall peptidoglycan, but it also exhibits catalysis-independent antimicrobial properties. For example, human lysozyme has been shown to inhibit HIV-1 infection *in vitro* by preventing the adsorption and penetration of the virus [79, 80]. HL9, a nonapeptide fragment of human lysozyme, blocks HIV-1 viral entrance and replication by binding to the ectodomain of gp41, the envelope glycoprotein of HIV-1 crucial to membrane fusion [80, 81]. These data strongly suggest that ALF and LYZ family have effective antiviral activity, and it seems reasonable to hypothesize that shrimp ALF and LYZ family are able to interact with WSSV structural proteins. In this study, one anti-LPS-factor ALF1 and one Lysozyme LYZ1 are chosen to explore their antiviral actions. In agreement with this hypothesis, our results reveal that ALF1 interacts with VP19, VP26, VP28, wsv134 and wsv321, while LYZ1 interacts with VP26, VP28, wsv134 and wsv321. Given their conserved sequences, the other AMPs, in addition to ALF1 and LYZ1, could be able to interact with some specific WSSV structural proteins. Interestingly, currently, no mechanistic analysis on LYZ family genes responsible for antiviral role against WSSV has been performed except for a role in modulating the humoral response to this virus infection [82]. Thus, LYZ1 in this study is the first LYZ family gene identified with the capacity to interfere with replication of this important pathogen, which suggests that LYZ could be a new type of effectors for restricting WSSV infection. Of note, one type of AMP can interact with more than one WSSV structural protein, and vice versa. Likewise, WSSV structural proteins VP19, VP26 and VP28 are shown to interact with each other to form a multiprotein complex [83]. So, it seems that AMPs interact with WSSV structural proteins as a manner of multiply layers or reticulation, which could be more effective to control virus. In addition to their important functions in maintaining the integrity of virion, WSSV structural proteins also play a key role in initiating viral infection [84], as showed by fact that some structural proteins such as VP28 and VP26 are shown to being key factors essential for virus attachment and entry into host cells [84–87]. Therefore, based on our data together with previous reports, it is highly conceivable that the Toll4-Dorsal pathway regulated AMPs interacts with WSSV structural proteins to both disrupt WSSV integrity and interfere with viral invasion.

**Fig 11:**
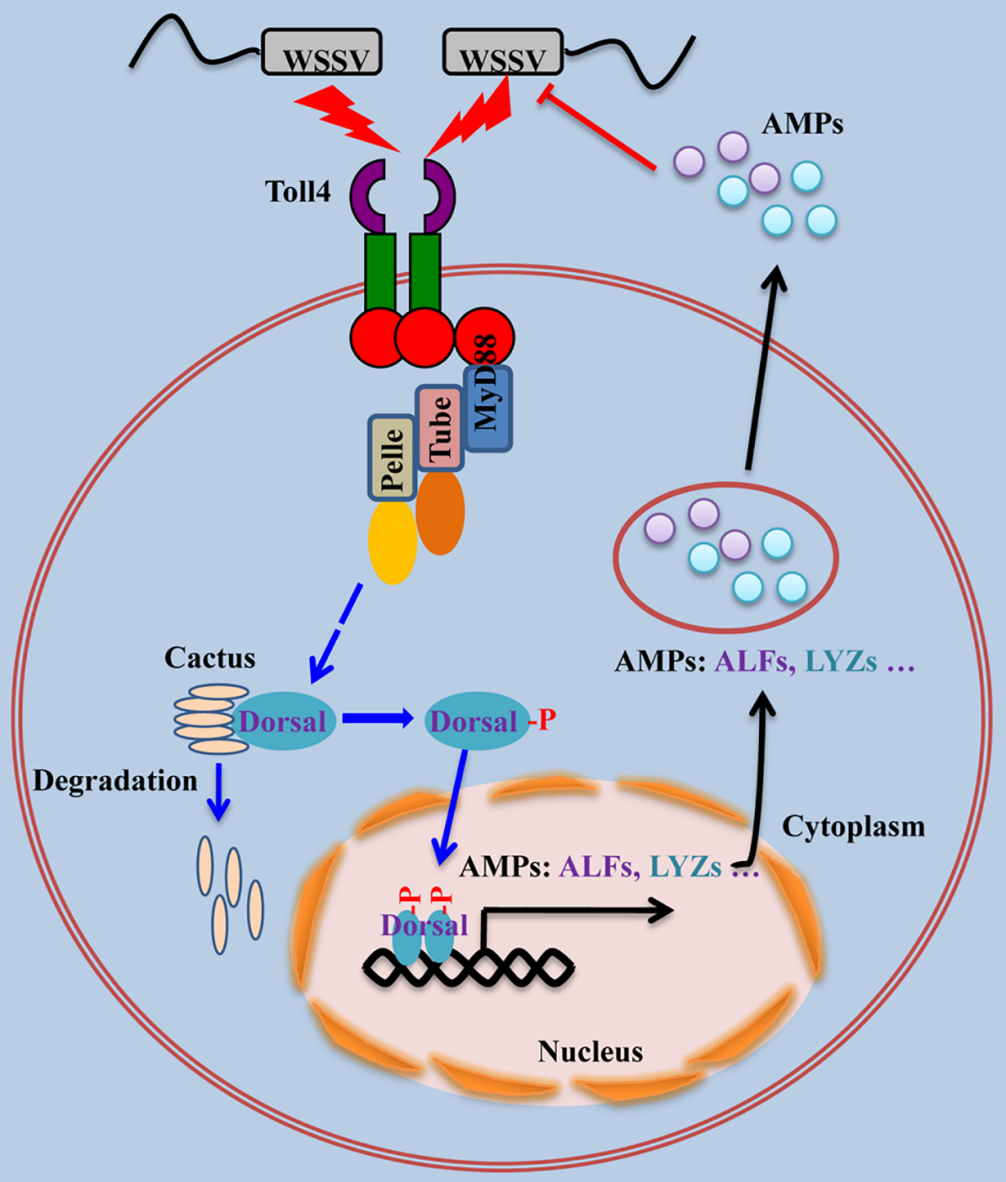
Model for Toll4 mediated antiviral mechanism against WSSV. In shrimp, Toll4 sensed WSSV infection and proceeded to the degradation of shrimp IκB factor Cactus. After Cactus degradation, the transcription factor Dorsal was phosphorylated and translocated into the nucleus, where it led to activation of the transcription of several sets of AMPs (ALF1, ALF2, ALF3, LYZ1, LYZ2 and LYZ4). These effector molecules (AMPs) were secreted to extracellular space and executed anti-WSSV activity through interacting with its structural proteins.

Toll-Dorsal-AMPs pathway is quite clear in *Drosophila*. Gram-positive or fungal infection trigger the activation of Toll-Dorsal-AMPs pathway, which lead to the systemic production of Drosomycin and Metchnikowin [88]. Although the role of Toll-Dorsal-AMPs pathway against bacterial infection in some invertebrates such as *Drosophila* and shrimps has been reported, there is little information about its antiviral role. In our paper, we clone and identify a total of nine Tolls from *L*. *vannamei*, RNAi screens Toll4 as a key antiviral factor against WSSV infection. Considering the data obtained in the present study, we propose the following antiviral immune signaling pathway in *L*. *vannamei* (Fig 11): i. viral recognition and signal transduction: Toll4 recognizes WSSV infection to converge on Dorsal translocated from the cytoplasm to the nucleus and phosphorylation on Ser342; ii. AMP induction: the activation of Dorsal in the nucleus triggers a specific set of AMPs expression such as ALFs and LYZs; and iii. viral inactivation: Toll4-Dorsal drived AMPs can bind with the components of the viral surface, subsequently resulting in the WSSV inactivation. Uncovering the Toll4-Dorsal-AMPs cascade mediated antiviral program may provide novel strategies for limiting WSSV infection in shrimp aquaculture, and dissecting the pattern of Toll4 sensing WSSV in the further will provide additional insights into how the canonical Toll pathway responds to viral infection.

## Materials and methods

### Animals and pathogens

Shrimps (*L*. *vannamei*, average weight 8 g each) were purchased from the local shrimp farm in Zhuhai, Guangdong Province, China, and fed with a commercial diet in a recirculating water tank system filled with air-pumped sea water (2.5% salinity) at 28°C. Before all experiment treatments, the shrimps (5% of total) were detected and confirmed to be free of common pathogens including white spot syndrome virus (WSSV), yellow head virus (YHV), taura syndrome virus (TSV), shrimp hemocyte iridescent virus (SHIV, also known as CQIV), infectious hypodermal and hematopoietic necrosis virus (IHHNV) and *Vibrio parahaemolyticus* by PCR or RT-PCR methods according to standard operation procedures by *Panichareon* et al [89] and *Qiu* et al [90]. Because many genes from the shrimp canonical Toll-Dorsal pathway can be activated by Gram-negative (G^-^) bacteria [47, 51], *V*. *parahaemolyticus* thus was used here as a positive activator of the shrimp Toll-Dorsal pathway. The Gram-negative bacteria *V*. *parahaemolyticus* were cultured in Luria broth (LB) medium overnight at 37°C, and the bacteria were harvested by centrifugation (5000 *g*, 10 min) and washed twice in phosphate buffer saline (PBS) to remove growth medium and finally resuspended in PBS to give 10^8^ cells per ml. A final injection density of *V*. *parahaemolyticus* was adjusted to yield approximately 1 × 10^5^ CFU/50 μl as a previous study [91]. WSSV was extracted from the WSSV-infected shrimp muscle tissue and stored at -80°C. Before injection, muscle tissue from WSSV infected shrimp was homogenized and prepared as WSSV inoculum with approximately 1 × 10^5^ copies in 50 μl PBS following a published method [92]. The SHIV-infected shrimp samples of cephalothoraxes were homogenized in PPB-Tris buffer (376.07 mM NaCl, 6.32 mM K_2_SO_4_, 6.4 mM MgSO_4_, 14.41 mM CaCl_2_, and 50 mM Tris-HCl, pH 7.0) and clarified at 9000 *g* for 10 min to gather the supernatant. The pellet was homogenized in PPB-Tris buffer again and clarified at 9000 *g* for 5 min, repeated three times and the supernatants were combined every time [93]. The supernatant was collected as the original stock, and diluted and adjusted to approximately 1 × 10^5^ copies in 50 μl PBS before injection. Because gills were the main target of IHHNV, the viral stock was prepared from IHHNV-infected shrimps gill. The gills were homogenized in PBS buffer and centrifuged at 9000 *g* for 10 min at 4°C [94]. The supernatant was collected as the original stock, and diluted and adjusted to approximately 1 × 10^5^ copies in 50 μl PBS before injection. The YHV stock was prepared from the gills of moribund shrimp showing clinical signs of yellow head disease. The gills of the infected shrimp were ground and further suspended in NTE buffer (0.02 M EDTA, 0.2 M NaCl, 0.2 M Tris-HCl, pH 6.5). The solution was filtered using a 0.22-μm MILLEX HP Fillter Unit and centrifuged at 9000 *g* for 10 min at 4°C [95]. The supernatant was collected as the original stock, and diluted and adjusted to approximately 1 × 10^5^ copies in 50 μl PBS before injection.

In the pathogenic challenge or immunocytochemical staning experiments, each shrimp was received an intraperitoneal injection of 50 μl WSSV, SHIV, IHHNV, YHV or *V*. *parahaemolyticus* solution at the second abdominal segment by a 1-ml syringe.

### Cloning of shrimp Tolls

In order to obtain the cDNA sequence of all candidate Toll genes from shrimp, the amino acid (aa) sequences of the Tolls and TLRs from *Drosophila* and human (DmToll1-9 and HsTLR1-10) were collected and used as query sequences for in silico searches of *L*. *vannamei* transcriptome data [34] using local TBLASTN alignment tool with E-value cutoff of 1e^-5^. Nine assembled EST sequences were identified as having high homology to the Toll family genes. Gene-specific primers (S1 Table) were designed for 5′ and 3′ rapid amplification of cDNA ends (RACE) PCR to obtain the 5′ and 3′ end of *L*. *vannamei* Toll genes. In brief, total RNA was extracted from pooled tissues of *L*. *vannamei* gill, hemocyte and intestine followed by the protocol described in the RNeasy Mini Kit (Qiagen). cDNA synthesis, 5′/3′-rapid amplification of cDNA ends (5′/3′-RACE) PCR, and nested PCR were performed using a SMARTer RACE cDNA amplification kit (Clontech, Japan) in accordance with the manufacturer's instructions. The final PCR products were cloned into pMD-19T Cloning Vector (TaKaRa, Japan) and 12 positive clones were selected and sequenced. Then, we performed TBLASTN again by using the aa sequences of nine Tolls as query sequences to search against several RNA-Seq databases from NCBI and others [35–38], but no new Toll was identified, suggesting there could be just a total of nine Tolls in shrimp.

### Sequence and phylogenetic analysis

Protein domains of Tolls and TLRs were identified by using Simple Modular Architecture Research Tool (SMART) (http://smart.embl.de/). Shrimp TIR domains of nine Tolls were aligned by using Clustal X v2.0 program [96] and GeneDoc software where the identities among each other were labeled. The neighbor-joining (NJ) phylogenic tree was constructed based on the deduced amino acid sequences of Tolls and TLRs (Supplement Data S3) by utilizing MEGA 5.0 software [97].

### Antibodies

The polyclonal antibodies for *L*. *vannamei* Dorsal and Cactus were produced in guinea pigs and rabbits, respectively, by GL Biochem antibody manufacturing company (China) from our previous study [49]. Polyclonal rabbit anti-NF-κB p65 (phospho S276) antibody (ab194726, Abcam) was used to detect the phosphorylated shrimp Dorsal [51]. Rabbit anti-Histone H3 (4499s), Rabbit anti-Hsp90 (ab13495), and the secondary antibodies Goat Anti-Guinea pig IgG H&L (Alexa Fluor 488) (ab150185), Goat anti-Guinea pig IgG H&L (HRP) (ab6908), anti-Mouse IgG H&L (HRP) (ab6789) and anti-Rabbit IgG H&L (HRP) (ab6721), were purchased from Abcam (USA). Mouse anti-Actin antibody was obtained from Merck (MAB1501). Mouse anti-6His antibody (H1029) and Mouse anti-GST antibody (SAB4200237) were gained from Sigma (USA).

### Detection of viral loads by absolute quantitative PCR

In order to monitor the WSSV copies, absolute quantitative PCR (qPCR) was conducted by utilizing with the forward and reverse primers of wsv069 (WSSV32678-F/WSSV32753-R), a WSSV single copy gene, and a TaqMan fluorogenic probe (WSSV32706) followed by a published method [72]. The primers used here were shown in S1 Table. In brief, a 675-bp DNA amplicon of wsv069 with a region of 32678 to 32753 from WSSV genome (AF332093.2) was obtained and subcloned into the pMD19-T plasmid. The plasmid pMD19-T containing the 675-bp DNA fragment was used as the internal standard, and serially diluted to 10-folds to generate a standard curve of absolute qPCR. Genomic DNA from shrimp muscle, hepatopancreases and/ or gill was extracted with Marine Animal Tissue Genomic DNA Extraction Kit (TianGen Biochemical Technology). The extracted shrimp DNA and the internal standard plasmid were subjected to absolute qPCR. The PCR reaction mixture and cycling conditions were the same as previous research [72]. Each sample from one shrimp was made in three replicates by absolute qPCR. The WSSV genome copies were calculated and normalized to 0.1 μg of shrimp tissue DNA.

### Semi-quantitative and quantitative reverse transcription PCR

Semi-quantitative reverse transcription PCR (Semi-qRT-PCR) was used to analysis the tissue distribution of nine Tolls in uninfected shrimp. Briefly, healthy shrimp tissues including hepatopancreases, gill, intestine, hemocyte, stomach, epithelium, heart and muscle were sampled. Three samples from each tissue were collected from 15 shrimps (5 shrimps pooled together). Total RNA was extracted from each tissue with RNeasy Mini Kit (Qiagen), and reverse transcribed to cDNA with PrimeScript II 1st Strand cDNA Synthesis Kit (Takara) following the manufacturer's instructions. The cDNA fragments of nine Tolls were amplified using the gene specific primers (S1 Table) under the following conditions: 1 cycle of 94°C for 2 min, 28 cycles of 94°C for 30 s, 60°C for 30 s, 72°C for 30 s, followed by elongation at 72°C for 5 min. As an internal loading control, the shrimp EF1α (GU136229) was amplified as the same PCR conditions.

Quantitative reverse transcription PCR (qRT-PCR) was conducted to detect the mRNA levels of genes (Tolls, Toll pathway components or AMPs) under the pathogenic challenge experiments or RNAi *in vivo*. The method of tissues collection, total RNA extraction and cDNA synthesis was as described above. qRT-PCR analysis was performed in the LightCycler 480 System (Roche, Germany) with a volume of 10 μl comprised of 1 μl of 1:10 cDNA diluted with ddH_2_O, 5 μl of 2 × SYBR Green Master Mix (Takara, Japan), and 250 nM of each primer (S1 Table). The cycling programs were the following parameters: 95°C for 2 min to activate the polymerase, followed by 40 cycles of 95°C for 15 s, 62°C for 1 min, and 70°C for 1 s. Cycling ended at 95°C with 5°C/s calefactive velocity to create the melting curve. Expression level of each gene was calculated relative to internal control gene EF-1α by using the Livak (2^-ΔΔCT^) method.

### DsRNA production and RNAi performance

The dsRNAs including Dorsal (accession No. ACZ98167), Tube (KC346865), Pelle (KC346864), MyD88 (AFP49302), nine Tolls (Supplement data S1), eight AMPs (Supplement data S2) and the control GFP, were synthesized by T7 RiboMAX Express RNAi System kit (Promega, USA) followed by the user's manual. More detailed information about the primers for dsRNA synthesis was listed in S1 Table. The quality of dsRNA was checked after annealing via gel electrophoresis. The RNA interference (RNAi) assay was performed as we described else [91]. Briefly, each shrimp was received an intraperitoneal injection at the second abdominal segment of dsRNAs (20 μg) for Dorsal, Tube, Pelle, Toll1, Toll2, Toll3, Toll4, Toll5, Toll6, Toll7, Toll8, Toll9, ALF1, ALF2, ALF3, ALF4, LYZ1, LYZ2, LYZ3, LYZ4 or GFP (as a control). The gills and/ or hemocytes were collected from the shrimp 48 h after the dsRNA injection, and total RNA was extracted and assessed by qRT-PCR using the corresponding primers (S1 Table) to evaluate the efficacy of RNAi.

To screen potential Toll with antiviral effects against WSSV, shrimps were divided into ten groups: one control group received GFP dsRNA injection and the other nine RNAi groups received each Toll dsRNA injection, respectively. At 48 h after the RNAi performance, each shrimp was challenged with 10^5^ copies of WSSV particles by intraperitoneal injection, and 48 hours later again, muscle, hepatopancreas and/ or gill tissue from 12 shrimps was sampled to examine the virus copies by absolute qPCR. To further investigate whether the lethality rates of Toll4 silenced shrimps was associated with viral levels, hepatopancreas, gill and muscle was sampled at 48 hpi to examine the virus copies by absolute qPCR. To evaluate potential antiviral role of the canonical Toll pathway components including MyD88, Tube, Pelle and Dorsal, shrimps with five groups were injected with each of the four components dsRNAs and the GFP dsRNA as control, respectively. Forty-eight hours later, each shrimp from the five groups was challenged with 10^5^ copies of WSSV particles and the gill tissues from 8 shrimps were sampled at 48 hours post infection to examine the virus copies by absolute qPCR. To explore the antiviral function of AMPs, a similar manipulation of RNAi plus WSSV challenge were performed with differences that gills were collected at more sampling times, orderly at the 24 h, 48 h, 72 h, 96 h and 120 h post infection.

To investigate the effects of Toll4 or Dorsal on the expression of AMPs *in vivo* after WSSV infection, AMPs expression in shrimps after receiving Toll4 dsRNA or Dorsal dsRNA plus WSSV challenge were analyzed. Hemocyte and/ or gill tissues from 9 shrimps were collected at 6 h post WSSV challenge, and the mRNA levels of fourteen AMPs were detected by qRT-PCR with specific primers (S1 Table).

### Shrimp mortality or survival assay

Healthy shrimps were injected with gene specific dsRNAs including Toll4, ALF1, ALF2, ALF3, ALF4, LYZ1, LYZ2, LYZ3, LYZ4 or GFP dsRNA (as a control) and PBS, and 48 h later were challenged with 10^5^ copies of WSSV particles in 50 μL PBS. Shrimps were kept in culture flasks for about 5–7 days following infection. The death of shrimp was recorded every 8 h and subjected to mortality or survival rate analysis.

### SDS-PAGE and western blotting

Hemocytes of normal shrimp and WSSV challenged shrimps were sampled with each sample collected and pooled from 5 shrimps. The nuclear and cytoplasmic fractions of hemocytes were extracted according to the protocol of NE-PER Nuclear and Cytoplasmic Extraction Reagents (Thermo, USA), while the total proteins were collected by RIPA lysis buffer. Samples were boiled for 5 min, separated on SDS-PAGE gels followed by transfer to polyvinylidene difluoride (PVDF) membranes. After blocking in 5% bovine serum albumin (BSA) in TBS with 0.1% Tween-20 (TBS-T) for 1 h, membranes were incubated with anti-Dorsal, anti-NF-κB p65 (phospho S276), anti-Cactus, anti-HSP90, anti-Histone H3 or anti-Actin for 16–18 h at 4°C. After washing in TBS-T, membranes were incubated for 1 h at RT with horseradish peroxidase (HRP)-labeled Goat secondary antibody to Guinea pig IgG (H+L), Goat anti-Rabbit IgG (H+L)-HRP or Goat anti-Mouse IgG (H+L)-HRP. Both primary and secondary antibodies were incubated in TBS-T with 0.5% BSA. Membranes were developed with the enhanced chemiluminescent (ECL) blotting substrate (Thermo Scientific) and chemiluminescence was detected using the 5200 Chemiluminescence Imaging System (Tanon). For relative densitometry of Dorsal, Dorsal-P or Cactus, the immunoblotted band volume was normalized to the corresponding internal protein volume in the lane, using the ImageJ software 1.6.0 (National Institutes of Health, Bethesda, MD). Statistical analysis of densitometry data from three independent experiments was performed by using the Student’s t test.

### Pull-down assay

The *L*. *vannamei* AMPs ALF1 (accession No. AVP74301), ALF3 (ABB22831.1), LYZ1 (ABD65298) and LYZ2 (AY170126.2) without N-terminal signal peptide were cloned into pET-32a (+) plasmid (Merck Millipore, Germany) specific primers (S1 Table), expressed in BL21 (DE3) *Escherichia coli* strain, and purified with Ni-NTA agarose (Qiagen, Germany) according to user's manual. WSSV structural genes including VP19 (accession No. NP_477936.1) [98], VP24 (NP_477524.1) [99], VP26 (NP_477833.1) [100], VP28 (NP_477943.1) [100], wsv134 (NP_477656.1) [101] and wsv321 (NP_477843.1) [57] were cloned into pGEX-4T-1 plasmid (GE Healthcare, USA) with specific primers (S1 Table), expressed in BL21 (DE3) *E*. *coli* strain, and purified with Pierce GST agarose (Thermo Scientific) recommended by user's operation. For His pull-down assay, purified His-tagged ALF1, ALF3, LYZ1 or LYZ2 was incubated with Ni-NTA beads, to which purified WSSV structural protein was added and incubated at 4°C for overnight with slight rotation. The mixture (beads and binding proteins) was washed three times with wash buffer (20 mM Imidazole, 50 mM Tris-HCl, pH 8.0), and then eluted in elution buffer (250 mM Imidazole, 50 mM Tris-HCl, pH 8.0). Elute was run in 10% SDS-PAGE, followed by coomassie staining and western blotting with anti-GST antibody to probe interacting proteins in the complex. For GST pull-down assay, purified GST-tagged WSSV structural protein and purified His-tagged ALF1 or LYZ1 were incubated with glutathione beads at 4°C for overnight with slight rotation. The mixture was washed three times with PBS and the bound proteins were eluted in elution buffer (10 mM reduced glutathionem, 50 mM Tris-HCl, pH 8.0) and analyzed by SDS-PAGE as described above, followed by coomassie staining and western blotting with anti-His antibody.

### AMPs antiviral activity assay

WSSV inoculum was incubated with His-tag fused proteins including ALF1 and LYZ1 or TRX (as a control) for 1 hour at room temperature before injection. Each shrimp was injected with mixture including 10^5^ copies of WSSV particles together with 10 μg His-tag fused proteins in 50 μL PBS. Shrimps were kept in culture flasks for about 5–7 days following infection. The death of shrimp was recorded every 8 h and subjected to survival rate analysis. The gill tissues from 6 shrimps were sampled at 96 hours post infection to examine the viral copies by absolute qPCR as described above.

### Immunocytochemical staning

Immunocytochemical staning was used to analysis shrimp Dorsal translocation in hemocyte recommended by a published method with a minor modification [51]. In short, hemocytes were collected by centrifugation (1000 *g*, 5 min) at room temperature (RT) and deposited onto a glass slide, and then fixed immediately with 4% paraformaldehyde at RT for 5 min. The hemocytes on the glass slides was washed with PBS three times, followed by incubated with prepared Guinea pig anti-Dorsal antibody serum (1:2000 diluted in 5% BSA) overnight at 4°C. The hemocytes were then washed with PBS and incubated with 5% BSA for 10 min; the Goat anti-Guinea pig IgG (H+L) Alexa Fluor 488 (Abcam, 1:5000 diluted in 5% BSA) was then added, and the samples were incubated for 1 h at RT in the dark. After being washed three times, the hemocytes were stained with Hochest (Sigma, 1 μg/ ml in PBS) for 10 min at RT and washed six times. Fluorescence was visualized on a confocal laser scanning microscope (Leica TCS-SP5, Germany). We calculated the colocalization percentage of Dorsal with nucleus stained with hochest, using the ImageJ according to the previously described methods [51, 102]. In brief, we opened the picture and chosen Image-Color-Split channels, while closed the no need channels, and then clicked Plugins-colocalization analysis-colocalization threshold-OK. The colocalization percentage of Dorsal with nucleus stained with hochest should be the quotient of the shared area of Dorsal and nucleus divided by area of nucleus.

### Inhibitor injection

The QNZ (EVP4593) (S4902, Selleck) was reported to be a high-affinity partial antagonist of NF-κB [103, 104]. Firstly, this NF-κB inhibitor with 0.5, 1.0 or 2 μg was injected into each shrimp (~8 g each) to explore the suppression effect on Dorsal. Then, the 2 μg NF-κB inhibitor for each shrimp was determined and used in the following treatments. DMSO injection was used as a control. The hemocytes of NF-κB inhibitor injected shrimp were sampled for protein and RNA extraction, as well as Dorsal translocation assay, at 6 h after WSSV challenge. The nuclear location of Dorsal was addressed by immunofluorescence staining as described earlier, the phosphorylation level of Dorsal was analyzed by western blotting with anti-NF-κB p65 (phospho S276) antibody, and the AMPs expressions were detected by qRT-PCR at 6 h after WSSV challenge. Besides, the gills were also collected for the AMPs expressions analysis by qRT-PCR.

### Genome walking

The 5' flanking regulatory regions of ALF1 and LYZ1 were cloned by Genome walking PCR amplification via GenomeWalker Universal Kit (Clontech) according to our previous paper [105]. Two pairs of primer AP1/ALF1-R1 and AP1/LYZ1-R1 were used to perform the first round of Genome walking PCR amplification, while AP2/ALF2-R2 and AP2/LYZ2-R2 were used for the second round PCR reaction. The PCR products were cloned to pMD-19T vector (Takara) and sequenced. Primers were listed in S1 Table.

### Dual luciferase reporter assay

The expression plasmid containing the ORF of *L*. *vannamei* Dorsal (FJ998202) was obtained from our previous study [106]. The 5' flanking regulatory regions of ALF1 or LYZ1 was gained by PCR amplification via the primer pairs of pGL3-ALF1-F/pGL3-ALF1-R or pGL3-LYZ1-F/pGL3-LYZ1-R, and subsequently cloned into pGL3-Basic (Promega) vector. Primers were listed in S1 Table. Dual luciferase reporter assay was performed as our previously described methods. In brief, *Drosophila* S2 Cells were plated into a 96-well plate (TPP) and transfections were performed on the next day. Plasmids were transfected using the Fugene HD Transfection Reagent (Promega) according to the user manual. S2 cells in each well of a 96-well plate were transfected with 50 ng reporter gene plasmids (pGL3-ALF1-κB or pGL3-LYZ1-κB), 5 ng pRL-TK renilla luciferase plasmid (as an internal control), 0, 50 or 100 ng expression plasmids (pAc5.1A-Dorsal). At 48 h post transfection, the activities of the firefly and renilla luciferases were measured according to user instruction. Each experiment was done at least three times, and for each assay, data were obtained from six repeated wells.

### Electrophoretic Mobility Shift Assay (EMSA)

After obtained the 5' flanking regulatory regions (promoter) of ALF1 and LYZ1, the potential NF-κB binding motif sequences were predicted and analyzed by JASPAR database (http://jaspardev.genereg.net/). We found that both ALF1 and LYZ1 contained a putative NF-κB binding motif in their promoter regions. The wild-type probes namely ALF1-κB-bio-probe (biotin labeled) or ALF1-κB-unbio-probe (un-biotin labeled) were designed as the sequence containing the NF-κB binding motif sequence (GGAAATGCAG). The mutant probe namely ALF1-κBm-bio-probe was designed as the sequence (GGCCCTGCCG) via replacing adenine with cytosine. Besides, the wild-type probes namely LYZ1-κB-bio-probe or LYZ1-κB-unbio-probe were designed as the sequences containing NF-κB binding motif sequence (GGAAAGGCCA). The mutant probe namely LYZ1-κBm-bio-probe was designed as the sequence (GGCCCGGCCC) via substituting cytosine for adenine. All the probes were synthesized by Life Technology and sequences were listed in S1 Table.

*Drosophila* S2 cells were transfected with pAc5.1A-Dorsal or pAc5.1A-GFP [106]. After 48 h, cells were collected and the nuclear proteins were extracted using NE-PER Nuclear and Cytoplasmic Extraction Reagents (Thermo). EMSA was performed using a Light Shift Chemiluminescent EMSA kit (Thermo) according to our previous method [49]. In brief, the nuclear proteins (10 mg) were incubated with 20 fmol probes (wild-type ALF1-κB-bio-probe or LYZ1-κB-bio-probe) for the binding reactions between probes and proteins, separated by 5% native PAGE, transferred to positively charged nylon membranes (Roche), and cross-linked by UV light. Subsequently, the biotin-labeled DNA on the membrane was detected by chemiluminescence. In competition binding assays, the complexes of wild-type probes and proteins were challenged with the un-biotin labeled probes, at 10-fold, 50-fold or 100-fold molar excess over the labeled probes.

### Statistical analysis

All data were presented as means ± SD. Student t test was used to calculate the comparisons between groups of numerical data. For mortality or survival rates, data were subjected to statistical analysis using GraphPad Prism software to generate the Kaplan–Meier plot (log-rank χ^2^ test).

## Supporting information

S1 Fig Multiple sequence alignment of the TIR domains of *L. vannamei* Toll1-9. The sequence identities among each other were calculated, and the values of greater than or equal to 50% were shaded.
(TIF)

S2 Fig Phylogenetic tree of Tolls/TLRs. The tree was constructed with the neighbour-joining (NJ) method based on the alignment of 117 Toll/TLRs full-length protein sequences by utilizing MEGA 5.0 software. The bootstrap values of 1000 replicates (%) were indicated on the branch nodes. *L*. *vannamei* nine Tolls (LvToll1-9) were indicated in red triangles. More detail information of sequences about these Tolls/TLRs was supplied with the Supplement Data S3.
(TIF)

S3 Fig The regulation of the promoter activities of ALF1 or LYZ1 by Dorsal *in vitro*. (A) Schematic diagram of the ALF1 or LYZ1 promoter regions in the luciferase reporter gene constructs. -1 indicated 1 bp before the translation initiation site. (B) Relative luciferase activity in S2 cells. The bars indicated mean values ± S.D. of the luciferase activity (*n* = 6). Statistical significance was determined by student T-test (** *p* < 0.01). (C) Binding of Dorsal with the putative NF-κB binding sites in ALF1 or LYZ1 promoter. EMSA was performed using biotin-labeled (Bio-) or unlabeled (Unbio-) probes containing or not containing the NF-κB binding motif of ALF1 or LYZ1. The nuclear proteins used were extracted from S2 cells expressing Dorsal or GFP. The asterisk (*) indicated non-specific binding bands. All experiments were performed three times, and similar results were obtained.
(TIF)

S4 Fig ALF3 or LYZ2 interacted with WSSV structural proteins. (A) Purified His tagged ALF3 and LYZ2. (B) His tagged ALF3 interacted with GST-VP24 and -VP26 via His pull-down assay and visualized by coomassie blue staining. (C) His tagged LYZ2 interacted with GST-VP24, -VP26, -VP28 and -wsv134 via His pull-down assay and visualized by coomassie blue staining. All experiments were performed three times, and similar results were obtained.
(TIF)

S1 Data The cDNA sequences of nine *L*. *vannamei* Tolls (Toll1-9) including the 5’-untranslated region (UTR), 3’-UTR containing a poly (A) tail, and open reading frame (ORF) underlined.
(DOCX)

S2 Data The cDNA sequences of fourteen *L. vannamei* AMPs including ALF1-4, LYZ1-4, PEN2-4 and CRU1-3. The open reading frames (ORFs) of these AMPs were underlined.
(DOCX)

S3 Data The sequences of Tolls and Toll like receptors (TLRs) were used in the phylogenetic tree analysis, the TIR domains were underlined. (DOC)

S4 Data Partial promoter sequences and the putative NF-κB binding sites of ALF1 and LYZ1. The putative NF-κB binding sites in their promoter are shadowed, and the transcription start site (G) and the translation initiation site (ATG) are showed.
(DOCX)

S1 Table Summary of primers in this study for dsRNA synthesis, Semi-quantitative reverse transcription PCR (Semi-qRT-PCR), quantitative reverse transcription PCR (qRT-PCR), absolute quantitative PCR, protein expression, genome walking, dual luciferase reporter assay and the probes used in EMSA. (XLSX)
