# Supplementary figures and images for: RNAi screening identifies a new Toll from shrimp Litopenaeus vannamei that restricts WSSV infection through activating Dorsal to induce antimicrobial peptides
Source: PLoS Pathog. 2018 Sep 26;14(9):e1007109. doi: 10.1371/journal.ppat.1007109 (PMC6175524; doi:10.1371/journal.ppat.1007109)

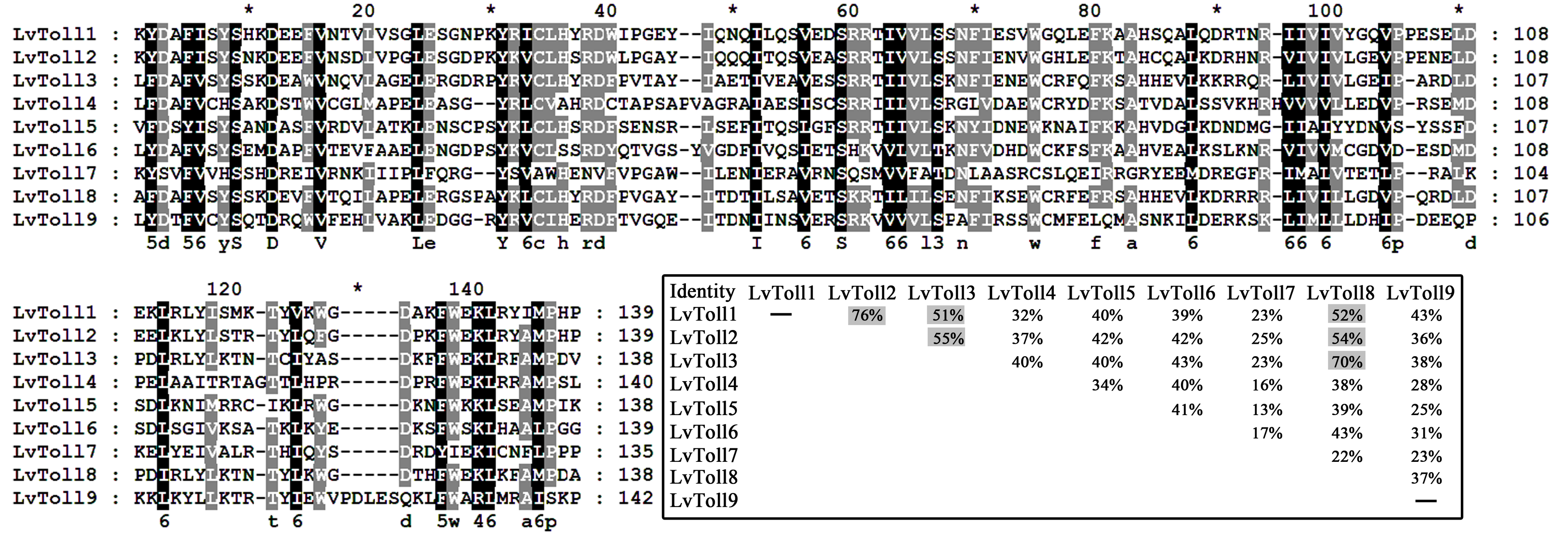

Supplement: S1 Fig — The sequence identities among each other were calculated, and the values of greater than or equal to 50% were shaded. (TIF) [file ppat.1007109.s001.tif]

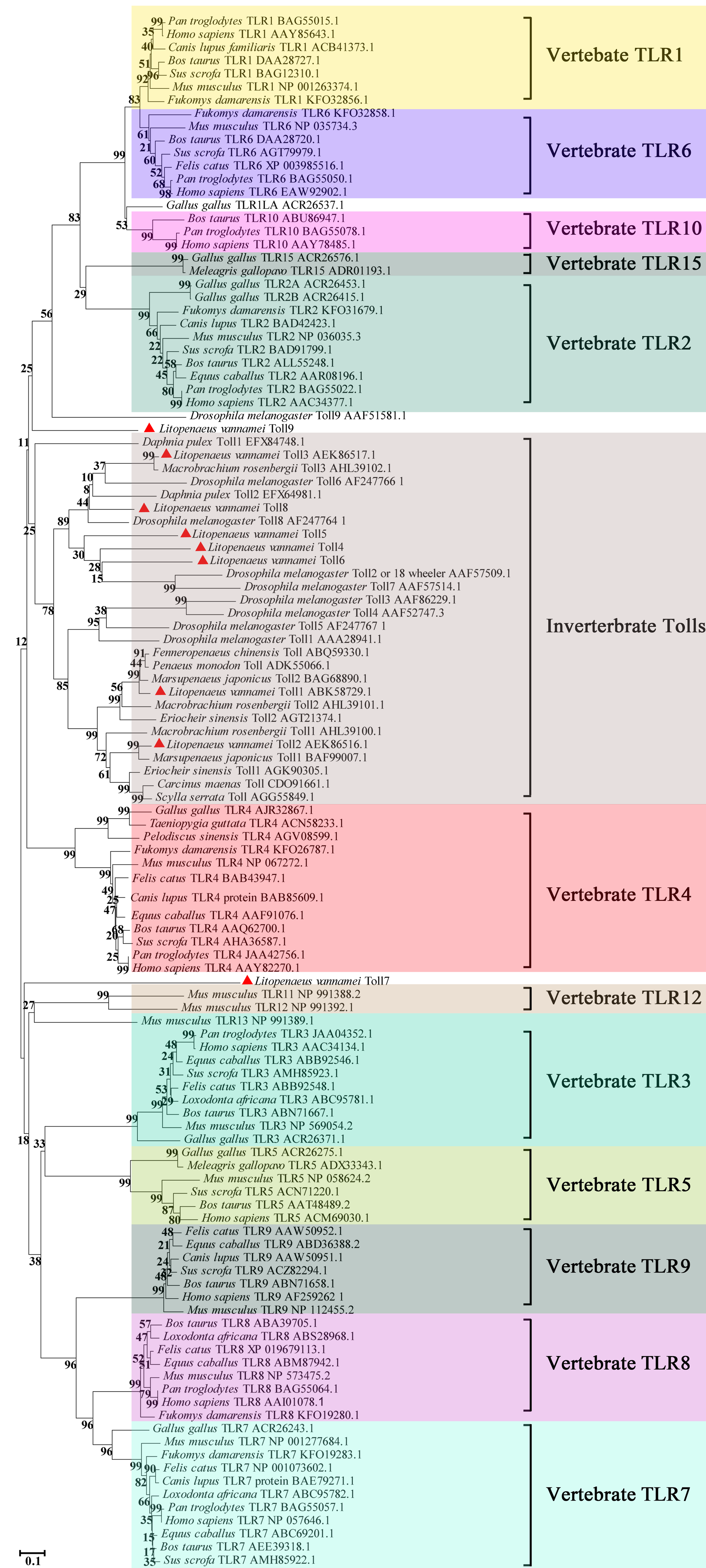

Supplement: S2 Fig — The tree was constructed with the neighbour-joining (NJ) method based on the alignment of 117 Toll/TLRs full-length protein sequences by utilizing MEGA 5.0 software. The bootstrap values of 1000 replicates (%) were indicated on the branch nodes. L. vannamei nine Tolls (LvToll1-9) were indicated in red triangles. More detail information of sequences about these Tolls/TLRs was supplied with the Supplement Data S3. (TIF) [file ppat.1007109.s002.tif]

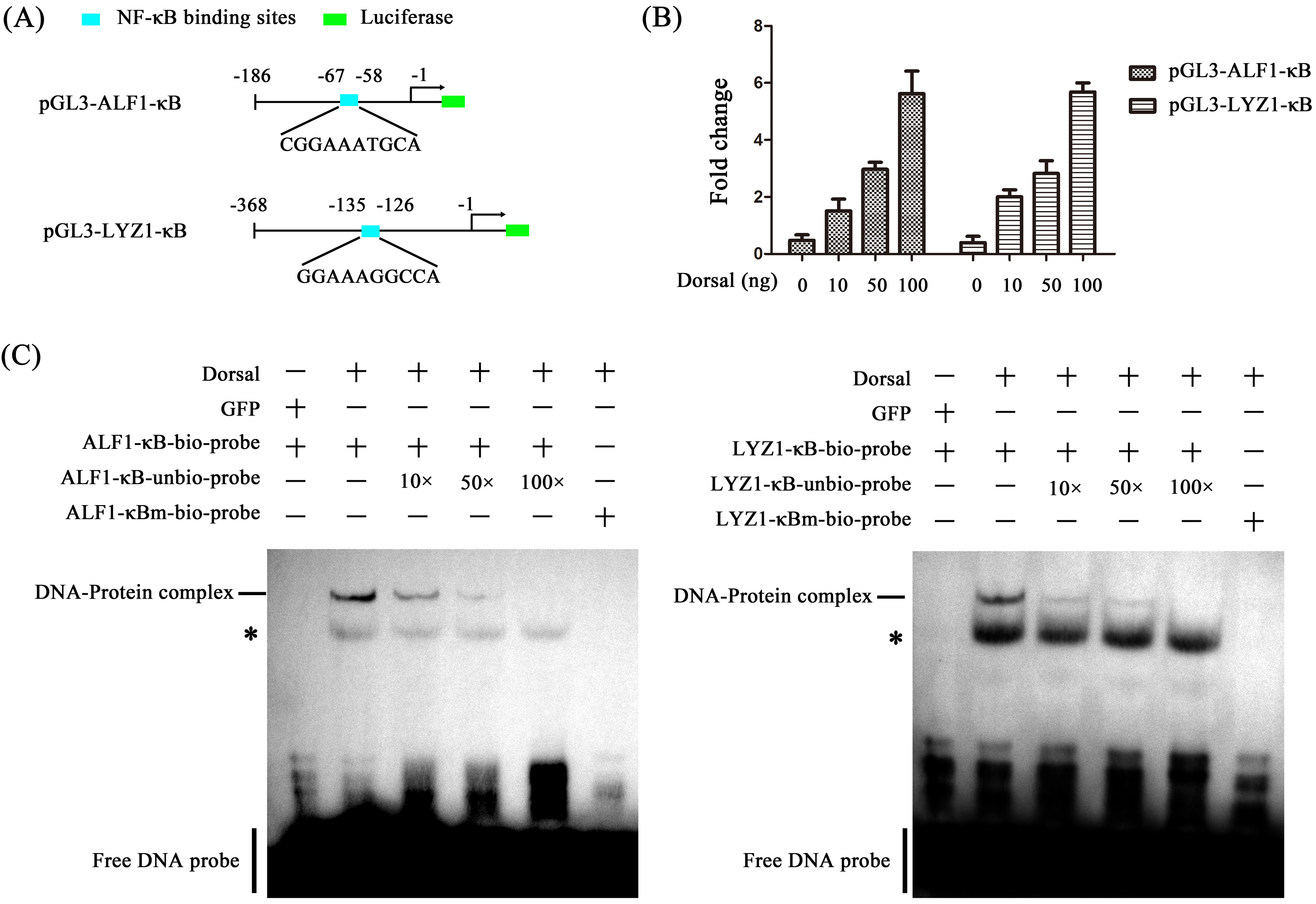

Supplement: S3 Fig — (A) Schematic diagram of the ALF1 or LYZ1 promoter regions in the luciferase reporter gene constructs. -1 indicated 1 bp before the translation initiation site. (B) Relative luciferase activity in S2 cells. The bars indicated mean values ± S.D. of the luciferase activity (n = 6). Statistical significance was determined by student T-test (** p < 0.01). (C) Binding of Dorsal with the putative NF-κB binding sites in ALF1 or LYZ1 promoter. EMSA was performed using biotin-labeled (Bio-) or unlabeled (Unbio-) probes containing or not containing the NF-κB binding motif of ALF1 or LYZ1. The nuclear proteins used were extracted from S2 cells expressing Dorsal or GFP. The asterisk (*) indicated non-specific binding bands. All experiments were performed three times, and similar results were obtained. (TIF) [file ppat.1007109.s003.tif]

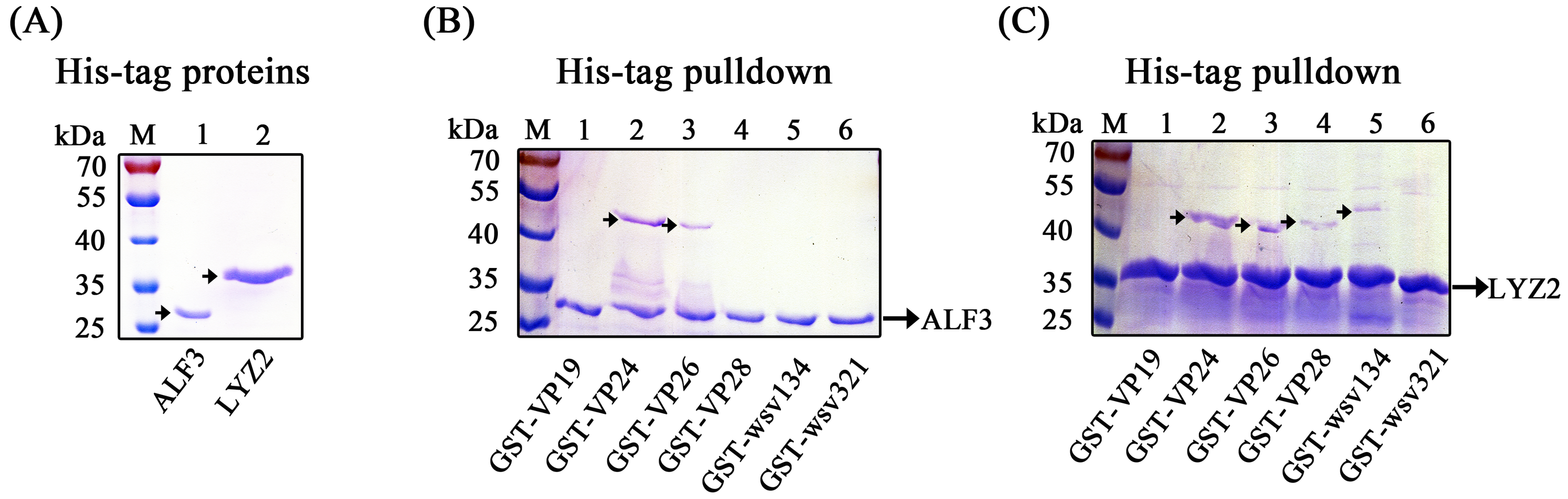

Supplement: S4 Fig — (A) Purified His tagged ALF3 and LYZ2. (B) His tagged ALF3 interacted with GST-VP24 and -VP26 via His pull-down assay and visualized by coomassie blue staining. (C) His tagged LYZ2 interacted with GST-VP24, -VP26, -VP28 and -wsv134 via His pull-down assay and visualized by coomassie blue staining. All experiments were performed three times, and similar results were obtained. (TIF) [file ppat.1007109.s004.tif]
