# Supplementary material for: RNAi screening identifies a new Toll from shrimp Litopenaeus vannamei that restricts WSSV infection through activating Dorsal to induce antimicrobial peptides
Source: PLoS Pathog. 2018 Sep 26;14(9):e1007109. doi: 10.1371/journal.ppat.1007109 (PMC6175524; doi:10.1371/journal.ppat.1007109)
Supplement: S1 Data — The cDNA sequences of nine L. vannamei Tolls (Toll1-9) including the 5’-untranslated region (UTR), 3’-UTR containing a poly (A) tail, and open reading frame (ORF) underlined. (DOCX) [file ppat.1007109.s005.docx]

**The cDNA sequences of nine *L. vannamei* Tolls (Toll1-9) including the 5’-untranslated region (UTR), 3’-UTR containing a poly (A) tail, and open reading frame (ORF) underlined.**

>Toll1

GAAATTTAGCCAGGGGTGGGTGTACATGTGCGTGCGGATACATTAATCTTCAACACTTCATCTTGCGTTGGGATATAGCGCTTCACTCTGTATTTTGGAGACAAGTTGATGAAAATCGTTTGTTTTTTGAGTATTAAAGAGTGTAATTCAAGTAAAAGGTCGTGGAATATTACCATGTACTCGGCCTTGTGGAGTGGCGAGTGCACCAGAGTCGCTGTCAAGTGTCTGTGTCAGACTGAACGTCAAACTCTCGACATCCAACACCCACATTGAAGGGTGTGTGAAGGAGCTGCGATGGTGCGTTCTGTGAGCCCCGAAGCGGAGCCCAGCTGATCCCGGCAAGCGTCCCCAAGATGAGCTCATGGTTGGTCCTGCCCGCCTTCCTGCTATGGGGGTGGGCGGCGGGCGGGGTCACACTTTCTCTGTCTTGTAGGCGTTGTGAAGGAGGCCCTGGCGGGTACACGTGCCCCAACTCAGAGAGTGCAGAGGCGTTTGTGCTCAAGACACTGCCAGATCAGGTTCTTCATGTGGAGTGTCGCAACAATATGGGGGACTTTTCACTGTTGAAGGACTGCAATTTCACCTCATTCAGAAAGTTTGAGTTTGAGAGATGCCCACTGCCTGGCGTGGCGTTTGGCGAGGTATTTCGGAGGATCGGAGTGCCAAGCAGTGATGTGAAATACCTCAGCTTCACGGCAGGCTCCTGGGATGCCTCCTCGGGTCTGGAGGAATGGCACCTGGACTCCCTCACAAACCTTCAAGCGCTACAGCTTGTTGACAACAACATCACTTCCTTCCCGCCTGCTCTGCTGACGAATACTCCCAAACTCGAGTTCTTTCGATTTATAGGAAATCGCGTGGGCAGTCTCCCGCACACCATGTTTGCAAGCACACCGAATCTCGTCATGGCAGACCTCGGGAACAACGAACTCACCAGTGTACCTGAAGACCTCTTCGCCAACCTCACGAAGCTGCTTAATGTCAGTCTCTGGAACAACCAGTTGACCGATATACAGAGAAGCTTATTTTCGGATATCCCAGGGCTCCGATTTTTAGACCTGAGAGACAACTTCTTGAGTGGCTTCACAAATAGGCAATTTCAAGGAATGAAAATATTAAGGAGACTAAACCTCGGAGGGAATAGAATCAGCAGTTTGACCGAGGATTCATTTAAGGATCTCAGGAGCTTGGAAGAACTCGAGCTTCATTCGAACTGGCTTGAAAGCTTACCCACAGGCATTTTTGACAACCAGAGGCTGATGAAGAAACTTATCCTGAGAAATAACAGTTTAATAAATTTGCCACAGAGAATATTCCAAAGATGCGAATCATTAAATATGCTTGATCTGAGCTTCAATCATTTGCAGTACATAGAAAGATTACAGCTTCCCAGTCCGAAAACTTCTCTAACATATCTCAATTTGGGAAGCAACAACATATCCTTTTCCAACACTGGGGCCCAGTTTATCCCGTACGACTTCCCTCTCTCTAACCAGTTGGAGCTGCAGCACATTTTCCTAGACAACAACAGGATCAACCATATTCCTACGCCATTAAACAATTTGTTTGTTGACCTGAAAACCGTCGACCTTTCTGGAAACTTGATTAGCTACTTGGAATTTCTCTCCATTCACTTCGTGTCGGACGGCGTCAAACTGAACTTGAAAAACAACCAAATTAAGGCAATTAATCTGCGTCGGTGGAAGCATTTTCCGTTTAATGAAATGATCAAGAATGTTACATTGTCACTGGAGGGAAATCCATTTACATGTAACTGTATACTTTACATATTTGCAACGATTGTTCAGGGAAAATTGAATGACCATAGTAAAACCTCATATCAGATATTAATTGATGATGCTGATAAAATAACATGCACCAGCTTAGAAGACCGGCAAATGCATGTTAAGACCCTCGATTTAAAATTCCTGACATGCAACCTGGACTTCTGCTCGGACAACTGTACTTGTTCATGGCGTCCATATGATGATATGTTGACTGTAGACTGTTCTTTCAAAGACATGAAGGAAATTCCCATGCCGACCAAGGACATGTACCAGCTCAAAACAAACTATTCTGTAACCCTAAATCTGATGAACAACAGCCTGGCAAACTTCGACGGCCTCGACCATCCCTTTTACACCAGACTGGCTAATCTGACCATTCCCTACAATAAAATCTCCCACATCAGTGAATCAGACCTTCCAGGCAACTTAAAAGTCCTGGATGTGCGAGGGAACAACCTGACCTTCTTATCAGACACCACTCTGGACTACCTCAATGTGACCGACGTGGTCCTCAGCCTTGGAGATAACCCCTGGACTTGCAATTGCGACATGATTGACTTCTTCACCTTTCTGCAAGTCCCTGAGAGGAAGGTATTAGACTCCAACAACATTAAGTGCGCCAGTGATGGAGAGCTGCTGTTGAGCATCAGTGAATATACCATCTGTCCATCATTCAGAAATCCCATGGTTATCGTGACAATCGTGCTCATCACAGTCTTCCTTCTCCTGTTTGCGGTTCTTGGTACGATGAGTTTCTACAAATACAAGCAAGGCATCAAAGTGTGGTTGTTTACCCATCGCATGTGTCTTTGGGCCATAACAGAAGACGAATTGGATGCTGATAAGAAATACGATGCCTTCATCAGCTATTCTCACAAGGATGAAGAATTTGTCAACACCGTCTTGGTGTCAGGACTGGAGTCAGGCAACCCCAAGTACCGCATTTGCCTTCACTACCGCGACTGGATCCCAGGAGAATACATCCAGAACCAGATCCTGCAAAGTGTAGAGGACAGCCGTCGAACTATTGTGGTGCTTTCGTCAAACTTCATCGAGAGTGTGTGGGGCCAGCTGGAGTTCAAGGCGGCTCACTCCCAGGCACTGCAGGACAGAACTAACAGGATAATAGTCATTGTGTATGGCCAGGTTCCTCCCGAGAGCGAGTTGGACGAGAAGTTACGACTGTACATCTCCATGAAGACCTACGTCAAGTGGGGAGACGCTAAGTTTTGGGAAAAGCTTCGGTACATCATGCCACACCCACAGGAACTTATACAGAAGAAACAGCAAAAGAACAGAAATGCAGACAAGCTTGAACTTGTTAAATCAAACTCGAAAAGTGTATAACGCAAGTTGAACCCAAAACTTTTTTTTCCGTGTACATGAGTAGCTGGACTACTGTCTTCACAGTGATGACTCGTTCAAAGAAAGTGTTCCAGATATGAAAACATATATAGACAGATGAATATATATTTATTTAGAAAATTATATGGACTTATTCCCAACAGTTCCTCAGATAGTAGGAATGTGGATATAAATGTTGTATGCAGCTAAACTTCTTACAACATTGACTTTGTACCATTGGTTGTAATTTGCCAATTGGCTGTGACCTGTATTGTAAACCAGGTACATATGTATATAGCAGGTTTATGAAAAAAAAAAAAAAAAA

>Toll2

GGTGTTCCGAAGCAGCGTAGTGGAATCAACAAGTGTGTGTGCGCGTGTGTTCATGCTGTAACCTTGTGGGCGTACAGAAACTTGTAGCGTCGCGTCTGTTGTGCGGGATTCCCTGCTACTCGGAAGAGTTAATGGACATCCATTCATAGAGCGGTAGCTCCCACTGCCATGACACGTCGCCCCGCCTGACATCGAAGAGCAACTGAACGAGACATGAATATGAAGCCTCTTTGGATCCTTCCTTGCCTCGTGGTGGTGTTTTCCATGGTGGCGGAAGTCCACGGCTTCAGCCCGTGTGGCAAGTGTGTGCGCGGAGAAGGCAAGCTCACGTGCCCGAGCGACATGGTCCCAGAGCGCTTCTCTGTCCTCATTTCGGACTACAACGAGACGAGCGAGAGCGTGTTCGAGTTGCGGTGCCTGCACGGGGCCAAAACGGTGAACTTCTCTCTGATTAACGACTGCAGTTTCCCGAACGTAAAATACGTGCTTTTCAATCGGTGCCCAAGGCCCAACGTGACGTTCGCCGAGGTGTTCCGGCGCGTCGGGATCGAGCCGGAGAACGTCGTCACCTTCTCCTTCCTTTCCGTGGACACCGGCCGCCCCGGCGAGGATCTGGAGGAGTGGCACTTCCGAGGACTGGGCAACCTGACTAGCCTGAAACTTCGAGGCAACCATTTCCAGTCTCTGCCGCCGAATATCCTTCAATACACGCCCAAACTGAACTATTTTCAGCTGTCATTCAACAATATTTCCACCCTCTCAGAAACGCTTTTTCAGAATACGACCCAACTCAAAATTCTCCACCTTTACGAAAACCAGTTCACTCACTTGCCTGATGGACTGTTCAAAAACCTGAATAAACTTACCAACATCAGCCTCTGGAGTAACAATATTGAACGAATTAGTCACAAACTATTCCAAAACCTGCCATCCTTGTGGTCACTGGAACTAGCCTTTAACAAAATATCGACCTTGCATCCCGATGCATTTGCAAGTCTCCCGAATTTAGGCAAGCTCTTGTTGGTTAGCAACAGGATCGAGAACCTTCCAGAATCCTTGTTTAGGAACTGCACCAACTTAGAATATGCTCATATGAGTAATAACAGAATAACGTCCATGCCTGCAGGACTGTTTAGAGAAACGAAGAATATCTACAGCATAGAGCTCAACAACAACATGATATCCAGCCTCCCAGATGATCTATTTAAGGGACTAAACAACTTAGGAAAGTTAAAGATGAAAAGAAATGCACTGAAGACCTTACCCTCAGGCCTCCTGGCAGACCTACCTAAGTTGGAAGTTCTGGATCTACAGTCAAATATCATTGAAGAACTTCCATCGGGATTCTTGGACAATCAGCGTATAACGGACATACTAATCTTGAAGAACAACAGCCTTGCAGAATTACCTGAGGGAATCTTCAAGAACTGCGCGGGGCTCCAGGAACTTTACCTGAGCCACAACAAACTAAGTATCCTGCAAAGCTCCTGGTTTCCTGCACCTGCCACGGCTCTTAGGAAAGTGGACTTGGGGAGCAATAATATCTCATTCTCGTCGTTTGCAAGTGGACAAGAAACTTCAGTTGAAGAGAACTTTCCTCTCTTGAATCAAGTCAACTTAGAGGAACTGTCTCTTGAAGATAACAGAATTACAGCTGTTCCCCAAGCATTTAGTAGTAACTTCGTGAACCTCACAAAACTGAACTTGTCTCATAATGATATTGAGTTTGTGGATGCCAATGATCTTATCTTTAAATCCGATGAAGTGGCTCTTGACCTCAAATACAACAAAATCAGAACTATTAACTTACAACACATTCAAAATATTGCACCGTATAAGGCAATTGATCTTTCCATAGCAGGCAACCCACTCGTATGTGACTGCAATCTATACTGGTTTTTAAGAATACTGCAAGGAAAAGATTTAGACAGAGAAGTACCTCAACTTCAAGTAAAAAGCCCTGAAAGCCTGACATGCTCATACATCGGCGATGAGACCACAGACAAACAGCTTCTGCGAGTCAGTTCAGAAATGCTTACTTGTAGCCTGCAGGAGTGTCCCGAGAGGTGCAAGTGTTTTACAAGGACACATGACAGGATGTATATTGTCGACTGTGCATATCAGAAGCTTCATGAGATACCGAGAATCATCAGTCAGGAAAAGCAGAATTTACGCAACTATTCCCTCACCTTAAATCTAAGGAACAATAGTATTTCTAACCTAGATCGGGTAGAAGACCCAGAGTACCGCTACTTGGTGAACTTGACAATTCCAAATAACAGTCTCATCTCCTTGAATGAATCCGTTCTACCAGACAGCTTACAAGTCCTTGATATCCGTGGGAACAACTTTACGTATTTAGAACCATCAGTGATAGACTACTTTAATAAGACAGATATAACTTTAAGTCTTGGAGAGAATCCATGGATCTGTGACTGCAAACTAACCGATCTCCAAAGCTTCCTCCGAATTCAAGAACTGAAGGTTTTGGACTTTTACAACATCCGATGCACGAATTTCAACGAGACACTGATAGACGTGACGGAAGGTGATCTGTGCCCCATTATTTTGCCGCCGGAGGTCATCATCGCCAGCACAGTGATCAGCATGTTCTTGATTCTCTCTGGGGTCTTGGCTACTGTTACCTTTTGGAAGTACAAGGAAGAAATCAAAGTTTGGCTCTTCACTCACCGGTTGTGCCTATGGGCTGTGGCGCACGAAGAATTTGATAACAAGAAGTACGATGCTTTCATCAGTTACTCAAATAAGGATGAAGAATTTGTGAACTCTGACTTAGTGCCAGGACTTGAGTCTGGTGACCCAAAGTACAAAGTGTGCTTACACTCTCGTGACTGGTTGCCTGGAGCCTACATTCAGCAGCAGATTACCCAGAGTGTGGAAGCAAGTCGCAGGACCATCGTTGTGCTCTCATCAAATTTCATAGAGAATGTCTGGGGTCATCTTGAGTTTAAGACGGCTCACTGCCAAGCTTTGAAAGACAGGCACAACAGAGTTATTGTTATTGTACTTGGAGAGGTTCCTCCTGAGAATGAGCTGGATGAAGAGCTGAAACTGTACCTCTCAACCAGAACTTACCTCCAGTTCGGTGACCCCAAGTTTTGGGAGAAGTTGCGGTATGCAATGCCACACCCCCATGACCTCATTTATAAAAAACAAAGGAAACGGAAAGACACAGACAAATTAGAATTGGTGAAATCAGATTCAAAGCAGAGTAAGTGAAAATTAACTTAATGAAAAAGCACATTGGAGTTCATAAGCAGAGTGTGGAAGTGGCAAATTCCTTTTAGACTTGAAAATTAGTTTCTGCTTACAAACTTTATTTACTCAAAAGTACTAATTACTGTCATCATTATCAGTGATACCTCTAATTGTACGGATTGCAAAAAAAAAAAAAAAAAAAAAAAAAAAAAA

>Toll3

AGTATGAGCTGAGTCAGTGGGAGGGAAGGACCATGACGCTCCACTACCCACTTACCGTGCGGTAATGTTGGTCCGTACAAAGTTCGAGACTTTGCGATTTCCCTCCTGTCTGCGGGAATGTCTCTCATGGCATTTCTCCTTGCAGTGAAATAATTTGCCAATCGAAATGTGGAGTGTTGGCCGCAGAGATGGCAAAGTGCTGGTCACTGTGTGGCAACTGTTAGCGTTTATTGTGTGCTGTGACTCTTCGGCTGTGCACTATAACAGCGAGGAGTGTACGAACCAAGACATCAATGCCCGAGAGAGAGCTCTCACTTGCTCTCTCAAGACTCTTGACGACGATTTAAGAGTCGCGAATCTTACGAGTGTGGCTGTGGACTCTGTTGCGCGCTTGTCTCTCGTGTGCAACGATGTCTACTTTTTCCAGAGTGTCTTGTCACCCTACACCCTGTCGGGGTTTGTGCGAGTGCGGGAACTCAATGTGGAATTTTGCAAGATTAGTGAACTGAAGGATAATGCGTTTATTAATCTCAGAAATTTAAGAAATTTAACACTTCGGACTAGAAACTTGGACTGGCCCGTGATGAGCTTGACCGCCAAGCCAGAAGTGTTCCGTCCTCTGCACCAGCTGGAGCGGTTAGATCTCAGCACTAACAATATTTGGGAACTGCCAGCTGGTGCCTTCTGCCACCTCGCCAACCTCAAACTGCTCAATCTAAGTCACAACCACCTGCAGGACATCACGCAGCTGGGGTTCGGCGGGGGCTCCTCGGACAGAAGCGTTTCCTCCTGCAGGTCCGACGTCAGTTCGCTCGATCTCTCTCACAACGACGTGACGGTGTTAGTGTCGGGCTCGCTGCAGGGCCTCGAACAGCTGCAGCATTTGTATCTCCAAAACAACGAACTCGGTAAGGTGGACGACAATGCTTTCCAAGGCCTGCGGAGCCTCCACACGCTCGACATTTCAAATAACCGTCTGGTGGCGCTGCCGGAGGACGCGTTCGCGCACACGCCAGGCCTCATGTACTGTCGGGCGAGGAACAACTCGCTCTCCGTGCTGGCGCCGGGGCTCTTCGGGGGGCTCGACCACCTCGTCGAGCTCGACCTCTCCTACAACGAACTCAAATCCGAATGGTTAACGTCCTCCATCTTCCAGGGGCTCGTCCGCCTCATGCTGCTGGATCTCTCGCACAACAAAATCTCCCAGCTGAACCAGCAGGTCTTCAGCGACCTGTACACGGTGCAGTTCCTCAGGCTCTCCCACAACCAGCTCAAGACGATACCCGCGGCGGCGTTTGCAGCCTGCGTCAACCTGCACACGCTCGACCTCTCGTACAACCAGCTGACGAGCGTCCCCGACAAGGCTTTTCAGGGAGTCGGTGTGCTGAGCTTCTTGGCCCTCGACAACAACAACATCAGCGAAGTGGGTCCGAATTCGCTGAAGAACCTGAGCAGCCTGGCGGACCTGAACCTCAACGGGAACGAGCTGACGGCCATTCCAGAGGCTGTGGCCCACCTGAAGTATCTGAAGACCTTGGACCTCGGGGAAAACCAGATTTCGGACCTCGCCAACATGCCCGTCAAGGGTCTGGAATTCCTGTACGGTCTGAGACTCGTGAACAACAAGATTAGGGGAAATCTAACGAAAGACACTTTTAGTGATATTCCTTCCCTTAAGATACTAAATTTAGCAAAGAATTCCATAACGGCCATTGAAACAGGCACGTTTGATAAAAATTTGAATTTGCAGGCAGTTCGCATTGATGCAAATCAGCTTTCCAGTATAAATGGGCTTTTTGAAAAGTTGCCAAATTTGTTGTGGTTGAATGTGTCCGATAATAATATTGAAGTGTTTGATTACCATTTCGTACCGCAAAGTCTAGAGTGGCTAGATTTACATAAAAACAAAATCAGTGAGCTTGGAAATTTTCTAGAAAGACACGACTTAAATTTGCAAACTCTCGATGCTAGTTTTAATAAGTTGCAGTACATCAACAGCATCCAAATTCCCGATAGTGTTCAGTTGTTATTTTTGAACGATAATAAGATTTCAGTTGTCGAGCCGTTCACGTTCTTCAAGAAAGTGAATCTGACCCGAGTTGACCTGTTTGCCAATCAGCTGTCAAGGATGGACATGTCAGCGCTGAGGCTCTCTCCCGTGCCCGTCGGAAAGTCCCTCCCGGAATTCTACCTGGGAGGGAACCCCTTCATCTGTGACTGTAATATGGAGTGGCTGCAGCGGATCAATGCATTAGAACACCGAAGGCAGCACCCCACCATCATGGATCTGGAGAGCATTTACTGCCAGATGCCATTCGCTCGCACGGGRGCCTTTATTCCTCTCGTGGACGTGAACCCCTCGCAGTTCCTCTGTCAGTACGAGACGCACTGCTTCGCTCTCTGCCACTGCTGCGAGTTCGACGCCTGTGATTGCGAGATGACGTGCCCCGACGGGTGTGGGTGTTACCACGATCAGTCTTGGAGATCAAACATTGTCGATTGTTCTCAGCAGGACGTGCAACAAGTGCCTGACCGCATTCCCATGGATGCCACGCAGGCTTACCTGGATGGCAACGACCTGAGGAACCTTTCGTCTCACTCCTTCATCGGCCGTAAGCATTTACAAATATTGTATGTTAATGCTTCTAACGTCAGGTCCCTCGATAACGAAACTTTTAGCGGGCTGAGTCGGCTGACGGCGCTCCACCTCGAGGACAACCTCCTTGAGGCACTTCGGGGGAACGAGTTTCAAGGCCTCGAGGTTGTCAGGGAATTGTACCTTCACAACAACCGTCTGAGATACGTCCATCAACACACCTTCGCCATGCTGTTCCACCTCGAGGTCCTCACTCTCCACAACAACCACCTCATTAACTTCCCCGTGTGGAGACTGGTAGACAACCCATACCTCAACCACGTCTCCCTGAGCACCAACCAGTGGTCCTGCCAGTGCCAGTTTGTCGAGTCTTTCGGCATATGGCTGAACGGCAACGAGAGGAAGGTGTCGGACGCGAGGGAAATCAAATGTTACACCGACGTCGCCGAAGAGGAGCCTGGTTCCTACATCATGGAATTCAACGTGACGACCTGCATGAACACCTCTTCATCTTCGACTGTCGTCCGGCCCATAGTGTTGGACAACTTGTTGCATCCAGTTATTGCTACATGTGTCGCCTTCGTCGTCGTGGTTATCCTGCTGTTATGTTTCGTGTACCGTGGCACCATCCGCGTGTGGATTTACTCGCAGTGCGGCTACAGAATGTGCCACAAGAATGTCTCCTCCGACGACCGAGATAAATTATTCGACGCCTTTGTATCCTACAGCTCTAAAGACGAGGCCTGGGTCAACCAGGTGTTAGCCGGCGAGCTGGAGCGAGGAGACCGGCCGTATCGCGTGTGTTTGCACTATCGCGATTTCCCCGTCACCGCCTACATCGCCGAGACGATCGTGGAAGCCGTGGAGTCTTCTCGGCGCACCATAATCGTCCTGTCGAAGAACTTCATCGAGAACGAGTGGTGTAGATTCCAGTTCAAAAGCGCCCATCACGAGGTCCTCAAGAAGCGGCGACAGAGACTCATCGTCATCGTCCTGGGCGAGATCCCCGCGCGGGACCTCGACCCCGACCTCCGTCTCTACCTCAAAACAAACACGTGCATCTATGCCAGTGACAAGTTCTTCTGGGAGAAGCTGAGATTCGCCATGCCAGACGTTCAGAACAGCCAGCGAGTGGTCCACACCTACAGTTCCATCCCCGAGAGATCTTCCTCTTCGGCCAACAAGTACAGTGTCAACAGTCCAGCGTCAATGCACCATAATTTACACGGTGGCACTGATGCGTACTGGGGCCTAACGCGTCTCCTAGTGGTCTTGCCGAATTGTAGTGGTCGTGTTGAGCTCTGAAGATCCTGCGGAACTCCCTGGTAGTCCTGTGGTACTCCAATGGCGACCCTTAGTCAAAGAGAGCTTTGTCGAAGGGCTCAGTCCTAGGTAGAGCTCTGAGATTATGCAACACGGCTGTAGTTTTACAAGGGCTCTGTGATGAAAGCTCCTGGCTGTGGTTATAACGAAAAGGGCTTTAGTGAGAAGGATACATAGCTTTAAAGTGACACATGTGGTTATAATGGACACGAAACGGAAGTGTAAGAATCGAGCTTGTGATGCAATGTTTTCGAAGTGTAGTGGAAGCAGGACCGTTTGTGGACGATGGAGCCATGTACATGTTATAGCAATCTCCATCGTTGAATATGTAACATCTAGTCACATATATATTTATATATATTGTATATGAAAGTGAACAGTGACTTGGCTTTCACTCCGTCATGTATATGGAAAAAAAACAACGAGCCGACAACTTTTTATGGCAAATATCTACCGTTAAAAAAAGCTTTTTTGTAAATGTGCGTGAAGGAATATGAAAAACAAACAAAACAAAAATACCCGCAGTATCTATTTGGCCTCAGCTGTTATTGTCATAAAGACATGATAAATAAAACTTTTTTGATGCATAAATATGTTGTGCTATGGATGTGATCATTGGTGCATGCAGCCAGTTATTTATTCAGACTATGCTTTATGGATATCGTTCACATGTGTTTTATTGACATATGGGAAATGTATCGTCACTTCAAACAGAGATCATGTTTATGTTATTGTTTATCTCTGTAATGTGTAACCATTGTCACGTCGTTTGAAAAAGACTTTTGTTAAAATCTGTTTGAAAAAAAAAAAAAAAAAAAAAAAAAAAAAAAA

>Toll4

CGGCGGGCTGCGGGCGGCTGTGGGCGGTCCAGCATCAGCTTCAGGCAGTGTAATCAACTGCCGCCCGTGACAGTACTGACCGGCCGCTGCGTCTGCCGCCCACCATCGATCAGCGCCCAGCAGCACACGCGCTCACGCCCGCCCTGTTTGCCACGTGCTCCCCATACCTGCGCGCCCGCCCACCACGTGTAGGTGATACATGTGGTAACTTAGTGACGACGGCCAGGTGTCGGGCGCGGGCGGGGCAGGGGGGCGGCCCGGCGGTACAAGTCGAGGCTCATCATGAAGACCAGGCCACCCTGCTGACGAGGCGGCGCGAGCAAGCACCCGCACCAGCCATGGTCTCCGTCAGCGCCCACGTGCCCGGGCGGGCCTTCGTGCTCATGGCCTTCCTGGTACTCCTGGGCGGCGTCGGCGGGCGGCCTCCCGAGTGCGAGTGGAAGCTGGAGAATGAGGGCGTGACGGCAGGCGGTAGTGAGCAAGTGCGGACCACATGCCACGTGCGGACTCTCAGTGCTGCCCTGCTGACGGAGGGCAACGGCAACGGGTCCCTCTCCGCCCTCAGCCACGCCACACACGTCACCGTCCTGTCGCTGCACTGCTCGCGGCGTGTGGTGTTTGAATCTGAAATTACTGCAGGAATGTTTGCTGTGTTCCCCCGCCTGGAGGATCTGCAGATCTTGGGATGCAAAGTGACAGATCTCCCACCAAGATCCCTGGCGGGGCTGCCACACCTGAGACGCCTAACACTGCGGGCTCATCACCAGGACTGGCCAGGAGCTGCATTGGCCCTCCATGAGGACGCTCTGGCAGATCTGAACCGCTTGGAGAGCTTAGACCTGTCATACAATGCCCTGTGGTCTTTGCCCCCCTCCATGCTGTGTGGTCTACCCTCCCTAACTGCACTAAATCTTACCCACAACCGTCTGCATCACCTACCGGATCTCGGCCTCGGAGGTAGCTGTTCCCAGAGCACTGACTTGGACAGAGAGAGTGAACGATCTCTCCCTCTACACCACTTGGATTTGACATATAACCAGGTGGCAGAGGTGCCAAAGAAAGCCTTCCACAGTGCCCGTGACCTCCAGTCTCTCTCCCTCAAGCACAATCGTCTAGCACACCTTGCTGACTGGGCATTTGGAGGCCTACAGACACTCAGACTCCTTGATCTATCTCATAACCGTCTAGTGGCAGTTCCACGCACTGCACTATCGGATCTACATCAACTGCGGGAATTACGACTAGCCAACAACTCGCTCAGTGTTCTCTCACCAGCTGCTTTTGGCAGTCTGGGCCAGTTACTCACACTCGATCTTTCCCACAACCAGCTAAGTCTCGGAGCCAGCAATGCAGAGCCTTTCACAGGTTTGATACGTTTGGTTGTATTAGATGTTTCTCACAACCGTCTTGTTCACCTGGGGCCTGACACCTTCCATGACCTCTATTCCCTCCAGGTCCTACGATTATCACACAACCAGCTATCCCATTTAGCAGATGCAACATTTGCAAGCCTCGCTAACCTTCACACACTGGATCTGAGTCAAAACCAACTTGGCACACTATCAGGCAAAGCATTGCAAGGATTGTCAGTGCTGACTCATCTATCTATTGACTACAATCAGTTAGAAGTTGTTCATGAAGCTTCACTTGATAACTGTTCCTCTTTGCATCAACTCAGTATGGCGCACAACCAGTTACAAATGTTACCAGAAGCAGTGAAGCGAGCACCTCGTCTTCGAACTCTTGACTTCAGCCACAACCAGATTCCTTCCTTAGAAGAGGGTGTCTTACATGGACTTGTACATCTGCAGGAACTTCGACTATCAGACAATGCCTTGAGTAACATCTCTCGCACAGCCTTCACAGAGGTCTCGTCTCTCATTCTGTTAGATCTCTCCAACAACTCCATATCTGAAATTGAATATGGAGCATTTGATGCAACTCCAAAGCTCAAAGGACTTGACCTTCACCACAATCTGTTAGGTGATGCTAATGGCCTTGTTATCCATCTAGAGAATCTAGTTTGGCTAAATGTGTCTTTTAACACCATAACATGGTTTGATTATGCCCTTGTCCATAAGAACCTTGAATGGATAGATTTAAGCAACAATATGATTAGCAAACTGGAGAACTTCTATCAAGTACAGAAATCTATAGGTCTGCAGAAGCTCTATGCTTCATATAACAATATTTCTGAAATCAGTGCTACTATCATTCCAGATGGAATTAAAGAACTTCACCTTCATCATAATTCTATCTCGTATGTTGCTTCCAACACCTTTTTGGACAAGATGAGTGTTTCACTGATTGACCTGAGGTACAACAGCCTCACCTTGCTGGAAGAAGCTGCTCTGAGGCTGTCACCTCGTCAGTCTCCTGCGCCCCTTCTCCTCCTATCCCATAATCCTCTTGAATGTGACTGTGGTGCAGATTGGTTATTGAGAGCAGCTGGTGCAGGTCTCTCTGGACCCATGACCGGAGGATCCATACTGCCACACTTAGGGGATGTAGGATCAGTACAATGCCGACTTCCAGGACTCTGGCATGGGGCCATGGTACCACTTATAGTAGTACAACCACAGCAATTCCTGTGCACTTATCGTCGCCATTGCTTTACTCTCTGCCACTGTTGTGATTTTGATGCTTGTGACTGTGAACAAACTTGCCCTAGAAATTGCACTTGCTACCACGACCACACATGGACACATAATGTTGTGGATTGTGGAGGTGGGTGGGGATCAATGCCATCTGGTGTGCCTATGGATGTAACTGAAGCATTTATGGATGGAAACAAGATGGGAATTCTTACCTCACATGCATTAATAGGACGTAAGAATCTGCGTGTTCTCTATCTTAATCACTCTGACATTTCAGCTATTCAGAATCGAACTTTCAATGGCCTTAAAAACCTGCAAGTTTTAAGACTTGACCATAATAAGATTGAAGCATTACACGGCTTTGAGTTTATCGACTTGCATGGATTACGTGAGCTATATCTTAACAATAATCATCTAAGACATCTCAGTAATGTTTCATTTTCTTCATTACGTGCTATAGAATTACTGCGACTAGACAATAACTACATTGTAACCTTCCCTGTATGGAATTTAGCCTTGAACCCATTCCTCTTGGAGGTCAGCTTGTATCATAATCCCTGGAGCTGTGAATGCAGTTACTTGGCCAACTTGAGAGCCTGGCTTGAAGCAAACCGAATAAAAGCAACTAACGCAAGTCTTGTGAGATGTCGACACAACAGCACAGGTATGATGGGTCCACCAGTTCTCTCAGACACACCACTCAGATGTGATCATTATGTTGCAACAACACGGATCAATAGTCTTATCATTCATGACTATGTTATGTTATTACTCATTACAGCAGCTCTAGTGCTACTATTAGTTGGTGCAGCTGTGACAGTGGTGGCATATAGACGACGATTAAAGCTTTGGGCAGTAAGTCGCTATGGTAAACGTCTCTTTGAAAAATCTTCAGCATATGTAGAAGAGAGAGAAAAACTCTTTGATGCCTTTGTGTGTCACAGTGCCAAAGACTCGACGTGGGTATGTGGTCTAATGGCTCCAGAGCTAGAAGCCTCAGGCTACAGGCTTTGTGTTGCTCACCGTGACTGCACAGCACCTTCAGCACCTGTAGCAGGTCGTGCAATTGCCGAGAGTATCTCATGCAGCCGCCGGATTATCTTAGTGTTGTCTCGTGGATTAGTTGATGCAGAATGGTGCCGCTACGACTTCAAGAGTGCTACTGTTGATGCTCTAAGTAGTGTAAAACATCGACATGTAGTGGTGGTCTTGCTAGAAGATGTGCCAAGATCTGAAATGGATCCAGAACTAGCGGCCATTACTCGCACAGCTGGGACAACACTCCATCCTCGAGATCCACGATTCTGGGAGAAGCTTCGACGGGCGATGCCTTCATTACGGCCACGCTTACGACAAGGGCTTGCAGGTACTATTGGTGGAAAAGCATCCAGTCGGCCTTTAGTGTCAGCAGAGCATCAGAATGGCCCGTCTTGGCCCCTGCCAGAAACGAAGACCTTAGGACACACCTCAGCTAAGTCTCTCATCATTAATCCATATTGGGAGACAGCTGTGGGGTCCAATGTTAGTGAAGCTGCATGGTGCTCCAGGAATGGTCCTGACCTCGGCGCACCCCCATGGGTCAGCAGCCCATCTAAACAAGCTCCGTCCACCTCACCTGAGAATGAGGCGAGGCTGGTAGGTTCCCCTACAGGTTCTACAGCCGAAGGCCAAGACCACACTTACATGAGTGTTAGTGAGTGTGGGGAAGTCAGGGCTTCACTGCTCCCGAACAACACAAACGCATCCACCTTGGGTGCAAAGCCCTCCCCTTGTGATGTCCCCTCAGGGGCAGACAGAGGATCAACAGGAGAAAAGGCCGAGGGGAGTAGTCGGCCTTCCGCGCCTCCTTTATTTCGCCGAGACGCCCCAGACTACCTCACCCGCAGTTGGATCTTCCACCCACCTCCCAATGAGCAACCTCCGCCCCCGGGCCAGACATACTTCGTATAGGGTGACATGCTCGTACCCAAACCATCCTATCTGCACGCCTACGCACTCTTTCATTCGTCCCTCTCTACTGAGTGTTACCACCCATGCCCTCCCCGCCTGCCCGCCCATTAAGCTAGTGCTGCATCTCGTCCATACCCCTGCAGCAGTAGGGAGCCTAATGCCTGCCCATTCTTCAGCCACAGTGTGCAACCTTGCCTCTCCCACCACCAGGCTATTGCTCTTGCACACGACCTCCTCCCGCTGATCCCCAAGATCCCACCCAAGGTTACCCTTTCCTCACCACCACCACTTCCATCAACACCCCTGCTCTTACTACCCAACTACGCTCCTTCATGCCCTCTGATCCTGATCCATTGATCAACACCTCATGCGAGGGATAGGCCCGCACACATTCCGCCCACACCAACAGTCTACCACAGCCATGATCGTCAGTGAGTGCTTAGACATAATGATGGTAAAAGCACTTGGCACTGAATCAGCGGCCGTGAAAGTTGAACCTGTGATGCCAAACGCATAGAAAGAAAAAGAAAGAAAAAATATTTACATAAATGAGGAGGTAACCCGCTAGTGCTTTCCGAAATGTGGACGCTGTGGTCATCAGTGTTGTAAAAATACTACTGATAATATAGATCAAGTAAGGTATTCATCATATGAAATTACATTGTTACCGTAGATAAGGCAACATGAAAAGCAATTAGCTGCAAGATGTAAATTTTAAGCTTAGCAAGAAGCTGTCAGTGTGCGTTTATAAATAATGCGATGCAGTGTTGCAATTTAGCTCTGCGCAAAGGGAGTGCCCTGTTGCAAGACTATGTTTTCCTATGCTGACTCCATATCTTGGATACTTAGTGAGGCAGCATCTTGCCAAAGCTGGAGGGAAAAAAAAAAAAAAAAAAAAAAAAAAA

>Toll5

ACCCGAGCCTCCGCCTTGCGCCTCGCCGGAAGTGAGCCAAGACGCACATCTTGCCAAGAACCCGATCTATTATGTCGGTTTTAATGCCTGGCGTCCTGTTCTGTCGGACTTCTATAGCCCATCGGAGAGGACAGTGATCATAAGAAAAAACTCCCAGCCGATAACTCGTCGCGCGGAGTGCCTCGTCGACAGTGAGTGAGCGAACGGGCCGTCGAACGGGCCCGGACGCCATGTCTTTCCCCTGAGAAAAAGGAATACAATCGCGCGGAAGAAGGGTCAGAAACAGACTTTAGGGACCATGCCGGGCTGGGGAGGCTGGAGCGCGTTCCTCCACCAGACCCTGCTCCTGGTGAGCGTCGTGGCGCCGGCCACCACCCTCGCCCAGACGCCCAGTGCCACCCCCGTCAGCAGTGCCGAAGCCCTCAGTGCCTCATCCAACGACACCTCGAAGGGCTCGCCATGGTGCCAGTGGGTGGCCCAGGAGGCCCTGGAGTGCCACATTCGAACCCTGGAGGACAGCCTGGGCTCTCAAGTGGTCAAGGGGCACTGGCAGGACCGCAACGACACGAGTGGCATCCCTGCGGCGGGACAGTGGCCGAGGGACCAGGTGCCGGGCCTGCCAGGGGCATGGGCCGCCCGGAGTGACACTCGCCACCTGCGCGTGCTGGGCGTGGAGTGCAGTCAGGTCCTCTACTATCAGAGCCGCCTTACGGCACGCACGTTCCAGGGCCTGGAAAACGTAGAGGAACTCGCCATCAACAACTGCAAGCTGGACAGTCTCCCACCGGGGACACTGTCCAGCCTGGGACGCTTGCGGAGTCTCGCGGTGACGACCCACAACGGCGAATGGGCGGCACTCGCCATGGAAGTGGTCTCGGGCTCCCTCCCTCTCACGCTGGAGCGGGTGTCCCTCGCCCACAACAACATCTGGACGCTTCCCCCGAGAGCCTTCTGCGGGCCCAACTCCCTCCACCACTTGGACTTGTCTCACAACCGCCTCCAAGACGTCCACGAACTCGGATTCATGGACCAGGTTCAACTGGACATTCTGATGATGAACATGAGCAACACAAGCTTCAGCGAACATTCTGCTGTGTTTTCAAACTTGGACGAGGAATCTAGTGCTGAAAGGTGCGGACAGGCTCTTCGTGAGTTGATCCTAGACCACAATGACTTAGTGAGGCTTCCTGACGGGAGCTTCCGCGTCCTGTCAGGGCTCAGAGAACTTCATCTACGTGACAATGATATCAGACTTATATCCAGTGAAGCCTTCAGTGGACTTACAGCCTTACAAGTGCTATATTTATCTAATAATCATATTATTGCCCTACATAATGGAACATTCTCTGACAATATAGCCTTAGAGAGGCTATATTTGAATAATAATTCACTCAGTGCTCTAAATTCTAAAGTTTTCCAGGACATGAAGGAGCTGCAAGTATTAGAAATCTCTAACAATAAATTATATCTCGATAACAGTCATGATGACCTCTTCAAAGGCCTTCGAAGGCTTGTCATTCTTGATCTGTCATGGAACTCCCTCACCACAATCACAAAGCTTTTATTCCGGGACTTGACATCCCTTCAGAGGCTGGTCCTTTCCCACAATGCCATACAGAGCCTCGAGGACGACAGTTTCACTTCTTTGTCCAACCTTTATGCCCTTGATCTCTCCCACAATTTGCTCCTCACTTTGGGTGAGGCTAATTTGAGGGGGCTTGTCGGCCTCAGTCTAATTCACTTGGCTAACAATTCTCTCTTTGAAATTCATCCTCATGCCTTCCGTCATAGCTCAAACCTGAAGCAGGTCTTCCTTAGTCACAACCACCTCCAGGCTATACCCAAAGCCCTTGAAAATCTCTCCTTCATCAAGACTCTGGACATGTCCTACAACAACATCGTCAGTATCCAACCCTTCCATTTTGGAGGTCTTGGAAATCTTGAGATGCTAAATGTGAGCCACAACAAACTAGATTTTATATCTCAGGGATCATTCAAAGGACTTGCTTCTGTAAAAGATTTGGACTTAAGGGACAATGCCATCCACACAGTGAATGAGGGTTCTTTTGATGGAGTGCCAAACGTCTTGAATTTGGTACTTGCTAGGAATCAGTTGAGTAATATAGATCACATATTTGCGGGTCTGCAACACCTTGAATCCCTGGATCTTTCAGAGAATAATATAAGGATGTTTGACTATGCATTCATCCCTCAACAACTAATTAATCTAGACCTGAAAAAGAACAAAATTGGACAACTTGGTAACTTCTTTAAAGTTCATACCGTCCTGACATTAGAGAATATTGATGCAAGTCATAATAGCATTAAAAGCCTTACTGAACTTTCTCTTCCAAACACCATCATGCATGTTATTCTGCACCATAACAATATTACAAGGATTTTGCCAAACAGTTTCCGAGACAAGGTGAATCTTCAGACTCTGGACCTCAGCATGAACTCCCTCCAAAGAATAAATCCCAAGTCTGTCAGCATGAGAGTAGCTTCTGGAAAACATACAAGTGCACAGATATATCTGTCGGGTAATCCTCTTATCTGCGACTGCGAAATGGAATGGCTTTACAATTCATTCAGAAGCACTCTGACTACAACTCCGGAAGCCACCGAGGTCACTTTCCTTCAGCCTCGCATTGATGACCTCGCCCGAGTAACATGTACGCTCCTTCATTCTCGGGAAGACACCACAGTCATGACACGTGTCTTGGAAACCTCTGCAGCCAACTACCTCTGTCCATACACGACACATTGCTTTACTCTCTGCCAGTGTTGTGATTTTATTGCTTGTGACTGCCAGATGAAGTGCCCAGATGCTTGTTCCTGTTTTCATGATGACACTTGGTCTATTAACCTGGTGGATTGTTCAGGAGGACACTTGGATCGGCTGCCGGACCGAGTGCCGATGGATGCTACAGTTGTTCTGCTAGATGGCAACAATCTTCAGATTCTCCATGCTCATCACTTTATTGGGCGACACAGCATTCAGCAGCTCTATCTGAATAATTCGCAAATCCAGACACTCCAAAATCGCACATTCCATGGATTAACTTCACTGCAAGTACTTCATTTACAAGATAACATGATTGTACAGTTGAATGGCTTCGAATTCTCTGGCCTTCATCATTTGAAAGAACTCTACTTGCAGAACAACCGTCTGTCATTCATCAATAATGCAACTTTTATTGGGTTAAAGAGCTTGGAAGTCCTCCGACTTGACAATAATTTCATTATAGACTTCCCAGTGTGGCTACTGAGTAACAATCGTTACCTGGCTAGTGTTACACTTGGAAACAATCCGTGGGACTGTGACTGCCAGTTTGTTGAATCTTTGAGGGAGTGGCAGAAGCAGCAGTCCCACCTTCTGATCAACCCTGAGGATGTGTTTTGTGTACATGGCGACTCTGGGGTGGTAGGGCCTAGCATAATCCTCCCAGAGTACTCTTGCACTGAGGCTCAACATGGCGTTACGCAGTATAAGTTTGGCCAGCAAGAACTGCCATTCCTGGCTGGGGGTTTGTGTGGTGGTGTTGCTCTCATCAGCGCTCTGGTAGTGATGGCAATGTTAGTTGCAAGGAGAAGGGCAGCAGCAGCTAACAAACTGGGAATTAACGGGTCTCCTGCATATTGCCAAGAGGAGGACGGAAAAGTTTTCGATTCGTACATTAGCTATAGTGCAAATGATGCCAGCTTTGTAAGAGATGTGTTAGCCACTAAGTTAGAAAACAGTTGCCCAAGTTACAAGCTTTGTCTTCATTCACGAGATTTCAGTGAAAATAGCCGCCTCTCTGAGTTTATCACTCAGTCATTAGGCTTCAGCAGGAGAACCATTATAGTACTTTCCAAGAACTACATAGACAACGAATGGAAGAATGCTATCTTCAAGAAAGCTCATGTTGATGGGCTAAAGGACAATGATATGGGGATCATTGCAATTTATTACGATAACGTTTCGTATTCCTCTTTTGACTCAGACTTAAAAAATATTATGAGGAGATGCATTAAACTTAGATGGGGTGACAAAAACTTCTGGAAAAAGTTATCAGAGGCAATGCCAATAAAACAAACATATGCTGGACTGCCAGTGTATGTATCAGAAAATTCCTATAAATCATCAACTTTGCCAACTCTCATCCCGCCCTCATCCTTGCCTCTTCCATCTTCTTCCTCCGTCCTCACCTCCACGACAGGGCTCACCACACCGTCAGAGATCGGGCACAGACCGTCATGCCAACAAGAGCCTCCTGCAACTACCTACAAGGCCCCTCCACCTCCTCGTCCTTGTTATACACCACCGTCATGTGATTACATTGTAATGACAGGACGAGACTGCCGAGACCCACAGTGTACCTGCCACCGACACTCTCACACAGCATACACCTACGTGGACGGCGACTCCTCGTCCCTCCACACCTACACCTCTCTTGAGCCCTTCACTTTACCCGACCCTCATGTACCAGAGTCATACACACGTGGACATTCGCCTGCCAGTAGCCATTACAGTGCTCTTGAACCCCCTGTCAGGAGGACTGTAAGAGCTAGCAAGCGGAAAAAGAAGAGACCTCTGAGTCAGAACTGTCCACCTGTGCATTGTGACACTCTGGAAAATCCTGCCTTCACCGAAGACGTGCATGGCGAACTTCCCTCTAACGGGACCTTCCGAAGAACCAAGAGTCTCCGGGCATCACGGGGAAACCAAGAACGTCATAGTGACTACTCGACAGATCACTCCAGTGACCGATCGAGTGGGAGATCGTACGACCACTCAGTGACCTCTGGCGGAGGCGTTGTTGACCGCAACGACGCCCTGTACATGGGTCTCGCCGATTCCCCGCCAGAACCGACGATGGTTACGACCGAAGAATGTTTCGTCTAGTCTTCACCAATTGAATAGTCTGTTACTCATAGATAGTAACCAATGAACTTCCTACTTGCAGTGTTTATTTCATTATCATGATGGTTTAGTCGAAAGCCATGTATGAATGTTGATTTTTTTTTTATAATTGAAAACAAGCGGACTCTCTGTAACGGGAGCCTCATATATGTAGCTCAGTGTGATGAGAGGAGCCTCCACTAATTCACTACAGAGTACTTACCGTCCCGGTGGCCCAGAGTCACCAGTGATTGCATAGGATGTAAATATTAATCTTAGGCTTTTATATATACATATATAAATATATATATCAAGTGGATATCTATTTCAGTATTACATGAAGAAGCAGGAACAAAATATTTCACGAATATTCTCACAGCTTTGTGAGCCTATTGAAAATGGGTAGTTAATACTTACAGTGTATAAATAGAAATCATGTCTTAATTTTTGTATTCATATGGTATATATGATTAATGCTTTTGACTAAAAGAACGCTCTAGACAAGGTTACCAAGCCAGACAAGGCCTAAGATTTTGTACAGGTATTCAGCTATAAAATGTGAACTGGATTGTGATTTGGTGCTGAGGAAGCGCCATGGCGAGTGCAAGGCTTAGAAAGACCTTCAAAAAAAAAAAAAAAAAAA

>Toll6

GTCGGAGGAGAGATGTGCCACTCGTGTCTCGTGGTCACTGTACAACTCCGCTGACATGATCGTAGTGTGTTGATAATACAAGTTAAGAAAAACTTTTTTGTGATTGAAGTGGTATTCGAACACTACCACCAAAATGAAGAATACTGTGGCATTATACGTGTTTGCAAGTGTTTTTGCGAGCTGTTCTGTAGCTTTCGTGTACAACGCTCCAGAAAATTGTGACTGGACCTTCCGCGACGAAGCGCAGAGAGAAGTGTCTTTATCGTGCAGCCTAAGGACAATCGGGAACGACTTCGACAGCAGCAATTTTTCTATCGCACAAAGTGAACATACGACTGAATTAGAAATCCTGTGCAGTGATGTGTTGTTCTTCCAATCGTCGCTTCAGCCTCGCGTGTTTCAGCGGCTCTACAACTTGGACACGATTTCGATCGAGTTCTGCAAGCTGACCAGCCTGCCCGCCGGGGCCTTCCTCGGCCTGGACGCTATGAAGGCGCTGGCGGTGCGGACGCACAACAGCGACTGGAGTGCCATGGCGTTAGAGCTGAACCCCGACAGCCTGGTGGGCATGCCTCATCTCGAGCGCCTCGACCTCGGCCAGAACAACATCTGGAATCTGCCTGAGCGCGTGTTCTGCCCGCTGCCTGCGCTGCGCCACCTCAACCTCACGTGGAACCGCCTGCAGGACGTGTCTGAGGTAGGCGTCTCCGGCAGCTGCGGCGCCCACTTGGTCACCCTCGACCTGTCAGGAAATGACCTGGTGGTTCTCCCCGAGGCGGGGCTCGCCGGCCTCGAGTCGCTGCGAGAGCTGTACCTGCAGTACAACGACGTGTCGATGCTGGCCGACGGGGCCTTCTCCGGGCTGTCCACCCTCAGCGTGCTGAACATATCGAGCAACCGCCTGGTGGCGCTGCCCCCGGAGGTGTTCAACGAGACCCTGGGCCTGACGGAGCTCCACCTGCAGAACAACAGCCTGAGCGTGCTGGCGCCGGGGCTCTTCTCCGCGCTCAGCCGCCTCACGGTGCTCGACATGTCGTTCAACCAGCTCACGTCGGAGTGGGTGACGGCGGAGACGTTCCGTGGCCTCCTCAGGCTCGTGGTGCTCAACCTCAGCCACAACCGCCTCACGCAGGTCACCCTCGACATGTTCCGCGACCTCTCCACGCTGCAGGTGCTCGACCTCCGCCACAACTCGCTCACCGTGCTGGGCGACATGACCTTCTCGCCCCTGGCCAACCTGCACAGGCTCGACCTCAGCTACAACAGCCTGGTGTCGGTCGAGTCTCGCGGCCTCTCGGGGCTGCACGTGCTGGCCTCGCTCTCGGTGGCGCACAACAACATCTCGCGCATCGCCCCCGAGGCCTTCCAGAACTGCACCAGCCTCCGCGACCTCCGCCTCGAGTACAACCTCCTTGAAGAGATTCCCGAGGCCGTGCGCGAGGCCAGCTCCCTGAGGACGCTTAGGATCAGCCACAACCAGCTGAGCGCCGTGGCCCAGGGCGACCTGACCAGCCTCTCGGCCCTCCGCCACCTCGACATGTCGAACAACTTCCTCCGCGGACTGTGCAAGTCGTGCCTGGCCGGCCTCGAGTACCTGGAGGTGCTCGACCTCTCGCAGAACGAGCTCAGCACAGTGCCACACGGCGCCTTCGACACCAACACTGGCCTGCAGCTGCTGCGCATGGACGGCAACAAGATGAGTGACATCAACGGTCTGTTTGCGTCTCTGTCCAACCTGTTATGGCTCAATGTGTCCGACAACAGGATCTCGTGGTTCGACTACGCGCTCATCCCCGATCAGCTGCAGTACCTTGACCTCCACAACAACAGAATCCGCGACCTTGGCAACTACTTCAGTCTGGAGAGCAAGCTGGAGCTGAGGACCCTCGACGTGAGCCACAACCAGCTGGAGAGCCTGAGCGCGTCTTCGGTGCCAGACAGCGTAGAGCTTCTCTTCGTGAACAGCAACAAAATCACTCGCATCGCCACTGGCACCTTCGCCGAGAAGCGAAACCTGTCCATGGTTGACCTGTACGACAATCTGCTCAGCAAGATCGACCTCAACTCCATCAACCTGCCCCGCGTGCCCGAGGAGCGCGACCTCCCCGAGTTCTACATCGGCGGGAACCCCATCTTCTGCGACTGCAACATGGAGTGGATGCACCGCGTCCACCAGATCAGCAGCCTCCGCCAGCACCCTCGCGTCATGGACCTCGACAAGGTGACGTGCACCCTCCCGTACCCCCGCTCGGCAGAGAACCGCGTGCCCTTCCTCGAGACTCAGCCCTCGCAGTTCCTGTGCCCCTACACGTCGCACTGCTTCGCCCTCTGCCACTGCTGCGACTTCATCGCCTGCGACTGCCAGATGACGTGCCCCATCGGCTGCTCCTGCTACCACGACGGCACGTGGGCCACCAACATCGTCGATTGCTCGGCCAGGAACCACCACCAGCTGCCGGACGACATCCCCATGGACGCCACGCTGGTGTACATGGACGGCAACGAGATGCCCTTCCTCGACGCCCACCACCTGATCGGCCGCAAGAACATGAGGGCCCTCTACCTCAACAGCTCTCGCGTGGAAAGGATTCAGAACCGAACTTTCCACGGTCTCTCGACGCTGAAGGAGCTCCATCTTCACGAAAATATGTTGGTCGAACTTGAAGGGTTCGAGTTCGAGCATCTGGAGCACCTTCGGGAGCTGTACCTTCAGAACAACCGTCTGAAGGTAATCAATAACGTCACCTTCGCAGGTCTGAAATCACTCGAAGTTCTTCGCCTGGATGGGAACTTCCTGTTTGAATTCCCTGTGTGGCACTTGAAGCTCAACAAGGGCCTCAAGGACGTTACCCTCGGCATCAACCTGTGGTCCTGCGAGTGCCAGTACATGGTCGACTTCAAGAACTGGCTCATCCGCGAGACGGACGTGGTGAAGGACGCCAAGAGCATCTTCTGCGTGTCCAACTCCACGGGCGAGCCAGGCCCTTACGTGCTGGAGAGTTCGTACTCCTGCGAGAACTTCGTGGCGACTTCCATCGTGCAGGAGAAGCTGGAGAACGACTTCCTGCAGCCGGTGCTGATCACGCTGGCCATCTTCTTCGTGGTGCTCGTGATGGGCGTCGTGTTCGCCGTGTTCCGGGTGCGCCTCCAGGCCAGCGTGTCCAAGAAGTGCGGCCTCAAGTGCTTCCCCTCCCAGCCCGCCCCCGCCAAGGAGGAGGAGAGGAACATGCTCTACGACGCCTTCGTCAGCTACAGCGAGATGGACGCGCCCTTCGTCACCGAAGTGTTCGCCGCCGAGCTGGAGAACGGTGACCCCTCCTACAAAGTGTGCCTTTCTTCCAGAGATTACCAGACTGTCGGTTCATATGTGGGTGATTTTATTGTGCAGTCGATTGAAACAAGTCACAAGGTGGTTCTAGTCCTTACGAAAAATTTCGTTGACCACGATTGGTGCAAATTTTCTTTCAAGGCCGCCCACGTCGAGGCGCTGAAAAGCCTGAAAAATCGTGTGATTGTGGTGATGTGTGGTGATGTAGATGAAAGTGACATGGATTCAGATTTATCAGGAATCGTCAAAAGTGCGACCAAGCTGAAGTACGAGGACAAGTCCTTCTGGAGCAAGCTCCACGCTGCCCTCCCCGGCGGCGCCAAGAAGATGTCCCAGCAGTGTTACATCACAGAGACCAACTACATCATGAGGAACAGCGTCCCCGTGCTGTCGCCGAACCACAACCTGAAGCAGAGCCAGTTCATGCCCAACATGGTGCTGACCAACACCCTCAAGACGCCCGTGTCCCACTACCACCAGCAGCATCACTATCAGCACACGCAGCCGTCCATAAACAGCGACACGGGCGACCTCGACAAGACCTTCGTCAGCGTGGAGACGGCCCAGTCCAGCCTCGCCCCGAGCCTCAACCACTCGTACATGTCGATCGACTACGCCGCCGCTCGCAACTCCCACATCTACGCCTCCATCGACGAGACGACGCCTGCGCTTCCTCCGTCCACGCTCCCTTCAGTGCACACGCTCCACCAGCACCTCCGTCAGCAGCAGCACGTGGAGCCCCTGCGTCAGTATCTACCCCAAGACGTGCTGTCGAGTCAGCAGCGCCGACCTCTCACGGGCGCGCCGATGCAGACCTTCGATCAACCAGCTGTCAGTGCGTCCTATTTCATATAAATATTGTATAAAAAAATATCTAAATATATATATTATCTATATTATAATTTTCTCTGATATGTGTGGCTGAGCTGTAATGATGAGAAAGTATGGAACGGGGAGCAATGACGAGATTTTGTGAGTGTTTTAGTAATAGACCACGTGAGTATGTCATTGTTTATGTCTAGTCAAGATGAGTGACAGCTGTACTAGTGCCTTCACATTACGGCCTTTGTTGTAGAACTGTTGCTGAGTCGTCTAGCTTATGTAGCGCTCCTTGCTTACTGTCACATTTGTATATATGTACTATATTTCCACTGTATAAATCGTGATATCTTTGGCCAAAGTGTGATAATATTTAGCTGTTACGTTGAGCTCAAATCGTGAGTCTATAACGGGTATTTTTATAAAATCAAATAATTACAAAAATAAAAGATGAATTGCTAAAAAAAAAAAAAAAAAAAAAAAAAAA

>Toll7

AATATATTTGAGCCGGGGCAGAACATCTTTTCCCGACTAGTGTACTGTAAGAGGCGTAGAACATCCTTCGCGTCTGGATCGTGTCTTTCCCGAATATTGCCAGATGGAATAGAGCAGACAGCAGACGACGACACAGAACAAAGCAGAAACAGCAGAGTCTCTCCTGAAAGCCGAGGATATATTAAAATAAGAGAATGCCCTTCCCGGCCTGGCAACTGGTCCTGATCAGTGCCGTTGTCTCACACGAAGTGCGCTTGGAGCCTCACTTGCAAGGGGCAATTGGCCCTTCGTATTCTCTGTCGGAATCCAGCGCGTCTCCGCCTCCTCGCCTGGGACAGAATGGAACTGTGCCACAATCTCATCCAGAATTAGCGAAGGAAGAGAGTGTTGCATCAGAAGCCCAGGAGCAAGATGTTCCTAATTGCGCCGTCGAAATCAACCATGCCACTTTATTCGACCGGGCATTTCTCTGCCCTCGGGCGGAATCTCTCTACAAGTTATTTGAAAATTTTAAAAGAGAGAGTTCTAACATGCGAGTCAAAATCACGGTGAAAAAGGTAGACGGGCCGTTGACGCTGCAGCCTCCTCCAAATATCATTATCGCGCTCTTTCTCCCCAACGCGAACGTAACACGCCTCTCTAATGGAACGGCAAATCAGGCTTTGCCCCACGCCCCGTTGGCATCCCTGGTCCAGCTGGACCTGAGGAACAACCCGCTCTTGGAGCTGGAGGGAATGCAGTGGCTAGGCGTCCTGCGCTCCCTGACGGTCCTCATTCTCCGAGAGACGCCGGTGACTGATCGAGAATTGCCGCCACTGTTGGCGCGTCTTCCTCTTCTCCAGCATCTTGACATTTCCTCTTCCGGTCTTCCTTACCTCCCGGAGGAAGCCTTCAACAAAAACCCGGAGCTGAAACACCTCGATTTATCGAATAATGAATTTAAGAAAATAACACTTCCTGAAAACCTAATCCGAGGGCTGACCCACCTCAACATCTCGAGCTGCCGGCTGGAGGAGGTGTCCGTGCCGGCGGCGGCATGGGAGGAGCGGTGGTCCACGCCCTCCTCCCAGGGGCGCCTGAGCACTCTGGACGTGTCCAAGAACCGCCTGAAGTGGCTGCCAAGCAGGCTGGTGGAGGCCCTCAATATATTTTCGTATGTTATTATCAAAGACAATCTGTGGAACGCCGCGTGCACCCGCTGTCCGCTCTACCACCTGTGGCAGTACTCGAGGCGGGCGTCGCGCGACGTGGTCGGCAAGGAGGAGCTCGACTGCTTTCGCCAGGACGTTCTGCTGAGCTGCGGCTGGGACAACTGCCCCACCGAGTGCTACTGCGACGATAGGAACAAGACGGTGAACTGCACGGGCAAAGGCCTGGTGGCACTGCCGACCATCGTGCCGTCCGAGACCGAGACCCTCGTGGTGGACGACAACGCCATCAGCAACCTGAACAACCTAGCCTCCCCCACGTACTGCAGCCTCAGGCACCTCAGCGTCAAGCGCAACCGCGTGACTGAGCTTTTGCTCTCAGAAGAAGGAAAATGCGAGTGCCATACACAAGAATACTACCCTAAGACACCGAAGTGTTTTCCTCAGCATCTCCACACAGTGTCCTTGGAGGATAACAGGATTGAGGGACTGACCGCGTCCGACTGCCCATTGCTCTATCCTCTCCACACACTTAGGATGCCTAGAAATAAGCTGGACAGCCTCGGAGCGCAGGTCTGTGGCTCCCTCGGCCGTCTCCGTGCGCTCGACCTCGCCCACAACAGTATCGCCAGCGTGACCTCGTCTGACCTCGCAACATACCCGTTTCTGCAGTCCCTCAACCTCTCACACAACGCCTTAGAAAAGCTCGTTCCTAATTTTCCGCCTGAATTCACAAGCCTGGACGTCAGTCCCAACTGCCTGTGGAGGCTAACCAGTGAGGACAAGAGTTTACCCAGAACACCATATCGGACAGAGAAAAGTTTGGAAGATCTGAAATTAACTGAAGGAGACAACTGTACGACCCCGGGTCGCCCTGGTCGAGGCGGCGAAGAGGAAGAGGAGGAGAAGAGGAGCTCCGTTTGTGATGGCATGAAGGAAAAGGTCTTCATGATTTTCATTAGCTGGGTCATCATAACGATCATTCTCCTCTACGTGTGCTACGTGTACGCCAGGCAGGGCAGATGCTGGTCGTCCCGTGAAGGAACCATCGCGCAGGAGAATGAAGACACGAGTGAGTGCAAGTACTCAGTGTTCGTCGTCCATTCCTCACATGACCGTGAGATAGTTCGAAATAAGATCATCATACCTCTTTTCCAACGCGGGTACAGTGTCGCCTGGCACGAGAATGTGTTCGTGCCCGGCGCTTGGATATTGGAGAACATCGAGCGAGCGGTCAGGAATTCGCAAAGCATGGTGGTCTTCGCTACTGACAATTTGGCAGCAAGCAGGTGTTCTCTCCAAGAGATTCGTCGCGGGCGCTACGAGGAAATGGACCGAGAGGGATTCAGGATTATGGCTTTGGTCACAGAAACGCTTCCCAGGGCGCTGAAAAAGGAACTCTACGAGATCGTCGCGCTCCGCACGCACATCCAGTACAGCGACAGAGACTACATCGAGAAGATCTGCAACTTTCTGCCCCCCCCGCGGCCGGTTCCGCTGCAGGACGCGTCCCTTCCGAACGCCTTCAGCATCCGGTCGCAACTGGATCGCTTTGATGAGATGCGGAGAGAAAACAGGAACCAGAGTATGTCTAATGTCGTGCACTTGCATCGGGACAATGAAGCTTTTGTCATCGACCAGTACACCGCGTCCATCACTAGTGGAACGTGGAAGGCCCCCTGGCCATCGATGTCGGAAGAGGAGATGCATCAGATGCTTTCAAGGTACTTGAAGAGCCCAGAGATGCCCGCGGAGTGCTACGCGGAAACAAGGAGTCTGAACTACGTGTTATTGGAGCAGGACAACGAGGATGAAAGTTCAGCCTGCATGGACGTTTTCTCCAGGCTCTCCGTCACGAGGACTACCTGTCGGTTTTGAGAGGGAGAGGGATAGGGATTAGGATTGGGAAGAAAAAAAAAAAAAAAAAAAAAAAAAAAAAAAAAAAAAAAA

>Toll8

CCTCTAGACACCCCGCACCGACGCACGCTCGGTCGCTCCCGCATTTTCGCTTGATTGGAGCTCTTACTCGACTATTTATGGATAAAGTACATTCTGTCACAATGTCGTGGATGACAGGGAAGCTGTACTACCTAGTGTGGAAGTGAAACGAAGTGATGGGACCAATCACCTTCAGGTGTTTTTTACCAAGGAGTAATGAGGACTCTGCATCGCGCATCCTAAGATGGCGAGACCGGTCGGCCCCGGACTAGCCAGGATAATGGCGCTCTCGTGAAACAGGCCGTGCGGCAAGATGTGGATACTGTGGGTCTGGTGCCTGGCAGCCCTTCTGTGGGCTGTCACGGGCAACGTAGGTCCGGACTACCACACTCCTGACGACTGCCAGGTCACCTCTCTCCCGGGCACCGACCTCGTCGCCCTCCTGTGTCGACTGCGGACTAACAACAGTGAGCTAGACGCCACGAATTTCAGCCTGGCGTCGACACGAAACACAGTGAAGCTTAGAATCGAGTGCAGTGACGTGATATTCTTCCAAAGTGCTCTCCAAAATAAAAGTTTCGTGCGATTACGCGAGTTGCAGGAACTCGATATCGAATATTGTAAGATCGGCGAGGTCCCGCGGGAGGCGTTCCTCGGCCTGACCAACCTGAGGAATCTGACACTTCGAACGTACAATGCCGACTGGTCTGTCGTGACCCTTAAGATCGCCAATGACGCCTTTATGGACCAGAAAAAGCTGGAGTACTTGGATCTGGGAAATAACAACATTTGGACTCTCCCGCCAGCGCTGCTGTGTGAGCTGGAAAATCTAAGGCTTCTCAACCTGTCAAGGAATAAGCTTCAGGACGTCACAGTTCTCAGTTTCAGTCAGGCAGAGCATATCTGCGCCCCGGGACTGCGCTCGTTAGATATTTCGTTTAACCACCTCGTAAGTGTTCCCGCATTTGCATTTGCAGCGCTCAAAAGCTTGCAGGTTTTAAATGTGAGTCTCAACGGAATTAGCAAACTGGAAGATAAAGCACTGTTCGGTATGTATTCATTAGAAGTGTTAGATTTATCGGGAAATCTACTGACCACGTTACCGCCGGAGTTATTTCAGGAAAATAAGAGACTGACAAAATTGTACATACGAAATAATTCTGTGAGTGTGCTCGCGCCTGGACTGTTCACAGGACTGAGTTTGTTGTTAGAATTGGAGTTGTCAGATAACCAGCTAACTAATACGTGGGTGAATTCCGAGACGTTCACGGATCTGCTCAGACTCGCTAGTTTAGACATGTCAAACAACAAAATCACGCGATTAGACGCGGCGACGTTTCGTGATCTGACGAACTTGCAGGTTTTGAAATTGCAACAAAATATGATCGAGACGATTTCTGACAACACTTTCAGTGGACTCTTCCGACTCCACACGCTCGTGTTATCGGACAACCGCGTCAAAATGATCAGTGACAAGACTTTCGGCGGCCTAATTGGGCTGCAGGTGTTAAAACTCGACGGAAACGAAGTGTTTAGCATCGACAGCGAAGCCCTTGTCAACTCCACGGGGCTTCAGGAGCTCCAGCTGAGCCACAACCACCTGCAGGATGTTCCCAAAGTGGTGAAGAGCCTCGCCTCCCTCAGGATCCTCGACCTGAGCGGCAACCATGTAAGTGTCATAAGCAACAAGTCCTTCCCGGACCTTCCTCACCTTAGTGTTCTAAGACTTGCTGCAAACGACATAGAAAACGTCACGAAAAGTGTGTTTACAAACCTCCCGTCTCTGCAAGTGCTTGATCTGTCAAGCAACAGGATTTTCACTGTAGAGAACGGAGCTTTTGATAACAACAGACATATTGAAGCTATCAGATTAGATGACAATATTTTGACAAATGTTAAAGGACTTTTTTCAAACCTGCCAAACCTTCAGTGGCTTAATTTGTCTAAAAATCATCTTGAAATGTTTGATTATGCATTTATTCCAAGAGGTTTAAAATATCTAGATCTGCGGTCAAATAACATTAATGAACTTGGTAATCATTATGAAATTGAAGGTGAGCCGCATTTAAAAATCATCGACGCTAGTTTTAACAGGTTATCCGACATCAGTGCAAGTTCATTTCCTGATAGTGTTGAAATAATTTTCCTGAACAATAATCTTATAGAAAGTGTGCAGCCTTTCGCCTTCTTCAGTAATAATAATCTTACAAAAGTGGATCTGTATGCAAATAAGATCAGAAATCTCGATCAGACGGCGCTCAGGCTTTCCCAAATGGATCCCTCTCGTGACCTGCCCGAGTTCTACCTGGGCGGGAACCCTTTCGAGTGCGACTGCACGATGGAGTGGCTGCAGACGATCAACAGCTATGACCAGCCACACCAGCGCCTGATGGTGATCGACCTCGACTCCATCGAGTGTCGCCTGATGAACAACAAGGGCAAAATCCCCCTGCTGGATGCCAAGAAGCTGCAGTTCCTGTGTGAGTACGACTCGCACTGTTTCACCCGCTGTAAATGCTGCGACTTCGACGCCTGTGACTGCGAGATGACGTGCCCCGCCAACTGCACCTGCTTCCACGACAACTCCTGGGCGGCCAACATCGTCGACTGCTCCCGCGCGGGTTACGGATCCGTGCCGGAGAGGATCCCGATGGACTCCTCCGAAGTGTACCTGGACGGCAACGCGCTCTCGTCCCTCTCCAGCCACACGTTCATCGGGCGAAAGAACATGAGAGTTTTGTATCTGAACGACAGCGGCGTCGAGGTCATACACAACAGGACATTCAACGGACTGAAGTTATTAGAAACACTCTACCTTCATAGGAATTCAATCAAGGAACTGAAGGGCTATGAGTTTGAACATCTTACCCTTTTACGTGAATTGTATTTACATGATAATGAATTGTTTTTCATTCAAAATACAACATTCCTGACCCTGGCCTCGCTTAGGGTGCTGAGGCTAGACAATAACCGCCTGAAAACATTTCCTGTGAACATGTTCTCTAAAAACCATAATCTCCATTCCCTTCACCTCAGCGAGAACCCCTGGACCTGCGATTGTGAGAACCTGAGGGACATTCAGGCATGGCTCCAGGGCGTTGGCAGGAAGCTCAGGGACGCGGATAAAATCTACTGCGCCCTCAACGGGTCGAGCGAAGTCGTGGCTCAGGTCTCGACCTACAATCACTCCTCGTGTAACAACGAGACCGAGACTACCACGATCCGGCACGAGGCTTACTTGGACTACGTGTTCCTGCCCACCATCACGCTCGGGGCCTTCGCCGTGCTCCTCACCATCACCCTCATAGTCTTCTGCAACCGCAACCGCATGCGGGTGTGGGTGTACGCCAAGTACGGCGTCAGACTCTTCTACCGGAGCGAGTACGAGGGCGACACCGACAAGGCTTTCGACGCGTTTGTGAGCTACAGCTCGAAGGACGAGGTGTTCGTGACGCAGATCCTCGCCCCCGAGTTGGAGCGCGGGAGCCCCGCGTACAAGCTGTGTCTGCATTACAGAGACTTCCCTGTCGGTGCCTATATAACGGACACCATCCTTAGCGCGGTGGAAACGAGCAAGCGCACGATACTAATTTTATCCGAAAATTTTATAAAATCAGAGTGGTGTCGCTTCGAATTCCGATCGGCCCACCACGAGGTCCTCAAAGACAGGCGTCGCCGCTTAATCGTCATACTATTAGGCGACGTCCCGCAGCGGGATTTGGACCCCGACATCCGACTCTACCTAAAAACAAACACGTACTTGAAGTGGGGCGACACGCATTTCTGGGAGAAGCTCAAGTTCGCCATGCCGGACGCACAGCCGCCCACCAGGAACCACCAGCTGCATGCCATCGCCGCCCAGCAGCAGCCGGGTCCTCGCCCTGTCCCTCTACACACCTGAAGTGGCCCTGACTGACGCGGAGCATCTTCAAAACACAACACGCAAACAGATGCAAGCCTGTTGCAGTGTGTGCCGCTCGTCTGCTGAGCCAGATTGCACGGTGCGGGTTGTTAGGCGAATGTCCCGAGTCGACGATCGGAAGGGAAAATTACCACATACCTATTTATCCTAAGGCATGATAGCAGCCTTGTATTCGCCTTTCCTTTCAACGAAAGTTCTTTGAAATTCGTTTGAAAGAGGGGAAAAATCCCTTCCGTTCTAGTTGACGACTGAAAATGGATCATTGCTTCCGCCATGCAGCTGCGACGGTCATGAGACGTCCAGGCAGCGACCGACGGCCGTGTTCCCGCGGGTCGCAGGTACACGCGAGAGAGGCCGGGCCGCGGCGTCTGTGTTTGTGTATGAGCGGCTTCTCTCTTGTCTGGCGCTGTGTGTGTCTGTGCGTGTGGCTGGCGCTCACATCCCGCCACTGTGTTCATCTTATTAGTGTTACGTGATGATTATCATTGTTATATCGTGCTTGTTGATCTTGAAATGATTATTTGTTGATTGTATATTTGTTGAATATGTTAAGAGATGAAAACAATACGTAGATCACGTGAAATTACGAATAATATTTCACCGGAGCAACACAAACATGATGAAGTGAGATTATCCGATGTGATAGTGACGAAAAATCCTCGCAATAACGACAAGAAAGTGGCATTAAAATGTGTGATTCGTGGCGAAGGTGCCCCGTGAGTTTTCGAGTGACAGTGAAGGCGGAGATGAAGACGAATAGTGAAAGGATAATGAAGAGGAGGCAGACGTCGGGAGCCGCGCCGAGCCTCGCCTTCCGAGCCAGGACGGAGCCACCCTTCCCACACGGAGCCACACCGGAGGCATTTGCTTTTGTAAATTACGTGGGCTTATATTTTCCCATTTTTACTTGTAAATACTACATATGCATTTCATTCTCTTTTTTAAACTGCCCTGGGTTTTGAGCTTAGGAATTTGTTTCTGTTATTGCTTTGTCTAGTCAATCAAAATGTTATTTTATTGAAGCGGTATGTGATAAATGAAACATATTATGTCTGATGCCAAATTGATTTAGAGATAGTTGAACTATCTGGAGAATACAGTAAGCCACACATGGTTTCCCATTGTGTTTAACACTTCAAAATTATTTTGTTGGAAAATATATATATCTATATCACATTTGTAATGATTTTTCTTTGGCATGTTTACTTATTATGTTAATTGTGTTATTACGTTAATTGGCTTCCGTTTTAAACCCCCCTAAAAGGTTAATGCTGAGATATGTTAATAATCTGTGATGATCGACTGTAGATATATTTATGAGTGTGTATGTATTTATCTCAAGGTCGGCCTTAAAGCAAGGTATGTTTGTTCAGTCAGACTGTAAGGCCTTCGGCTGTGTTTTTAGTGCTTTATTTGTTGATGTTTGATGCCTCACTCTGTATACTACCATTTCTCGATGTGTTAGGAAAGATTGTGTTTATATTGTTTATGTCATCGCCATGGTGTCACCTCTCCTCGGTTCTCTGCCCCGTGGTGCAAACGCTGTATTTCATAAAAAAAAAAAAAAAAAAAAAAAAAAAAAAAAAAAA

>Toll9

AGGACTTGCAATATTTCTGAGGATAAATTCAAATGCGTTTATGAATGACATTTTGTGATATAAACACAGTGCGATACCTTACTCTAGAGACATCAGTGCGAATATTAGGTTGTTAAGCATATTTTACATAATTTTTATCATATGCTAAAATAACTCATAAACTGCTCATTTAAATGGTATGTGATTCTCGTGCTTCGTTCCCCTAAATAGACAGCCACAGATAAGGTTCAAATTAACCCCATCACCCAACCCGAACCTGACAAGCTCGTCACAGATGACAGGTCCATCTGGTTTGTGATAACTAGTCGATGATAGTAGCTGATAAGTAACGAGTGCTGATAACTCGGAACGATAAACAGTAAACATTTCACTTTTTTTTGTTGTTTTTTTGGTTACTTAACTCCGCCATTGTTTCATAATGAAGCCAAAGCCATAAGGAAGCATGCTAGACGCGGAACCAGGGATGGGGTGGTTACATTTCACACATTTCGGAGATTGAGAGATATTCAAGTCTTGATAATTTGCACAAAAAGTAATACAAGGAAGCCAGAGATCCTTGCATGGCTGTGAGATAACCATCCAAGTGTTGCGGGTCGTCATGCTCGTATCAAGACTCACTGCCCTGGTGTTCGGCCTCTTGGTTGTCCTCGTCTCCCTCTCGCTCGGGGCGGCGATCAGGGGCGCGTCGGGGTTCCCTCACAGCAGCCTCAGGAGGGTCAAGTCCCCGGTGTCTCAGAACGATTTTTTAGGACCTTCCGATTCCAAAGGGTCTGACGAACGCCATGGGGAAGCAACGGTTGGAGAAGTGCCGCGGACGCTGGACTTATCCTTGAACGTGAAAGTGACGGAAAACGATAGATACGACAACGATGCTGTGTCATATGTCAGTCAGGCGGGGGGCAATGGGTACGCAGCGGAGAAAAGAGCTACGGAGGAATTGGTCAAAAATGGAGAAAGGCTGACCTCTAGCGCCATGACTAACGAGGTCCTGACAACAAAGACTAAGACTCAAGAGAGAAAAGAAGCCAGTAAGAACCAAGTCCCTTTCCTTTCCTCGACTAAGAGTTTTCAGAAATCAGAAGAATCCGCTTTGGTCTCAGAAGACCCCACGTTTCCTTCAACAGGAAAGGGAAGAATAAAGGAGGAAAAGGGTGATACTGCCAAAAACGAGACCCGTTCGTCACATGCGAAGAACAGGTGCGAGTTCAGATCGGCGCCACCTGACGTAGTCTTCCCAGATGCCTTCGAGGAGTTTTCTAGGTCGATGGAAGCAAACCTCAGCCCCGATGACCAACCTGACGAAGAGCTCCTCGACAGCCTCCTTCCTGACGGATGCCACTACACGGAGAGGCAGAAGAAGAAGGTCATGTGCACCGGAGCCAACATGACGTCCATCCCCGAGTTCGAGCACGCGAGGAACATCGAAACGCTGCACTTCAGCGGGACGTCCATTGTGCAGGTGACCAACCTCGACCCGCTCCCGAGGTCCCTCAAGGCGCTCTACTTCTCCAACGGCATGCTCAAGGTCTTCGACGGCAGGAACCTCAACAGGGTATCAGGACTCGAGGTCCTCCATCTCGACAACAACTTCATCACCAGCTGGAGCCTCGTCACCACCTTCTACTCCATGGGGGGCTTCGCTGAGCAGAACACCATCAAAACACTCAACATTCGAAGCAACCAAATAACTTATCCACCTCAGCCGGTCGGAGACAACGAGACAGTATTGCCTTACCTGGAGACTTTCGTGCTGAGCGAAAACCCCTTGTGTTACTTGCCAGACACGCTCTTCAAGCCTCTTAGGAACAGCAATGTCACCAGTCTTTATCTGAAGAACTGCAATATCGACGAATTTTACGGATCGCCCTTGTCCTACCTACCAAACTTGGAAGTCCTGGACCTCACCGGCAACAGGGCCATCAACGAGACCGAGCTGAGGGACCTTCTGCTACCACTTGGCCGCCTGAAACAGCTGTTCCTCGGTAACAACAACTACCAGACAGTTCCGACCAAAGCCCTCTCCTTGGTGAACGGCACCTTAGAGAACCTGGACCTCCATTCCTCGACGTTCACCTGCCTCGACAATTCGTCCTTCCCAATTATGCCAGTCTTGACTCACCTCGACCTCAAGTACTGCAGGATCAACGCGATCCGGGAACACACATTCCAGGGATTCCCGATGCTCAGGGAACTGAACTTGGACGGTAACAGCCTCACTACAGTTCCCCCTGAAGTGCTGCTTCCCTCGTTACAGATCCTGACGCTGAGCGACAACCCTCGCGCCAACGGGAACGACGGCGACCAACGCTTCAGCATGGAGGACGTCAGCTTCCAAGATATGGTGAACCTGAAGACGATACAGCTAAATCAGGTTATTATGGAAAAGATCGAGAGATCCTACTTCAACGACCTGTACAACCTCGAGGAGCTCTCCTTAACGGGGTGCGGCATCAAAACCATCGAAAACTTCAGCTTCGTAAACCTCACCAAGTTGCAGCATCTGAACCTCAGCGAGAACTACATCACCACCCTCTACAACGACAGCTTGGTCGGCCTCGTGAATCTGATCTCCCTGGACCTCTCCAACAACAAGCTGAAAGGGATCAACCGCATGGGGCGTTCTGGGGTCTCGTCCTCCGCGGTTCGAACCGATTCGCTTTCGTCCGGTACTGTTTCGGACGCTCGGGAAATCGACTCGTTTTTAAAGCGCGTCAGAGCCCCCTCGCCTCTGATTCCTTGGCTGTCCAGTATACGAAGGAACGCGAGGGCCGTGGGACCCCTAGGATGGCAGGGGAAGGATCTGCCGAGGACAACGATAGCCGCTTACGCCTTCAGCGATCTTGTTCGCCTGAGGACACTCAACCTCTCCGAAAACATGATCATGCTACTCCCGCCGGAATTATTCCACAACCTCACGAATCTTCTGATTCTCGACATCAGCTACAACAGGCTGATGACCTGGGACGACCCGGTGCTCGGTTCCATCCCCAACCTCACGGAGCTCCACCTGAGGACGAACTTGCTCGACGGCATCACCGACGCGATGGAGGTGGACTTCCGGAAGGAGAGCCTCAAGCTCGTCGACCTCCAGGACAACACCTTCAAGTGCGACTGCAGCCTGAGCAAGTTCAACCGGTCGCTCAACACGTCGAACTTCTTGAACTGGCCTTATCTGTGCAGGGAAGGGAAAGCAGACGTGGACATGGAGGAATACATCGCAAGGGCGCCGTGTAACTTCGCGACCCAGCCGGAGAACCACGGCCGGATGCGGGCCATCGTCATCAGCACGATTGTCTCGAGCTTGCTCCTCGTGGCCTCCGTCATGGTCTACAGGAAGCGATGGTATGTGCGCTACTTCATGTACACGGTGAGGATGAGGACGAAGGTGGTGCGCGAGGAAGCCGACAAGTACCTCTACGACACCTTCGTCTGCTACTCGCAAACGGACCGCCAGTGGGTGTTCGAGCACCTGGTGGCCAAGCTGGAGGACGGGGGAAGGTACCGAGTGTGTATCCACGAGCGAGACTTCACAGTAGGTCAGGAGATAACAGATAATATTATCAACAGCGTCGAAAGGTCGCGTAAGGTCGTGGTGGTTCTGTCGCCGGCCTTCATCAGGAGCAGCTGGTGCATGTTCGAACTCCAGATGGCCAGCAACAAGATTCTCGACGAGAGGAAATCCAAACTGATCATGCTGCTTCTGGATCACATCCCGGACGAGGAGCAGCCCAAGAAACTGAAGTACTTGCTCAAGACGAGAACCTACATCGAGTGGGTTCCGGACCTCGAGAGTCAAAAGCTCTTCTGGGCGAGGTTGATGAGAGCCATATCCAAGCCTTCGGATAGCGAGGCCATCGCAGCGTCGACGAAGTTATAGCTGAGGCTCCAAAGTCCAAGGCCTGACTCCTGTTTACATTAAGTGTTTGCTTGCACCGGCGACTGAACGCTGTTTTTGCTGGAACCCGAGCTGGTCGCCTCTGCGGAACGTTTGAACTAGACAGGTTTAGGCATCGTGAATGATATTCCTGTTCATCTTTGGTTGCCTTGCGAGTTTTGTAGTATAAACTGTGAAATCTTATATGATAATGAATCCATCTTGTTTGTCCTTGTTGCTAACCACAAAGGTTTGTCGAATGTGATACTGAGTAATTACAATAACACACATACACACACACACACCGACACACACAAAAAAAAAAAAAAAAAAAAAAAAAAAAAAAAAAA
