# Supplementary material for: RNAi screening identifies a new Toll from shrimp Litopenaeus vannamei that restricts WSSV infection through activating Dorsal to induce antimicrobial peptides
Source: PLoS Pathog. 2018 Sep 26;14(9):e1007109. doi: 10.1371/journal.ppat.1007109 (PMC6175524; doi:10.1371/journal.ppat.1007109)
Supplement: S2 Data — The open reading frames (ORFs) of these AMPs were underlined. (DOCX) [file ppat.1007109.s006.docx]

**The cDNA sequences of fourteen *L. vannamei* AMPs including ALF1-4, LYZ1-4, PEN2-4 and CRU1-3. The open reading frames (ORFs) of these AMPs were underlined.**

>ALF1 Genbank Accession No. AVP74301

GCCTTGACTTCGGGGGGAAAAGACGACGATGCGGGTGCTGGTCAGCTCTGTAGTGGCACTCGCCCTGATTGCTCTTGTGCCACGGAGCCAGGGTCAGGGAGTGCAGGACCTCATCCCTTCGCTAGTCCAGAGGATAGTCGGGTTGTGGCACTCGGATGAGGTGGAGTTCATGGGTCACAGCTGCAGGTACAGTCAGCGGCCCTCCTTCTATAGGTGGGAGCTTTACTTCAATGGCAGGATGTGGTGTCCTGGATGGGCTCCCTTCACTGGCAGATCTCGCACCCGCAGCCCTTCCGGCGCCGTCGAGCACGCGACGAGGGACTTCGTGCAGAAGGCGCTGCAGAGTAATCTCATCACGGAGGACGACGCTAGAATTTGGCTCGAGCACTAAGGCCTTTGTCTCAAGGTCATTCCCATTTCCTTTTGACGCGATGAAGGTCGAAGCGATATTTGTAAATCGTGAATAAGAAGAATGATGTCAGC

>ALF2 AVP74302

AGACTCGGCAATTCTAACTGCTGAACTGAACTCCTCGGGACACTGTGGCAGCCGGAACAGCGTCAAGCAGACAGTCAGCGTTAAGAGAGTGGTTTCCCGTCCTTCAGGAACTTCGATTCATAACACTTTCAAGATGCGAGTGTCTGTCCTCAGCATGGCCCTCGTGGTGGTGGTGGCTGCGTCCTTCGCGCCGCAGTGCCAAGCGAGTGGCTGGGAGGCGCTGGTGCCGGCCATTGCGAACAAACTCACTGGACTGTGGGAGAGCGGAGAGCTGGAGCTGTTAGGACACTACTGCAACTTTAGCGTGACACCGAAATTCAAGCGCTGGCAACTGTATTTCAGGGGTCGCATGTGGTGCCCAGGATGGACAGCCATCAGAGGCCAAGCCGAGACCCGTAGCAGATCGGGCGTGGTAGGCAGAACTACGCAAGACTTCGTCAGGAAAGCTTTCGGCGCGGGTCTCATCACCGAATCAGAGGCCCAAGTTTGGCTTAACAGTTAAGGCGAAGAAGAACGACACGCAGATATAATTTATAAGAGCGCTATGGAAGGGATCTCATTAATGGTAAACCCAAATTCTTCCGCGAATGTTGATACCTCATCCAATAAACATTAATATAACGAAAAAAAAAAAAAA

>ALF3 ABB22831

GCTTGAGAGTAACTTTCTTAATTTAGAGGATGCGTGTCTCCGTGTTGACAAGCCTGGTGGTGGTGGTGTTCCTGGTGGCACTCTTCGCCCCAGAGTGCCAGGCGCAAGGATGGCAGGCTGTGGCAGCGGCCGTCGCCAGCAAGATCGTTGGGCTGTGGAGGAACGAGGAGACGGAGCTGCTGGGACATAAGTGCCGCTTCACCGTCAAACCTTACATCAAGAGGTTACAGCTGAACTACAAGGGGAAGATGTGGTGCCCCGGCTGGACGACTATCAGAGGGGAAGCCAGGACACGCAGCCATTCCGGGGTGGCTGGAAGGACGGCCAGGGACTTCGTCGAGAAAGCCTTCAGGGACGGCCTCATCTCCGAACAAGATGCTAAGCGGTGGCTGAACTAACGGGCCCTCTCCTGCGTGAGGAGTTGTCGGGGTTCGAGCCTTCGTTGGCCGTGGAAGCTCTGCCATCTTGAGCTGTTGTATCTCTCTCTTCCACGTGGGGTTGACGTCTTGAGCTATTCTTGTTGTTCGATTTTTGGTTATAACAACTACACGAAAGTATATAACAAAAGCCGGTAATTGACGGTCTTAGAAGGATCCTCAGACTCTTTTGTTATTGATATTAAGGCAAAGTAGATTCCTTGAAAAAAAAAAAAAAAAAAAAAAAAAAAAA

>ALF4 AHG99284

CTTCGAGGCCAGAGCATCTACTGAATCTACTGAAACCTTCACTGAAAAGGCTCTTTCCCGCAGCATCTACCCACCTCTCACCAGCGGAAGAAAATAATGAAGCTCTCATTCCTGGTCGGCGTGGTGGCATTGGTGGCAGCGGTGGGATTGCTCGCTACCCCGTGCCAGGGTCAGGTGTGGGAGACCCTCATTCCTCTCATCACGCAGCAGGTCGTGGGGTTATGGAAGACTGGCGAGAGGGAGATGTTTGGCCACGAGTGCACGTACTTCGTTACACCCAAAGTAAAGAGTTTTGAGCTGTACTTTAAGGGAAGAATGACCTGTCCAACCCTGAGCAACGTGAGAGGAGAAGCTTTGACTCGCAGTCGCTCAGGTGTGGAGGCGAAGACAGTCGAAGACTACGTGAGGAAGGTCGTAGAAAAGGGCGTGATAACGGAGGAGGAGGCGAAGGCGTGGCTTAACAAGTAATT

>LYZ1 ABD65298

GCAGACACAGCCAAGCAACTTACACTTCGGAACCAGAAGACATAATGCGCGCATCACCATTCTTCCTGGCGGTTGCCCTCAGCGCCGCGGTTGCGGAATTAGAAGACAGTTGCCTAGCCTGCATGTGTTACGTATCCAGTGATGGTTGCGTCATGCCAGATGAAGTGTGTAGAACCACATCATGGTCAGAAGTGTGTGGACCCTGGGCCGTTACCAAACCTTACTGGGAAGACGCACATAAACCAGGAGGAGAATTTTACACTTGCATGGGTGACTGGGACTGCAACGAGCAGACCGTCCGGGCTTACCTGGACAGGTACGTAAGTAATCCTTACGCCAGCTGCGAGACCTACGCCCGCACGCACTACGGAGGGCCGTGGGGGATGAATGAGGACTACGCGACCGATTACTGGCTACAGGTCAAGGACTGCCTAGACTACGGGCTTTTCACACCACCTCCAAGTGTAGAGTGAAATACCTCGTGTTTATAGCCCAAGAAAGCTAAGCTGCCGAATGTGGTCGCAGCAAAGACTGGAAAAATAAATCTCCAAAACGATTAAAAAAAAAAAAAAAAAAAAAAAAAAA

>LYZ2 AAL23948

CGGGAAGTGCGAATTCGCGGAGGACTGCACGCTCGGCCTCCGCAAGAAACATTCACTTGCTATTTCTGAGTGATAATACTTGGATCATAGAAATGAGGGTGCTTCCTCTGGCGCTGCTGGGCGCGCTCCTGGCTGCTTCCGACGCCAAGGTCTTCGGGAAGTGCGAATTCGCGGAGTTGTTGAAAAGGGATTATTACCTCTCCAATGATGACATCAAGAACTGGGTGTGCATCGCGGAGTTCGAGTCGTCCTTCAACACGGCCGCCATAAACCGCAACAGAAACCGCAGCACGGACTACGGCATCTTCCAGATCAACAACAAGTACTGGTGCGGAAGCGACTACGGCAAGAACGTCTGCAAAATCCCATGTTCCGATCTGATGTCCGATGATATCACGGAGGCCCTGCGGTGCGCCGAGACTATCCGCCGCGACACCGAGCGCTTCAGGGGCCGCGGGAAAGGCTATTCTGCCTGGGTGGCTTACAACAGCAAGTGTAAGAATCGCGACCTCGATCAGTACATGGCAGAGTGCTGGTCTCACGGTTCCAACTCTGTCTTCCCGTTCTAGAGGACCACTTCGAGCTCTATGTTCTAGATTTGCAGGCTTGTCACCAGGGTTACCATTATTATAGCAAATAATACGAATGCAATGCTAAAGCCAAGATTTCAGCATTAGTTGGGGTTAATATTCGATGATTGCCTGTATATCATGATGTATGAATTTATACAAAATTGAAATGCAATTAACAGAATTACAAAACACAGCTGAAATGCAGACGTTGTAATGGTTGATAAATAAGTACATCATTAGCGGAAGTCTAGGCTCAACCATCGCATTCGCTTGGTTTGTGGCAATGATGGTATCTATGCTTGGCACGTCTAGATATTAGATACATGTCATCTCAGTTTTCAGTATGATTCAAGTACAGCACTTCGGCTCAGGAATTTCATGATTTCTGGTGAAACTGGATTACAAGTGTTTTGGAAACATCAAATTGTACTTTATGTTTGTTATGAATAAAATTTAAGGGCGTTCTTCATCTGGACATGTTTCACAAAAAAAAAA

>LYZ3 Under submit to NCBI

GTTGTCACTCGTCATACATTGGACACGTGGCCGCTGCGCCCTACGAAAGGTCCCGCTGCATGGCCGCTCGCCCATACATTCCGTATATAGCTGTCTTCCAAATCCGGGCGCTCACAAACAGCTGCGATGAATGGTCGAGTGCGGGTCTTCCTGGCGGCGGCGGTGGCGGTGTTGGCGGCGCTGACAACGGTGTCCGGCTCATCCGCCTCTCCGGGCGTCGGCGACGCCTGCCTCGCCTGCATGTGCTGGGCCTCAAGCAACTGCTCCATGCCAACGCCGCCGTGCAAGATGAACGGCTGGGGCGAGGTGTGCGGACCCTGGGCCATCACACAGCCGTACTGGATCGACGGAGGGCGTCTCTTTAACGACTTTTACAAATGCGTAGAAGATTGGAAATGTAACGAAGATACTGTACGCAATTACTTGAATTTTTACGTCACGGATCCTGACGCCAAGTGCCAGGACTACGCACGCACGCACGCCGGCGGTCCCCTTGGTGCCTGGAACGACAGCACCCTCCCGTACTGGTACTCGGTAAAGGAATGCCTTGACTACGGCATATTCACACCGGCATCGGTGTAAGGATGGTTATGGTATCTAGTGCGAAATAAATTGTATTTATGTATGTGTACGTGTATACATACATACACACACACATACATAC

>LYZ4 AVP74306

GACAGACCGCCGTGTGTCTGTTGGTGCGTCAGTGAGCTCGCGAACTCAGATCGCCTTGGTGTTGAATCCGCCATGAGGACATTGGCACTATTGCTGTTGGCGGCGGTGAGTGTCTCGGCGAAGATATTCGAGAAGTGTGAGTTGGCATCACTGCTCGAAACCAAGCATCAGATGCCGAGAGAAGATGTGAAGAAGTGGACGTGCATTGCCCAGTACGAGTCCACGTTTAACTCCGCGGCCATCAACACCGCCAACTGGGACGACAGCAAGGACTACGGCCTCTTTCAGCTGAACAACAAGTACTGGTGCGACGACGAGTTTGGCAAGAACGTGTGCGGAATTCCGTGTTCGGCCCTCCTGGACGACGACTTAACAGACGACCTTGCGTGTGCCAGAAAGGTCATTAAAGACACCGAACGATGGAAGGGCAAAGGAGAAGGCCTGGCCGCATGGGTCGCCTACGTTAACAGATGTCAGAACCGAAACTTGGACGAGTACATATCGGAATGCTGGACAGGTGATGCGACGGGTTCCAACATCATCAACATAAAGAACGAGTCCCCCATCGAATCAGCTGACACAGAAAACGAGGAGGTAGTCGGCGTCAGTGCTGGGGGTGACTCGCCTATCATCAATAATGTTAGAGTCCCCATTCAGTATCAAGTGTTGCCTATTTTTCAGCCTGTTCCACACGTTGCTTATTCATCTCCTATTGTGATGAGGAATCCCTATGGATATATTTACCAGCATGCTCTCCAGCAATAGGTCAGATCTGCTGACCGGAGAAGGCATTTGCGAATTTAGGTGGATGAACACATTGCATGAATTATATGCAGCGCGTAATTCATTCACGTTTGTTTATGTGTTTGTTGTTATTCATATTTGCGTTATGTATTTATTCATGCACTATCATGGTAGTTTGCAAACCTTTTGCTGTCAATATTCACAAATCAATAAAGATATAACAGCAAAAAAAAAAAAAAAAAAAAA

>PEN2 DQ206401

AAAAAACTAGTTCCTTATTTTTATTTTATCGATAATAGCAAAATATTGTTAATGTTAACTGCTGTAACTTTGTTTTAGTATAAATACACCCTATAAATTTGTTGAGGTAGTTGTATTTGGGCTCAAATAAGCGACTACTGGAAATGTTACGGTCCTGGCCCCGGGGTCGTTGCCTGTCGGCGGTCCTGCATATACATATACATACATACGACGCCTGAAGGTGCTTTCACAACCGCGTGGCTTCTCCATAAAAGGCATGGCACCACGGCCTCCGGTGCCACTCGGCGCTTGGCTCTCCCTCGAGCCTCACCTGCAGAGACCGACGCTCCGAGCCCGGGTTCCCTCCTGCGTCCGCCATGCGCCTCGTGGTCTGCCTGGTCTTCTTGGCCTCCTTCGCCCTGGTCTGCCAAGGCGAAGCGTACAGGGGCGGTTACACAGGCCCGATACCCAGGCCACCACCCATTGGAAGACCACCGTTCAGACCTGTTTGCAATGCATGCTACAGACTTTCCGTCTCAGATGCTCGCAATTGCTGCATCAAGTTCGGAAGCTGTTGTCACTTAGTAAAAGGATAAAGAAATTGACGGAGAAGACAATGGAAACCTGGCTTGACAACTTGTTAATTAATACTCATATGTGAAGAGATTGCAACCCTGATTTTGAGCTGTATTTTCTCGTTCAATTTTGTTTACTTTTGCTTGTGGAAAGGATGTGGGTATTTCGTCTATCCATCGCTAAAGATTCTTCCATGAATGTATGATGAAGGAAAGTGCATGTGTGTAAGTATGTATGTATGTGCTTACAGGTATTTGTTGCATTAAGTGTCCGTGTATTTAGGATCTGCAACACACGAGGAAGAGAATATTTGCCA

>PEN3 DQ206403

GACTACTGGAAATGTTTACGGTCCTGGCCCCGGGGTCGTTGCCTGTCGGCGGTCCTGCATATACATATACATATATACGACGCCTGAAGGTGCTTTCACAACCGCGTGGCTTCACCATAAAAGGCATGGCACCACGGCCTCCGGTGCCACTCGGCGCTTGGCTCTCCCTCGAGCCTCACCTGCAGAGACCGACGCTCCGAGCCCGGGTTCCCTCCTGCGTCCGCCATGCGCCTCGTGGTCTGCCTGGTCTTCTTGGCCTCCTTCGCCCTGGTCTGCCAAGGCCAAGTGTACAAGGGCGGTTACACGCGCCCGATACCCAGGCCACCACCCTTCGTGAGACCTTTGCCAGGAGGGCCTATTGGTCCATACAACGGTTGCCCTGTCTCATGCCGGGGAATTTCCTTCTCACAAGCGCGTTCTTGCTGCTCCCGGTTAGGGCGTTGCTGTCACGTGGGAAAGGGATATTCCGGTTGACGGAGAACACGATGAAAACCTCGCTTGACAACCTGTTGATTGATACTTGTATGTGAAGAGACTGTGATCCTGATTTTGCACCGTATTTTCTCGTTCAATATTCTTACTCTGGCTTGTGGAATGGATGTAGTTATTTGACCCTATGTTTTTTTTTTAAAGATTTTTCCATGAATGCGCGATGAATGAAAGCTTGCGTGATATGAATGAGTGCATCCACTTTTCAACGTCCCAGCAGGTGGCGCCGTATTCATGATTTGTGACACACGAGGAAGTGAATCCATGCCATCTGCCTTTCGTTGTAATTTTTAGTGAGTATGGATCTGTGTGTGGTTGATTTTTACAAATCTCTCAAAGGACTTTTAGAAATGTTACTCCTTTACAAATAAAATTGGTATCTTG

>PEN4 DQ206402

ACATGCAGATACAGATACATATATTCATATTTATATAATAAGTATGTATTTATCTACCCATGCCTTTATATTTACAATAATAGATGCCTATATGTATGCGAGTCAGAATAGAGGGCAACCATAACGGAAACACACAACGCTCATCGGGTCATTGCAATCTGTTTGAAAACTTCCCTAAAGACGATCGCTAACAGTGTGACTATTGTGTAGTTTTAGGTGATCTTCATTACGAGTGTTATTTATGTTTAGAAAATAAGGGGTTTTAGATATCAAAATTGATGATTAACAAGGATTCACTCGAAAAAATTATTTCATTTCTACCCTCTGTTCATTTGTTTATTTGTCTCATAGTCTGTTTAGCTATTTATTTCTCAGCCTGTCGTTCTGCTTAGCTACGTCACTTTATCTATCTGTCTCTCTTATCTCTCTTATCCCCCTTCTCTTTCTTTCTCCTACCCAGTTTCCCGTTTTCCCTTCCCTCCTATGCATCCTTTTTTCTCTCTCCCTTATGCCCTTTCGCTCTCTATATCTCTTCCATCCTGTCCTTTCTCTCTCCCAAGTTGCTTGCACAACCGCGCGGCGTCTCCATAAAAGGCATGGCACCGAAGCGTCCGGTGCCACTCGGCGCTTGGCTCTCCCTCGAGCCTCACCTGCAGAGACCGACGCTCCGAGCCCGGGTTGCCTCCTGCGTCCGCCATGCGCCTCGTGGTCTGCCTGGTCTTCTTGGCCTCCTTCGCCCTGGTCTGCCAAGGGCACAGCAGCGGTTACACGCGCCCGTTACCCAAACCATCCCGACCTATTTTTATTCGACCGATTGGGTGCGATGTATGCTACGGAATTCCCTCCTCAACAGCTCGACTTTGCTGCTTCAGATACGGGGATTGTTGTCACAGAGGATAGTCTGGTTGATGGAGAAGACGATGAAAACCGGGCTTGAAAACGTCTTAATTCATACTTGTGTGTGAAGAGACTGTGATCCTGATTTTGCACTGTGTTTTCTCGTTCCATGTTCTTGATTTTGCTTGTGAAATGGACGTAGGCATTCGGTCTATGCTTTGCAAGGATTAGCTAAAGATTGTTCCATGAATGTACGGTGAATGAAAGCGCGCTTGGTATGTATGTGCTGCATCTAGTTTTATCTGTCCCAACAGTTGCTCCCGTATTCATCAA

>CRU1 AF430071

ACCACTAGCTTGTACTGGAGGCAACCATGAAGGGCATCAAGGCGGTGATTCTGTGCGGCCTCTTTACGGCGGTTTTGGCTGGCAAGTTTCGCGGCTTCGGACAGCCATTTGGAGGTCTGGGTGGTCCAGGAGGCGGTGTAGGTGTTGGTGGTGGTTTCCCCGGAGGCGGTTTAGGTGTAGGTGGCGGTCTTGGTGTAGGTGGCGGTCTTGGTGTGGGTGGCGGTCTTGGTGTAGGTGGCGGTCTTGGAACTGGCACAAGCGACTGCAGGTATTGGTGCAAGACTCCGGAGGGTCAAGCCTACTGCTGCGAGTCGGCCCACGAACCAGAGACACCTGTTGGCACCAAGCCACTCGACTGCCCACAAGTCCGTCCCACATGCCCACGTTTCCATGGGCCCCCTACAACCTGTTCCAACGACTACAAGTGTGCTGGCCTCGATAAGTGTTGCTTCGACAGGTGTTTGGGAGAACACGTGTGCAAGCCTCCCTCATTCTTCGGATCGCAGGTTTTCGGATGAAGGATAAGCACGAAAGAATTTGAAAGGATGAAGAGAAAGAAGAAAAGACCATCTGAAGAACGACCGATGTTTTGGAATTTGACTGAAAAAAGAAAGAAAAACAGGGAATTCTTTCTTTCTGTAGGATTTATCTGATTACCATGATTTTTTTTATTTGTGAATTAGACTATTCTTCTGTCAAAAGAAACTTATAGGCC

>CRU2 JQ824114

GTTCATCGCACAGCAAGGAGAAGTATTACAAAGATGCTGAAGTTTGTAGTATTAGCCGTTGTCGCCGTGGCCGTGGCGCACGCGCAGGATAAAGACAAGGCCGGCACTCGCTTAGGAGGAGGATTCGGGGTTCCTGGAGCCGGTGGCGTCTTCCCAGGAGCCGGTGGCGTCCCTGGAGTAGGTGGCGTCTTTCCTGGAGCCGGTGGCGTCTTCCCTGGAGCCGGTGGTATCGGTCCTGGACCCGGCGGCCTCATCCCCGGAGGCGGATTCAACTGCAATTACTGCAGGACGCCCGTCGGGTACGTCTGCTGCAAGCCCGGTAGGTGCCCTCCGGTTCGAGACGTCTGCCCGTCGACCCGCTTCGGACCCCCGGTCTGCCGCCAGGACCTGGACTGCTCCGGCTCCGACAAGTGCTGCTATGACGTCTGCCTGGAAGACACAGTCTGCAAACCCATCGTGGCAGGTTCTCAGGGATAAGCCTGCATGTGAAACTTATCAAGC

>CRU3 AY465833

GGCACGAGGGAAGCACAGGATCTGATTCCACAATGGTCAGCGTCAAGGAGGTTCTGGTCGTGTTGGTCTTGGTGGCCGCTGTGGCCGTCTCCCCCGCCGATGCTGTTCCGACGAGACACAGTCGGCCCCGTCCCCAGCCCAGGCCCCGGCCGGGCACGTGCCCAGATACGAGCGACGTCATCGGCCCCTGCGTCATCACGGAGAGGAACTGCTTGTCGGACAGTCAGTGCGCACCCGGCCAGAAGTGCTGTCCGATCGGCTGCGGCAGAGAGTGCCTGGCTGTAGGTCCTCCTTACGGAAACGGAAGAAGGTAAAAGGAGTAAAAAAAAATGTCATGCTGATGTCATCGGTTCTCTGCGTCTTCCTGGAAAGGTTATTGAAGTGTGTTGATTTATTGCAAATAAAATTGATGTTTAAAAAAAAAAAAAAAAAA
