# Supplementary material for: RNAi screening identifies a new Toll from shrimp Litopenaeus vannamei that restricts WSSV infection through activating Dorsal to induce antimicrobial peptides
Source: PLoS Pathog. 2018 Sep 26;14(9):e1007109. doi: 10.1371/journal.ppat.1007109 (PMC6175524; doi:10.1371/journal.ppat.1007109)
Supplement: S3 Data — (DOC) [file ppat.1007109.s007.doc]

**The sequences of Tolls and Toll like receptors (TLRs) were used in the phylogenetic tree analysis.**

>*Litopenaeus vannamei* Toll1 ABK58729.1

MSSWLVLPAFLLWGWAAGGVTLSLSCRRCEGGPGGYTCPNSESAEAFVLKTLPDQVLHVECRNNMGDFSLLKDCNFTSFRKFEFERCPLPGVAFGEVFRRIGVPSSDVKYLSFTAGSWDASSGLEEWHLDSLTNLQALQLVDNNITSFPPALLTNTPKLEFFRFIGNRVGSLPHTMFASTPNLVMADLGNNELTSVPEDLFANLTKLLNVSLWNNQLTDIQRSLFSDIPGLRFLDLRDNFLSGFTNRQFQGMKILRRLNLGGNRISSLTEDSFKDLRSLEELELHSNWLESLPTGIFDNQRLMKKLILRNNSLINLPQRIFQRCESLNMLDLSFNHLQYIERLQLPSPKTSLTYLNLGSNNISFSNTGAQFIPYDFPLSNQLELQHIFLDNNRINHIPTPLNNLFVDLKTVDLSGNLISYLEFLSIHFVSDGVKLNLKNNQIKAINLRRWKHFPFNEMIKNVTLSLEGNPFTCNCILYIFATIVQGKLNDHSKTSYQILIDDADKITCTSLEDRQMHVKTLDLKFLTCNLDFCSDNCTCSWRPYDDMLTVDCSFKDMKEIPMPTKDMYQLKTNYSVTLNLMNNSLANFDGLDHPFYTRLANLTIPYNKISHISESDLPGNLKVLDVRGNNLTFLSDTTLDYLNVTDVVLSLGDNPWTCNCDMIDFFTFLQVPERKVLDSNNIKCASDGELLLSISEYTICPSFRNPMVIVTIVLITVFLLLFAVLGTMSFYKYKQGIKVWLFTHRMCLWAITEDELDADKKYDAFISYSHKDEEFVNTVLVSGLESGNPKYRICLHYRDWIPGEYIQNQILQSVEDSRRTIVVLSSNFIESVWGQLEFKAAHSQALQDRTNRIIVIVYGQVPPESELDEKLRLYISMKTYVKWGDAKFWEKLRYIMPHPQELIQKKQQKNRNADKLELVKSNSKSV

>*Litopenaeus vannamei* Toll2 AEK86516.1

MNMKPLWILPCLVVVFSMVAEVHGFSPCGKCVRGEGKLTCPSDMVPERFSVLISDYNETSESVFELRCLHGAKTVNFSLINDCSFPNVKYVLFNRCPRPNVTFAEVFRRVGIEPENVVTFSFLSVDTGRPGEDLEEWHFRGLGNLTSLKLRGNHFQSLPPNILQYTPKLNYFQLSFNNISTLSETLFQNTTQLKILHLYENQFTHLPDGLFKNLNKLTNISLWSNNIERISHKLFQNLPSLWSLELAFNKISTLHPDAFASLPNLGKLLLVSNRIENLPESLFRNCTNLEYAHMSNNRITSMPAGLFRETKNIYSIELNNNMISSLPDDLFKGLNNLGKLKMKRNALKTLPSGLLADLPKLEVLDLQSNIIEELPSGFLDNQRITDILILKNNSLAELPEGIFKNCAGLQELYLSHNKLSILQSSWFPAPATALRKVDLGSNNISFSSFASGQETSVEENFPLLNQVNLEELSLEDNRITAVPQAFSSNFVNLTKLNLSHNDIEFVDANDLIFKSDEVALDLKYNKIRTINLQHIQNIAPYKAIDLSIAGNPLVCDCNLYWFLRILQGKDLDREVPQLQVKSPESLTCSYIGDETTDKQLLRVSSEMLTCSLQECPERCKCFTRTHDRMYIVDCAYQKLHEIPRIISQEKQNLRNYSLTLNLRNNSISNLDRVEDPEYRYLVNLTIPNNSLISLNESVLPDSLQVLDIRGNNFTYLEPSVIDYFNKTDITLSLGENPWICDCKLTDLQSFLRIQELKVLDFYNIRCTNFNETLIDVTEGDLCPIILPPEVIIASTVISMFLILSGVLATVTFWKYKEEIKVWLFTHRLCLWAVAHEEFDNKKYDAFISYSNKDEEFVNSDLVPGLESGDPKYKVCLHSRDWLPGAYIQQQITQSVEASRRTIVVLSSNFIENVWGHLEFKTAHCQALKDRHNRVIVIVLGEVPPENELDEELKLYLSTRTYLQFGDPKFWEKLRYAMPHPHDLIYKKQRKRKDTDKLELVKSDSKQSK

>*Litopenaeus vannamei* Toll3 AEK86517.1

MWSVGRRDGKVLVTVWQLLAFIVCCDSSAVHYNSEECTNQDINARERALTCSLKTLDDDLRVANLTSVAVDSVARLSLVCNDVYFFQSVLSPYTLSGFVRVRELNVEFCKISELKDNAFINLRNLRNLTLRTRNLDWPVMSLTAKPEVFRPLHQLERLDLSTNNIWELPAGAFCHLANLKLLNLSHNHLQDITQLGFGGGSSDRSVSSCRSDVSSLDLSHNDVTVLVSGSLQGLEQLQHLYLQNNELGKVDDNAFQGLRSLHTLDISNNRLVALPEDAFAHTPGLMYCRARNNSLSVLAPGLFGGLDHLVELDLSYNELKSEWLTSSIFQGLVRLMLLDLSHNKISQLNQQVFSDLYTVQFLRLSHNQLKTIPAAAFAACVNLHTLDLSYNQLTSVPDKAFQGVGVLSFLALDNNNISEVGPNSLKNLSSLADLNLNGNELTAIPEAVAHLKYLKTLDLGENQISDLANMPVKGLEFLYGLRLVNNKIRGNLTKDTFSDIPSLKILNLAKNSITAIETGTFDKNLNLQAVRIDANQLSSINGLFEKLPNLLWLNVSDNNIEVFDYHFVPQSLEWLDLHKNKISELGNFLERHDLNLQTLDASFNKLQYINSIQIPDSVQLLFLNDNKISVVEPFTFFKKVNLTRVDLFANQLSRMDMSALRLSPVPVGKSLPEFYLGGNPFICDCNMEWLQRINALEHRRQHPTIMDLESIYCQMPFARTGAFIPLVDVNPSQFLCQYETHCFALCHCCEFDACDCEMTCPDGCGCYHDQSWRSNIVDCSQQDVQQVPDRIPMDATQAYLDGNDLRNLSSHSFIGRKHLQILYVNASNVRSLDNETFSGLSRLTALHLEDNLLEALRGNEFQGLEVVRELYLHNNRLRYVHQHTFAMLFHLEVLTLHNNHLINFPVWRLVDNPYLNHVSLSTNQWSCQCQFVESFGIWLNGNERKVSDAREIKCYTDVAEEEPGSYIMEFNVTTCMNTSSSSTVVRPIVLDNLLHPVIATCVAFVVVVILLLCFVYRGTIRVWIYSQCGYRMCHKNVSSDDRDKLFDAFVSYSSKDEAWVNQVLAGELERGDRPYRVCLHYRDFPVTAYIAETIVEAVESSRRTIIVLSKNFIENEWCRFQFKSAHHEVLKKRRQRLIVIVLGEIPARDLDPDLRLYLKTNTCIYASDKFFWEKLRFAMPDVQNSQRVVHTYSSIPERSSSSANKYSVNSPASMHHNLHGGTDAYWGLTRLLVVLPNCSGRVEL

>*Litopenaeus vannamei* Toll4

MVSVSAHVPGRAFVLMAFLVLLGGVGGRPPECEWKLENEGVTAGGSEQVRTTCHVRTLSAALLTEGNGNGSLSALSHATHVTVLSLHCSRRVVFESEITAGMFAVFPRLEDLQILGCKVTDLPPRSLAGLPHLRRLTLRAHHQDWPGAALALHEDALADLNRLESLDLSYNALWSLPPSMLCGLPSLTALNLTHNRLHHLPDLGLGGSCSQSTDLDRESERSLPLHHLDLTYNQVAEVPKKAFHSARDLQSLSLKHNRLAHLADWAFGGLQTLRLLDLSHNRLVAVPRTALSDLHQLRELRLANNSLSVLSPAAFGSLGQLLTLDLSHNQLSLGASNAEPFTGLIRLVVLDVSHNRLVHLGPDTFHDLYSLQVLRLSHNQLSHLADATFASLANLHTLDLSQNQLGTLSGKALQGLSVLTHLSIDYNQLEVVHEASLDNCSSLHQLSMAHNQLQMLPEAVKRAPRLRTLDFSHNQIPSLEEGVLHGLVHLQELRLSDNALSNISRTAFTEVSSLILLDLSNNSISEIEYGAFDATPKLKGLDLHHNLLGDANGLVIHLENLVWLNVSFNTITWFDYALVHKNLEWIDLSNNMISKLENFYQVQKSIGLQKLYASYNNISEISATIIPDGIKELHLHHNSISYVASNTFLDKMSVSLIDLRYNSLTLLEEAALRLSPRQSPAPLLLLSHNPLECDCGADWLLRAAGAGLSGPMTGGSILPHLGDVGSVQCRLPGLWHGAMVPLIVVQPQQFLCTYRRHCFTLCHCCDFDACDCEQTCPRNCTCYHDHTWTHNVVDCGGGWGSMPSGVPMDVTEAFMDGNKMGILTSHALIGRKNLRVLYLNHSDISAIQNRTFNGLKNLQVLRLDHNKIEALHGFEFIDLHGLRELYLNNNHLRHLSNVSFSSLRAIELLRLDNNYIVTFPVWNLALNPFLLEVSLYHNPWSCECSYLANLRAWLEANRIKATNASLVRCRHNSTGMMGPPVLSDTPLRCDHYVATTRINSLIIHDYVMLLLITAALVLLLVGAAVTVVAYRRRLKLWAVSRYGKRLFEKSSAYVEEREKLFDAFVCHSAKDSTWVCGLMAPELEASGYRLCVAHRDCTAPSAPVAGRAIAESISCSRRIILVLSRGLVDAEWCRYDFKSATVDALSSVKHRHVVVVLLEDVPRSEMDPELAAITRTAGTTLHPRDPRFWEKLRRAMPSLRPRLRQGLAGTIGGKASSRPLVSAEHQNGPSWPLPETKTLGHTSAKSLIINPYWETAVGSNVSEAAWCSRNGPDLGAPPWVSSPSKQAPSTSPENEARLVGSPTGSTAEGQDHTYMSVSECGEVRASLLPNNTNASTLGAKPSPCDVPSGADRGSTGEKAEGSSRPSAPPLFRRDAPDYLTRSWIFHPPPNEQPPPPGQTYFV

**>***Litopenaeus vannamei* Toll5

MPGWGGWSAFLHQTLLLVSVVAPATTLAQTPSATPVSSAEALSASSNDTSKGSPWCQWVAQEALECHIRTLEDSLGSQVVKGHWQDRNDTSGIPAAGQWPRDQVPGLPGAWAARSDTRHLRVLGVECSQVLYYQSRLTARTFQGLENVEELAINNCKLDSLPPGTLSSLGRLRSLAVTTHNGEWAALAMEVVSGSLPLTLERVSLAHNNIWTLPPRAFCGPNSLHHLDLSHNRLQDVHELGFMDQVQLDILMMNMSNTSFSEHSAVFSNLDEESSAERCGQALRELILDHNDLVRLPDGSFRVLSGLRELHLRDNDIRLISSEAFSGLTALQVLYLSNNHIIALHNGTFSDNIALERLYLNNNSLSALNSKVFQDMKELQVLEISNNKLYLDNSHDDLFKGLRRLVILDLSWNSLTTITKLLFRDLTSLQRLVLSHNAIQSLEDDSFTSLSNLYALDLSHNLLLTLGEANLRGLVGLSLIHLANNSLFEIHPHAFRHSSNLKQVFLSHNHLQAIPKALENLSFIKTLDMSYNNIVSIQPFHFGGLGNLEMLNVSHNKLDFISQGSFKGLASVKDLDLRDNAIHTVNEGSFDGVPNVLNLVLARNQLSNIDHIFAGLQHLESLDLSENNIRMFDYAFIPQQLINLDLKKNKIGQLGNFFKVHTVLTLENIDASHNSIKSLTELSLPNTIMHVILHHNNITRILPNSFRDKVNLQTLDLSMNSLQRINPKSVSMRVASGKHTSAQIYLSGNPLICDCEMEWLYNSFRSTLTTTPEATEVTFLQPRIDDLARVTCTLLHSREDTTVMTRVLETSAANYLCPYTTHCFTLCQCCDFIACDCQMKCPDACSCFHDDTWSINLVDCSGGHLDRLPDRVPMDATVVLLDGNNLQILHAHHFIGRHSIQQLYLNNSQIQTLQNRTFHGLTSLQVLHLQDNMIVQLNGFEFSGLHHLKELYLQNNRLSFINNATFIGLKSLEVLRLDNNFIIDFPVWLLSNNRYLASVTLGNNPWDCDCQFVESLREWQKQQSHLLINPEDVFCVHGDSGVVGPSIILPEYSCTEAQHGVTQYKFGQQELPFLAGGLCGGVALISALVVMAMLVARRRAAAANKLGINGSPAYCQEEDGKVFDSYISYSANDASFVRDVLATKLENSCPSYKLCLHSRDFSENSRLSEFITQSLGFSRRTIIVLSKNYIDNEWKNAIFKKAHVDGLKDNDMGIIAIYYDNVSYSSFDSDLKNIMRRCIKLRWGDKNFWKKLSEAMPIKQTYAGLPVYVSENSYKSSTLPTLIPPSSLPLPSSSSVLTSTTGLTTPSEIGHRPSCQQEPPATTYKAPPPPRPCYTPPSCDYIVMTGRDCRDPQCTCHRHSHTAYTYVDGDSSSLHTYTSLEPFTLPDPHVPESYTRGHSPASSHYSALEPPVRRTVRASKRKKKRPLSQNCPPVHCDTLENPAFTEDVHGELPSNGTFRRTKSLRASRGNQERHSDYSTDHSSDRSSGRSYDHSVTSGGGVVDRNDALYMGLADSPPEPTMVTTEECFV

**>***Litopenaeus vannamei* Toll6

MKNTVALYVFASVFASCSVAFVYNAPENCDWTFRDEAQREVSLSCSLRTIGNDFDSSNFSIAQSEHTTELEILCSDVLFFQSSLQPRVFQRLYNLDTISIEFCKLTSLPAGAFLGLDAMKALAVRTHNSDWSAMALELNPDSLVGMPHLERLDLGQNNIWNLPERVFCPLPALRHLNLTWNRLQDVSEVGVSGSCGAHLVTLDLSGNDLVVLPEAGLAGLESLRELYLQYNDVSMLADGAFSGLSTLSVLNISSNRLVALPPEVFNETLGLTELHLQNNSLSVLAPGLFSALSRLTVLDMSFNQLTSEWVTAETFRGLLRLVVLNLSHNRLTQVTLDMFRDLSTLQVLDLRHNSLTVLGDMTFSPLANLHRLDLSYNSLVSVESRGLSGLHVLASLSVAHNNISRIAPEAFQNCTSLRDLRLEYNLLEEIPEAVREASSLRTLRISHNQLSAVAQGDLTSLSALRHLDMSNNFLRGLCKSCLAGLEYLEVLDLSQNELSTVPHGAFDTNTGLQLLRMDGNKMSDINGLFASLSNLLWLNVSDNRISWFDYALIPDQLQYLDLHNNRIRDLGNYFSLESKLELRTLDVSHNQLESLSASSVPDSVELLFVNSNKITRIATGTFAEKRNLSMVDLYDNLLSKIDLNSINLPRVPEERDLPEFYIGGNPIFCDCNMEWMHRVHQISSLRQHPRVMDLDKVTCTLPYPRSAENRVPFLETQPSQFLCPYTSHCFALCHCCDFIACDCQMTCPIGCSCYHDGTWATNIVDCSARNHHQLPDDIPMDATLVYMDGNEMPFLDAHHLIGRKNMRALYLNSSRVERIQNRTFHGLSTLKELHLHENMLVELEGFEFEHLEHLRELYLQNNRLKVINNVTFAGLKSLEVLRLDGNFLFEFPVWHLKLNKGLKDVTLGINLWSCECQYMVDFKNWLIRETDVVKDAKSIFCVSNSTGEPGPYVLESSYSCENFVATSIVQEKLENDFLQPVLITLAIFFVVLVMGVVFAVFRVRLQASVSKKCGLKCFPSQPAPAKEEERNMLYDAFVSYSEMDAPFVTEVFAAELENGDPSYKVCLSSRDYQTVGSYVGDFIVQSIETSHKVVLVLTKNFVDHDWCKFSFKAAHVEALKSLKNRVIVVMCGDVDESDMDSDLSGIVKSATKLKYEDKSFWSKLHAALPGGAKKMSQQCYITETNYIMRNSVPVLSPNHNLKQSQFMPNMVLTNTLKTPVSHYHQQHHYQHTQPSINSDTGDLDKTFVSVETAQSSLAPSLNHSYMSIDYAAARNSHIYASIDETTPALPPSTLPSVHTLHQHLRQQQHVEPLRQYLPQDVLSSQQRRPLTGAPMQTFDQPAVSASYFI

**>***Litopenaeus vannamei* Toll7

MPFPAWQLVLISAVVSHEVRLEPHLQGAIGPSYSLSESSASPPPRLGQNGTVPQSHPELAKEESVASEAQEQDVPNCAVEINHATLFDRAFLCPRAESLYKLFENFKRESSNMRVKITVKKVDGPLTLQPPPNIIIALFLPNANVTRLSNGTANQALPHAPLASLVQLDLRNNPLLELEGMQWLGVLRSLTVLILRETPVTDRELPPLLARLPLLQHLDISSSGLPYLPEEAFNKNPELKHLDLSNNEFKKITLPENLIRGLTHLNISSCRLEEVSVPAAAWEERWSTPSSQGRLSTLDVSKNRLKWLPSRLVEALNIFSYVIIKDNLWNAACTRCPLYHLWQYSRRASRDVVGKEELDCFRQDVLLSCGWDNCPTECYCDDRNKTVNCTGKGLVALPTIVPSETETLVVDDNAISNLNNLASPTYCSLRHLSVKRNRVTELLLSEEGKCECHTQEYYPKTPKCFPQHLHTVSLEDNRIEGLTASDCPLLYPLHTLRMPRNKLDSLGAQVCGSLGRLRALDLAHNSIASVTSSDLATYPFLQSLNLSHNALEKLVPNFPPEFTSLDVSPNCLWRLTSEDKSLPRTPYRTEKSLEDLKLTEGDNCTTPGRPGRGGEEEEEEKRSSVCDGMKEKVFMIFISWVIITIILLYVCYVYARQGRCWSSREGTIAQENEDTSECKYSVFVVHSSHDREIVRNKIIIPLFQRGYSVAWHENVFVPGAWILENIERAVRNSQSMVVFATDNLAASRCSLQEIRRGRYEEMDREGFRIMALVTETLPRALKKELYEIVALRTHIQYSDRDYIEKICNFLPPPRPVPLQDASLPNAFSIRSQLDRFDEMRRENRNQSMSNVVHLHRDNEAFVIDQYTASITSGTWKAPWPSMSEEEMHQMLSRYLKSPEMPAECYAETRSLNYVLLEQDNEDESSACMDVFSRLSVTRTTCRF

**>***Litopenaeus vannamei* Toll8

MWILWVWCLAALLWAVTGNVGPDYHTPDDCQVTSLPGTDLVALLCRLRTNNSELDATNFSLASTRNTVKLRIECSDVIFFQSALQNKSFVRLRELQELDIEYCKIGEVPREAFLGLTNLRNLTLRTYNADWSVVTLKIANDAFMDQKKLEYLDLGNNNIWTLPPALLCELENLRLLNLSRNKLQDVTVLSFSQAEHICAPGLRSLDISFNHLVSVPAFAFAALKSLQVLNVSLNGISKLEDKALFGMYSLEVLDLSGNLLTTLPPELFQENKRLTKLYIRNNSVSVLAPGLFTGLSLLLELELSDNQLTNTWVNSETFTDLLRLASLDMSNNKITRLDAATFRDLTNLQVLKLQQNMIETISDNTFSGLFRLHTLVLSDNRVKMISDKTFGGLIGLQVLKLDGNEVFSIDSEALVNSTGLQELQLSHNHLQDVPKVVKSLASLRILDLSGNHVSVISNKSFPDLPHLSVLRLAANDIENVTKSVFTNLPSLQVLDLSSNRIFTVENGAFDNNRHIEAIRLDDNILTNVKGLFSNLPNLQWLNLSKNHLEMFDYAFIPRGLKYLDLRSNNINELGNHYEIEGEPHLKIIDASFNRLSDISASSFPDSVEIIFLNNNLIESVQPFAFFSNNNLTKVDLYANKIRNLDQTALRLSQMDPSRDLPEFYLGGNPFECDCTMEWLQTINSYDQPHQRLMVIDLDSIECRLMNNKGKIPLLDAKKLQFLCEYDSHCFTRCKCCDFDACDCEMTCPANCTCFHDNSWAANIVDCSRAGYGSVPERIPMDSSEVYLDGNALSSLSSHTFIGRKNMRVLYLNDSGVEVIHNRTFNGLKLLETLYLHRNSIKELKGYEFEHLTLLRELYLHDNELFFIQNTTFLTLASLRVLRLDNNRLKTFPVNMFSKNHNLHSLHLSENPWTCDCENLRDIQAWLQGVGRKLRDADKIYCALNGSSEVVAQVSTYNHSSCNNETETTTIRHEAYLDYVFLPTITLGAFAVLLTITLIVFCNRNRMRVWVYAKYGVRLFYRSEYEGDTDKAFDAFVSYSSKDEVFVTQILAPELERGSPAYKLCLHYRDFPVGAYITDTILSAVETSKRTILILSENFIKSEWCRFEFRSAHHEVLKDRRRRLIVILLGDVPQRDLDPDIRLYLKTNTYLKWGDTHFWEKLKFAMPDAQPPTRNHQLHAIAAQQQPGPRPVPLHT

**>***Litopenaeus vannamei* Toll9

MLVSRLTALVFGLLVVLVSLSLGAAIRGASGFPHSSLRRVKSPVSQNDFLGPSDSKGSDERHGEATVGEVPRTLDLSLNVKVTENDRYDNDAVSYVSQAGGNGYAAEKRATEELVKNGERLTSSAMTNEVLTTKTKTQERKEASKNQVPFLSSTKSFQKSEESALVSEDPTFPSTGKGRIKEEKGDTAKNETRSSHAKNRCEFRSAPPDVVFPDAFEEFSRSMEANLSPDDQPDEELLDSLLPDGCHYTERQKKKVMCTGANMTSIPEFEHARNIETLHFSGTSIVQVTNLDPLPRSLKALYFSNGMLKVFDGRNLNRVSGLEVLHLDNNFITSWSLVTTFYSMGGFAEQNTIKTLNIRSNQITYPPQPVGDNETVLPYLETFVLSENPLCYLPDTLFKPLRNSNVTSLYLKNCNIDEFYGSPLSYLPNLEVLDLTGNRAINETELRDLLLPLGRLKQLFLGNNNYQTVPTKALSLVNGTLENLDLHSSTFTCLDNSSFPIMPVLTHLDLKYCRINAIREHTFQGFPMLRELNLDGNSLTTVPPEVLLPSLQILTLSDNPRANGNDGDQRFSMEDVSFQDMVNLKTIQLNQVIMEKIERSYFNDLYNLEELSLTGCGIKTIENFSFVNLTKLQHLNLSENYITTLYNDSLVGLVNLISLDLSNNKLKGINRMGRSGVSSSAVRTDSLSSGTVSDAREIDSFLKRVRAPSPLIPWLSSIRRNARAVGPLGWQGKDLPRTTIAAYAFSDLVRLRTLNLSENMIMLLPPELFHNLTNLLILDISYNRLMTWDDPVLGSIPNLTELHLRTNLLDGITDAMEVDFRKESLKLVDLQDNTFKCDCSLSKFNRSLNTSNFLNWPYLCREGKADVDMEEYIARAPCNFATQPENHGRMRAIVISTIVSSLLLVASVMVYRKRWYVRYFMYTVRMRTKVVREEADKYLYDTFVCYSQTDRQWVFEHLVAKLEDGGRYRVCIHERDFTVGQEITDNIINSVERSRKVVVVLSPAFIRSSWCMFELQMASNKILDERKSKLIMLLLDHIPDEEQPKKLKYLLKTRTYIEWVPDLESQKLFWARLMRAISKPSDSEAIAASTKL

>*Bos taurus* TPA: Toll-like receptor 1 DAA28727.1

MPDILSLSKLKILIISHNRIQYLDLSVFKFNQELEYLDLSHNNLEKISCHPTLNLKHLDLSFNPFDALPICQEFGNMSQLEFLGLSATQLQKSSVQSITHLHISKVLLVLGDTYGEREDAESLQDLKTQSLHIVFPTGKEFHFILDVSVGTTVSLELSNIKCVLDDNGCPYFENVLSKLQKNSRLSNLTLNNIEITWNSFFTILQLVWRTNIEYFSISNVKLQGYLDSRDFDYSDTSLKALSIHKVVHDVFSLPQGYVYKILSNMNIQHLTVSAAHMVHMVCPSQISPFLYLNFSNNLLTDTVFINCTNLANLKTLILQKNQLKELVNIVHMTQEMKSLQQLDVSQNSLMYDESEGNCPWARNLLSLNMSSNILTDSVFRCLPPQIKVLDLHNNRIRSIPKDVTGLETLQELNLASNSLAHLPGCGIFSSLSILIIDYNSISNPSADFFQSCQKIRSLKAGNNPFQCSCELRDFIQSIGQVSSDVVEGWPESYKCDYPESYKGTPLKDFQVSELSCNTALLIVTIVVPGLVLAVAVTVLCIYLDLPWYLRMVCQWTQTRRRARNVPLEELQRTLQFHAFISYSGHDSAWVKNELIPNLEKEDIRICLHERNFVAGKSIVENIINCIEKSYKSIFVLSPNFVQSEWCHYELYFAHHNLFHEGSDNLILILLDPIPQYSIPSSYHKLRALMAQRTYLEWPKEKSKHGLFWANLRASINIKLMEKAAEIH

>*Bos taurus* Toll-like receptor 2 ALL55248.1

MPRALWTAWVWAVIILSTEGASDQASSLSCDPTGVCDGHSRSLNSIPSGLTAGVKSLDLSNNDITYVGNRDLQRCVNLKTLRLGANEIHTVEEDSFFHLRNLEYLDLSYNRLSNLSSSWFRSLYVLKFLNLLGNLYKTLGETSLFSHLPNLRTLKVGNSNSFTEIHEKDFTGLTFLEELEISAQNLQIYVPKSLKSIQNISHLILHLKQPILLVDILVDIVSSLDCFELRDTNLHTFHFSEASISEMSTSVKKLIFRNVQFTDESFVEVVKLFNYVSGILEVEFDDCTHDGIGDFRALSLDRIRHLGNVETLTIRKLHIPQFFLFHDLSSIYPLTGRVKRVTIENSKVFLVPCLLSQHLKSLEYLDLSENLMSEETLKNSACKDAWPFLQTLVLRQNRLKSLEKTGELLLTLENLNNLDISKNNFLSMPETCQWPGKMKQLNLSSTRIHSLTQCLPQTLEILDVSNNNLDSFSLILPQLKELYISRNKLKTLPDASFLPVLSVMRISRNIINTFSKEQLDSFQQLKTLEAGGNNFICSCDFLSFTQGQQALGRVLVDWPDDYRCDSPSHVRGQRVQDARLSLSECHRAAVVSAACCALFLLLLLTGVLCHRFHGLWYMKMMWAWLQAKRKPRKAPRRDICYDAFVSYSERDSYWVENLMVQELEHFNPPFKLCLHKRDFIPGKWIIDNIIDSIEKSHKTIFVLSENFVKSEWCKYELDFSHFRLFDENNDAAILILLEPIDKKAIPQRFCKLRKIMNTKTYLEWPVDETQQEGFWLNLRAAIRS

>*Bos taurus* Toll-like receptor 3 ABN71667.1

MSRPLPYHIHFFSGLLTCWILCTSSAHKCTVRHEVADCSHLKLTQIPDDLPTNITVLNLTHNQLRRLPPANFTRYSQLTILDGGFNSISKLEPELCQSLPWLEILNLQHNEISQLSDKTFIFCMNLTELHLMSNSIQKIKNDPFKNLKNLIKLDLSHNGLSSTKLGTQLQLENLQELLLSNNKISSLTPEEFDFLGNSSLKRLELSSNQIKEFSPGCFHTLGELSGLSLNNAKLSPSLTEKLCLELSNTSIENLSLSSNQLDTISHTTFDGLKQTNLTTLDLSRNSLRVMGNDSFAWLPHLEYLSLEYNNIEHLSSRSFYGLSNLRRLDLRRSFTRQSISLTSLPKIDDFSFQWLKCLEYLNMDDNNFPGIKRNTFTGLVRLKFLSLSNSFSSLRTLTNETFLSLAGCPLLLLNLTKNKISKIQSGAFSWLGHLEVLDLGLNEIGQELTGQEWRGLDNIVEIYLSYNKYLELTTNSFTSVPSLQRLMLRRVALKNVDCSPSPFRPLPNLVILDLSNNNIANINDELLKGLEKLEILDLQHNNLARLWKHANPGGPVQFLKGLFHLHILNLGSNGFDEIPVEAFKDLRELKSIDLGMNNLNILPQSVFDNQVSLKSLSLQKNLITSVQKTVFGPAFRNLSYLDMRFNPFDCTCESIAWFVNWINSTHTNISELSNHYLCNTPPQYHGYPVMLFDVSPCKDSAPFELLFMININILLIFIFIVLLIHFEGWRISFYWNVSVHRVLGFKEIDRAEQFEYAAYIIHAYKDRDWVWKHFSPMEEEDHTLRFCLEERDFEAGVLELEAIVNSIRRSRKIIFVVTQNLLKDPLCKRFKVHHAVQQAIEQNLDSIILIFLEEIPDYKLNHALCLRRGMFKSHCILNWPVQKERVNAFHHKLKVALGSRNSAH

>*Bos taurus* Toll-like receptor 4 AAQ62700.1

MMARARLAAALIPATAILSCLRTESWDPCVQVVPNISYQCMELNLYKIPDNIPISTKMLDLSFNYLRHLGSHNFSSFPELQVLDLSRCEIKIIEDDTFQGLNHLSTLILTGNPIQSLAWGAFSGLSSLQKLVAVETNLVSLNDFPIGHLKNLKELNVAHNFIHSFKLPEYFSNLPNLEHLDLSNNKIQNIYYEDVKVLHQMPLLNLSLDLSLNPLDFIEPGTFKEIKLNGLTLRSNFNSSHVMKTCIQGLAGLKTNRLVLGEFKNERKLQRFDRSFLEGLCNLTIEQFRIAYLDKFSGDDTDLFNCLANVSVISLLSISLGSLQALLKDFRWQHLEIINCDFDKFPALKLSSLKKFVFTDNKDISTFTEFQLPSLQYLDLKRNHLSFKGCCSHTDFGTTNLKHLDLSFNDVITLGSNFMGLEQLEHLDFQHSTLKQINAFSAFLSLRNLRYLDISYTNIRIVFHGIFTGLVSLQTLKMAGNSFQNNLLPDIFTELTNLTVLDLSKCQLEQVAQTAFHSLSSLQVLNMSHNKLLSLDTFLYEPLHSLRILDCSFNRIMASKEQELQNLPRSLTWLNLTQNAFACVCEHQSFLQWVKDQRQLLVGAEQMMCAEPLDMEDMPVLSFRNATCQLSKTIISVSVVTVLLVSVVGVLVYKFYFHLMLLAGCKKYGRGESTYDAFVIYSSQDEDWVRNELVKNLEEGVPPFQLCLHYRDFIPGVAIAANIIQEGFHKSRKVIVVVSQHFIQSRWCIFEYEIAQTWQFLSSRAGIIFIVLQKLEKSLLRQQVELYRLLSRNTYLEWEDSVLGRHVFWRRLRKALLAGKPQSPEGTADAETNPQEATTST

>*Bos taurus* Toll-like receptor 5 AAT48489.2

MGDCLDLLLGVVLLTSPALGMSSCFFDGWRAIYLSCNLTQVPQVPNTTKSLLLSFNYIRTVTTASFPFLEQLQLLELGTQFTPLTIYREAFRNLPNLRILDLGGSQINFLHPDAFQGLPHLTKLRLFSCGLSDAVLKDGYFRNLASLTHLDLSKNKIQSLYLHPSFRELNSLKSIDFSFNKIPIVCEQEFKPLQGKTLSFLSLADNQLYSRVSVDWNKCLNPFRNMVLETLDVSGNGWGVDIMRNFSNAINGSQIFSLVLTRHIMGSSFGFSNLKDPDYHTFAGLARSSMIQLDISHGYIFSVNFRIFETLQELKVLNLAYNKINKIADEAFYGLDNLQVLNLSYNLLGELYSSNFYGLPKVAYIDLQKNHIAIIQDQTFKFLGKLNTLDLRINALKTIYFLPSIPNIFLSGNKLMTLPNIPLTANFIQLSENRLENLNDLYFLLQVPHLQILILNQNRFSFCHQNHAPSENSSLEKLFLGENMLQLAWETGSCQDIFKGLSHLQLLYLNPIYLNFLPPGVFHHLTALRGLSLKDNRLTVLFPGDLPANLEILDISGNQLLSPDPDLFASLSAIDITHNNFICECELSAFIHWLNQTNITIAGSPADMYCMYPNSLAGVSIYSLSTESCEEEEVLESLKFSLFILVTVTLTLFLVITLAVTKFRGFCFICYKKAQSLLFKDPIKGRESDTYKYDAYLCFSSKDFEWVQNALLKHLDVQYHSQNRFNLCFEERDFMPGENHIANIQDAVWSSRKIVCLVSRHFLRDGWCLEAFSYAQSRCLADLNGALIMVVVGSLSQFHLMRHQSIRGFVQKRQYLRWPEDLQDVDWFLNKLSQWLPKKKKERKKDMWIPLQNVPTIS

>*Bos taurus* TPA: Toll-like receptor 6 DAA28720.1

MIKDKESPIRSCHFVYIVALVFGTIIQFSDESEFVVDMSKTSLIHVPKDLPPKTKVLDLSQNNISELHLSDISFLSGLRVLRLSHNRIQGLDISIFKFNHDLEYLDLSHNQLQKISCHPITTTLKHLDLSFNDFDALPICKEFGNLTQLNFLGLSATKLQQLDLLPIAHLHLSCILLDLEDYMKENKKESLQILNTKKLHLVFHPNSFFSVQVDISANSLGCLQLTNIKLNDYNCQVLLKFLSGLTGGPTLLNFTLNHMETTWKCLVKVFQFLWPKPIEYLNIYNLTIVESIDEEVFTYYKTTLKALKIEHITNKVFIFSQTALYTVFSEMNILMLTISDTRFIHMLCPQEPSTFKFLNFTQNSFTDSVFQNCDTLARLETLILQKNELKDLFKTSLMTKDMLSLETLDVSWNSLEYDRSNGNCSWVGSIVVLNLSSNALTDSVFRCLPPRIKVLDLHNNRIRSIPKDVTGLETLQELNLASNSLAHLPGCGIFSSLSILIIDYNSISNPSADFFQSCQKIRSLKVGNNPFQCSCELRDFIQSVGQVSSDVVEGWPESYKCDYPESYKGTPLKDFQVSELSCNTALLIVTIVVPGLVLAVAVTVLCIYLDLPWYLRMVCQWTQTRRRARNVPLEELQRTLQFHAFISYSEHDSAWVKNELIPNLEKEDIRICLHERNFVAGKSIVENIINCIEKSYKSIFVLSPNFVQSEWCHYELYFAHHNLFHEGSDNLILILLDPIPQYSIPSSYHKLRALMAQRTYLEWPKEKSKHGLFWANLRASINIKLMEKAAEIH

>*Bos taurus* Toll-like receptor 7 AEE39318.1

MGDLFLYFQVFPMWTLKRQFPILFNMILISGLLGARWFPKTLPCDVTLDAPNTHVIVDCTDKHLTEIPGGIPANATNLTLTINHIAGISPASFHRLDHLVEIDFRCNCVPVRLGPKDNVCTKRLQIKPNSFSKLTYLKSLYLDGNQLLEIPQDLPPSLQLLSLEANNIFLIMKENLTELANLEILYLGQNCYYRNPCNVSFTIEKDAFLNMRNLKLLSLKDNNISAVPTVLPSSLTELYLYNNIITKIQEDDFNNLSQLQVLDLSGNCPRCYNVPFPCTPCENNSPLQIDPNAFDALTELQVLRLHSNSLQHVPQRWFKNINKLKELDLSQNFLAKEIGDAKFLHLLHNLVNLDLSFNYDLQVYHAVINLSDAFSSLKNLKVLRIKGYVFKELNSLNLFPLHNLPNLEVLDLGTNFIKIANLSIFNQFKTLKFIDLSVNKISPSGDSPEGGFCSNRRTSVEGHGPQVLETLHYFRYDEYARSCRSKSKEPPSFLPLNEDCYMYGQTLDLSRNNIFFIKPSDFQHLSFLKCLNLSGNSISQTLNGSEFQPLVELKYLDFSNNRLDLLYSTAFEELHNLEVLDISSNSHYFQSEGITHMLNFTKNLKVLRKLMMNYNDIATSTSRTMESESLQILEFRGNHLDILWRDGDNRYLKFFKNLLNLEELDISENSLSFLPLGVFDSMPPNLKTLSLAKNGLKSFSWERLQSLKNLETLDLSFNQLKTVPERLSNCSRSLKKLILKNNQIRCLTKYFLQGAFQLRHLDLSSNKIQVIQKTSFPENVLNNLNILFLHHNRFLSNCDAVWFVWWVNHTEVTIPYLATDVTCMGPGAHKGQSVVSLDLYTCELDLTNFILFSLSISAVLSLMMITIANHLYFWDVWYSYHFCKAKIKGYRRLISPNSCYDAFIVYDTKDPAVTEWVLDELVAKLEDPREKCFNLCLEERDWLPGQPVLENLSQSIQLSKKTVFVMTDKYAKTENFKIAFYLSHQRLMDEKVDVIILIFLEKPLQKSKFLQLRKRLCGSSVLEWPTNPQAHPYFWQCLKNALATDNHVTYSQVFKETA

>*Bos taurus* Toll-like receptor 8 ABA39705.1

MTLHFLLLTSLFLLISDSCEFFTEASYPRSYPCDVKNENGSFIAECNGRRLQEVPQTVDKDVTEVDLSDNFITRVTNESFQGLQNLTKINLNHNAKSQSGNPAVKKAMTITDGAFLNLKHLRELLLEDNQLQQIPAGLPESLKKLSLIQNNIITLTKKNTSGLGNLESLYLGWNCYFACDKKFTIENGAFQNLTKLKVLSLSFNPLHSVPPSLPSSLTELYLSNTHIGNVSEEDFKELSNLRVLDLSGNCPRCFNAPFPCVPCQGDASIQIHPLAFQTLTQLRYLNLSSTSLRKVPASWFDNMHNLKVLDLEFNYLMDEMPSLEILDLSYNYELKKYPQYINISKNFSKLISLQMLHLRGYVFQELRKEDFEPLRNLSNLTTINLGVNFIKQIDFSIFHWFPNLKIIYLSENRISPLVSDTEQHDANGTSFQSHILKRRSADIQFDPHSNFYHNTRPLIKTECSRLGSALDLSLNSIFFIGVSQFKDFGNISCLNLSSNGNGQVLNGTEFSCLSGIKYLDLTNNRLDFDDDAAFSELPLLEVLDLSYNAHYFRIAGVTHRLGFIEHLTNLRVLNLSNNDIFTLTETQLKSASLGELVFSGNRLDLLWNAQDVRYWQIFQNLKDLTRLDLARNNLRNVSSQAFLNLPQTLTDLHINDNMLKFFNWTLLQQFPRLELLDLSGNQLFFLTNSLSTFASSLETLLLSRNRISHLPSDFLSGASSLIHLDLNSNQLKMLNRSTFETKTATKLTVLELGGNPFDCTCDFGDFLEWMDRNLNVRVPRLTDVICASPGDQEGKSIVSLDLSTCVSDTIAAIFCFLTFSVTISVMLAALAHHWFYWDAWFIYHVCLAKVKGYRSLSTSQTFYDAYISYDTKDASVTDWVMNELRFHLEESEDKNVLLCLEERDWDPGLAIIDNLMQSINQSKKTIFVLTKKYAKNWNFKTAFYLALQRLMEENMDVIVFILLEPVLQHSQYLRLRQRICKSSILQWPDNPKAEGLFWQSLKNVVLTANDSRYNNLYVNSIKQY

>*Bos taurus* Toll-like receptor 9 ABN71658.1

MGPYCAPHPLSLLVQAAALAAALAEGTLPAFLPCELQPHGQVDCNWLFLKSVPHFSAGAPRANVTSLSLISNRIHHLHDSDFVHLSNLRVLNLKWNCPPAGLSPMHFPCRMTIEPNTFLAVPTLEELNLSYNGITTVPALPSSLVSLSLSHTSILVLGPTHFTGLHALRFLYMDGNCYYMNPCPRALEVAPGALLGLGNLTHLSLKYNNLTEVPRRLPPSLDTLLLSYNHIVTLAPEDLANLTALRVLDVGGNCRRCDHARNPCRECPKNFPKLHPDTFSHLSRLEGLVLKDSSLYKLEKDWFRGLGRLQVLDLSENFLYDYITKTTIFNDLTQLRRLNLSFNYHKKVSFAHLHLASSFGSLVSLEKLDMHGIFFRSLTNITLQPLTRLPKLQSLRLQLNFINQAQLSIFGAFPSLLFVDLSDNRISGAATPAAALGEVDSRVEVWRLPRGLAPGPLDAVSSKDFMPSCNLNFTLDLSRNNLVTIQQEMFTRLSRLQCLRLSHNSISQAVNGSQFVPLTSLRVLDLSHNKLDLYHGRSFTELPQLEALDLSYNSQPFSMQGVGHNLSFVAQLPSLRYLSLAHNGIHSRVSQKLSSASLRALDFSGNSLSQMWAEGDLYLCFFKGLRNLVQLDLSENHLHTLLPRHLDNLPKSLRQLRLRDNNLAFFNWSSLTVLPRLEALDLAGNQLKALSNGSLPPGIRLQKLDVSSNSIGFVIPGFFVRATRLIELNLSANALKTVDPSWFGSLAGTLKILDVSANPLHCACGAAFVDFLLERQEAVPGLSRRVTCGSPGQLQGRSIFTQDLRLCLDETLSLDCFGLSLLMVALGLAVPMLHHLCGWDLWYCFHLCLAHLPRRRRQRGEDTLLYDAFVVFDKVQSAVADWVYNELRVQLEERRGRRALRLCLEERDWLPGKTLFENLWASVYSSRKTMFVLDHTDRVSGLLRASFLLAQQRLLEDRKDVVVLVILRPAAYRSRYVRLRQRLCRQSVLLWPHQPSGQGSFWANLGIALTRDNRHFYNRNFCRGPTTAE

>*Bos taurus* Toll-like receptor 10 ABU86947.1

MRYIRSIYIFCSIVTSVRSGASELPEERELTTNFSSMSLTKVPEGLTPITTTLDLSYNLLFQLQHSDFRSLSKLKVLILCHNRIQELDIKTFEFNKELSYLDVSNNRLKSVTWFSLAGLRHLDLSFNDFDTLPISVETGNMSHLETLGLSGAKIQKSDFQKIAHLQLNTVLLGLRTLSHYEEGSLPILNTTRLHIVLPVNTNFWVLLHDGIKTSKILEVINIDLQKSQFTSYESQQNPILENAKTSILLLNKVDLSWDDLFLIFQLVWHTSVEYFQIQHVTFGGKVYLDHNSFDYSNTVMRTIKLEHVHFRIFNIPQESIYLLFTKMDIENLTISDAQMPHMLFPMYPTRFQYLNFANNILTDDVFKKSIQLPHLKTLILKDNKLETLSLVSHFASNTSLRHLDLSENLLQHENDENCLWPETLVTMNLSFNKFADSVFGCLPRNIQILDLNSNKIQTVPKAITHLTSLRELNLAFNFLTDLPGCSHFRRLLVLNVEMNLILSSSLDFFQSCQEVKTLNAGRNPFRCTCELRDFIQLGKYSEGMMVGWSDSYICEYPLNLKGTQLKDVHLPEISCNTGLLIVTIVVVMLVLGMAVAFCCLHFDLPWYLRMLHQWTQTWLRVRKTTQEQLKRSVQFHVFISYSEHDSAWVKYELIPSLEKEDGSVLICLHEGNSDPGKSMTEDTINCIEKSYKSIFVLSPSFVQTEWCHYEPYFAHHNLFHESLDYIILILLEPIPLYCIPTRYPELKALMEKKAYLEWPKDRRKCGLFWANLRAALHVNLLDTRGTCELQTFTELNEGFGGSAISLIRTDCL

>*Felis catus* Toll-like receptor 3 ABB92548.1

MSQSSPYIYSFLGLLPFWILCTSSTNKCVVRHEAADCSHLKLTQVPDDLPANITVLNLTHNQLRRLPPDNFTRYSQLTTLDGGFNSISKLEPELCQKLPLLEILNLEHNELSHLSERTFIFCVNLMELHLRSNSIQKIENDPFQNLKNLIKLDLSHNGLSSTKLGSQLQLENLQELLLSNNKINSLRREELDFLGNSSLKKLELSSNPIKEFSPGCFHAIGKLFGLSLNNAQLNPNLTEQLCLELSNTSIQNLSLSNTQLYRTSNMTFVGLKHTNLTVLDLSHNNLNVIDNGSFVWLPRLEYFFLGYNNIEHLFSHSLYGLLSVRYLDLRRSFTKQSTALTSRPKIDDFSFQWLKCLEYLNMGDNNFPGIRSNMFTGLIKLKHLSLSDSFTSLRTLTNETFLSLAQSPLVTLNLTKNKISKIESGAFSCLGHLQVLDLGLNEIGQELTGQEWRGLGNIIEIYLSYNKYLQLTPGSFALVPSLQRLMLRRVALRNVDSSPSPFHSLRNLVILDLSNNNIANINDKLLEGLEKLEILELQHNNLARLWKRANPSGPVYFLKGLSHLHILNLESNGFDEIPAEVFKGLSELKSIDLGLNNLNIFPLSVFDDQASLKSLNLQKNLITSVEKDVFGPAFRNLSNLDMSFNPFDCTCESIAWFVNWINSTHTNISDLSSHYLCNTPPQYHGFPVMLFDISACKDSAPFELFFIINTSILLIFIFTVLLIHFEGWRISFYWNVSVHRVLGFKEIDRQPEQFEYAAYIIHAYKDRDWVWENFSPMEEKDQTLRFCLEERDFEAGVLELESIVNSIKRSRKTIFVITQHLLKDPLCKRFKVHQAVQQAVEQNLDSIILIFLEDIPDYKLNHALCLRRGMFKSHCILNWPVQKERVNAFHHKLQVALGSRNSIH

>*Felis catus* Toll-like receptor 4 BAB43947.1

MMPPTRLAGTLIPAMAFLSCLRPESWDPCVEVVPNITYQCMDLNLHKIPDNIPSSTKDLDMSFNPLRNLGSHSFSNFPELQVLDLSRCEIQIIEDDAYQGLNHLSILILTGNPIQRLFPGAFSGLSSLQTLVAVETNIASLEDFPIGHLKTLKELNVAHNLIHSFKLPEYFSNMSNLEYLDLSNNKIQNIYHKDLQVLHQKPLLNLSLDLSLNPLDFIQPGAFKEVKLRELTLRSNFNSTDVMKASIQGLAGLQIHQLVLGEFKNERNLGRFDKSILEGLCNLIIEKFRIAYFDKFSEDAIDSFNCLANVSTISLVHLYFKGLKQLPKNLGWQRLELVNCEFEQFPTWKLDPLKELVFSANEVRNAFTQVKLESLEFLDLSRNDFSLKSCCSERDLGTTRLKHLDLSFNNIITISSNFLGLEQLEYLDFQHSSLKQVSDFSVFLPLKNLRYLDISYTHTQVAFHGIFNGLISLQILKMAGNSFQDNFLPNIFMELTNLTILDLSDCQLEQVSQVAFNSLPKLQLLNMSHNHLLSLDTLPYEPLHSLQTLDCSFNRIVASKEQELRHFPSNLSSLNLTRNDFACVCEHQSFLQWVKDQRQLLVEVEQMVCAKPLDMQGMPMLNFRNATCQVRKTIITGSVFTVLLVFLVVVLVYKFYFHLMLLAGCKKYSRGESTYDAFVIYSSQDEDWVRNELVKNLEEGVPPFQLCLHYRDFIPGVAIAANIIQEGFHKSRKVIVVVSQHFIQSRWCIFEYGIAQTWQFLSSRAGIIFIVLQKLEKSLLRQQVELYRLLNRNTYLEWEDSVLGRHIFWRRLRKALLDGKPRCPEGMADAEGS

>*Felis catus* Toll-like receptor 6 XP_003985516.1

MTKDKESITRSFCFVYILTLIVGTIIQFSDESEFAVDMSKMKLTHVPKDLSPKTKVLDISQNYISKLHISDISYLLGLEVLTLSYNRLQCLDFSIFKFNQDLEYLDLSHNQLQKTSCHLIRSLKHLDLSFNDFDVLPICKEFGNLTQLNFLGLSGTRLRQLDLLPIAHLHLSHILLDLEGYYAKESETESLQILNTKTLHLVFHPNQLFSVQVNISVNSLRCLQLTNIKLNNDNCQILIKFLSELIRGPTLLNFTLKHVETTWKCLVRVFQFLWPKPVEYLNIYNLIIVESIDEEDFTYSKTALKALKIEHVTNRVFIYSQTVLYTFFSEMNIMMLTLSDTPFIHMLCPQTSSTFKFLNFTQNVFTDSVFQNCSKLVRLETLILQKNKLKGLYNIGLMTKHMTSLEILDVSWNSLEYDRHDGNCTWGGSIVVLNLSSNILTDSVFRCLPPKVKVLDLYDNRIRSIPKLIMKLEALQELNVASNSLAHLPDCGAFSSLSVLIIDHNSISNPSADFFHSCQKIRSIRAGNNPFQCTCELREFIQNIGQVSSEVVEGWPDSYKCDNPESYKGTPLKDFHVSQLSCNTALLLVTIGVTVLVLTVTVTALCMYFDLPWYLRMVCQWTQTRHRARNLPLEELQRTLQFHAFISYSEHDSAWVKNELVPYLEKEDLRICLHERNFVPGKSIVENIINCIEKSYKSIFVLSPNFVQSEWCHYELYFAHHNLFHEGSNNLILILLEPIPQNCIPSKYHKLKALMTQRTYLEWPKEKSKHGLFWANIRAAFNMKLTLIAENNNAETYKKSGNSI

>*Felis catus* Toll-like receptor 7 NP_001073602.1

MVFPMWALKRQSLILFNIILISKLLGARWFPKTLPCDVTLDAPKAHVIVDCTDKHLTEIPEGIPTNATNLTLTINHIPGISPASFHQLDYLVEIDFRCNCIPIRLGPKDNMCPRRLQIKPRSFSRLTYLKSLYLDGNQLLEIPEGLPPNLQLLSLEANSIFCIMKNNLTELTNIEKLYLGQNCYFRNPCNVSFFIEKDAFLSLKNLKLLSLKDNNITYVPTTLPSTLTELHLYNNAIAKIQEDDFHNLNQLQILDLGGNCPRCYNVPFPCTPCENNSPLQIHMKAFDALTELQVLRLYSNSLQHVPQRWFKNIKKLKELDLSQNFLAKEIGDAKFLLLLHNLVQLDLSFNYELQVYRATLNLSDAFSSLKNLKVLRIKGYVFKELSSHNLSPLRSLSNLEVLDLGTNFIKIADLSIFEQFKTLKVIDLSMNKISPSGDSSEVGFCSNTRTSVDGNAPQVLETLHYFRYDEYARSCRFKNKETPSFLPFNKDCYVYGQALDLSRNNIFFVKSSDFQHLSFLKCLNLSGNTIGQTLNGSEFQPLVELKYLDFFNNRLDLLYSTAFEELRNLEILDISSNSHYFQSEGITHMLNFTKNLKVLKKLMMNNNDISMSTSRTMESESLRILEFRGNHLDVLWRDGDNRYLKFFKNLLNLEELDISENSLSFLPSGVFDGMPPKLKTLSLVKNGLKSFNWGRLQYLKNLETLDLSYNELKSVPERLYNCSRSLKKLILKYNQIRHLTKHFLQDAFQLRYLDLSSNKIQIIQKTSFPENVLNNLEMLLLHHNRFLCTCDAVWFVWWVNHTEVTIPYLATDVTCVGPGAHRGQSVVSLDLYTCEVDLTNLILFSLSVSVALSLMVITTANHLYFWDVWYSYHFCKAKIKGYQRLTSLDSCYDAFVVYDTKDPAVTEWVLDELVAKLEDPREKHFNLCLEERDWLPGQPVLENLSQSIQLSKKTVFVMTNKYAKTENFKIAFYLSHQRLMDEKVDVIILIFLEKPLQKSKFLQLRKRLCKSSVLEWPTNPQAHPYFWQCLKNALATDNHVTYSQVFKETV

>*Felis catus* Toll-like receptor 8 XP_019679113.1

MTLQSLLLTCLFLLISDSREFFTEANYSRSYPCDEKRQNGSIIAQCNDRRLREVPQTVSKYVTALDLSYNFITHITNESFQGLQNLTRINLNHNANQQHLNENPDINRSGMNITDGAFLNLQNLNQLLLEDNQLDQIPAGLPGSLRELSLIQNNIIWVAKKNTSGLTNLQRLYLSWNCYFGNNCNNKTFDIEDGTFESLTNLQVLSLSFNKLVHVPPKLPNSLTELYLSNAKIKVISQEDFKGLTNLRVLDLSGNCPRCFNAPFPCTPCEGGSSIQIHPLAFQTLTELRYLNLSSTSLQKIPAMWFQSMHNLKVLHLEFNYLVDEIASGEFLTKLPSLEILDLSYNYVKAKYPRYINISQNFSHLKLLQTLHLRGYVFQELRAEDFQPLMNLSNLKTINLGINFIKQINFTLFQNFSNLSIIYLSENRISPLVNDIKQNDMSGSSFQSHIRRLRSADTEFDPHSNFYHNTNPLIKPQCTVYGKALDLSLNSIFFIGREQFKAFHDIACLNLSSNGNGQVLHGTEFSAVPHIKYLDLTNNRLDFDDDNALSDLPELEVLDLSYNAHYFRIAGVTHRLGFIQNLTQLKVLNLSHNSIYTLTEQDLRSMSLKELVFSGNRLDILWNAEGDKYWKIFTNLGNLTWLDLSSNNLRHIPNEAFLNLPQSLTKLYIRDNVLNSFNWTLLQLFPHLQLLDLSGNRLSSLTNSLSKFTPSLRTLLLRRNRISHLPSNFLSEASSLIHLDLSSNLLKMINKSTLQTKTATNLTILELGRNPFDCTCDIGDFRRWMDENLDVTIPRLADVICSSPGDQRGKSIVSLELTTCVSDTIAAILCFFTFFITITVMLAALGHHWFYWDVWFIYHVCLAKIKGYRSLSTSQTFYDAYVSYDTKDASVTDWVINELRFHLEESEEKNVLLCLEERDWDPGLAIIDNLIQSINQSKKTIFVLTKKYAKNWNFKTAFYLALQRLMDENMDVIIFILLEPVLQNSQYLRLRQRICKSSILQWPDNPKAEGLFWQSLKNVVLTENDSRYNNLYVDSIKQY

>*Felis catus* Toll-like receptor 9 AAW50952.1

MGPCHGALHPLSLLVQAAALAVALAQGTLPAFLPCELQRHGLVNCDWLFLKSVPHFSAAAPRGNVTSLSLYSNRIHHLHDSDFVHLSSLRRLNLKWNCPPASLSPMHFPCHMTIEPHTFLAVPTLEELNLSYNSITTVPALPSSLVSLSLSRTNILVLDPANLAGLHSLRFLFLDGNCYYKNPCPQALQVAPGALLGLGNLTHLSLKYNNLTAVPRGLPPSLEYLLLSYNHIITLAPEDLANLTALRVLDVGGNCRRCDHARNPCMECPKGFPHLHPDTFSHLNHLEGLVLKDSSLYNLNPRWFHALGNLMVLDLSENFLYDCITKTTAFQGLAQLRRLNLSFNYHKKVSFAHLHLAPSFGSLLSLQQLDMHGIFFRSLSETTLRSLVHLPMLQSLHLQMNFINQAQLSIFGAFPGLRYVDLSDNRISGAMELAAATGEVDGGERVRLPSGDLALGPPGTPSSEGFMPGCKTLNFTLDLSRNNLVTIQPEMFARLSRLQCLLLSRNSISQAVNGSQFMPLTSLQVLDLSHNKLDLYHGRSFTELPRLEALDLSYNSQPFSMQGVGHNLSFVAQLPALRYLSLAHNDIHSRVSQQLCSASLRALDFSGNALSRMWAEGDLYLHFFRGLRSLVRLDLSQNRLHTLLPRTLDNLPKSLRLLRLRDNYLAFFNWSSLVLLPRLEALDLAGNQLKALSNGSLPNGTQLQRLDLSSNSISFVASSFFALATRLRELNLSANALKTVEPSWFGSLAGTLKVLDVTGNPLHCACGAAFVDFLLEVQAAVPGLPGHVKCGSPGQLQGRSIFAQDLRLCLDEALSWDCFGLSLLTVALGLAVPMLHHLCGWDLWYCFHLCLAWLPRRGRRRGADALPYDAFVVFDKAQSAVADWVYNELRVRLEERRGRRALRLCLEERDWLPGKTLFENLWASVYSSRKMLFVLAHTDRVSGLLRASFLLAQQRLLEDRKDVVVLVILRPDAHRSRYVRLRQRLCRQSVLLWPHQPSGQRSFWAQLGTALTRDNQHFYNQNFCRGPTTAE

>*Equus caballus* Toll-like receptor 2 AAR08196.1

MPHALWTVWVLGAVISLSKEGVPDQPSSLSCDPTGVCDGRSRSLNSIPSGLTAAVKSLDLSNNKIASVGNSDLWKCVNLKALRLGSNDINTIEEDSFSSLRSLEHLDLSNNHLSNLSSSWFRPLSSLKFLNLLGSTYKTLGETSLFSHLTNLRILKVGNIHFTEIQGKDFAGLTFLEELEIDATNLQRYEPKSFKSIQNISHLILRMKQPVLLPEIILDTLSSLEYLELRDTYLNTFHFAEVSDPETNTLIKKFTFRNVKITDESFDEIVKLLNYISGVSEAEFDECTLDGLGEFRTPDIDKIKVIGKLETLTIRRLRIPQFYLFRDLSSIYSLTERVKRITIENSKVFLVPCSLSRHLKSLEYLDLSDNLMVEEYLKNSACERAWPSLQTLILRQNHLTSLGKTGETLLTLKNLTKLDISKNSFHSMPETCQWPEKMKYLNLSSIRIDRLTQCIPQTLEVLDISNNNLNSFSLILPQVKELYISRNKLKTLPDASFLPMLLVMRISRKTINTFSKEQLDSFQKLKTLEAGGNNFICSCEFLSFTQEEQALDQILIDWPENYLCDSPSHVRGQRVQDTHLSVSECHRTALVSAVCCALFLSILLTGVLCHHFHGLWYMKMMWAWLQAKRKPRTAPQRDICYDAFVSYSERDSYWVENLMVQELEHFNPPFKLCLHKRDFIPGKWIIDNIIDSIEKSHKTIFVLSENFVKSEWCKYELDFSHFRLFDENNDAAILILLEPIDKKAIPQRFCKLRKIMNTKTYLEWPTDEAQQEGFWLNLRAAIKS

*>Equus caballus* Toll-like receptor 3 ABB92546.1

MSQSLPYHVYSFLRLLPFWVLCASCNKCAVRHEVADCSHLKLTQIPDDLPANITVLNLTHNQFRRLPPDNFTRYSQLTILDGGFNCISKLEPELCQKLPLLEILDLQHNELSQFSDKTFMFCMNLTELHLRSNSIQKIQNNPFKNLKNLIKLDLSHNGLSSTKLGTQLQLENLQELLLSNNKIQALRRDELDFLGNSSLKKLELSSNQIKEFSPGCFQAIGKLFGLSLNSVQLGPSLTEKLCLELSNTSIQNLSLSNTQLYRTSNTTFFGLKQTNLTMLDLSHNNLXVIGNDSFAWLPHLEYFFLEYNNIEHLYVHSFYGLFNVRYLNLRRSFTKQSISLASLPKIDDFSFQWLKCLEYLNMEDNNFPGIKSNMFTGLVKLKHLSLSNSFSSLRTLTNETFLSLAHSPLLTLNLTKNKISKLESGAFSWLGHLKILDLGLNEIGQELTGQEWRGLENIFEIYLSYNKYLQLTSNSFALVPSLQRLMLRRVALKNVDSFPPPFRPLHNLTILDLSNNNIANINDELLEGLEKLEVLDLQHNNLARLWKHANPGGPVHFLKGLSHLHVLNLESNGFDEIPAEAFKNLFELKSINLGLNNLNILPPSVFDDQVSLKSLSLQKNLITSVEKNVFGPALKNLSSLDMSFNPFDCTCESIAWFVNWINGTHTNISELSSHYLCNTPPQYHGFPVMLFDTSSCKDSAPFELLFMINTSFLLIFIFIVLLIHFEGWRISFYWNVSVHRVLGFKEIDSQPEQFEYAAYIIHAYKDRDWVWEHFSPMEEQDQTLKFCLEERDFQAGVLELEAIINSIKRSRKIIFIITQHLLKDPLCKRFKVHHAVQQAIEQNLDSIILIFLEEIPDYKLNHALCLRRGMFKSRCILNWPVQKERINAFHHKLQVALGSRNSAH

>*Equus caballus* Toll-like receptor 4 AAF91076.1

MMPPTRLAGTLIPAMAFLSCLRPESWDPCVQVVPNTTYQCMDLNLYKIPENIPTSTKELDLSFNPLKELGSHSFSNFPELQVLDLSRCEIEMIEDDAYQGLNHLSTLILTGNPIRSLALGAFSGLSSLQTLVAVETKLSSLEKFPIGHLKTLKELNVAHNLIHSFKLPEYFSKMPNLEHLDLSNNKIQNISHEDLRVLHQMPLLNLSLDLSLNPLEFIQPDAFKEIKLHKLTLRSNFDSIDVMKSCIQGLAGLKVNRLVLGEFKNERKLERFDTSALRGLHNLTIEEFRLAYIDNYSSKDSIDLLNCLADISKISLVSLDLGNLKDFPKGFGWQDFELVNCRIEGFPTLELTSLKRLVFTSNKDMKSFNEVKLPSLEFLDLSRNRLSFKSCCSEADLKTTRLKHLDLSFNDVISMSSNFMGLEQLEHLDFQHSTLKQASDFPVFLSLKNLRYLDISYTNTRVVFHGIFDGLVSLQVLKMAGNSFKDNFLPNIFREMTNLTTLDLSKCNLEQVSQEAFCLLPRLRVLNMSHNNLLFLDMLPYKPLHSLQILDCSFNRIVAFKWQELQHFPSSLASLNLTQNDFACVCEYQSFLQWVKDQRQLLVEVEHLVCAIPLQMRGMPVLGFNNATCQISKTIVGGSVFSILMVSVIAVLVYKFYFHLMLLAGCKKYGRGESIYDAFVIYSSQDEDWVRNELVKNLEEGVPPFQLCLHYRDFIPGVAIAANIIQEGFHKSRKVIVVVSQHFIQSRWCIFEYEIAQTWQFLSSRAGIIFIVLHKLEKSLLRQQVELYRLLNRNTYLEWEDSVLGRHIFWRRLRKALLDGKPWSPAGTADAAESRQHDAETST

>*Equus caballus* Toll-like receptor 7 ABC69201.1

MMFPMWTLKREFLILFNMILISKLLGARWFPKTLPCDVFLDAPKAQVIVDCTDKHLTDIPGGIPTNATNLTLTINHIPGISPASFHQLENLLEIDFRCNCVPVRLGPKDNVCKNRLQIKPRSFSRLTNLKSLYLDGNQLLEIPQDLPPSLQLLSLEANNIFLIMKKNLTELANIEMLYLGQNCYYRNPCNVSFFIEKDAFLNLKNLKLLSLKDNNITAVPTILPSSLTELYLYNNIIAKIQEDDFKMLNQLQILDLSGNCPRCYNVPYPCTPCENNSPLQIHANAFDALTELQVLRLHSNSLQYVPQRWFKNINKLKELDLSQNFLAKEIGDAKFLHLLHNLVQLDLSFNYELQVYHASMNLSEAFSSWKNLRVLRIKGYVFKELKDLNLSPLRNLSNLEVLDLGTNFIKIADLSIFKQFKTLKVIDLSMNKISPSGESSEVGFCSNTRTSVAGHGPQVLETLHYFRYDEYARSCWFKNRETSSFLPFNEGCYMYGQTLDLSKNNIFFIKSSDFRHLSFLKCLNLSGNSISQTLNGSEFQPLVELKYLDFSNNRLDLLYSTAFEELRNLEVLDISSNSHYFQSEGITHMLNFTKNPKVLRKLMMNNNDISTSTSRTMESESLTILEFRGNHLDVLWKDGDNRYLKFFKNLLNLKELDISENSLSFLPPGVFESMPPNLKTLYLVNNKFKSFNWGKLQLLKNLETLDLSYNQLRTVPERLSNCSRSLKKLILKNNQIRRLTKYFLQDAFQLRYLDLSSNKIQIIQKSSFPENVLNNLDMLLLHRNRFLCTCDAVWFVWWVNHTEVTIPYLATDVTCTGPGAHKGQSVVSLDLYTCELDLTNLILFSLSMSMALFLMVVTTANHLYFWDVWYSYHFCKAKIKGYQRLTLTDSCYDAFIVYDTKDPAVTEWVLDELVAKLEDPREKHFNLCLEERDWLPGQPVLENLSQSIQLSKKTVFVMTDKYAKTENFKIAFYLSHQRLIDEKVDVIILIFLEKPLQKSKFLQLRKRLCGSSVLEWPRNPQAHPYFWQCLKNALATDNHVTYSQVFKETV

>*Equus caballus* Toll-like receptor 8 ABM87942.1

MILQPLLLTCLFLIISDSCEFFTETNYSRSYPCDEKRENVSVIAECNDRRLEEVPQTVGKYVTELDLSDNFIIHITNESFQGLQNLTKINLNHNGKARSQNENPDVNKNGMNITDGAFLNLQNLRELLLEDNQLDKIPTGLPGSLRELSLIQNRITSVTKKNTSGLMNLEYLYLGWNCYFGNICNKTFDIEDGTFERLTNLKVLSLSFNNLSHVPPKLPNSLRELYLSNTKIKNITQEDFKELRNLTVLDLSGNCPRCFNAPFPCTPCERDSSIQIHPLAFQDLTELRYLNLSSTSLRKVPAIWFDNMHHLKVLHLEFNYLVQEIASGEFLTKLPSLEILDLSFNYIVTKYPKYIEISPNFSNLTSLQILHLRGYVFQEVRKEHFRPLMSLSNLKTINLGVNFIKQIDFTFFQHFPNLTVIYLSENRISPLVNDSLQNYTNGSAFQSHILKRRSADFEFNPHSNFYHNTNPLIKPQCTAYGKALDLSLNSIFFIGQKQFKAFHDIACLNLSSNGIGQPLHGTEFSAVPHIKYLDLTNNRIDFDDDNALRELPELEVLDFSYNAHYFRIAGVTHRLGFIQNLTQLRVLNLSHNSIYTLTEYNMNSMSLEELVFSGNRLDLLWNAEDRRYRKIFKCLRNLTRLDLSFNNLQHIPDEAFLNLPQNLTELYINDNRLHFFNWTLLQHFPHLHLLDLSRNKLSSLTNNLSKFSPSLRTLLLSQNKISHLPSGFFSEASGLIHLDLRFNRLKMINKTTLQTKTTINLAVLELGGNPFDCTCDIGDFRRWMDENLNIAIPRLADVICASPGDQRGKSIVSLELTTCVSDAIAAILCFFTFFITVTVMLAALAHHWFYWDVWFIYHMCLAKIKGYRSLSTSQTFYDAYVSYDTKDASVTDWVINELRFHLEESEEKNVLLCLEERDWDPGLAIIDNLMQSINQSKKTIFVLTKKYAKNWNFKTAFYLALQRLMDENMDVIVFILLEPVLQHSQYLRLRQRICKSSILQWPDNPKAEGLFWQSLKNVVLTENNSRYNNLYVDSIKQY

>*Equus caballus* Toll-like receptor 9 ABD36388.2

MGPCHGALQPLSLLVQAAMLAVALAQGTLPPFLPCELQPHGLVNCNWLFLKSVPHFSAAAPRDNVTSLSLLSNRIHHLHDSDFAQLSNLQKLNLKWNCPPAGLSPMHFPCHMTIEPNTFLAVPTLEELNLSYNGITTVPALPSSLVSLILSRTNILQLDPTSLTGLHALRFLYMDGNCYYKNPCGRALEVAPGALLGLGNLTHLSLKYNNLTTVPRSLPPSLEYLLLSYNHIVTLAPEDLANLTALRVLDVGGNCRRCDHARNPCVECPHKFPQLHSDTFSHLSRLEGLVLKDSSLYQLNPRWFRGLGNLTVLDLSENFLYDCITKTKAFQGLAQLRRLNLSFNYHKKVSFAHLTLAPSFGSLLSLQELDMHGIFFRSLSQKTLQPLARLPMLQRLYLQMNFINQAQLGIFKDFPGLRYIDLSDNRISGAVEPVATTGEVDGGKKVWLTSRDLTPGPLDTPSSEDFMPSCKNLSFTLDLSRNNLVTVQPEMFAQLSRLQCLRLSHNSISQAVNGSQFVPLTSLQVLDLSHNKLDLYHGRSFTELPRLEALDLSYNSQPFSMRGVGHNLSFVAQLPTLRYLSLAHNGIHSRVSQQLCSTSLWALDFSGNSLSQMWAEGDLYLRFFQGLRSLIRLDLSQNRLHTLLPCTLGNLPKSLQLLRLRNNYLAFFNWSSLTLLPNLETLDLAGNQLKALSNGSLPSGTQLQRLDVSRNSIIFVVPGFFALATRLRELNLSANALRTVEPSWFGFLAGSLEVLDVSANPLHCACGAAFVDFLLQVQAAVPGLPSRVKCGSPGQLQGRSIFAQDLRLCLDESLSWDCFGLSLLVVALGLAMPMLHHLCGWDLWYCFHLGLAWLPRRGWQRGADALSYDAFVVFDKAQSAVADWVYNELRVRLEERRGRRALRLCLEERDWLPGKTLFENLWASVYSSRKMLFVLAHTDQVSGLLRASFLLAQQRLLEDRKDVVVLVILSPDARRSRYVRLRQRLCRQSVLFWPHQPSGQRSFWAQLGMALTRDNRHFYNQNFCRGPTMAE

>*Canis lupus familiaris* Toll-like receptor 1 ACB41373.1

MKTNPSIFQFAIIFILILEIRIQLSEESDFLVNRSKAGLFHIPKDLSLKTTILDISQNYISELQTSDILSLSKLRILIVSYNRIQYLDISVFKFNQELEYLDLSHNELGRISCHPTVNLKHLDLSFNAFDDLPICKEFGNMSQLEFLGLSATQLQKSSMLPIASLHIRKVLLVLGDTYGKKEDPESLQKLNTESLHIVFPIRKEFSFTLDVSVSTAVSLELSNIKCVPDGHGWSYFQNVLSKLQKNSRLSSLTLNNIETTWNFFIMLLQLVWHTSIEYFSISNVKLQGYPDFRDFDYSDTSLKALSIHQVVSNAFNLPQSYIYKIFSNMNIQNFTVSGTHMVHMVCPSQISPFLHLDFSNNLLTDIVFKNCRNLIKLETLSLQMNQLKELASIAQMTNEMKSLQQLDISQNSLRYDENEGNCSWTRSLLSLNMSSNILTDSVFRCLPPKVKVLDLHDNRIRSIPKPIMKLEDLQELNVASNSLAHFPDCGTFNRLSVLIIDSNSISNPSADFLQSCHNIRSISAGNNPFQCTCELREFVQSLGQVASKVVEGWPDSYKCDSPENYKGTLLKDFHVSPLSCNTTLLLVTIGVAVLVFTVTVTALCIYFDLPWYLRMVFQWTQTRRRARNTPLENLQRTIQFHAFISYSGHDSAWVKSELLPNLEKEELRICLHERNFIPGKSIVENIINCIEKSYKSIFVLSPNFVQSEWCHYELYFAHHNLFHEGSNNLILILLEPIPQYSIPSSYHKLKNLMAQRTYLEWPKEKSKHGLFWANLRASINIKLREQAKK

>*Canis lupus familiaris* Toll-like receptor 2 BAD42423.1

MSRVLWTLWVLGAVTNLSKEEAPDQSSSLSCDPTGVCDGRSRSLNSMPSGLTAAVRSLDLSNNEITYIGNSDLRDCVNLKALRLESNGINTIEEESFLSLWSLEHLDLSYNLLSNLSSSWFRPLSSLKFLNLLGNPYKSLGETPLFSQLTNLRILKVGNIYSFTEIQDKDFAGLTFLEELEIDASNLQRYEPKSLKSIQNISYLALRMKQPVLLVEIFVDLSSSLKHLELRDTHLDTFHFSEASINETHTLVKKWTFRNVKVTDRSFTGVVRLLNYVSGVLEVEFEDCTLYGLGDFDIPDVDKIKNIGQIETLTVRRLHIPHFYSFYDMSSIYSLTEDVKRITVENSKVFLVPCLLSQHLKSLEYLDLSENLMVEEYLKNSACEDAWPSLQTLVLRQNHLASLERTGETLLTLKNLTNIDISKNSFHSMPETCQWPEKMKYLNLSSTRIHSVTGCIPKTLEILDVSNNNLNLFSLNLPQLKELYISRNKLMTLPDASLLPMLLVLKISRNAITTFSKEQLDSFHTLKTLEAGGNNFICSCEFLSFTQEQQALAKVLIDWPANYLCDSPSHVRGQQVQDVRLSVSECHRTALVSGMCCALFLLILLTEVLCHRFHGLWYMRMMWAWLQAKRKPRKAPSRDVCYDAFVSYSEHDSYWVENLLVQKLEHFNPPFKLCLHKRDFIPGKWIIDNIIDSIEKSRKTIFVLSENFVKSEWCKYELDFSHFRLFDENSDAAILILLEPIEKKAIPQRFCKLRKIMNTKTYLEWPTDDAQQEGFWLNLRTAIKS

>*Canis lupus familiaris* Toll-like receptor 4 protein BAB85609.1

MPLLNLSLDLSLNPLYFIQPGSFKEIKLHKLTLRSNFNSTDVMKTFIQSLAGLKINQLVLGEFKNERKLESFDNSLLEGLCNLTIEKFRIAYFDSFSKDTTNLFNQLVNISAISLAHLYLDTPKYLPKNLRWQRLEIVNCNLEQFPAWELDSLKEFVLTSNKGMNTFADMKMESLEFLDLSRNRLSFKTCCSHSDFGTTRLKHLDLSFNEIITMSSNFLGLEQLEYLDLQHSSLKQASDFSVFLSLRNLRYLDISYTRTEVAFQGIFDGLVSLEVLKMADNSFPDNSLPNIYKGLTNLTILDLSRCHLERVSQESFVSLPKLQVINMSHNSLLSLDTLAYEPLLSLQILDCSFNRIVAFKEQGQQHFPSNLVSLNLTRNSFACDCEHQSFLQWVKDHRQLLVKVEQMVCAKPLDMKDMPLLSFRNATLSEEARLSISVSVFTVLHGFSGSSFSRYKFYFHLMLLAWLAKGITEGKVPMMHFVIYSSQDEDWVRNELVKNLEEGVPPFQLCLHYRDFIPGVAIAANIIQEGFYKSRKVIVVVSQHFIQSRWCIFEYEIAQTWQFLSSRAGIIFIVLQKVEKSLLRQQVELYRLLSRNTYLEWEDSVLGRHIFWRRLRKALLDGKPWSPEGTEDAEKS

>*Canis lupus familiaris* Toll-like receptor 7 protein BAE79271.1

MVFPMWTLKRQFFILLNIILISKLLGARWFPKTLPCDVSLDAPKAHVIVDCTDKHLTEIPGGIPSNATNLTLTINHIPGISPASFHQLDYLVEIDFRCNCIPVRLGPKDHLCTRRPQIKPRSFSSLTYLKSLYLDGNQLLEIPEGLPPSLELLSLEANSIFSIMKYNLTELTNIERLYLGQNCYFRNPCNVSFFIEKDAFLSLKNLKLLSLKDNNITYVPTTLPSTLTELYLYNNAIAKIQEDDFNNLNQLRILDLSGNCPRCYNVPFPCTPCENNSPLQIHESAFDALTELQVLRLHSNSLQRVPQRWFKNIKKLKELDLSQNFLAKEIGDAKFLYLLHDLVQLDLSFNYELQVYRAALNLSDAFSSLKNLKVLRIKGYVFKELSSHHLSPLQSLTNLEVLDLGTNFIKIADLSIFEQFKTLKVIDLSMNKISPSGDSGEVGFCSSTRTSVEGHAPQVLETLHYFRYDEYARSCRFKNKETPSFLPFNKDCYMYGQTLDLSRNNIFFIKSSDFQHPSFLKCLNLSGNTIGQTLNGSEFQPLVELKYLDFSNNRLDLLYSTAFEELRKLEVLDISSNSHYFQSEGITHMLNFTKNLKVLKKLMMNNNDIATSTSRTMESESLKILEFRGNHLDVLWRDGDNRYLKFFKNLLNLEELDISENSLSFLPSGVFDGMPPNLKTLSLVKNGLKSFHWERLQYLKNLETLDLSYNELKIVPERLYNCSRSLKKLILKYNQIRQLTKHFLQDAFQLRYLDLSSNKIQIIQKTSFPENVLNNLEMLLLHHNRFLCTCDAVWFVWWVNHTEVTIPYLATDVTCVGPGAHKGQSVVSLDLYTCELDLTNLVLFSFSLSLALFLMVITTANHLYFWDVWYSYHYCKAKIKGYRRLKSLDSCYDAFVVYDTKDPAVTEWVLDELVAKLEDPREKHFNLCLEERDWLPGQPVLENLSQSIQLSKKTVFVMTNKYAKTENFKIAFYLSHQRLMDEKVDVIILIFLEKPLQKSKFLQLRKRLCKSSVLEWPRNPQAHPYFWQCLKNALATDNHVTYSQVFKETV

>*Canis lupus familiaris* Toll-like receptor 9 AAW50951.1

MGPCRGALHPLSLLVQAAALALALAQGTLPAFLPCELQPHGLVNCNWLFLKSVPRFSAAAPRGNVTSLSLYSNRIHHLHDYDFVHFVHLRRLNLKWNCPPASLSPMHFPCHMTIEPNTFLAVPTLEDLNLSYNSITTVPALPSSLVSLSLSRTNILVLDPATLAGLYALRFLFLDGNCYYKNPCQQALQVAPGALLGLGNLTHLSLKYNNLTVVPRGLPPSLEYLLLSYNHIITLAPEDLANLTALRVLDVGGNCRRCDHARNPCRECPKGFPQLHPNTFGHLSHLEGLVLRDSSLYSLDPRWFHGLGNLMVLDLSENFLYDCITKTKAFYGLARLRRLNLSFNYHKKVSFAHLHLASSFGSLLSLQELDIHGIFFRSLSKTTLQSLAHLPMLQRLHLQLNFISQAQLSIFGAFPGLRYVDLSDNRISGAAEPAAATGEVEADCGERVWPQSRDLALGPLGTPGSEAFMPSCRTLNFTLDLSRNNLVTVQPEMFVRLARLQCLGLSHNSISQAVNGSQFVPLSNLRVLDLSHNKLDLYHGRSFTELPRLEALDLSYNSQPFSMRGVGHNLSFVAQLPALRYLSLAHNGIHSRVSQQLRSASLRALDFSGNTLSQMWAEGDLYLRFFQGLRSLVQLDLSQNRLHTLLPRNLDNLPKSLRLLRLRDNYLAFFNWSSLALLPKLEALDLAGNQLKALSNGSLPNGTQLQRLDLSGNSIGFVVPSFFALAVRLRELNLSANALKTVEPSWFGSLAGALKVLDVTANPLHCACGATFVDFLLEVQAAVPGLPSRVKCGSPGQLQGRSIFAQDLRLCLDEALSWVCFSLSLLAVALSLAVPMLHQLCGWDLWYCFHLCLAWLPRRGRRRGVDALAYDAFVVFDKAQSSVADWVYNELRVQLEERRGRRALRLCLEERDWVPGKTLFENLWASVYSSRKTLFVLARTDRVSGLLRASFLLAQQRLLEDRKDVVVLVILCPDAHRSRYVRLRQRLCRQSVLLWPHQPSGQRSFWAQLGTALTRDNRHFYNQNFCRGPTTA

>*Sus scrofa* Toll-like receptor 1 BAG12310.1

MTKENLSIFHFAIIFILILEIRIQLSEESEVLVDRSKTGLTHVPKDLSLETTILDLSQNSISELQTSDILSLSKLRVFIISHNRIQYLDVSVFKFNQELEYLDLSHNKLEKISCHPMLNLKHLDHSFNAFDALPICQEFGSMFQLEFLGLSATQLQKSSVLPIAHLHIGKVLLVLGDSYGEREDPESLQDLNTQSLHIVYPPGKEFHFMLDVSVSTAVNLELSSIRCVLDANGCHHFQNVLLKLQKNSKLSNLTLNNIETTWNSFITTLQFVWHTSIEYFSISSVKLQGQLDFRDFDYSDTSLKALSLHQAVSEVFSFPQSYIYKIFSNMNIQYLTVSATHMVHMLCPSQISPFLYLDFSNNALTDMVFKNCANLANLNTLSLQMNRLKELVNVIHMTKEMQSLQQLDVSQNTLRYDENEGSCTWTGSLLSLNLSSNILTDSVFRCLPPRIKVLDLHNNRIRSIPKDVAHLEALQELNVASNSLAHLPGCGSFSSLSILIIDYNSISNPSADFFQSCQKIRSLKAGNNPFQCTCELRGFIQSLGQVSSDVAESWPDSYECEYPESYKGTLLKDFRVSELSCNTALLIVTIGVTGLALALTMTGLCVYFDLPWYLRMLCQWTQTRRRARNVPLEELQRTLQFHAFISYSGHDSAWVKNELLPNVEKEGIKICLHERNFVPGKSIMENIINCIEKSYKSIFVLSPNFVQSEWCHYELYFAHHNLFHEGSDNLILILLDSIPQYSIPSSYHKLKALMAQRTYLEWPKEKSKHGLFWANLRASINIKLMEKAEEISYTQI

>*Sus scrofa* Toll-like receptor 2 BAD91799.1

MPCALWTAWVLGIVISLSKEGAPHQASSLSCDPAGVCDGRSRSLSSIPSGLTAAVKSLDLSNNRIAYVGSSDLRKCVNLRALRLGANSIHTVEEDSFSSLGSLEHLDLSYNHLSNLSSSWFKSLSTLKFLNLLGNPYKTLGETPLFSHLPNLRILKIGNNDTFAEIQAKDFQGLTFLQELEIGASHLQRYAPKSLRSIQNISHLILHMRRPALLPKIFVDLLSSLEYLELRNTDFSTFNFSDVSINEPSTVMKKFTFRKAEITDASFTEIVKLLNYVSGALEVEFDDCTLNGRGDFSTSALDTIKSLGNVETLTVRRLHIPQFFLFYDLRSIYSLTGAVKRITIENSKVFLVPCSLSQHLKSLEYLDLSENLMSEEYLKNSACEHAWPFLHTLILRQNHLKSLEKTGEVLVTLKNLTNLDISKNNFDSMPETCQWPEKMKYLNLSSTRIHSLTHCLPQTLEVLDISNNNLNSFSLSLPQLKELYISRNKLKTLPDASFLPMLSVLRISRNTINTFSKEQLDSFQKLKTLEAGGNNFICSCDFLSFTQGQQALAQVLSDWPENYLCDSPSHVRGQRVQDTRLSLTECHRVAVVSVVCCALFLLLLLTGALCHHFHGLWYMKMMWAWLQAKRKPRKAPRRDVCYDAFVSYSEQDSYWVENLMVQELEHFQPPFKLCLHKRDFIPGKWIIDNIIDSIEKSQKTIFVLSENFVKSEWCKYELDFSHFRLFDENDDTAILILLEPIEKKAIPQRFCKLRKIMNTRTYLEWPADETQREGFWLNLRAAIKS

>*Sus scrofa* Toll-like receptor 3 AMH85923.1

MSRSLPCHIYSFWVLLPFWILYTTSTNKCTVRHEIADCSHLKLTQIPDDLPANITVLNLTHNQLRRLPPANFTIYSQLTTLDGGFNTISKLEPELCQSLPLLEILNLQHNELSQLSDKTFIFCMNLIELHLMSNSIQKIQNNPFKNLKNLIKLDLSHNGLSSTKLGTQLQLENLQELLLANNKISALKREELDFLGNSSLKRLELSSNQMQEFSPGCFHAIGKLFGLSLNNVKLSPSLTEKLCLELSNTSIENLSLSNIQLYKTSNTTFFGLKQTNLSMLDLSHNSLSVIGNDSFAWLPHLKYFFLEYNNIERLSSRSLYGLSNVKYLNLRRSFTKQSISLASLPKIEDFSFQWLKSLEYLNMEDNNFPGIKRNTFTGLIKLKSLSLSNSFSSLRTLTNETFISLADSPLLILNLTKNKISKIESGAFSWLGHLKVLDLGLNEIGQELTGQEWRGLKNIVEVYLSYNRYLELTTDSFALVPSLQQLMLRRVALRNMDCSPSPFHPLFNLTILDLSNNNIANINDELLKGLEKLQILDLQHNNLARLWKHANPGGPVQFLKGLSHLHILNLESNGFDEIPADAFRDLSELKSIDLGLNNLNILPPSVFDNQVSLKSLSLQKNLITSVKKTVFGPAFQKLSNLDMRFNPFDCTCESIAWFVSWINSTHTNISELSSHYLCNTPPQYHGLPVILFDTSPCKDSAPFELFFMITASMLLIFIFIILLIHFEGWRISFYWNVSVHRVLGFKEIDKQPEQFEYAAYIIHAYKDRDWVWEHFAPMEEKDETLRFCLEERDFEAGALELEAIVNSIKRSRKIIFVITQHLLKDPLCKRFKVHHAVQQAIEQNLDSIILIFLEEIPDYKLNHALCLRRGMFKSHCILNWPVQKERINAFHHKLQVALGSRNSVH

>*Sus scrofa* Toll-like receptor 4 AHA36587.1

MIPRIRLAVATIPAMAFLSCLRSESWDPCVQVVPNISYQCMELNFYKIPDNIPTSVKILDLSFNYLSHLDSNSFSSFPELQVLDLSRCEIQTIDDDAYQGLNYLSTLILTGNPIQSLALGAFSGLPSLQKLVAVETNLASLEDFPIGHLKTLKELNVAHNHIHSFKLPEYFSNLPNLEHLDLSKNKIENIYREHLQVLHQVPLLNLSLDLSLNPLNFIEPGAFNKIRLNGLTLRSNFNSSDVMKTCIQGLAGSKINQLVLGEFKNERNLESFDKSVLEELCNLTLEQFRIAHFGEFPDDVSDLFNCLANASVISLLGLNLRGLEALPNDFRWQHLEVVNCKLQQFPALKFNSLKKFVFKDNKHMHTFTEINLPNLQFLDLSGNHLSFKGCCSHNEFGTTKLKHLDLSFNEIITMKSNFMGLEQLEYLDFQHSSLKQANDFSIFLSLRNLHYLDISYTNIHVVFRGIFAGLVSLQTLKMAGNSFQNNLLPDVFTDLTNLILLDLSKCQLEQVSQRAFHSLPRLQVLNMSHNRLLFLDTLPYKPLHSLRILDCSYNLIVASKEQELQHLPRSLAFLNLTKNDFSCACEHQTFLQWVKDQKQLLVGAEQMVCTQPLEMQDLPVLSFRNATCQISEAVISASVLTFLLVSVAGILVYKFYFHLLLFVGCKKYGRGESTYDAFVIYSSQDEDWVRNELVKNLEEGVPPFHLCLHYRDFIPGVAIAANIIQEGFHKSRKVIVVVSQHFIQSRWCIFEYEIAQTWQFLRSHAGIIFIVLQKLEKSLLRQQVELYRLLSRNTYLEWEDSVLGRHIFWRRLKKALLDGKPWSPEGTEDSESNQHDTTAFT

>*Sus scrofa* Toll-like receptor 5 ACN71220.1

MGDCLVLLLTLLVASPALGMPSCFFDGQRAIYRGCNLTQVPQVPSGTKSLLLSFNYIRTVTAGSFPFLEGLQLLELGTQLTPLSIDREAFRNLPNLRILDLGHSQIAFLHPDAFQELPHLSELRLFSCGLSDAILKDGYFRNLASLTRLDLSKNQIQSLHLHPSFQELNSLKAIDLSLNQIPLVCEQGLKPLQGKTLSFLSLADNNLYSRVSVDWGKCMNPFRNMALEMLDVSGNGWTADTTRNFSQAVNGSQISSLVLAHHIMGSGFGFHNIKDPDHHTFASLGRSSLIQLDLSHGFIFSLNFRLFGTLKELKVLNLAFNKINKIADQAFHGLDNLQILNMSYNLLGELYNSNFEGLPKLAYIDLQKNHIGIIQDQTFRFLKKLNTLDLRDNALKTIQFIPSIPTLFLGGNKLVTLPNIRLTANFIHLSENRLENLDNLYFLLQVPHLQILILNQNRFSNCNQRHAPSENPSLEQLFLGENMLQLAWEAGFCWDVFKGLSHLQVLYLNNNYLNFLPPGVFRHLTALRGLSLSYNRLTVLYPGDLPAHLEVLDLSRNQLLSPDPDLFTSLSAVDLSHNKFVCDCELSTFINWLNQTNVTIFGSRDDIYCMYPSSLAGSPLNTVSMAGCSEEEVLESLKFSLFILFTVTLTLLFVAILVVMKFRGFCFICYKKVQRLVFKDPSLGRESDTYKYDAYLCFSGKDFEWVQEALLKNLDAQYSDQNRLNLCFEERDFVPGEDRISNIQDAVWSSRKVVCLVSRHFLRDGWCLEAFSYAQSRCLADLNGTLIMVVVGSLPQYQLMKHQSIRGFVRKQQYLRWPEDLQDVSWFLNKLSQYILKKVKEKKKDSDIQLQSVTIS

>*Sus scrofa* Toll-like receptor 6 AGT79979.1

MSKDKEPTVISLHSVYVMTLVWGTLIQFSEESEFVVDKSKIGLTRVPKDLPPQTKVLDVSQNFITELHPSDISFLSQLTVLRLSQNRMQCLDISVFKFNQDLEYLDLSHNQLQTILCHPITSLKHLDLSFNDFEALPICKEFGNLTQLNFLGLSATKLQQLDLLPIAHLHLSCILLDLERYYMKENEKESLQILNTKKLHLVFHPNSFFSVQVNISVKSVGCLQLANIKLSDDNCQVFITFLLELTQGPTLLNFTLNHVETTWKCLVGIFQFLWPKPVEYLSIYNLTIVESIDEEDFIYYETTLKGVKIEHITKRVFIFSQTALYRVFSDMNIRMLTIADTHFIHMLCPQVPSTFNFLNFTQNVFTDSVFQNCKTLARLETLILQKNKLEDLFKISLMTKDMLSLEILDVSSNSLEYDRHGENCTWVGSIVVLNLSSNILTDSVFRCLPPRIKVLDLHSNRIRSIPKDVAHLEALQELNVASNSLAHLPGCGSFSSLSILTIDYNSISNPSADFFQSCQKIRSLKAGNNPFQCTCELRDFIQSLGQVSSDVVESWPDSYECEYPESYKGTLLKDFRVSELSCNTALLIVTIGVTGLALALTMTGLCVYFDLPWYLRMLCQWTQTRRRARNVPLEELQRTLQFHAFISYSEHDSAWVKNELVPCLEKEGIKICLHERNFVPGKSIMENIINCIEKSYKSIFVLSPNFVQSEWCHYELYFAHHNLFHEGSDNLILILLDPIPQNSIPGKYHKLKALMAQRTYLEWPKEKSKHGLFWANIRAAFNIKLKLVAEEDDVKT

>*Sus scrofa* Toll-like receptor 7 AMH85922.1

MVFPVWTLKRQFLILFNIVLISELLGARWFPKTLPCDVSLDAPNAHVIVDCTDKHLTAIPGGIPTNATNLTLTINHIASITPASFQQLDHLVEIDFRCNCIPVRLGPKDNLCTRRLQIKPSSFSKLTYLKALYLDGNQLLEIPRDLPPSLQLLSLEANNIFWIMKENLTELANLEMLYLGQNCYYRNPCNVSFSIEKDAFLSLRNLKLLSLKDNNISAVPTVLPSTLTELFLYNNIIAKIQEDDFNNLSQLQVLDLSGNCPRCYNVPFPCTPCENNAPLQIHLHAFDALTELQVLRLHSNSLQYVPQRWFQNLNKLKELDLSQNFLAKEIGDAKFLHLLHNLVKLDLSFNYELQVYHTFMNLPDSFSSLKNLKVLRIKGYVFKELKSLNLSPLRNLPNLEVLDLGTNFIKIANLSIFKQFKTLKFIDLSVNKISPSGDSSESGFCSGMRTSAESHGPQVLESLHYFRYDEYARSCRFKNKEPSSSLPLNEDCSMYGQTLDLSRNNIFFIRSSEFQHLTFLKCLDLSGNSISQALNGSEFQPLVELKYLDFSNNRLDLLHSTAFEELRNLEVLDISSNSHYFQSEGITHMLDFTKNLKVLKKLMMNNNDIATSTSTTMESESLRILEFRGNHLDILWRDGDNRYLKFFKNLHKLEELDISENSLSFLPSGVFDGMPPNLKTLSLAKNGLKSFNWGKLQYLQNLETLDLSYNQLKTVPERLSNCSRSLKKLILKNNEIRNLTKYFLQDAFQLRHLDLSSNKIQVIQKTSFPENVLNNLQILFLHHNRLLCNCDAVWLVWWVNHIEVTIPFLATDVTCMGPGAHKGQSVVSLDLYTCELDLTNFVLFSLSLSAVLFLIVITIANHLYFWDVWYSYHFCKAKIKGYQRLISPNSCYDAFIVYDTKDPAVTEWVLDELVAKLEDPREKHFNLCLEERDWLPGQPVLENLSQSIQLSKKTVFVMTDKYAKTEKFKIAFYLSHQRLMDEKVDVIILIFLEKPLQKSKFFQLRKRLCGSSVLEWPTNPQAHPYFWQCLKNALATDNHVTYSQVFKETA

>*Sus scrofa* Toll-like receptor 9 ACZ82294.1

MGPRCTLHPLSLLVQVTALAAALAQGRLPAFLPCELQPHGLVNCNWLFLKSVPHFSAAAPRANVTSLSLLSNRIHHLHDSDFVHLSSLRTLNLKWNCPPAGLSPMHFPCHMTIEPNTFLAVPTLEELNLSYNSITTVPALPDSLVSLSLSRTNILVLDPTHLTGLHALRYLYMDGNCYYKNPCQGALEVVPGALLGLGNLTHLSLKYNNLTEVPRSLPPSLETLLLSYNHIVTLTPEDLANLTALRVLDVGGNCRRCDHARNPCRECPKDHPKLHSDTFSHLSRLEGLVLKDSSLYNLDTRWFRGLDRLQVLDLSENFLYDCITKTTAFQGLARLRSLNLSFNYHKKVSFAHLHLAPSFGHLRSLKELDMHGIFFRSLSETTLQPLVQLPMLQTLRLQMNFINQAQLSIFGAFPGLLYVDLSDNRISGAARPVAITREVDGRERVWLPSRNLAPRPLDTLRSEDFMPNCKAFSFTLDLSRNNLVTIQSEMFARLSRLECLRLSHNSISQAVNGSQFVPLTSLRVLDLSHNKLDLYHGRSFTELPRLEALDLSYNSQPFTMQGVGHNLSFVAQLPALRYLSLAHNDIHSRVSQQLCSASLCALDFSGNDLSRMWAEGDLYLRFFQGLRSLVWLDLSQNHLHTLLPRALDNLPKSLKHLHLRDNNLAFFNWSSLTLLPKLETLDLAGNQLKALSNGSLPSGTQLRRLDLSGNSIGFVNPGFFALAKQLEELNLSANALKTVEPSWFGSMVGNLKVLDVSANPLHCACGATFVGFLLEVQAAVPGLPSRVKCGSPGQLQGHSIFAQDLRLCLDETLSWNCFGISLLAMALGLVVPMLHHLCGWDLWYCFHLCLAWLPHRGQRRGADALFYDAFVVFDKAQSAVADWVYNELRVQLEERRGRRALRLCLEERDWVPGKTLFENLWASVYSSRKTLFVLAHTDRVSGLLRASFLLAQQRLLEDRKDVVVLVILRPDAYRSRYVRLRQRLCRRSVLLWPHQPRGQGSFWAQLGTALTRDNHHFYNRNFCRGPTTAE

>*Gallus gallus* TLR1LA ACR26537.1

MGSLTSIYVFACVFLSILWNNIQPTVENKITANYSGHLLTEVPKNIPVHTHILDLSHNSISEITNFRFTSLSDLQVLNLSHNLITELDFSAFMFNQDLEYLDLSHNNIWTAYCQLLARLRHLDLSFNKFTVLPICQEFGIMFHLEYLGLSAMMIRRSDFRYVAHLQLDTVFLTLEDFSLYEPLSLTALNTRSLHIVFATNQNFNFSLLYDGMSTSEKLKIVNLRYTLSHKDFPSPSLELQKKIKTTDLTLDTVDLEWTVILQIFLLVWDSSVEHLTVRNLIFRGPVVELTEYKHVPLLRSLEQLLSLGSSMKALTLERVRNKLYYFNQEILYRQFSEMNIDSLTIHDACMPHMLCPKKRSSFQYINFSRNALTDELFQNCDTLANLKILILHRNKFESLSKVSFMTSRMKSLRYLDMSSNLLRNSRAEGRCQWADSLAELDLSSNQLTEAVFECLPANINKVDLQNNQIANVPKGITELHSLQELNLASNRLADLPGCRAFTGLEILNIERNLILTPSADFFETCPSVKELQAGQNPFKCSCELQDFLRLERQSGGKLSGWPEAYVCKYPEDLSGTQLEDFHLTELACNTTLLLVTALLLTLVLVAVVAFPCIYLDVPWYVRMLWQWTQTKRRAWHDCPEERETALQFHAFISYSERDSLWVKNELIPNLEKGEGCIQLCQHERNFIPGKSIVENIINCIEKSYKSIFVLSPNFVQSEWCHYELYFAHHRLFSENSNSLILILLEPIPSYVIPARYHKLKALMAKRTYLEWPKERSKHALFWANLRAVVNIKLPTSFETDEEQSDVTSTSSITQCLIK

>*Gallus gallus* TLR2A ACR26453.1

MFNQSKQKPTMKLMWQAWLIYTALAAHLPEEQALRQACLSCDATQSCNCSFMGLDFIPPGLTGKITVLNLAHNRIKLIRTHDLQKAVNLRTLLLQSNQISSIDEDSFGSQGKLELLDLSNNSLAHLSPVWFGPLFSLQHLRIQGNSYSDLGESSPFSSLRNLSSLHLGNPQFSIIRQGNFEGIVFLNTLRIDGDNLSQYEPGSLKSIRKINHMIISIRRIDVFSAVIRDLLHSAIWLEVREIKLDIENEKLVQNSTLPLTIQKLTFTGASFTDKYISQIAVLLKEIRSLRELEAIDCVLEGKGAWDMTEIARSKQSSIETLSITNMTILDFYLFFDLEGIETQVGKLKRLSIASSKVFMVPCRLARYFSSLLYLDFHDNLLVNNRLGETICEDAWPSLQTLNLSKNSLKSLKQAARYISNLHKLINLDISENNFGEIPDMCEWPENLKYLNLSSTQIPKLTTCIPSTLEVLDVSANNLQDFGLQLPFLKELYLTKNHLKTLPEATDIPNLVAMSISRNKLNSFSKEEFESFKQMELLDASANNFICSCEFLSFIHHEAGIAQVLVGWPESYICDSPLTVRGAQVGSVQLSLMECHRSLLVSLICTLVFLFILILVVVGYKYHAVWYMRMTWAWLQAKRKPKRAPTKDICYDAFVSYSENDSNWVENIMVQQLEQACPPFRLCLHKRDFVPGKWIVDNIIDSIEKSHKTLFVLSEHFVQSEWCKYELDFSHFRLFDENNDVAILILLEPIQSQAIPKRFCKLRKIMNTKTYLEWPPDEEQQQMFWENLKAALKS

>*Gallus gallus* TLR2B ACR26415.1

MHTWKMWAICTALAAYLPEEQALRQACLSCDATQSCNCSFMGLDFIPPGLTGKITVLNLAHNRIKVIRTHDLQKAVNLRTLLLQSNQISSIDEDSFGSQGKLELLDLSNNSLAHLSPVWFGPLFSLQHLRIQGNSYSDLGESSPFSSLRNLSSLHLGNPQFSIIRQGNFEGIVFLNTLRIDGDNLSQYEPGSLKSIRKINHMIISIRRIDVFSAVIRDLLHSAIWLDVRKLAFSVPEKIQLLRIMSSSFAKKISLKQCLFTDATVPEIVSILEGMPKLMEVEMKDCTLLGTGKWYKQIHANQSQSLRILTIENLSIEEFYLFTDLQSVLDLLSLFRKVTVENTKVFLVPCKLSQHLLSLEYLDLSANLLGDQSLEHSACQGAWPSLQTLNLSQNSLSDLKMTGKSLFHLRNLNLLDISENNFGEIPDVCEWPENLKYLNLSSTQIPKLTTCIPSTLEVLDVSANNLQDFGLQLPFLKELYLTKNHLKTLPEATDIPNLVAMSISRNKLNSFSKEELTVRGAQVGSVQLSLMECHRSLXVSLICTLVFLFILILVVVGYKYHAVWYMRMTWAWLQAKRKPKRAPTKDICYDAFVSYSENDSNWVENIMVQQLEQACPPFRLCLHKRDFVPGKWIVDNIIDSIEKSHKTLFVLSEHFVQSEWCKYELDFSHFRLFDENNDVAILILLEPIQSQAIPKRFCKLRKIMNTKTYLEWPPDEEQQQMFWENLKAALKS

>*Gallus gallus* TLR3 ACR26371.1

MLEEVKLAELKKLDPENLTEYSNLIYLNAGYNIISKLQPGLCKSLPLLQILKLEHNQLHELPDGVFASCSNLTELNLGYNIIEVKNDPFKTLENLNILDLSHNHLKSANLGLQQQLKNLRELVLYSNQITELNKEDLKFLSNTSLNSLDLSSNPLKEFHTGCLHAIGNLFGLILNNVELGENRTKTLCTELSDTAIQNLSLSHVKLSHINRLTLQGLQGTNLTVLNLSKNSLSVIEDDSFQWLSKLEYLNLEDNNIINVSSHLFYGLSSITHLNLINSLTGKIEDFSFQWLHHLEYLIMDNNNFPRITTNMFTGLKNLKYLSLYNCNTNLQRITNKTFVSLANSSLQVLNLTKTRISTVESGAFSSLGQLKILDLGLNEINQELTGHEFEGLNNIEYIYLSYNKNVTLRSESFIFVPSLRKLMLRKVGCNNLAISPSPFHPLRNLTVLDISNNNIANIKEDLFNGLHELDILNLQHNNLARLWKCANPGGPVLFLKDVPNLHILNLKSNGFDEIPVHVFKGLHQLKDLDLGSNNLNLLPATLFDHQTSLNTLNLQKNLITSVEENVFGPAFKSLRTLEMDFNPFDCTCESISWFASWLNDTQAYIPGLQSQYICNTPPKYHGTLVLHFDTSACKDSAPFKLLFLITTTVVMQFMFIVLLIHFEGWRIAFYWNISINRILGFKELDRLPGVFDYDAYVIHARKDTNWVLTNFTSLEENEQFQVKFCLEERDFEAGISEFEAIINCIRRSRKIIFIVTEHLLQDPWCRKFKVHHALQQAIEQSRDSIILIFLHNIQDYKLNHALCLRRGMFRSCCILNWPVQKERINAFHQQLMMALKSNSK

>*Gallus gallus* TLR4 AJR32867.1

MPSRAAPTALTLGVLLQLLLVLSLLAGCIPSPCLEVIPSTAFRCTGQNISGVPAEIPNTTLDLDLSFNSLKLLSSNYFSSVPELQFLDLSRCHIHTIEDNSFVDLYNLSTLILTANSLQHLGLAAFHGLTSLKKLVLVETSISSLSDLPIGHLNTLQELNLGHNNIASLKLPKYFANLTSLRHLSFSSNNITYISKGDLDALRETNRLNLTLVLSLNNIKYIQSGSFAKIHLGELILRSSFENLNAMHSSLQGLAGLQVNRLIVGEFTNILKITAFQNGLLSGLCQVQMQEFVLMCFREFENDTDTLFDCIGNVTTIRLVDLNLETLSEVPMFSQVKHLEWKRCKFQELPAEKLSLFKELRVLRITKSKDLNGFEQKFGSLTHLEVVDLSENRLSFLTCCSPKFPRSPNLKHLNLSFNSDISLTGEFANLRNLLYLDLQHTKLIHHGTYPVFLLLQKLIYLDISYTKTHVMSHLIFHGLNSLQVLKMAGNSFENNTLTNNFENVRRLRILDISSCKLVWVDQSTFNALSELKELIISNNKLLTFDPVTYKPLQALTALDFSNNQMSFLSDSALEILPDSLVLLDISHNLFECSCTHLNFLKWVKEKQDLLRNKHSMICHTPAYMKNMSLSNFDMSSCHPNPTTVACSVTVLLAAGVFLFLIYKYYFQLYYSLVLLSGCKHSAERGDIYDAFVIHSSKDQEWVMKELVEPLEEGKPPFQLCLYFRDFLPGVPIVTNIIQEGFLSSRNVIEVISADFLESKWCSFEFDIARSWQLVEGKAGIIMIILGEVDKTLLRQRLGLSRYLRRNTYLEWKNKEISRHIFWRQLTSVLLEGKKWNHEEIKLM

>*Gallus gallus* TLR5 ACR26275.1

MMLHQRLIIVFGIALAGDICASRSCYSEDQVSMYNSCNLTGVPPVPKDTAKLFLTYNYIRQVTATSFPLLEDLFLLEIGTQRVFPLYIGKEAFRNLPNLRVLDLGFNNILLLDLDSFAGLQRLTILRLFQNNLGDSILEERYFQDLRSLEELDLSGNQITKLHPHPLFYNLTILKAVNLKFNKISNLCESNLTSFQGKHFSFFSLSTNTLYRTDKMIWAKCPNPFRNITFNSLDVSENGWSTETVQYFCTAIKGTQINYLSFRSHTMGSGFGFNNLKNPDTDTFTGLARSDLHLLDISNGFIFSLNSLIFESLRNLEFLNLFRNKINQIQKQAFFGLENLEILNLSSNLLGELYDYTFEGLHSIMYIDLQQNHIGMIGEKSFSNLVNLKIIDLRDNAIKKLPSFPHLTSAFLSDNKLMSVAHTAIVATHIELERNWLANLGDLYVLFQVPGVQYLLLKQNRFSYCVKHVDAIENNQLIYMDLGENMLQLVWERGLCLDVFRTLSKLQVLHLNNNYLSALPQEIFNGLTSLKRLNLASNLLSHLSLRVFPQSLINLNLSGNQLFSPKPEVFMTLSILDITHNKYVCDCALKSLLVWLNETNVTLAGSESDRYCVYPPALAGVPVSFLTYDDCDEDELQQTLRFSVFVFLSVTLLMFLMSTIIFTRCRGICFVWYKTITKTLIGSHPPAADTSEYMYDAYLCYSKNDFEWVQNSLLKHLDSQYFDKNRFTLCFEERDFLPGEEHINNIRDAIWKSRKTICVVTRQFLKDGWCVEAFNFAQSRYFSDLKEVLIMVVVGSLSQYQLMKHKPIRIFLQRSRYLRWPEDYQDIGWFLDNLSSQILKEKKVQRNVSGIELQTIATVSH

>*Gallus gallus* TLR7 ACR26243.1

MTNLSEVAAHRKMVHHARTSNALLFVLLFLFPMLLSGRWFPKTLPCDVEAFESTVRVDCSDRRLKEVPRGIPGNATNLTLTINHIPRISPASFTQLENLVEIDFRCNCVPPRLGPKDNVCVTPPSIENGSFAALTRLKSLYLDANQLSKIPRGLPATLRLLSLEANNIFSIKKNTFSELRNIELLYLGQNCYYRNPCNVSFEIEETAFLNLKNLTVLSLKSNNLTFIPPNLSSTLKELYIYNNRIQEVQEHDLSNLYNLEILDLSGNCPRCYNAPYPCTPCPNISIKIHSKAFYSLKKLRILRLHSNSLQSIPSSWFKNIKNLKNLDLSQNFLIKEIGDAEFLKLIPSLVELDLSFNFELQMYSPFLNLSKTFSCLSNLETLRIKGYVFKELREENLDPLLNLRNLTVLDLGTNFIKIADLRVFKKFRSLKIIDLSMNKISPSSGEGNFYGFCSDHRITVEQYSRHVLQEMHYFRYDEYGRSCKSKDKEADSYQPLVNGDCMSYGETLDLSRNNIFFVNSIDFQDLSFLKCLNLSGNAISQTLNGSEFYYLSGLKYLDFSNNRIDLLYSTAFKELKFLEILDLSNNKHYFLAEGVSHVLSFMKNLAYLKKLMMNENEISTSISTGMESQSLQTLEFRGNRLDIFWSDGKKEYLSFFKNLTNLEQLDISSNMLNFLPPDVFEAMPPELKILNLTSNRLHTFNWGKLHLLTKLITLDLSNNLLTTVPRKLSNCTSTLQELILRNNRITRITKYFLRGAIQLTYLDLSSNKIQIIKKSSFPENIINNLRMLLLHNNPFKCNCDAVWFVGWINQTQVAIPLLATDVTCAGPGAHKGRSLVFLDLNTCELDTSYFIMYALSTSAVLCLMMFAVMSHLYFWDVWYSYHYCTAKLKGYRRIPLPDACYDAFIAYDNTDLAVNEWVMTELVEKLEDQKARQFNLCLEERDWLPGQPVFDNLSQSIQLSKKTIFVLTNKYIKSGTFKTTFYMAHQRLLDEKIDVIILIFLEKVLQKSRYVQLRKRLCRSSVLEWPTNPRSQPYFWQRLKNAIAMNNTLSYNKLLQETV

>*Gallus gallus* TLR15 ACR26576.1

MRILIGSLYFYFISFLFSKVNGFLTQRTSPVSSFPFYNYSYLNLSSVSQAQAPKTARALNFSYNAIEKITKRDFEGFHVLEVLDLSHNHIKDIEPGAFENLLSLVSVDLSFNDKNLLVSGLAPHLKLIPTSGASGPSQIYMYFQKSAEAALEPSAPAELLPHLEDPPNPGNVNPRFRQRRTEENKTSPPAATLRPDLCGAPINGLLDLSRTKLSNEELTAKLDADLCQAQLGTVLEFNISHSDLEMDLLSLFILFLPMKDIQSVDASYNRITINNIDVEAICHFPFSNFSFLNISNNPINSLETVCLPASITVIDLSFTNISTIPANFAKKLSKLERMYVQGNQLIYTVRPENPSATPRPPPGTVQISAISLVRNQAGTPIESLPESVKHLKVSNCSIVELPEWFANRMQELLFLDLSSNRISMLPDLPISLQQLDISNSDIKIIPPRFKSLSNLTVFNIQNNKLTEMHPEYFPSTLTTCDISKNKLKVLSLTKALENLESLNVSGNLITRLEPACQLPSLTNLDSSHNLISELPDHLGQSLLMLKHFNLSGNKISFLQRGSLPASLEELDISDNAITTIVQDTFGQLTSLSVLTVQGKHFFCNCDLYWFVNIYIRNPHLQINGKDDLRCSFPPDRRGSLVKSSNLTLLHCSLGIQMAITACMAILVVLVLTGLCWRFDGLWYVRMGWYWCMAKRRQYKKRPENKPFDAFISYSEHDADWTKEHLLKKLETDGFKICYHERDFKPGHPVLGNIFYCIENSHKVLFVLSPSFVNSCWCQYELYFAEHRVLDENQDSLIMVVLEDLPPDSVPQKFSKLRKLLKRKTYLKWSPEEHKQKIFWHQLAAVLKTTNEPLVRAENGPNEDVIEME

>*Taeniopygia guttata* Toll-like receptor 4 ACN58233.1

MPRRGAPPVGTLLVLLQLAFVPSPLAGCLLDPCLEITPNTTFRCTGLNISGVPDGVPNTTQNLDLSFSNLKSLGSNYFASVPELQILDLSRCHLHTIEDNSFMDLPRLSTLVLTANSFQHLGKAAFYGLTSLKKLVLVETNRTSLSELPIGHLHTLQELNLGHNSITSLKLPKYFTNMTSLRLLSFSSNKITSISRGDLDALREGNRLNLTLVLSLNNIKSIEPGAFAGIHLAELALRSAFEKPTMQTALQGLAGLQVSRLIVGEFRNIERLQDFEGRLLDGLCQVQMEEFVLICLRGFKDDTDTLFNCVGNVSTIRLVDLGLKEISQVPVWSKVRQLECKKCHFEDVPAQKLSLFKELRVLRINDNRNLKTFEHKFKGLSNLEVIDLSENRLTFSSCCSPQFQNCPNLKHLNLSFNSNIRLTGDFTNVKNLLYLDLQHTTLFGPGSYPVFLSLQRLIYLDISYTKTEVKSQCTFCGLNSLQVLKMAGNSFADNKLANNFKNLSHLHTLDISSCKLEHVDQSTFDALSELKELNISNNKLMTFDPVVYQPLQALRVLDFSRNQLAVLLDPARGILPDSLVLLDISQNLFDCSCVYLDFLKWVKEKQELLQNEELMLCHTPSYVANMSLPSFDLSSCHISAGQVASPVVVLFCVVVFLFLVYRYYFQLYYSMVLLSGCKHYAERGDTYDAFVIHSSKDQEWVMKELVEPLEGGTPRFRLCLYYRDFLPGVPIVTNIIEEGFLSSRNVIAVISADFLQSKWCSFEFDIAQSWQLVEGKAGLIMIVLEDVNKALLRQRLGLSRYLRRNTYLEWKNKEISKHIFWRQLIGVLLEGKNWNHEEEKLM

>*Pelodiscus sinensis* Toll-like receptor 4 AGV08599.1

MLGRGALSFPTCLVLSSAILFLSQLMACSFNPCVEVIPNITYRCMDLNLSGVPAEIPPSTENLDLSFNPLVSLTSNYFSEVPELRLLDLTRCHIQTIEDYAFKDLHKLLTLILTANPLQHLGPTAFYGLASLQRLIAVEDDISSLADLPIGHLHTLQELNVANNQVDSLKLPGYFSHLSLLRFLSLKSNRISSISTGDLAALQGEKKHNLTLVLSLNDIKCIQPGSFEGILLHELSLRACFENTGLMKACIQGLAGLQVNRLVLGEFRNIGRVEDFSNGLLDGLCQVQLQEFVFISFQRFDNCTDTLFDCLVNASTIRLVDLSLDQVSAVPTASRIHHLEFNTCKFIEVPAKKLSSFKELRVLRITNSKYLTGFREKFKDLPNLEVLDLSVNRLSFIQCCDSYLKRIPKLKHLNLSFNSIISVTGGLNITELVSLDFRHSKLNDFGSFPVFHCLGKLIYLDISYTSSHIASQCTFSGLKSLQVLKMAGNSFQDNNLGNSLMNLTQLLTLDVSSCKLTQVSPSTFKSLTKLQELNISHNKLLAFDPLAYKPLQALTVLDFSSNQLTVLTEKAMGSLPSCLVHLDLSYNLFDCSCNHLSFLKWAKEHGELLKSTQLMVCNNPVHMQNMSVLSFDLSSCHLSPVEWVMSVSVPFAGILFVILIYKYYFLLYYGLVLHSGCRRYTDRNDTYDAFVIHSSKDIEWVRKELAEKLEGGMPPFQLRLHYRDFIPGVPITTNIIQEGFLSSRRVIAVISNHFMESKWCSFELEIAQSWQFVDGKASLIMIVLEEVDKVLLRRKLELSRYLRRNTYLEWRDQEISRHLFWRQLRTALLDDKT

>*Meleagris gallopavo* Toll-like receptor 5 ADX33343.1

MMLHQRLIIVFRISLAGDICASRSCYSEDQVSMYNSCNLTSVPPVPKDTAKLFLTYNYIRQVTATSFPLLEDFFLLEIGTQRVFPLYIGKEAFKNLPNLRVLDLGFNNILLLDLDSFAGLQRLTILRLFQNNLGDSILEERYFQDLSSLEELDLSGNQITKLHPHPLFYNLTILKAVNLKFNKISNLCESNLTSFQGKHFSFFSLSSNTLYKTDKMVWAKCPNPFRNITFNSLDVSENGWSTETVQYFCTAIKGTQINYLSFRLHTMGSGFGFNNLKNPDTDTFTGLARSDLRLLDISNGFIFSLNSLIFESLRNLEFLNLFRNKINQIQKQAFFGLENLEILNLSSNLLGELYDYTFEGLHSIMYIDLQQNHIGMIGEKSFSNLVNLKIIDLRDNAIKKLPSFPHLTTAFLGDNKLMSVAHTAIAATHIELERNWLANLGDLYVLFQVPGVQYLFLKQNRFSYCVKHVNAIENSQLIYMDLGENMLQLVWERGLCLDGVQVTGLNFRVLHLNNNYLSALPQEIFNGLTSLKRLNLASNLLSHLSLRVFPQSLINLNLSGNQLFSPKPEVFMTLSILDITHNKYVCDCALKSLLVWLNETNVTLAGSESDRYCVYPPALAGVPVSFLTYDDCDEDELQQTLRFSVFIFISVTLLMFLMSTIIFTRCRGICFVWYKTITKTLIGSHPPAADTSEYMYDAYLCYSKNDFEWVQNSLLKHLDSQYFDKNRFTLCFEERDFLPGEEHINNIRDAIWKSRKTICVVTRQFLKDGWCVEAFNFAQSRYFSDLKEVLIMVVVGSLSQYQLMKHKPIRIFLQRSRYLRWPEDYQDIDWFLDSLASQILKEKKVQRDVSGTELQTIATVSR

>*Meleagris gallopavo* Toll-like receptor 15 ADR01193.1

MRVLTGSLYFCFISFLFSRANGFLVQTTSPMSSFPFYNYSYLNLTSVSEAQAPKTARALNFSHNAIEKITKRDFEGFDVLEVLDLSYNHIKDIEPGAFENLLSLVSVDLSFNDENLLVSGRASHLKLIPTSGALGPSQTYMYFQKSAEATLVPSTPAELLPHLEDPSNPGNVSPRFRQRRAEENTTSPPAATLRPDVCGAPINGLLDLSRTKLSDEELAAKLDADLCQAKLGAVLEFNISHSDLEMDLLSLFILFLPMKDIRSVDASYNRITINNIDVEAICHFPFSNFSFLNISNNPINRLETVCLPPTIRVIDLSFTNISTIPANFAKKLSKLECMYVQGNQLIYTVRPESSSAATRPPPGTVHISAISFVRNQAGTPIESLPERVKHLKASNCSIVELPEWFANRMQELLFLDLSSNRISMLPDLPISLQHLDISNSDIKMIPPSFKSLSNLTVFNIQNNKITDMHPEYFPSTLTTCDISKNKLKVLTLTRALVNLESLNVSGNLITRLEPTSQLSALTNLDSSHNLISELPDNFGQSLLMLKHFNLSGNKISFLQRGSLPASLEELDISDNAITTIVQDTFGQLTSLSVLTVQGKHFFCNCDLYWFVNVYIHNPHLQINGKDDLRCSFPPDRRGSLVKSSNLTLLHCSLGIQMAITACMAILVVLVLTGLCWRFDGLWYVRMGWYWCMAKRKQYKKRPENKPFDAFISYSEHDADWTKENLLKKLETDGFKICYHERDFKPGHPVLGNIFYCIENSHKVLFVLSPSFVNSCWCQYELYFAEHRVLDENQDSLIMIVLEDLPPNSVPQKFSKLRKLLKRKTYLKWSPEEHKQKIFWHQLAAVLKTTNEPLVRAENGPNEDIIEME

>*Fukomys damarensis* Toll-like receptor 1 KFO32856.1

MTQMKLRIFYFAIIFMLICEIKPQVSDESEFLVDRSQTGLTHVPKDLSLKTTTLNISQNYISEIQSSDILSLSKLRILMLSHNKIQYLDISVFKFNRELEHLDLSHNKLRKISCHPTVNFKHLDLSFNAFDDLPICKEFGNLAQLKFLGLSATQLRKSSVQPVAHLNISEVLLVLGDSYGEEEESDSLRYFNTRSLHVVFPTKKEFRFILDVSVSTAVNLELSNIRCVLPDDECHFLDVLSKLHKTPRLSKLTLNNIETSWSIFIAIIQIGWHIKMEYFSISNVKLQGHLGFRKFDYSNTSLKALSIQQVVSDVFSFPQSNIYSIFSNMKIQNFTVSATHMLHMLCPSQISPFLYLDFSNNLLTDIIFKNCTTLTALKTLSLKMNQLKELANIILMTKEMKSLQQLDISQNSLRYNEDENTCSWTKSLLSLNMSSNTLTDSVFRCLPPKVKILDLHNNRITNIPKNVTHLQALQELNIAFNSLTDLPGCGTFSSLSALIIDHNSMSHPSADFFQSCQKIRSMRAGNNPFLCTCELREFIKNIDQVSSEVAQDWPDSYKCDYPESSKGVLLKDFHVSPLSCNTTLLILTVVTTMLVLAATVTFLCHYFDLPWYLRMMCQWTQTRHRARNICLEELQGTLQFHAFISYSGHDSAWVKNELLPNLEKENLQICLHERNFVPGKSILENIINCIEKSYKSIFVLSPNFIQSEWCHYELYFAHHNLFHGHFDNLILILLEFIPQYSIPSSYHKLKALMAQRTYLEWPAEKSKHGLFWANLRAAININLMNQVKEINHT

>*Fukomys damarensis* Toll-like receptor 2 KFO31679.1

MSRALWTVWLLGLVIRPSKEGSLGRASLSCDPAGVCDGRSRSFTSVPSGLSASVKSLDLSNNRITSVGRRDLHRCVNLKTLVLKSSGIHTIEGDAFLSLGRLEHLDLSKNHLSELSSLWFRPLSSLKFLNLLENPYQTLGEMSLFSHLSNLRILRVGNGAFIGMQKIDFAGLTLLEELEIEASNIQSYEPQSLKSIQSIGHLILHMREPLFLMEVFVDILGSVEHMGLSGTNLNEFSFSDLSVSVTNSSIKKLTFRNVKFADGSFNDVLRLSGYVPELLEVEFEDCIYNGIGNFGVSGIEEVYPGKADTITIRRLYIPNFYLFYDLSDMYSLAERFKRITLENSKVFLVPCSLSRHLKSLEYLDLSENLMVEENLGNAACEGGWPSLQTLVLRKNHFTSIEKTGEVLLTLHNLTSLDISKNSFHSMPEICQWPEKMSYLNLSSTQIHRLTSCIPQTLEILDVSNNDLSSLSLFLPRLQELYISRNKLKTLPEGSRLPALRVLKIRRNMINTFSKEELDSFHGLQALEAGGNSFICSCDFLSLAWERRGLPEVLSDWPAGYRCDSPRDLCFDAFVSYSERDARWVEELLVQELEHRHPPLRLCLHKRDFVPGKWILDNIIDSIEKSRKTVFVLSQNFVKSEWCKYELDFSHFRLFDENDDAAILVLLEPIEKKAIPQRFCKLRKIMNTRTYLEWPAEEARREGFWANLRSAIKA

>*Fukomys damarensis* Toll-like receptor 4 KFO26787.1

MTSLRGLAGTLLPAMAFLAFARPEIWEPCVEVVPNITYDCMEQNLYKIPSSIPSSAKHLDLSFNPLMFLGSQSFTAFPELEVLDLSRCEIQRIEDDAYWGLNYLSTLILTGNPIQILAPGAFSGLLSLQKLVAVEINLSSLENFPIGHLKTLKELNVAHNLIHSFKLPKYFSYLTNLEYLDLSNNKIQNIYHKDLHVLYNKSKLSLDLSLNPFDFIQPGAFDKMRLQELTLRSNFDNTNVMKTCIQGLAGLEVNQLVLGEFKNERHIEEFDKSALEGLCSVATEEFRLPYLDDFPNNFNGFFDCLVNVSAVSLANLHLKSLEGLPNGLKWQSLEMVRCKVNNFPPFAIPSLKRFSFTANRVENSFAGLTLKSLEFLDLSGNGMSFKGCCSLKDLETPNLKYLNLSFNDVITMSSNFMGLELLEHLEFQYSTLKQTSEFSVFLSLSKLLYLDISYTHTHVAFPGIFSGLVSLQVLKMAGNSFKDNTLSSIFAELTNLTFLDLSKCQLEYVSQGVFDKLLRLQFLNMSYNDFLLLDALSFKPLHTLQGLDLSFNRIASSNGQELQNFPSNLTTLNLTQNPFDCSCEHQSFLQWITDQKRLLVEAEQLECVTPPDMQGKMVLSFRNDTCQIPKVIIIVTVVSVLVVSVIACLVYKFYFHLMLLAGCKKYGRGENTYDAFVVYSSQDEDWVRNELVKNLEEGVPPFKLCLHYRDFIPGVAIAANIIQEGFHKSRKVIVVVSQHFIQSRWCIFEYEIAQTWQFLSSQSGIIFIVLQKLEKSLLRQQVELYRLLSRNTYLEWEDHALGRHIFWRRLRKALMDGKTWSPEGTADAENIQQEATTLT

>*Fukomys damarensis* Toll-like receptor 6 KFO32858.1

MTQDKETVIRTFYLICIMTLIVGTITQICEESEFKVCMSKNNLTRIPKTLLPTTKSLDISQNYISELQVSDISFLSGLKVLKLSHNRIRRLDLSVFKFNQDLEYLDLSHNQLQKMCCYPVVSLKHLDLSFNDFDVLPICVEFGNLTQLNFLGLSAVRFQQIDLLPVAHLHLNYILLVLGDYYIKENVSEKLQILNTKTLHFVFHPNSLFSVQVNISVNALGCLQLTNIKLNERNCQVFVKFLLGLIRGPMLLNITLNNIETPWKCLVEILKFLWPRPVEYLNIYNLTLDEIVSDIQVTYPETTLKALKIEHITKRVFIFSQTVLYKVFSEMNIMMLTISDTPFIHMLCPVASSTFKFLNFTQNVFTDSVFDTCSTLVRLETLILRKNSLKNLYKVSLMTKNMPSLEILDVSWNSLESHRYEENCMWVESIVVLNMSSNTLTDSVFRCLPPKVKILDLHNNRITNIPKNVTHLQALQELNIAFNSLTDLPGCGTFSSLSALIIDHNSMSHPSADFFQSCQKIRSMRAGNNPFLCTCELREFIKNIDQVSSEVAQDWPDSYKCDYPESSKGVLLKDFHVSPLSCNTTLLILTVVTTMLVLAATVTFLCHYFDLPWYLRMMCQWTQTRHRARNICLEELQGTLQFHAFISYSGHDSAWVKNELLPNLEKENLQICLHERNFVPGKSILENIINCIEKSYKSIFVLSPNFIQSEWCHYELYFAHHQLFRKGSNNLILILLEPISQNDIPIAFWCSECLTLASARLTPALDLEPLSLESAWLSSPLRLTPTPPGQRASRRPLATS

>*Fukomys damarensis* Toll-like receptor 7 KFO19283.1

MILISKLLEARWFPKTLPCDISLDPSKSHVLVDCTDKHLTEIPKGIPTNATNLTLTINRIPNISPDSFHNLDHLVEVDFRCNCVPTRMGAKNNVCTRRLQIKPRSFSRLTYLKALYLDANQLLQIPQDLPPSLQLLSLEANNIFFIMKKNLTELVNIEMLYLGQNCYYRNPCNVSYSIEKNAFLNLRNLKVLSLKDNNITAVPTLLPSNLTELYLYNNIITNIQKDDFNNLNHLQILDLSGNCPRCYNVPFPCTPCENNSALQIHINAFDSLTELQVLRLHSNSLQHVPLEWFKHMSNLKELDLSQNYLAKEIGDAKFLHFLPNLIQLDLSFNYELQVYHASINLSHSFSSLKNLKILRIKGYVFKELKCLNLSPLHDLHNLEVLDLGTNFIKIADLSIFKQFQRLKLIDLSMNKISPSGDSSETGFCSNSRTSAESNGPQVLETLHYFRYDEYARSCRVKNNESSFFALNNDCYMYGQTLDLSRNNIFFVKPSDFQHLSFLKCLNLSGNAIGQTFNGSEFQHLEELKYLDFSNNRLDLLHSTAFEELHNLEILDLSSNSHYFQSEGITHMLNFTKNLKFLKKLMMNDNDISTSTSRTMESKSLKILEFRGNHLDILWRDGDNRYLKFFQNLLNLEVLDISRNSLSFLPPGVFEGMPPNLKNLSLAKNGLRTFNWGKLPFLKNLEFLDLSHNQLTTVPERLSNCSRTLRTLILMKNQIGHLTKYFLEDAFQLQYLDLSSNKIQVIQKTSFPQNVLNNLETLLLHHNRFLCNCDAIWFVWWVNHTDVTIPYLATEVTCAGPGAHKGQSVVSLDLYTCELNLINLILFSVSISVALFLMVVMTASHLYFWDMWYIYHFCKAKIWGYQRLRSAECCYDAFIVYDTKDPAVTEWVLDELVAILEDPREKHFNLCLEERDWLPGQPVLENLSQSIQLSKKTVFVMTEKYAKTENFKIAFYLSHQRLMDEKIDVIILIFLEKPLMKSKFLQLRKRLCGSSVLEWPTNPQAHPYFWQCLKNALAMDNHVVYSQMFKETA

>*Fukomys damarensis* Toll-like receptor 8 KFO19280.1

MPLQSSFLICIFLLSSGSCEFFAEYSRSFPCDESRHNASVIAECNHRRLQEVPKTVGRNVTDLDLSDNFITHITNESFQGLQKLTRINLNHNANQQHQDENPSINKSGMNITDGAFLSLKNLKELLLEDNQLYKIPTGLPESLREFSLIQNNIVLVTKKNTLELGNLEKFYLSWNCYFACNKTFEVKDGAFEKLTNLKELSLSFNNLYRVPPKLPSSLVTLYLSNAKIQTIGQEDFKGLENLLSLDLSGNCPRCFNAPFPCTPCEGGASLKIHPNAFQHLTKLLFLNLSSTSLQTVNSIWFDNMTSLKALHLEFNYLVKEIASGGFLTKLPNLEKLDLSFNYVKTEYFQYINLSVNFSKLEKLEALHLRGYVFQKLRREDFQPLMGLPHLKTINLGVNFIKQIDFKIFKRFPSLKVIYLSENRISPVVDDTRQNCINGSPLQNHNRKPRSPDSEFDPHSNFYHPTHPLMKPQCTSYGKALDLSLNSIFFIGRKQFEDFHDIVCLNLSSNGNAQVFNGSEFASVSQVKYLDLTNNKLDFDDDHALSSLHNLEVLDLSRNAHYFKIAGVTHRLGFLQNLTHLKVLNLSYNSIYTLTEEKELKSKSLEELNFSGNRLDLLWKAGDHSYWPIFKCLSNLTRLDLSDNNLQRIPNEALLSLPQGLIHLYLKDNILNFFNWTVLQQFPQLQVLDLSGNQLSSLTSSLSRFTSSLRTLLLSQNRIFHLPSGFLSGASSLIHLDLSSNLIKMINKSTLQIKTTTNLASLKLDGNPFDCTCDIGDFRRWMDENLNIAIPRLADVICASPGDQSGKSIVTLELTTCVSDSTAAALFFLTFFITTLVMLVALAHHFFYWDVWFMYQMCLAKVKGYRSLSTSQTFYDAYISYDTRDASVTDWVINELRYHLEESKDKNVLLCLEERDWDPGLAIIDNLMQSINQSKKTIFVLTKKYAKSWNFKTAFYLALQRLMDENMDVILFILLEPVLQHSQYLRLRRRICKSSVLQWPDNPKAEGLFWQSLKNVVLTENDSRYNNLYVDSIRPY

>*Loxodonta africana*Toll-like receptor 3 ABC95781.1

MSQGLPYRIYSFWGLLPLWMLCASSANKCVVRREVADCSHLRLTQVPADLPANITVLNLTHNQLRKLPPANFTRYSQLIILDGGFNSISKLESELCQKLPLLKVLNLQHNELSQLSDKTFVYCVNLTELHLTSNSIQKIKSNPFKNLKNLIKLDLAHNGLSSTKLGTELQLENLQELSLSNNKIHALRREELDFLGNSSLKKLELSSNQIKEFSSGCFHAIGKLSGLFLNNIQLGSSLTEKLCLELSNTSIQSLSLSNTQLYRTSNMTFFGLKETNLTMLDLSHNSLNKIANDSFAWLPHWEYLTLENNNIEHLSSRSFYGLLNVRYLNLKRSFTKQSTSLASLPKIDDFSFQWLKYLEHLNMEDNNFPGIKNNMFTGLIRLKYLSLSSSFTSLPTLTNETFLSLANSPLLILNLTRNKISKIESGAFSWLGHLEVLDLGLNEIGQELTGQEWRGLGNIVEIYLSYNKYLQLTSNSFALVPSLQQLMLRRVACKNVDISPSPFCSLRNLTILDLSNNNIANINEDMLEGLDKLEILDLQHNNLARLWKHANPGGPVHFLKGLSHLHVLNLESNGFDEIPQGAFKDLSELKSIDLGLNNLNILPPSQFDSQTSLKSLKLEKNLITSVEKNVFGPPFKNLTNLDMRFNPFDCTCESIAWFVNWINGTHANISGLPDDYLCNTPPQYHGLSVTFFDTSACKNSAPFELLFVINTSTLLFFIFIVLLVHFEGWRISFYWNVSVHRILGFKEIDRQPEQFEYAAYVIHAHKDRDWVWQHFSPMEEKDQSLKFCLEERDFEAGALELEAIVNSIKRSRKTIFVITQHLLKDPLCKRFKVHHAVQQAVEQNLDSIILIFLEEIPDYKLNHALCLRRGMFKSRCILNWPVQKERISAFHHKLQVALGSRNSVY

>*Loxodonta africana* Toll-like receptor 7 ABC95782.1

MVFWMRTLNRLFLILLNVVLISKPLGAIWFPKTLPCEVTVDVPKAHVIVDCTDKHLMEIPGGIPTNATNLTLTINHIPAISPASFRRLEHLVEIDFRCNCVPIRLGPKDNICTRGLQIKPKSFSRLTSLKALYLDGNQLLEIPQGLPAGLKLLSLEANTIFSIMKENLTELANIEILYLGQNCYYRNPCNVSFSIEKDAFLSLKNLKVLSLKDNNITAVPTVLPSTLTELYLYNNIITKIEEHDFNNLNQLQHLDLSGNCPRCYNVPFPCTPCENNSPLQIHVNAFDALLELRVLRLHSNSLQHVPKRLFQNIKKLEELDLSQNFLAKEIGDAKFLHFLPNLVQLDLSFNYELQVYHAHMNLSNEFSSLRNLRVLRIKGYVFKELKSLNLFPLQSLPYLEVLDLGTNFIKVADLSIFKQFERLKFIDLSMNKISPSGDSSDVGFCPNSRTSVDSYGPQVLETGHYFRYDDYARSCRFKYKETPVLPFNENCNMYGQTLDLSKNNIFFIKSSDFQHLSFLKCLNLSGNSISQTLNGSELQPLVELKYLDFSNNRLDLLHSTAFEGLRNLEVLDISSNGHYFQSEGLTHMLNFTKNLNVLKKLMMNNNGISTSSSRTMESNSLKTLEFRGNYLNVFWKDGDNRYLQFFKNLQNLEELDISENSLSFLPPGVFDGMPPNLKTLSLAKNGLKSFNWGRLEVLKNLETLDLSNNQLKTVPERLANCSSSLKKLILKNNQIRRLTKYFLQGAFQLRYLDLSSNKIQIIQKTSFPENVLNNLELLLLHHNRFLCTCDAVWFVWWVNHTEVTIPYLATDVTCVGPGAHRGQSVVSLDLYTCELDLTNLILYSFSISIALFLMVVTTASHLYFWDVWYSYYFCKAKIKGYRRLISPDSCYDAFIVYDTKDPAVTEWVLDELVAKLEDPREKHFNLCLEERDWLPGQPVLENLSQSIQLSKKTVFVMTDKYAKTENFKIAFYLSHQRLIDEKVDVIILIFLEKPLQKSKFLQLRKRLCGSSVLEWPTNPQAHPYFWQCLKNALATDNHVAYSQVFKETV

>*Loxodonta africana* Toll-like receptor 8 ABS28968.1

MIPQSSLLICLFLLIAASWEFFAEANYTRSYPCDERKQNGSVIAECNNRQLQEVPQTVGRYVTELDLSNNFIKHITNESFQGLQNLMKINLNHNAMNNNAMNITDGAFLNLKNLRELLLEDNQLYQIPAALPESLKELSLIQNNITVVTKKNASGLMNLERLYLGWNCYFGHDCKKLFDIESGTFEKLTNLKVLSLSFNDLSHVPPKLPSSLTELYLSNTNIQIIRQEDFKELKNLTILDLSGNCPRCFNAPFPCKPCEGNSSIQIHPLAFQTLTQLRYLNLSSTSLQKIPATWFYNMTRLKVLYLEFNYLLYEIASGAFLTYLPSLEILDLSFNYARTEYPQYINLSKNFSKLESLQVLHLRGYVFQELRKEEFRPLLGLRNLRAINLGINFIKQIDFTIFQHFPNLSIIYLSENRISPLVSDMRQDDTNRSSSQSHILQPRSAYTEFDPHSNFYHSSNPLVKPQCSAYGKALDLSLNSIFFIGQKQFEAFDDIACLNLSSNGNGQVLHGTEFSAVPHIKYLDLTSNRLDFDDDKALTELVELEVLDLSYNAHYFRIAGVTHRLGFITNLTQLKVLNLSHNSIYTLTEYNLTSMSLEELVFSGNRLDLLWNYEDDRYWHLFTFLRNLTRLDLSFNNLRRIPNEAFRNLPHSLRELYINNNVLNFFNWTLLQHFPHLRLLDLSRNKLSTLTNSLSAFTSSLQTLLLRQNRISHLPSGFFSEARSLAHLDLSSNLLKMINKSTLQTKTATNLAILELGGNPFDCTCDIGDFRRWMDENLNVSIPRLTDVICASPGDQRGKCIVSLELTTCVSDAIAAILFFFTFFVTTMVMLIALAHHLFYWDVWFIYHVCLAKIKGYRSLSTSQTFYDAYISYDTKDASVTDWVINELRYHLEESEDKNVLLCLEERDWDPGLAIIDNLMQSINQSKKTIFVLTKKYAKNWSFKTAFYLALQRLMEENMDVIIFILLEPVLQHSQYLRLRQRICKSSILQWPDNPKAEGLFWQSLKNVVLTENDSRYNNLYVDSIK

>*Pan troglodytes* Toll-like receptor 1 BAG55015.1

MPSIFHFAIIFMLILQIRIQLSEESEFLVDRSKNGLIHVPKDLSQKTTILNISQNYISELWTSDILSLSKLRILIISHNRIQYLDISVFKFNHELEYLDLSHNKLVKISCHPTVNLKHLDLSFNAFDALPICKEFGNMSQLKFLGLSTTHLEKSSVLPIAHLNISKILLVLGETYGEKEDPEGLQDFNTESLHIVFPTNKEFHFILDVSVKTVANLELSNIKCVLEDNKCSYFLSILAKLQTNPKLSSLTLNNIETTWNSFIRILQLVWHTTVWYFSISNVKLQGQLDFRDFDYSGTSLKALSIHQVVSDVFSFPQSDIYEIFSNMNIKNFTVSGTRMVHMLCPSKISPFLHLDFSNNLLTDTVFENCGHLTELETLILQMNQLKELSKIAEMTTQMKSLQQLDISQNSVSYDEKKGDCSWTKSLLSLNMSSNILTDTIFRCLPPRIKVLDLHSNKIKSVPKQVVKLEALQELNVAFNSLTDLPGCGSFSSLSVLIIDHNSVSHPSADFFQSCQKMRSIKAGDNPFQCTCELREFVKNIDQVSSEVLEGWPDSYKCDYPESYRGSPLKDFHMSELSCNITLLIVTIVATMLVLAVTVTSLCIYLDLPWYLRMVCQWTQTRRRARNIPLEELQRNLQFHAFISYSGHDSFWVKNELLPNLEKEGMQICLHERNFVPGKSIVENIITCIEKSYKSIFVLSPNFVQSEWCHYELYFAHHNLFHEGSNNLILILLEPIPQYSIPSSYHKLKSLMARRTYLEWPKEKSKRGLFWANLKAAINIKLTEQAKK

>*Pan troglodytes* Toll-like receptor 2 BAG55022.1

MPHTLWMVWVLGVIISLSKEESSNQASLSCDRNGICKGSSGSLNSIPSGLTEAVKSLDLSNNRITYISNSDLQRCVNLQALVLTSNGINTIEEDSFSSLGSLEHLDLSYNYLSNLSSSWFKPLSSLTFLNLLGNPYKTLGETSLFSHLTKLQILRVGNMDTFTKIQRKDFAGLTFLEELEIDASDLQSYEPKSLKSIQNVSHLILHMKQHILLLEIFVDVTSSVECLELRDTDLDTFRFSELSTGETNSLIKKFTFRNVKITDESLFQVMKLLNQISGLLELEFDDCTLNGVGNFRASDNDRVIDPGKVETLTIRRLHIPRFYLFYDLSTLYSLTERVKRITVENSKVFLVPCLLSQHLKSLEYLDLSENLIVEEYLKNSACEDAWPSLQTLILRQNHLASLEKTGETLLTLKNLTNVDISKNSFHSMPETCQWPEKMKYLNLSSTRIHSVTGCIPKTLEILDVSNNNLNLFSLNLPQLKELYISRNKLMTLPDASLLPMLLVLKISRNAITTFSKEQLDSFHTLKTLEAGGNNFICSCEFLSFTQEQQALAKVLIDWPANYLCDSPSHVRGQQVQDVRLSVSECHRTALVSGMCCALFLLILLTGVLCHRFHGLWYMKMMWAWLQAKRKPRKAPSRNICYDAFVSYSERDAYWVENLMVQELENFNPPFKLCLHKRDFIPGKWIIDNIIDSIEKSHKTVFVLSENFVKSEWCKYELDFSHFRLFDENNDAAILILLEPIEKKAIPQRFCKLRKIMNTKTYLEWPMDEAQREGFWVNLRAAIKS

>*Pan troglodytes* Toll-like receptor 3 JAA04352.1

MRQTLPCIYFWGGLLPFGMLCASSTTKCTVTHEVADCSHLKLTQVPDDLPTNITVLNLTHNQLRRLPASNFTRYSQLTSLDVGFNTISKLEPELCQKLPMLKVLNLQHNELSQLSDKTFAFCTNLTELHLMSNSIQKIKNNPFVKQKNLITLDLSHNGLSSTKLGTQVQLENLQELLLSNNKIQALKSEELDIFANSSLKKLELSSNQIKEFSPGCFHAIGRLFGLFLNNVQLGPSLTEKLCLELANTSIRNLSLSNSQLSTTSNTTFLGLKWTNLTMLDLSYNNLNVVGNDSFAWLPHLEYFFLDYNNIQHLFSHSLHGLFNVRYLNLKRSFTKQSISLASLPKIDDFSFQWLKCLEHLNMEDNDIPGIKSNMFTGLINLKYLSLSNSFSSLRTLTNETFVSLAHSPLHILNLTKNKISKIESDAFSWLGHLEVLDLGLNEIGQELTGQEWRGLENIFEIYLSYNKYLQLTRNSFALVPSLQRLMLRRVALKNVDSSPSPFQPLRNLTILDLSNNNIANINDDMLEGLEKLEILDLQHNNLARLWKHANPGGPVYFLKGLSHLHILNLESNGFDEIPVEVFKDLFELKIIDLGLNNLNTLPASVFNNQVSLKSLNLQKNLITSIEKKVFGPAFRNLTELDMRFNPFDCTCESIAWFVNWINETNTNIPELSSHYLCNTPPHYHGFPVRLFDTSSCKDSAPFELFFMINTSILLIFIFIVLLIHFQGWRISFYWNVSVHRVLGFKEIDRQTEQFEYAAYIIHAYKDKDWVWEHFSSMEKEDQSLKFCLEERDFEAGVFELEAIVNSIKRSRKIIFVITHHLLKDPVCKRFKVHHAVQQAIEQNLDSIILVFLEEIPDYKLNHALCLRRGMFKSHCILNWPVQKERIGAFRHKLQVALGSKNSVH

>*Pan troglodytes* Toll-like receptor 4 JAA42756.1

MMSASRLAGTLIPAMAFLSCVRPESWEPCVEVVPNITYQCMELNFYKIPDNLPFSTKNLDLSFNPLRHLGSYSFFSFPELQVLDLSRCEIQTIEDGAYQSLSHLSTLILTGNPIQSLALGAFSGLSSLQKLVAVETNLASLENFPIGHLKTLKELNVAHNLIQSFKLPEYFSNLTNLEHLDLSSNKIQSIYCTDLRVLHQMPLLNLSLDLSLNPMNFIQPGAFKEIRLHKLTLRNNFDSLNVMKTCIQGLAGLEVHRLVLGEFRNEGNLEKFDKSALEGLCNLTIEEFRLAYLDYYLDDIIDLFNCLTNVSSFSLVSVTIKSVKDFSYNFGWQHLELVNCKFGQFPTLKLKSLKRLTFTSNKGGNAFSEVDLPSLEFLDLSRNGLSFKGCCSQSDFGTTSLKYLDLSFNGVITMSSNFLGLEQLEHLDFQHSNLKQMSEFSVFLSLRNLIYLDISHTHTRVAFNGIFNGLSSLEVLKMAGNSFQENFLPDIFTELRNLTFLDLSQCQLEQLSPTAFNSLSSLQVLNMSHNNFFSLDTFPYKCLNSLQVLDYSLNHIMTSKKQELQHFPSSLAFLNLTQNDFACTCEHQSFLQWIKDQRQLLVEVERMECATPSDKQGMPVLSLNITCQMNKTIIGVSVLSVLVVSVVAVLVYKFYFHLMLLAGCIKYGRGENIYDAFVIYSSQDEDWVRNELVKNLEEGVPPFQLCLHYRDFIPGVAIAANIIHEGFHKSRKVIVVVSQHFIQSRWCIFEYEIAQTWQFLSSRAGIIFIVLQKVEKTLLRRQVELYRLLSRNTYLEWEDSVLGRHIFWRRLRKALLDGKSWNPEGTVGTGCNWQEATSI

>*Pan troglodytes* Toll-like receptor 6 BAG55050.1

MTKDKEPIVKSFHFVCLMIIIVGTRIHFSDGNEFAVDKSKRGLIHVPKDLPLKTKVLDMSQNYIAELQVSDMSFLSELKVLRLSHNRIQLLDLSVFKFNQDLEYLDLSHNQLQKISCHPIVSFRHLDLSFNDFKALPICKEFGNLSQLNFLGLSAMKLQKLDLLPIAHLHLSYILLDLRNYYIKENETESLQILNAKTLHLVFHPTSLFAIQVNISVNTLGCLQLTNIKLNDDNCQVFIKFLSELTRGPTLLNFTLNHIETTWKCLVRVFQFLWPKPVEYLNIYNLTIIESIHEEEFTYSKTTLKALKIEHITNKVFLFSQTALYTVFSEMNIMMLTISDTPFIHMLCPHAPSTFKFLNFTQNVFTDSIFEKCSTLVKLETLILQKNGLKDLFKVGLMTKDMPSLEILDVSWNSLESGRHKENCTWVESIVVLNLSSNMLTDSVFRCLPPRIKVLDLHSNKIKSVPKQVIKLKALQELNVAFNSLTDLPGCGSFSSLSVLIIDHNSVSHPSADFFQSCQKMRSIKAGDNPFQCTCELRQFVKSIDQVSSEVLEGWPDSYKCDYPESYRGTPLKDFHMSELSCNITLLIITIGATMLVLAVTVTSLCIYLDLPWYLRMVCQWTQTRRRARNIPLEELQRNLQFHAFISYSEHDSAWVKTELVPYLEKEDIQICLHERNFVPGKSIVENIINCIEKSYKSIFVLSPNFVQSEWCHYELYFAHHNLFHEGSNNLILILLEPIPQNSIPNKYHKLKALMMQRTYLQWPKEKSKRGLFWANIRAAFNMKLTLVTENNDVKS

>*Pan troglodytes* Toll-like receptor 7 BAG55057.1

MVFPMWTLKRQILILFNIILISKLLGARWFPKTLPCDVTLDVPKNHVIVDCTDKHLTEIPGGIPTNTTNLTLTINHIPDISPASFHRLDHLVEIDFRCNCVPIPLGSKNNMCIKRLQIKPRSFSGLTYLKSLYLDGNQLLEIPQGLPPSLQLLSLEANNIFSIRKENLTELANIEILYLGQNCYYRNPCYVSYSIEKDAFLNLTKLKVLSLKDNNVTAVPTVLPSTLTELYLYNNMIAKIQEDDFNNLNQLQILDLSGNCPRCYNAPFPCTPCKNNSPLQIPVNAFDALTELKVLRLHSNSLQHVPPRWFKNINKLQELDLSQNFLAKEIGDAKFLHFLPNLIQLDLSFNFELQVYRASMNLSQAFSSLKSLKILRIRGYVFKELKSFNLSPLHNLQNLEVLDLGTNFIKIANLSMFKQFKRLKVIDLSVNKISPSGDSSEVGFCSNARTSVESYEPQVLEQLHYFRYDKYARSCRFKNKEASFMSVNESCYKYGQTLDLSKNSIFFVKSSDFQHLSFLKCLNLSGNLISQTLNGSEFQPLGELRYLDFSNNRLDLLHSTAFEELHKLEVLDISSNSHYFQSEGITHMLNFTKNLKVLQKLMMNDNDISSSTSRTMESESLRTLEFRGNHLDVLWREGDNRYLQLFKNLLKLEELDISKNSLSFLPSGVFDGMPPNLKNLSLAKNGLKSFSWNKLQCLKNLETLDLSHNQLTTVPERLSNCSRSLKNLILKNNQIRSLTKYFLQDAFQLRYLDLSSNKIQMIQKTSFPENVLNNLKMLLLHHNRFLCTCDAVWFVWWVNHTEVTIPYLATDVTCVGPGAHKGQSVISLDLYTCELDLTNLILFSLSISVSLFLMVMMTASHLYFWDVWYIYHFCKAKIKGYQRLISPDCCYDAFIVYDTKDPAVTEWVLAELVAKLEDPREKHFNLCLEERDWLPGQPVLENLSQSIQLSKKTVFVMTDKYAKTENFKIAFYLSHQRLMDEKVDVIILIFLEKPFQKSKFLQLRKRLCGSSVLEWPTNPQAHPYFWQCLKNTLATDNHVAYSQVFKETV

>*Pan troglodytes* Toll-like receptor 8 BAG55064.1

MENMFLQSSMLTCIFLLISGSCELCAEENFSRSYPCDEKKQNDSVIAECSNRRLREVPQTVGKYVTELDLSDNFITHITNESFQGLQNLTKINLNHNPNVQHQNGNPGIQSNGLNITDGAFLNLKNLRELLLEDNQLPQIPSGLPESLTELSLIQNNIYNITKEGISRLINLKNLYLAWNCYFNKVCEKTNIEDGVFETLTNLELLSLSFNSLSHVPPKLPSSLRKLFLSNTQIKYISEEDFKGLINLTLLDLSGNCPRCFNAPFPCVPCDGGASINIDRFAFQNLTQLRYLNLSSTSLRKINAAWFKNMPHLKVLDLEFNYLVGEIASGAFLTMLPRLEILDLSFNYIKGSYPQHINISKNFSKLLSLRALHLRGYVFQELREDDFQPLMQLPNLSTINLGINFIKQIDFKLFQNFSNLEIIYLSENRISPLVKDTRQSYANSSSFQRHILKRRSTDFEFDPHSNFYHFTRPLIKPQCAAYGKALDLSLNSIFFIGPNQFENLPDIACLNLSANSNAQVLSGTEFSAIPHVKYLDLTNNRLDFDNASALTELSDLEVLDLSYNSHYFRIAGVTHHLEFIQNFTNLKVLNLSHNNIYTLTDKYNLESKSLVELVFSGNRLDILWNDDDNRYISIFKGLKNLTRLDLSLNRLKHIPNEAFLNLPASLTELHINDNMLKFFNWTLLQQFPRLELLDLRGNKLLFLTDSLSDFTSSLRTLLLSHNRISHLPSGFLSEVSSLKHLDLSSNLLKTINKSALETKTTTKLSMLELHGNPFECTCDIGDFRRWMDEHLNVKIPRLVDVICASPGDQRGKSIVSLELTTCVSDVTAVILFFFTFFITTMVMLAALAHHLFYWDVWFIYNVCLAKVKGYRSLSTSQTFYDAYISYDTKDASVTDWVINELRYHLEESRDKNVLLCLEERDWDPGLAIIDNLMQSINQSKKTVFVLTKKYAKSWNFKTAFYLALQRLMDENMDVIIFILLEPVLQHSQYLRLRQRICKSSILQWPDNPKAEGLFWQTLRNVVLTENDSRYNSMYVDSIKQY

>*Pan troglodytes* Toll-like receptor 10 BAG55078.1

MRLIRNIYIFCSIVMTVEGDAPELPEERELMTNCSNMSLRKVPADLTPATTTLDLSYNLLFQLQSSDFHSVSKLRVLILCHNRIQQLDLKTFEFNKELRYLDLSNNRLKSVTWYLLAGLRYLDLSFNDFDTMPICEEAGNMSHLEILGLSGAKIQKSDFQKIAHLHLNTVFLGFRTLSHYEEGSLPILNTTKLHIVLPMDTNFWVLLRDGIKTSKILEMTNIDGKSQFVSYEMQRNLSLENAKTSILLLNKVDLLWDDLFLILQFVWHTSVEHFQIRNVTFGGKAYLDHNSFDYSNTVMRTIKLEHVHFRVFYIQQDKIYLLLTKMDIENLTISNAQMPHMLFPNYPTKFQYLNFANNILTDELFKRTIQLPHLKTLILNGNKLETLSLVSCFANNTPLEHLDLSQNLLQHKNDENCSWPETVVNMNLSYNKLSDSVFRCLPKSIQILDLNNNKIQTVPKETIHLMALRELNIAFNFLTDLPGCSHFSRLSILNIEMNFILSPSLDFVQSCQEVKTLNAGRNPFRCTCELKNFIQLETYSEVMMVGWSDSYTCEYPLNLRGTRLKDVHLHELSCNTALLIVTIVVIMLVLGLAVAFCCLHFDLPWYLRMLGQCTQTWHRVRKTTQEQLKRNVRFHAFISYSEHDSLWVKNELIPNLEKEDGSILICLYESYFDPGKSISENIVSFIEKSYKSIFVLSPNFVQNEWCHYEFYFAHHNLFHENSDHIILILLEPIPFYCIPTRYHKLKALLEKKAYLEWPKDRRKCGLFWANLRAAINVNVSATREMYELQTFTELNEESRGSTISLMRTDCL

>*Marsupenaeus japonicus* Toll1 BAF99007.1

MKPLWILLPCFLVVSSIVTGVWGFGSCGKCVRGVNQLICPDLMSRERFDGTRENVFHMKCQHKAKSVNFSLINDCSFPNVEYVIFERCPKPNVTFAEVFRSSGIEPEKVLTFSFVSVFTGDPGDDLEEWHFQGLSNLTSLKLRGNNFQTLPPNILNYTPQTDLLFNPRSTILALSLNHSSRIRPNSKLCTFTKTNLLICLKGLFQNLNKLANISLWSNDIEQIGPKLFYNLPSLWSLELTSNKISNLDSGVFSSLTNAGKILLDSNMIENLPEDLFWNCTNLEFIHMSNNRLTSIPSELFKETKKVYSIEFNNNMVSSLPGNLFKGLERVGKIKMKRNALRTLPAGLFSDLSKLEVLDLQSNIIEELPPGFFDNQRIMDMLILKNNSLAELPEGIFRNCAGLQELYLSHNKLSTLQSSWFPAPVTTLRELDLGSNNISFSSFANGQEISVEKNFPLLSQASLEEISLENNRITAVPQAFSISFVNLTILNLSGNDIEFVDASDLLFKSDEVVLHLEKNKIKTVNLQHINNIAAYKIIKLFIGENPLVCDCNLYWFVRIFQGKHLDGEVPQLEIRDFKEKRRDSKEQTCSYTDDDTVAEKLKWVRSEILTCRLQECPESCKCFTRTHDSMYIVDCAYQKLRNVPQIIRLEEQNLQNYSLTLNLRNNSISNLDQLQDPEYHNLVNLTIPNNSLFSLNESTLPSSLRVLDIRGNNFTYFEESVIDYFNKTDIILSLGENPWVCDCELIDLQSFLRVQEMKVLDFHNIRCINFNETLVDVTEADLCPILLPPKVIIASTVISMFLILSGVLATVSFWEYKQGIKVWLFTHRLCLWAVVNEEYDNNKKYDAFISYSDKVEEFVNTVLVPGLESGDPKYKVCLHYRDWLPGAYIQQQINQSVEASRRTIVVLSSNFIENVWGHLEFKTAHYQALKDRHNRIIVIVLGEVPPENELDEELKLYLSTRTYLQFGDPKFWEKLRYAMPHPYDLIYKKQRKRRDTDKLELVKSDSKESK

>*Marsupenaeus japonicus* Toll2 BAG68890.1

MMSSWMVLPAFLLWGWAAGGVTHSLSCGLCEGGPDGYTCPHSESEEKYVLRPLPNQVLHVKCSNLVRDFSLIKDCNLTTFRQFEFERCPLPSVSFGEVFRRIGVPSGNVRSLSPTAGAWNASSGLQGWHLDSLTNLQTLQLVDNNFTSFPPALLTNTPKLKFFSFIGNQVDSLPHTMLASTPDLVMADLGNNGLATVPEDLFVNLTKLTNVSLWNNQLTDIQRNLFPDIPNLKFLDLRDNLLSAITNRQFRGMKILKRLNLGGNRLSSLNNDSFLDLRSLEELELHSNLLEKLPSGIFDNQRLIKKLILRNNSFSKLPGKIFQKCESLNMLDLSYNNLQYIERLQLPGPTTSLTYLDLGNNNISFSEDYISESGAQFIPYDFPISNQLKLQHIFLDNNRINHIPPSFNNLYLDLETIDLSGNLISYLEFRSIHFLSDSVKLNLKNNKIKVINLRQLQIWPKDEKCKNVTLSLEGNPLLCNCLLYIFAKIAQGKSDELSKTSFKILIDDADKVTCTSLENRKMYVKTLDFRMLTCQLEHCLGNCTCEFRRHDEMLIVDCSFKGMKEIPIPNDDIYNIKNFSVTLNLMNNNITNFDGLEHPFYSKLVNLTIPYNKISHFNESDLPEHLKVLDVRGNNLTLLSATTLDYLNVTDMTLSLGDNPWTCNCELIDFFTFLQVPERKVLDQNNIKCASDGELLLNINEYTICPSFRQPMVIVTIVLITVFLLLFAVLGTMSFYKYKQGIKVWLFTHRMCLWAITEDELDADKKYDAFISYSHKDEEFVNTVLVPGLESGDPKYRICLHYRDWIPGEYIQNQILQSVEDSRRTIVVLSSNFIESVWGQLEFKAAHSQALQDRTNRIIVIVYGQVPPESELDEKLRLYISMKTYVKWGDAKFWEKLRYIMPHPQELIQKKQQKCKNADKLELVKSNSKSV

>*Fenneropenaeus chinensis* Toll ABQ59330.1

MMRSWMVLPAFLLWGWAAGGVTLSLSCGRCEGGPDGYTCPSSDSAQAYVLRALPDQVLRVECRNNGGDFSLLKGCNFTAFRQFEFERCPLPDVSFGEVFRRIGVPSGDVKSLSLTAGSWNASSGLQEWHLDSLTNLQTLQLVDNNFTSFPRALLTNTPKLEFFRFIGNRVGNLPHTMFASTPNLVMAELGSNGLTSVPEDLFANLTKLLNVSLWNNQLTDIQRSLFSDITGLRFLDLRDNFLSDITNRQFQGMKILKRLNLGGNQISSLNKDSFGDLRNLEELELQSNWLENLPTGIFDNQRLMQKLILRNNSLSKLPDRIFQKCESLKMLDLSVNNLQYIERSQLPAHTSALTYLNLGSSNISLSEDFISDSGTHFITYDFPLSDQLELQHIFLDNNRINHIPSLFNNLFVDLKTIDLSGNLISYLDFPSIHFISDGVKLNLENNLIKTISLRKLKFFSFKEKIKNVTLSLEGNPLVCNCLLYRFTKIVQEKSELLSKSSFQILINDADKVTCTSLENRKMHVKTLDFKMLTCELELCLDNCTCSWRPHDEMFVVDCSFKDMKEIPIPSKDIYKLENYSITLNLMNNSIANFDGLDHPFYTRLANLTIPYNKISHFNKSDLPDNLKVLDVRGNNLTFLSASTLDYLNVTAMTLSLGDNPWICNCDIIDFSTFLQVPERKVLDPNNIKCASDGEELLGNNEYTMCPSFRQRMVIVTIVLITVFLLLFAVLGTMSFYKYKQGIKVWLFTHRMCLWAITEDELDADKKYDAFISYSHKDEEFVNTVLVPGLESGDPKYRICLHYRDWIPGEYIQNQIMQSVEDSRRTIVVLSSNFIESVWGQLEFKAAHSQALQDRTNRIIVIVYGQVPPESELDEKLRLYISMKTYVKWGDAKFWEKLRYIMPHPQELTQKKQQKCINADKLELVKSNSKNV

>*Penaeus monodon* Toll ADK55066.1

MMSPWMVLPAFLLWGWAAGGVTLSLSCGRCEGGPDGYTCPSSDSAQAYVLRALPDQVLRVECRNNVGDFSLLKDCNFTTFRQFEFERCPLPDVSFGEVFRRIGVPSGDVKSLSFTAGSWNASSGLQEWHLDSLTNLQTLQLVDNNSASFPPALLTNTPKLEFFRFIGNRVGSLPHTMFASTPNLVMAELGDNGLTSVPEDLFANLTKLLNVSLWNNQLTDIQRSLFSDITGLRFLDLRDNFLSDITNRQFQGMKILKRLNLGGNRISNLNKDSFGDLRSLEELELHSNWLENLPTGIFENQRLMQKLILRNNSLSKLPDRIFQKCESLKMLDLSVNNLQYIERSQLPTPKTSLTYLNLGSNNISLPEDYISDSGAQFIPYDFPLSNQLELQHIFLDNNRINHIPSSFNNLFVDLKTIDLSGNLISYLDFPPIHFISDGVKLNLKNNLIKAISLRQLKFWPIKEKIKNVTLSLEGNPLVCNCLLYIFAKIVQEKSELLSKSSFQVLIDDADKVTCISLENRKMHVKTLDFKMLTCELEQCLDNCTCSWRPHDEMFIVDCSFKDMKEIPMPSKDIYNLKNYSVTLNLMNNSIANFDGLDHPFYTKLANLTIPYNKISHINESDLPDNLKVLDVRGNNLTFLSATTLDYLNVTDMTLSLGDNPWTCNCDMIDFFTFLQVPERKVLDSNNIKCASDGEELLSINEYTICPSFRQPMVIVTIVLITVFLLLFAVLGTMSFYKYKQGIKVWLFTHRMCLWAITEDELDADKKYDAFISYSHKDEEFVNTVLVPGLESGDPKYRICLHYRDWIPGEYIQNQILQSVEDSRRTIVVLSSNFIESVWGQLEFKAAHSQALQDRTNRIIVIVYGQVPPESELDEKLRLYISMKTYVKWGDAKFWEKLRYIMPHPQELIQKKQQKCKNADKLELVKSNSKSV

>*Macrobrachium rosenbergii* Toll1 AHL39100.1

MLTENRIETLPESLFRNTRNLTKLELQNNGISSLPKSLFAGLTNLRNISLWENRLSQFQPELLLDSPKLWSLELTHNDISKLDPDVFSGLKEIRKIHISNNHLDAFPETVFHGCPNLEVLEFENNRITDLPENIFKETVNIKRLNMNRNGLTWLPEKLFSNMTNLENLKLKRNGIRSISPGLFEGLEKLQVLDLQSNVLEDLPVGIFDDLVSIEMLILQNNSLSELPDEIFANCESLQELYLSYNKLNRLLPTMFPNSTLMSILDLSNNNLSFSYSRTELSPDNKTITVQEYFPLAHLRGLKRLLLKNNQITEMPLALNTEFERLEVLDLNNNSISYLDHNDLTFRSIRPMQIDFRNNHIRVINLNHMNFSSNGEKEVSIYVADNPLICNCELYKFTLLAQGKLGDDTVLAVQDDSAVTCSNPERLNEAFYVTSIDETSLTCRLQKCVDNCTCMTRAHDKMFFVDCSYRGLKNIPSLKKELLPKDDDYGITLILRNNSITSLDGLGSREYAGLVNLTIPNNKLSVINETFLPVTLEVLNVRGNNISFLTTSMVEFLNLTDIQLSIGNNPWKCDCNTAELHKFLRDPDRKILDIHDVSCANIPNEVLISLSDEDLCPLIQQPFVIASITAVSVCLLVVAILGTVSFYTYRQDIKVWLFTHRLCLWAVAEEEKDDDKKYDAFISYSSKDEEFVNNILVPGLENEDPKYKLCLHYRDWVPGDYIQNQIHHSVEESRRTIVVLSRNFIENVWGQFEFKAAHSKALKDKTNRVIVIVLGEVPPQDELDEELRLYLTTRTYLLKTDPKFWEKLRYAMPHPPDLLTKKNKKKEKQIQKFELG

>*Macrobrachium rosenbergii* Toll2 AHL39101.1

MAAWKCLPALLLLVWVAGGISTTTTTTTCGNCKENGNYVICRHSAGGDVYEVEVLMETELLLQCKPSTTNLNLTFMEGCSFPSVEQIKVYGCPLPNASFGELFFQMGVYPENVTTFGFYDRNERDRPLEGWHLDGLEGLLTLEFVNNDFLGLPVDLFTPTPSLRRIKVVSDSFSSLPESIFFNLPDLETLNFERNRLETLPPGLFTNLTALTNVTFSHNYLSSVDPDFFSDKVNLESLDLSHNQIASLERNIFENLGALRNLSMQGNLLESLPSGVFSKLLSLEVLDLSFNVLRQLPEKAFDRLIDVKNIDLSNNSLDELPSEAFSKCESLARLSLRHNNLRKLQAEMFPRQTSLTFLDLGHNNISFTVNSAKILQEGPVSYEGNFPLNSQIRLQELLMNDNKIDFIPSAINNIFVDLKKVDLSGNEITDFSYHSLVFYQDSVILNLKNNNISYVDFHEIYDLLNEKEVKLYLEGNNLFCDCRMYLFTRIVQGQPIRKMKIMDIEVEDIEKVTCRNHDGIQQLLSQVDSTQLYCVIPDVQCIRNCSCLWRAHDSMVIIDCSFLGLQELPDLSILSKPLTKISVTLDMKNNSLYTLKGLKDPQFANLVNLTVSNNKISFINESDIPTTLKVLDVRGNELTNIPEPVLEVLNATDMILSFSGNPWDCDCDFINFLNFLHTPSRKVTDFDEITCARGNVYLHDLTEYSLCPYFMQPMVIVTLVAIGAFLLFFAVFGTVSFYKYKQGIKVWLYAHRMCLWAITEDEMDADKKFDAFISYSHKDEEFVNTVLVPGLENGDPKYRICLHYRDWVPGEYIQNQILQSVEASRRTIVVLSSNFIESVWGQLEFKAAHSQALQDGTSRIIVIVYGEIPPESELDDKLQLYISTKTYVKWGDAKFWEKLRYFMPHPPKKLNN

>*Macrobrachium rosenbergii* Toll3 AHL39102.1

MSCRATRRYGRAAAVALCRISVLASLWSYALANRYGDGGECESQALSSSDRERILTCSLKTLDEDVRVANLTSVSVENVVRLTVVCNEVYHFESVLKSTSLSGFQRVKEFNVEFCKISELDENAFVNLRNLRNLTLRTRNSDWPVMSLTANSEVFRPLHQLERLDLSMNNIWELPAGAFCHLASLKFLNLSHNHLQAITQLGFGQNAAASVDRASSSGALASCRSDISSLDLSSNDVTVLVSGSLQGQTHLQHLFLQNNELGKIDDNAFYGLGSLRTLDISDNQLVALPEDAFLHTPGLMNCRARNNSLSVLGPGLFRGLDQLVELDLSHNELKSEWLTSTIFQGLIRLMLLDLSHNKISQLNQQVFRDLYSVQVLRLSHNQLQTIPAAAFAACVNLHTLDLSYNQLTNIPDRAFQGLNVLSFLALDNNQISNVGVSSLTNLTSLEDLNLNGNQLTVIPQAIGRLRYLKTLDLGENQIASLDNMPVKGLEFLYGLRLASNKISGNVSKNTFSNIPSLKILNLAKNGITGIESNAFENNLNLQAVRIDANMLSSMNNLFENLPKLLWLNVSDNQIETFDYHFVSESLQWLDLHKNKIGQLGNFMERHDLNLQTIDASHNKLEYISSIQIPDSVQLLFLNDNKISVVEPFTFFKKVNLTRVDLYANQLSKMDMSALRLTKLPPEKSLPEFYLGGNPFVCDCNMEWLQRINGLEHERQNPKIMDLQSIYCQMPFARTGAFKALVEVNPSQFLCQYETHCFALCHCCEFDACDCEMTCPDGCGCYHDQSWRSNIVDCSMQDVQQVPDRIPMDATQVYLDGNDLKNLSSHSFIGRKHLQILYLNGSNVKSVDNETFSGLTRLIALHLQDNLLEALRGNEFQGLEIVRELYLHNNKLRYVHQHTFATLSHLEVLTLHHNHLINFPVWRLVDNPYLGRVSLSANRWSCHCQFMESFGIWLNGNERKVTDSQEIKCFADESGKQPGSSIIDFNVTTCMNTTATSTVIQPIVLDNLLHPVLATVVALIVVVSLIVCVLYRGTIRVWIYSHCGFRMCHKDTNSEDRDKLFDAFVSYSSKDEAWVNQVLAGELERGERPYRICLHYRDFPVTAYIADTIVEAVESSRRTIIVLSKNFIENEWCRFQFKSAHHEVLKKRRQRLIVIVLGEIPARDLDPDLRLYLKTNTCIYASDKYFWEKLRFAMPDVQNSQRVVHTYSSVPERSSSYTNKYSVNSPSSMHQNLRGGSDTYWA

>*Drosophila melanogaster* Toll1 or Toll AAA28941.1

MSRLKAASELALLVIILQLLQWPGSEASFGRDACSEMSIDGLCQCAPIMSEYEIICPANAENPTFRLTIQPKDYVQIMCNLTDTTDYQQLPKKLRIGEVDRVQMRRCMLPGHTPIASILDYLGIVSPTTLIFESDNLGMNITRQHLDRLHGLKRFRFTTRRLTHIPANLLTDMRNLSHLELRANIEEMPSHLFDDLENLESIEFGSNKLRQMPRGIFGKMPKLKQLNLWSNQLHNLTKHDFEGATSVLGIDIHDNGIEQLPHDVFAHLTNVTDINLSANLFRSLPQGLFDHNKHLNEVRLMNNRVPLATLPSRLFANQPELQILRLRAELQSLPGDLFEHSTQITNISLGDNLLKTLPATLLEHQVNLLSLDLSNNRLTHLPDSLFAHTTNLTDLRLEDNLLTGISGDIFSNLGNLVTLVMSRNRLRTIDSRAFVSTNGLRHLHLDHNDIDLQQPLLDIMLQTQINSPFGYMHGLLTLNLRNNSIIFVYNDWKNTMLQLRELDLSYNNISSLGYEDLAFLSQNRLHVNMTHNKIRRIALPEDVHLGEGYNNNLVHVDLNDNPLVCDCTILWFIQLVRGVHKPQYSRQFKLRTDRLVCSQPNVLEGTPVRQIEPQTLICPLDFSDDPRERKCPRGCNCHVRTYDKALVINCHSGNLTHVPRLPNLHKNMQLMELHLENNTLLRLPSANTPGYESVTSLHLAGNNLTSIDVDQLPTNLTHLDISWNHLQMLNATVLGFLNRTMKWRSVKLSGNPWMCDCTAKPLLLFTQDNFERIGDRNEMMCVNAEMPTRMVELSTNDICPAEKGVFIALAVVIALTGLLAGFTAALYYKFQTEIKIWLYAHNLLLWFVTEEDLDKDKKFDAFISYSHKDQSFIEDYLVPQLEHGPQKFQLCVHERDWLVGGHIPENIMRSVADSRRTIIVLSQNFIKSEWARLEFRAAHRSALNEGRSRIIVIIYSDIGDVEKLDEELKAYLKMNTYLKWGDPWFWDKLRFALPHRRPVGNIGNGALIKTALKGSTDDKLELIKPSPVTPPLTTPPAEATKNPLVAQLNGVTPHQAIMIANGKNGLTNLYTPNGKSHGNGHINGAFIINTNAKQSDV

>*Drosophila melanogaster* Toll2 or 18 wheeler AAF57509.1

MPATSSIITIIAVAACLLLLVADAHAQQQCNWQYGLTTMDIRCSVRALESGTGTPLDLQVAEAAGRLDLQCSQELLHASELAPGLFRQLQKLSELRIDACKLQRVPPNAFEGLMSLKRLTLESHNAVWGPGKTLELHGQSFQGLKELSELHLGDNNIRQLPEGVWCSMPSLQLLNLTQNRIRSAEFLGFSEKLCAGSALSNANGAVSGGSELQTLDVSFNELRSLPDAWGASRLRRLQTLSLQHNNISTLAPNALAGLSSLRVLNISYNHLVSLPSEAFAGNKELRELHLQGNDLYELPKGLLHRLEQLLVLDLSGNQLTSHHVDNSTFAGLIRLIVLNLSNNALTRIGSKTFKELYFLQILDMRNNSIGHIEEGAFLPLYNLHTLNLAENRLHTLDNRIFNGLYVLTKLTLNNNLVSIVESQAFRNCSDLKELDLSSNQLTEVPEAVQDLSMLKTLDLGENQISEFKNNTFRNLNQLTGLRLIDNRIGNITVGMFQDLPRLSVLNLAKNRIQSIERGAFDKNTEIEAIRLDKNFLTDINGIFATLASLLWLNLSENHLVWFDYAFIPSNLKWLDIHGNYIEALGNYYKLQEEIRVTTLDASHNRITEIGAMSVPNSIELLFINNNIIGQIQANTFVDKTRLARVDLYANVLSKISLNALRVAPVSAEKPVPEFYLGGNPFECDCSMEWLQRINNLTTRQHPHVVDLGNIECLMPHSRSAPLRPLASLSASDFVCKYESHCPPTCHCCEYEQCECEVICPGNCSCFHDATWATNIVDCGRQDLAALPNRIPQDVSDLYLDGNNMPELEVGHLTGRRNLRALYLNASNLMTLQNGSLAQLVNLRVLHLENNKLTALEGTEFRSLGLLRELYLHNNMLTHISNATFEPLVSLEVLRLDNNRLSSLPHLQYRHSLQGLTLGRNAWSCRCQQLRELAQFVSDNAMVVRDAHDIYCLDAGIKRELELIGNLANGPDCSDLLDASASNISSSQDLAGGYRLPLLAAVLVLIFLVVVLIIVFVFRESVRMWLFAHYGVRVCEPRFEDAGKLYDAIILHSEKDYEFVCRNIAAELEHGRPPFRLCIQQRDLPPQASHLQLVEGARASRKIILVLTRNLLATEWNRIEFRNAFHESLRGLAQKLVIIEETSVSAEAEDVAELSPYLKSVPSNRLLTCDRYFWEKLRYAIPIELSPRGNNYTLDHHERFKQPVSPGMIFRQAPPPPAYYCTEEMEANYSSATTATPSPRPTRPGGAARIVDSMPMPMRPPSEHIYHSIESEYSAYDQHEALSMIPTGLMHQHQQQQLRLHQQQQQQQQQRLLQPQFRAMPQQAIPAPSAPVHLRSGSGLSQASTSTQSTAQASTSAAAAQQQQQQQQQQAAGSEAANKNGQAFLV

>*Drosophila melanogaster* Toll3 AAF86229.1

SHLRSLTFIGFQIENLSTKPFAQFINLKRMVLTNCTVRNLTFLRTLQKSLEHLELDIDNEVDLKYFTNFSSLKFMKVRNYIPNKNFTALICTHKNCNFIRGINGLECPKLCQCLYIIDDLELNIDCSNLGLLQIPPLPIPIYGDVKLNFSNNSLSQLPTMTLPGYKLVKRLDVSRNRLTNLSINHLPAKLDYLDVSFNEIINMGNDVIKYLRTVPIFKQTGNQWTIHCDDKPLLNFFRHLKLIIRMKSAEMKPMFLHSLTELPKGFLKFLGKHFIWLGVRKQEYYLINEEQLLQSMHRKLNNLNTIMSIYKYMEWLHRKLIFVNREYDLFYIRQMAAPCPHKCECCYSRDSLILKIDCRNKFVYNFPDIVARNSRLMRKQNMSSPMELHLSKNNISNITIAMLPKELRFLDLRFNNLVTLDDKVLSYLKKNSIKTKLSGNPWNCDCKSRSVLSILRDHEPLEYDVTLKRCNISPTDCPDVCVCCLDNLTWPSFIVDCRGEGLLQMPSLSSRVTYVDLRNNNLTALSQKNRSSIENRSLKLHLLDNPWSCSCNDIEKINFMKSVSSSIVDFTEIKCSNGEKLVSINQHIVCPSDLFYYLALAISLVATIIALNFLIWFRQPVLVWFYEHGVCLSLAAKRELDKDKRFDAFLAFTHKDEALLEEFVDRLERGRPRFQLCFYLRDWLAGESIPDCIGQSIKDSRRIIVLMTENFMNSTWGRLEFRLALHATSRDRCKRLIVVLYPNVKNFDSLDSELRTYMAFNTYLERSHPNFWNKLIYSMPHTKLR

>*Drosophila melanogaster* Toll4 AAF52747.3

MEHSKLWDLRPEVRERRFKWTSDGQQQQQQQLGWCNNKDDPPNSHQKSKSNNDARNLNSRVRVRARVRVVPGEMRMGDINCSNGLGNIREDYCEIYLDELGENGTCSIANNEVTTEDYQMKLVFLKLEINWTSPVFHGWNIFKICNETDYELVIISVLGIRSEVDMRISPAVQYLSLLGIREISGYDIYLPSVLITEMDVHHANGPKMVTFKYLYDSTVNSVITNNYIRKTMNNTEKIKIYYHNTFEKTTLTMEKNIFHGKNKMSALIFNGLKIKGLTNNTFENLTSLNTLIFDNVFLKDLSFLRSSTLQSSLTYCIMKVDNMVDLKSFEKFTNLEIIEVSQYKGFKNFTAFICEPYKSHCKFTLGINEVACPLKCNCSYNRDKSQLEIDCWQKNLTTIPSLPVPKKGSSALVFQSNLLAELPDNSLEGYHNLKSLDVSYNQLTSLSVSQLPESLHYLDIRHNKITTLSPQVVEYLYSVNVFNQYGNKWSIYCDEYHLQEFFWYKAKLLRIKTSKFQTIMEYIELSSKGSFVENFFVQNIDQLYLEANEDEIIDAFGPSDKYFNLKLMEALNHAIWLFSGEFDEIILHHLNSPCPYRCSCCFEWHTGEFLINCRNLSLDIYPRLPNSIPYKTTLYLDRNEIRKLTNTESLVVAGHASIHKLHMSQNLLRELPLHLLPENITYLDVRNNLLKYLDDGVIAFLEYRENITKIELSGNPWECNCKAKAFLSFLRRHEPMEYETVLRRVEITDDKCPEDCICCVDTSNSDSLAYVVDCSGKELSEIPQLPTPTYGQTTLVFERNSLKKWPSSLLPGYSSVTRFYLAHNRLSDIDQLPDKLEYLDISNNNFSALDDRVRGFLQKRMNSSQLQLSLFGNPWTCRCEDKDFLVFVKEQAKNIANASAIQCIDTGRSLIEVEETDICPSVLIYYTSLAVSLLIIALSINVFICFRQPIMIWFYEHEICLSLAARRELDEDKKYDAFLSFTHKDEDLIEEFVDRLENGRHKFRLCFYLRDWLVGESIPDCINQSVKGSRRIIILMTKNFLKSTWGRLEFRLALHATSRDRCKRLIVVLYPDVEHFDDLDSELRAYMVLNTYLDRNNPNFWNKLMYSMPHASHLKRSRSDAETKV

>*Drosophila melanogaster* Toll5 AF247767_1

MLTYLPVVWLFFALLVLRSATGQIIPLPTFCLGLSPQCTCAAEGNVVRFHCPDEYAMLLEVSEPGASLYMSYYASTELQWLPRFNISSLVKIEFDAYIFWPEKFLSDLLKTLGVQTVKTIIFRDRTLETVVTRDVLNSGNGYMETSQPENITTWHFGSVPGLKKFKFFSHVPELQESIFHGFDTLRDLHLSVNVTTLPGNMLSTVNGTLKTLTIESPGIVSFGNPLLRELQQLRNLSLALIHPFHERDKQLQPHFFGSMTNLEEVRLASATSSVNRSMFKGTNKLQLIKMNGNDDLMELPGEIFLDQVNLKTLDLSCNAIVTLHEDVFKGLGNLTLLDLSKNRLTNLSSTIFAPLTSLNVLRLNKNSLTAMSPSVFQDVVSLNYIEMVNTQFYGATLLMNYEAVVCTNDEACQYKSAEWQCDPRCICWVQRSVGSLIVDCRGTSLEELPDLPRTTLLSTVLKVGNNSLTSLPTVSEHSGYANVSGLFLSDNNLTSLGSGDQLPDNLTHLDVRGNQIQSLSEEFLLFLQEPNNTMTLSLSGNPITCGCESLSLLFFVRTNPQRVRDIADIVCTKQKKSFQQMEAFELCPSYVLLISCVVGGLVIVICLLTVFYLMFQQELKIWLYNNNLCLWWVSEEELDKDKTYDAFISYSHKDEELISKLLPKLESGPHPFRLCLHDRDWLVGDCIPEQIVRTVDDSKRVIIVLSQHFIDSVWARMEFRIAYQATLQDKRKRIIIILYRELEHMNGIDSELRAYLKLNTYLKWGDPLFWSKLYYAMPHNRRVLKGQKKHAGPLI

>*Drosophila melanogaster* Toll6 AF247766_1

MIYYMLLILPVVLAQDQQHTTESLSTKHHQQQQLSHSNAIMGEAGVSNSQLMQPSTPARTLRPLTAGAGGDPSLYDAPDDCHFMPAAGLDQPEIALTCNLRTVNSEFDTTNFSVIPAEHTIALHILCNDEIMAKSRLEAQSFAHLVRLQQLSIQYCKLGRLGRQVLDGLEQLRNLTLRTHNILWPALNFEIEADAFSVTRRLERLDLSSNNIWSLPDNIFCTLSELSALNMSENRLQDVNELGFRDRSQEPTNGSTESTSTTESAKKSSSSSTSCSLDLEYLDVSHNDFVVLPANGFGTLRRLRVLSVNNNGISMIADKALSGLKNLQILNLSSNKIVALPTELFAEQAKIIQEVYLQNNSISVLNPQLFSNLDQLQALDLSMNQITSTWIDKNTFVGLIRLVLLNLSHNKLTKLEPEIFSDLYTLQILNLRHNQLENIAADTFAPMNNLHTLLLSHNKLKYLDAYALNGLYVLSLLSLDNNALIGVHPDAFRNCSALQDLNLNGNQLKTVPLALRNMRHLRTVDLGENMITVMEDSAFKGLGNLYGLRLIGNYLENITMHTFRDLPNLQILNLARNRIAVVEPGAFEMTSSIQAVRLDGNELNDINGLFSNMPSLLWLNISDNRLESFDYGHVPSTLQWLDLHKNRLSSLSNRFGLDSELKLQTLDVSFNQLQRIGPSSIPNSIELLFLNDNLITTVDPDTFMHKTNLTRVDLYANQITTLDIKSLRILPVWEHRALPEFYIGGNPFTCDCNIDWLQKINHITSRQYPRIMDLETIYCKLLNNRERAYIPLIEAEPKHFLCTYKTHCFAVCHCCEFDACDCEMTCPTNCTCFHDQTWSTNIVECSGAAYSEMPRRVPMDTSELYIDGNNFVELAGHSFLGRKNLAVLYANNSNVAHIYNTTFSGLKRLLILHLEDNHIISLEGNEFHNLENLRELYLQSNKIASIANGSFQMLRKLEVLRLDGNRLMHFEVWQLSANPYLVEISLADNQWSCECGYLARFRNYLGQSSEKIIDASRVSCIYNNATSVLREKNGTKCTLRDGVAHYMHTNEIEGLLPLLLVATCAFVAFFGLIFGLFCYRHELKIWAHSTNCLMNFCYKSPRFVDQLDKERPNDAYFAYSLQDEHFVNQILAQTLENDIGYRLCLHYRDVNINAYITDALIEAAESAKQFVLVLSKNFLYNEWSRFEYKSALHELVKRRKRVVFILYGDLPQRDIDMDMRHYLRTSTCIEWDDKKFWQKLRLALPLPNGRGNNNKRVVSGCLSGRTPSVNMYATSHEYQAGNGGVIPPPSARYADCGSNNYATINECAAAGGGRGYKPIPTSASAAAAACKFNTMNQLSKKQQRDLSVAGMAKTLEHQHHHNHQANRRSQHEYAVPSYLPSAAPAYDSVDYAKQQIRNNANCECVNLGTAKRAAGKNPASGLPSSFSSNFVPPGGASYNCKKSCSCIGDDELLCSCGGGGGIGVNLLESGTQSSATMSSSSNNSRQPELTHYESNLSLNDDEDEDHDQQKNLWA

>*Drosophila melanogaster* Toll7 AAF57514.1

MAAILLLLLGFSWSLAVESALAPKESESSASAMLGAGTGAAATVSLSGDYSSLLSNVPAASPVPANPSQPSGPANQCSWSYNGTSSVHCALRLIERQPGLDLQGADGSSQLTIQCSELYLFESTLPVAVFARLQTLEALRLDSCKLLQLPNNAFEGLATLKSLRLSTHNSEWGPTRTLELFPDSLGGLKQLTDLDLGDNNLRQLPSGFLCPVGNLQVLNLTRNRIRTAEQMGFADMNCGAGSGSAGSELQVLDASHNELRSISESWGISRLRRLQHLNLAYNNLSELSGEALAGLASLRIVNLSNNHLETLPEGLFAGSKELREIHLQQNELYELPKGLFHRLEQLLVVDLSGNQLTSNHVDNTTFAGLIRLIVLNLAHNALTRIDYRTFKELYFLQILNLRNNSIGHIEDNAFLPLYNLHTLNLAENRLHTLDDKLFNGLYVLSKLTLNNNLISVVEPAVFKNCSDLKELDLSSNQLNEVPRALQDLAMLRTLDLGENQIRTFDNQSFKNLHQLTGLRLIDNQIGNITVGMFQDLPRLSVLNLAKNRIQSIERGSFDKNFELEAIRLDRNFLADINGVFATLVSLLWLNLSENHLVWFDYAFIPSNLKWLDIHGNYIEALGNYYKLQEEIRVKTLDASHNRITEIGPMSIPNTIELLFINNNLIGNVQPNAFVDKANLARVDLYANQLSKLQLQQLRVAPVVAPKPLPEFYLGGNPFECDCTMDWLQRINNLTTRQHPRVMDMANIECVMPHARGAAVRPLSGLRPQDFLCRYESHCFALCHCCDFDACDCEMTCPSNCTCYHDQIWSTNVVDCGGQQTTELPRRVPMDSSVVYLDGNNFPVLKNHAFIGRKNLRALYVNGSQVAAIQNRTFASLASLQLLHLADNKLRTLHGYEFEQLSALRELYLQNNQLTTIENATLAPLAALELIRIDGNRLVTLPIWQMHATHFGTRLKSISLGRNQWSCRCQFLQALTSYVADNALIVQDAQDIYCMAASSGTGSAALEDSSSNSGSLEKRELDFNATGAACTDYYSGGSMLQHGIPESYIPLLAAALALLFLLVVIAMVFAFRESLRIWLFAHYGVRVFGPRCEESEKLYDAVLLHSAKDSEFVCQHLAAQLETGRPPLRVCLQHRDLAHDATHYQLLEATRVSRRVVILLTRNFLQTEWARCELRRSVHDALRGRPQKLVIIEEPEVAFEAESDIELLPYLKTSAVHRIRRSDRHFWEKLRYALPVDYPTFRGNNYTLELDHHNHERVKQPASPGLLYRQAPPPAYCGPADAVGIGAVPQVVPVNASVPAEQNYSTATTATPSPRPQRRGEQPGSGSGGNHHLHAQYYQHHGMRPPSEHIYSSIDSDYSTLDNEQHMLMMPGAPGGLAMEAAQRAQTWRPKREQLHLQQAQAGTLGSKASQAAHQQQQQQQQQQQQQPNPTAVSGQQQGPHVQAYLV

>*Drosophila melanogaster* Toll8 AF247764_1

MLATTHMLYVLIATCVIPIFGAALSKTVLYQAPDECRWSGGGEHDITLVCHLRTINSELENTNFSVIQPQNTVRLRLECNDALFFQSSLSPDSFRSLVELRDLTIEYCKLGNLTDGSFRGLQELRNLTIRTHNGDWSTMSLEMASNSFVEFRQLERLDLSLNNIWLIPDGMVCPLKSLQHLNASYNKIQDISNFYFSASLSSRKARVCGSTLQSLDLSANKMVSLPTAMLSALGRLTHLNMAKNSMSFLADRAFEGLLSLRVVDLSANRLTSLPPELFAETKQLQEIYLRNNSINVLAPGIFGELAELLVLDLASNELNSQWINAATFVGLKRLMMLDLSANKISRLEAHIFRPLASLQILKLEDNYIDQLPGGIFADLTNLHTLILSRNRISVIEQRTLQGLKNLLVLSLDFNRISRMDQRSLVNCSQLQDLHLNDNKLQAVPEALAHVQLLKTLDVGENMISQIENTSITQLESLYGLRMTENSLTHIRRGVFDRMSSLQILNLSQNKLKSIEAGSLQRNSQLQAIRLDGNQLKSIAGLFTELPNLVWLNISGNRLEKFDYSHIPIGLQWLDVRANRITQLGNYFEIESELSLSTFDASYNLLTEITASSIPNSVEVLYLNDNQISKIQPYTFFKKPNLTRVDLVRNRLTTLEPNALRLSPIAEDREIPEFYIGHNAYECDCNLDWLQKVNRESRTQPQLMDLDQIHCRLAYARGSSHVSLIEAKSDDFLCKYASHCFALCHCCDFQACDCKMECPDRCSCYHDQSWTSNVVDCSRASYEQTLPSHIPMDSTQLYLDGNNFRELQSHAFIGRKRLKVLHLNHSRIEVLHNRTFYGLLELEVLQLQSNQLKALNGNEFQGLDNLQELYLQHNAIATIDTLTFTHLYHLKILRLDHNAITSFAVWNFLPSYLNELRLASNPWTCSCEFIDKLRDYINRHEYVVDKLKMKCDVISGNSTQQMVIYPGSGEPASLPVVQCSQTLPLGLDNNFNYAEQAGGENASNATSTKMILNQPPKLDYIPILVAILTAFIFVMICISLVFIFRQEMRVWCHSRFGVRLFYNAQKDVDKNEREKLFDAFVSYSSKDELFVNEELAPMLEMGEHRYKLCLHQRDFPVGGYLPETIVQAIDSSRRTIMVVSENFIKSEWCRFEFKSAHQSVLRDRRRRLIVIVLGEVPQKELDPDLRLYLKTNTYLQWGDKLFWQKLRFALPDVSSSQRSNVAGQSCHVPINHASYHHHHHVHQQAMPLPHSVHHHQQQFMLPPPPQQPGSFRRQPSLHQQQQQQQQIRGNNNTTQQQQQQQAALLMGGGSVGGPAPQMIPLAGGIQQQSLPLPPNQQPTPASRNLHM

>*Drosophila melanogaster* Toll9 AAF51581.1

MCPKYIWDVIVLVCLFLGNVREAYTEFSIQDGLIIEPDSATTSSEEAEEVSKERTDLKSLMLKYESDDGNSCLLDLIKDEVIWWQFPNGTLRDSTKKYAHKLYLDLSHGNLKDDSDLFREAKLSRKVTIWRTEVFSAAFNTLTAAPFRTLYSMRESLKLLSLRGNNFAELIPDAEDFARFVNESRLEASNSVPHHCELLLLHNTTDLYDRECYLYFNNNTNMGQSITTGRNYTNFIKVLKDRFDQHGSSSQSIAWATFPKMPRLVELDISNCSIEYVSKEAFRNVSNLRRLFMSDNKIMTISHDTFYYVQGVQYLDLSFTNFLTYSYQLQLPTLEMALSLIYGLKIQQNVFKYLPELIYLDLSHSKMTRNSAVAFAHLGDKLKFLSLCYTAIPMVSSTIFKNTVLEGLDLSGNPYLSYNIIDDAFDGIANTLKYLYFERSNIKDLEWSKSLKNLQVLGLAGNNINALTPAMFQSLESLEILDLSSNHVGNWYRSAFHNNSALRVLNLRSNTINMLSNEMLKDFERLDYLSLGDNDFICDCHLRAVVEVAAANNKDADCSYRLLNYSQNAVGEEVISLAESLIIDRKLWQSRYIPWLQRSYSNIREFNRANHIIKLRFSSEDYMVAKCSAAQPYHLGDLDGDLTLKFQLLDYEASQYYCFNNTDQLQVDELNCQIRSMSDLAEELHHVTNTVIAVMGSLVGACILGFIIYLKRWHIHYYYSSLKSAALLSSASKESVNKFTNISQRDPSAVYDIFISYCQNDRTWVLNELLPNVEETGDVSICLHERDFQIGVTILDNIISCMDRSYSLMLIISSKFLLSHWCQFEMYLAQHRIFEVSKEHLILVFLEDIPRRKRPKTLQYLMDVKTYIKWPTAKEDRKLFWKRLKRSLEVIGINSREISV

>*Homo sapiens* TLR1 AAY85643.1

MTSIFHFAIIFMLILQIRIQLSEESEFLVDRSKNGLIHVPKDLSQKTTILNISQNYMSELWTSDILSLSKLRILIISHNRIQYLDISVFKFNQELEYLDLSHNKLVKISCHPTVNLKHLDLSFNAFDALPICKEFGNMSQLKFLGLSTTHLEKSSVLPIAHLNISKVLLVLGETYGEKEDPEGLQDFNTESLHIVFPTNKEFHFILDVSVKTVANLELSNIKCVLEDNKCSYFLSILAKLQTNPKLSSLTLNNIETTWNSFIRILQLVWHTTVWYFSISNVKLQGQLDFRDFDYSGTSLKALSIHQVVSDVFGFPQSYIYEIFSNMNIKNFTVSGTRMVHMLCPSKISPFLHLDFSNNLLTDTVFENCGHLTELETLILQMNQLKELSKIAEMTTQMKSLQQLDISQNSVSYDEKKGDCSWTKSLLSLNMSSNILTDTIFRCLPPRIKVLDLHSNKIKSIPKQVVKLEALQELNVAFNSLTDLPGCGSFSSLSVLIIDHNSVSHPSADFFQSCQKMRSIKAGDNPFQCTCELGEFVKNIDQVSSEVLEGWPDSYKCDYPESYRGTLLKDFHMSELSCNITLLIVTIVATMLVLAVTVTSLCIYLDLPWYLRMVCQWTQTRRRARNIPLEELQRNLQFHAFISYSGHDSFWVKNELLPNLEKEGMQICLHERNFVPGKSIVENIITCIEKSYKSIFVLSPNFVQSEWCHYELYFAHHNLFHEGSNSLILILLEPIPQYSIPSSYHKLKSLMARRTYLEWPKEKSKRGLFWANLRAAINIKLTEQAKK

>*Homo sapiens* TLR2 AAC34377.1

MPHTLWMVWVLGVIISLSKEESSNQASLSCDRNGICKGSSGSLNSIPSGLTEAVKSLDLSNNRITYISNSDLQRCVNLQALVLTSNGINTIEEDSFSSLGSLEHLDLSYNYLSNLSSSWFKPLSSLTFLNLLGNPYKTLGETSLFSHLTKLQILRVGNMDTFTKIQRKDFAGLTFLEELEIDASDLQSYEPKSLKSIQNVSHLILHMKQHILLLEIFVDVTSSVECLELRDTDLDTFHFSELSTGETNSLIKKFTFRNVKITDESLFQVMKLLNQISGLLELEFDDCTLNGVGNFRASDNDRVIDPGKVETLTIRRLHIPRFYLFYDLSTLYSLTERVKRITVENSKVFLVPCLLSQHLKSLEYLDLSENLMVEEYLKNSACEDAWPSLQTLILRQNHLASLEKTGETLLTLKNLTNIDISKNSFHSMPETCQWPEKMKYLNLSSTRIHSVTGCIPKTLEILDVSNNNLNLFSLNLPQLKELYISRNKLMTLPDASLLPMLLVLKISRNAITTFSKEQLDSFHTLKTLEAGGNNFICSCEFLSFTQEQQALAKVLIDWPANYLCDSPSHVRGQQVQDVRLSVSECHRTALVSGMCCALFLLILLTGVLCHRFHGLWYMKMMWAWLQAKRKPRKAPSRNICYDAFVSYSERDAYWVENLMVQELENFNPPFKLCLHKRDFIPGKWIIDNIIDSIEKSHKTVFVLSENFVKSEWCKYELDFSHFRLFDENNDAAILILLEPIEKKAIPQRFCKLRKIMNTKTYLEWPMDEAQREGFWVNLRAAIKS

>*Homo sapiens* TLR3 AAC34134.1

MRQTLPCIYFWGGLLPFGMLCASSTTKCTVSHEVADCSHLKLTQVPDDLPTNITVLNLTHNQLRRLPAANFTRYSQLTSLDVGFNTISKLEPELCQKLPMLKVLNLQHNELSQLSDKTFAFCTNLTELHLMSNSIQKIKNNPFVKQKNLITLDLSHNGLSSTKLGTQVQLENLQELLLSNNKIQALKSEELDIFANSSLKKLELSSNQIKEFSPGCFHAIGRLFGLFLNNVQLGPSLTEKLCLELANTSIRNLSLSNSQLSTTSNTTFLGLKWTNLTMLDLSYNNLNVVGNDSFAWLPQLEYFFLEYNNIQHLFSHSLHGLFNVRYLNLKRSFTKQSISLASLPKIDDFSFQWLKCLEHLNMEDNDIPGIKSNMFTGLINLKYLSLSNSFTSLRTLTNETFVSLAHSPLHILNLTKNKISKIESDAFSWLGHLEVLDLGLNEIGQELTGQEWRGLENIFEIYLSYNKYLQLTRNSFALVPSLQRLMLRRVALKNVDSSPSPFQPLRNLTILDLSNNNIANINDDMLEGLEKLEILDLQHNNLARLWKHANPGGPIYFLKGLSHLHILNLESNGFDEIPVEVFKDLFELKIIDLGLNNLNTLPASVFNNQVSLKSLNLQKNLITSVEKKVFGPAFRNLTELDMRFNPFDCTCESIAWFVNWINETHTNIPELSSHYLCNTPPHYHGFPVRLFDTSSCKDSAPFELFFMINTSILLIFIFIVLLIHFEGWRISFYWNVSVHRVLGFKEIDRQTEQFEYAAYIIHAYKDKDWVWEHFSSMEKEDQSLKFCLEERDFEAGVFELEAIVNSIKRSRKIIFVITHHLLKDPLCKRFKVHHAVQQAIEQNLDSIILVFLEEIPDYKLNHALCLRRGMFKSHCILNWPVQKERIGAFRHKLQVALGSKNSVH

>*Homo sapiens* TLR4 AAY82270.1

MMSASRLAGTLIPAMAFLSCVRPESWEPCVEVVPNITYQCMELNFYKIPDNLPFSTKNLDLSFNPLRHLGSYSFFSFPELQVLDLSRCEIQTIEDGAYQSLSHLSTLILTGNPIQSLALGAFSGLSSLQKLVAVETNLASLENFPIGHLKTLKELNVAHNLIQSFKLPEYFSNLTNLEHLDLSSNKIQSIYCTDLRVLHQMPLLNLSLDLSLNPMNFIQPGAFKEIRLHKLTLRNNFDSLNVMKTCIQGLAGLEVHRLVLGEFRNEGNLEKFDKSALEGLYNLTIEEFRLAYLDYYLDDIIDLFNCLTNVSSFSLVSVTIERVKDFSYNFGWQHLELVNCKFGQFPTLKLKSLKRLTFTSNKGGNAFSEVDLPSLEFLDLSRNGLSFKGCCSQSDFGTTSLKYLDLSFNGVITMSSNFLGLEQLEHLDFQHSNLKQMSEFSVFLSLRNLIYLDISHTHTRVAFNGIFNGLSSLEVLKMAGNSFQENFLPDIFTELRNLTFLDLSQCQLEQLSPTAFNSLSSLQVLNMSHNNFFSLDTFPYKCLNSLQVLDYSLNHIMTSKKQELQHFPSSLAFLNLTQNDFACTCEHQSFLQWIKDQRQLLVEVERMECATPSDKQGMPVLSLNITCQMNKTIIGVSVLSVLVVSVVAVLVYKFYFHLMLLAGCIKYGRGENIYDAFVIYSSQDEDWVRNELVKNLEEGVPPFQLCLHYRDFIPGVAIAANIIHEGFHKSRKVIVVVSQHFIQSRWCIFEYEIAQTWQFLSSRAGIIFIVLQKVEKTLLRQQVELYRLLSRNTYLEWEDSVLGRHIFWRRLRKALLDGKSWNPEGTVGTGCNWQEATSI

>*Homo sapiens* TLR5 ACM69030.1

MGDHLDLLLGVVLMAGPVFGIPSCSFDGRIAFYRFCNLTQVPQVLNTTERLLLSFNYIRTVTASSFPFLEQLQLLELGSQYTPLTIDKEAFRNLPNLRILDLGSSKIYFLHPDAFQGLFHLFELRLYFCGLSDAVLKDGYFRNLKALTRLDLSKNQIRSLYLHPSFGKLNSLKSIDFSSNQIFLVCEHELEPLQGKTLSFFSLAANSLYSRVSVDWGKCMNPFRNMVLEILDVSGNGWTVDITGNFSNAISKSQAFSLILAHHIMGAGFGFHNIKDPDQNTFAGLARSSVRHLDLSHGFVFSLNSRVFETLKDLKVLNLAYNKINKIADEAFYGLDNLQVLNLSYNLLGELYSSNFYGLPKVAYIDLQKNHIAIIQDQTFKFLEKLQTLDLRDNALTTIHFIPSIPDIFLSGNKLVTLPKINLTANLIHLSENRLENLDILYFLLRVPHLQILILNQNRFSSCSGDQTPSENPSLEQLFLGENMLQLAWETELCWDVFEGLSHLQVLYLNHNYLNSLPPGVFSHLTALRGLSLNSNRLTVLSHNDLPANLEILDISRNQLLAPNPDVFVSLSVLDITHNKFICECELSTFINWLNHTNVTIAGPPADIYCVYPDSFSGVSLFSLSTEGCDEEEVLKSLKFSLFIVCTVTLTLFLMTILTVTKFRGFCFICYKTAQRLVFKDHPQGTEPDMYKYDAYLCFSSKDFTWVQNALLKHLDTQYSDQNRFNLCFEERDFVPGENRIANIQDAIWNSRKIVCLVSRHFLRDGWCLEAFSYAQGRCLSDLNSALIMVVVGSLSQYQLMKHQSIRGFVQKQQYLRWPEDLQDVGWFLHKLSQQILKKEKEKKKDNNIPLQTVATIS

>*Homo sapiens* TLR6 EAW92902.1

MTKDKEPIVKSFHFVCLMIIIVGTRIQFSDGNEFAVDKSKRGLIHVPKDLPLKTKVLDMSQNYIAELQVSDMSFLSELTVLRLSHNRIQLLDLSVFKFNQDLEYLDLSHNQLQKISCHPIVSFRHLDLSFNDFKALPICKEFGNLSQLNFLGLSAMKLQKLDLLPIAHLHLSYILLDLRNYYIKENETESLQILNAKTLHLVFHPTSLFAIQVNISVNTLGCLQLTNIKLNDDNCQVFIKFLSELTRGSTLLNFTLNHIETTWKCLVRVFQFLWPKPVEYLNIYNLTIIESIREEDFTYSKTTLKALTIEHITNQVFLFSQTALYTVFSEMNIMMLTISDTPFIHMLCPHAPSTFKFLNFTQNVFTDSIFEKCSTLVKLETLILQKNGLKDLFKVGLMTKDMPSLEILDVSWNSLESGRHKENCTWVESIVVLNLSSNMLTDSVFRCLPPRIKVLDLHSNKIKSVPKQVVKLEALQELNVAFNSLTDLPGCGSFSSLSVLIIDHNSVSHPSADFFQSCQKMRSIKAGDNPFQCTCELREFVKNIDQVSSEVLEGWPDSYKCDYPESYRGSPLKDFHMSELSCNITLLIVTIGATMLVLAVTVTSLCIYLDLPWYLRMVCQWTQTRRRARNIPLEELQRNLQFHAFISYSEHDSAWVKSELVPYLEKEDIQICLHERNFVPGKSIVENIINCIEKSYKSIFVLSPNFVQSEWCHYELYFAHHNLFHEGSNNLILILLEPIPQNSIPNKYHKLKALMTQRTYLQWPKEKSKRGLFWANIRAAFNMKLTLVTENNDVKS

>*Homo sapiens* TLR7 NP_057646.1

MVFPMWTLKRQILILFNIILISKLLGARWFPKTLPCDVTLDVPKNHVIVDCTDKHLTEIPGGIPTNTTNLTLTINHIPDISPASFHRLDHLVEIDFRCNCVPIPLGSKNNMCIKRLQIKPRSFSGLTYLKSLYLDGNQLLEIPQGLPPSLQLLSLEANNIFSIRKENLTELANIEILYLGQNCYYRNPCYVSYSIEKDAFLNLTKLKVLSLKDNNVTAVPTVLPSTLTELYLYNNMIAKIQEDDFNNLNQLQILDLSGNCPRCYNAPFPCAPCKNNSPLQIPVNAFDALTELKVLRLHSNSLQHVPPRWFKNINKLQELDLSQNFLAKEIGDAKFLHFLPSLIQLDLSFNFELQVYRASMNLSQAFSSLKSLKILRIRGYVFKELKSFNLSPLHNLQNLEVLDLGTNFIKIANLSMFKQFKRLKVIDLSVNKISPSGDSSEVGFCSNARTSVESYEPQVLEQLHYFRYDKYARSCRFKNKEASFMSVNESCYKYGQTLDLSKNSIFFVKSSDFQHLSFLKCLNLSGNLISQTLNGSEFQPLAELRYLDFSNNRLDLLHSTAFEELHKLEVLDISSNSHYFQSEGITHMLNFTKNLKVLQKLMMNDNDISSSTSRTMESESLRTLEFRGNHLDVLWREGDNRYLQLFKNLLKLEELDISKNSLSFLPSGVFDGMPPNLKNLSLAKNGLKSFSWKKLQCLKNLETLDLSHNQLTTVPERLSNCSRSLKNLILKNNQIRSLTKYFLQDAFQLRYLDLSSNKIQMIQKTSFPENVLNNLKMLLLHHNRFLCTCDAVWFVWWVNHTEVTIPYLATDVTCVGPGAHKGQSVISLDLYTCELDLTNLILFSLSISVSLFLMVMMTASHLYFWDVWYIYHFCKAKIKGYQRLISPDCCYDAFIVYDTKDPAVTEWVLAELVAKLEDPREKHFNLCLEERDWLPGQPVLENLSQSIQLSKKTVFVMTDKYAKTENFKIAFYLSHQRLMDEKVDVIILIFLEKPFQKSKFLQLRKRLCGSSVLEWPTNPQAHPYFWQCLKNALATDNHVAYSQVFKETV

>*Homo sapiens* TLR8 AAI01078.1

MENMFLQSSMLTCIFLLISGSCELCAEENFSRSYPCDEKKQNDSVIAECSNRRLQEVPQTVGKYVTELDLSDNFITHITNESFQGLQNLTKINLNHNPNVQHQNGNPGIQSNGLNITDGAFLNLKNLRELLLEDNQLPQIPSGLPESLTELSLIQNNIYNITKEGISRLINLKNLYLAWNCYFNKVCEKTNIEDGVFETLTNLELLSLSFNSLSHVPPKLPSSLRKLFLSNTQIKYISEEDFKGLINLTLLDLSGNCPRCFNAPFPCVPCDGGASINIDRFAFQNLTQLRYLNLSSTSLRKINAAWFKNMPHLKVLDLEFNYLVGEIASGAFLTMLPRLEILDLSFNYIKGSYPQHINISRNFSKLLSLRALHLRGYVFQELREDDFQPLMQLPNLSTINLGINFIKQIDFKLFQNFSNLEIIYLSENRISPLVKDTRQSYANSSSFQRHIRKRRSTDFEFDPHSNFYHFTRPLIKPQCAAYGKALDLSLNSIFFIGPNQFENLPDIACLNLSANSNAQVLSGTEFSAIPHVKYLDLTNNRLDFDNASALTELSDLEVLDLSYNSHYFRIAGVTHHLEFIQNFTNLKVLNLSHNNIYTLTDKYNLESKSLVELVFSGNRLDILWNDDDNRYISIFKGLKNLTRLDLSLNRLKHIPNEAFLNLPASLTELHINDNMLKFFNWTLLQQFPRLELLDLRGNKLLFLTDSLSDFTSSLRTLLLSHNRISHLPSGFLSEVSSLKHLDLSSNLLKTINKSALETKTTTKLSMLELHGNPFECTCDIGDFRRWMDEHLNVKIPRLVDVICASPGDQRGKSIVSLELTTCVSDVTAVILFFFTFFITTMVMLAALAHHLFYWDVWFIYNVCLAKVKGYRSLSTSQTFYDAYISYDTKDASVTDWVINELRYHLEESRDKNVLLCLEERDWDPGLAIIDNLMQSINQSKKTVFVLTKKYAKSWNFKTAFYLALQRLMDENMDVIIFILLEPVLQHSQYLRLRQRICKSSILQWPDNPKAEGLFWQTLRNVVLTENDSRYNNMYVDSIKQY

>*Homo sapiens* TLR9 AF259262_1

MGFCRSALHPLSLLVQAIMLAMTLALGTLPAFLPCELQPHGLVNCNWLFLKSVPHFSMAAPRGNVTSLSLSSNRIHHLHDSDFAHLPSLRHLNLKWNCPPVGLSPMHFPCHMTIEPSTFLAVPTLEELNLSYNNIMTVPALPKSLISLSLSHTNILMLDSASLAGLHALRFLFMDGNCYYKNPCRQALEVAPGALLGLGNLTHLSLKYNNLTVVPRNLPSSLEYLLLSYNRIVKLAPEDLANLTALRVLDVGGNCRRCDHAPNPCMECPRHFPQLHPDTFSHLSRLEGLVLKDSSLSWLNASWFRGLGNLRVLDLSENFLYKCITKTKAFQGLTQLRKLNLSFNYQKRVSFAHLSLAPSFGSLVALKELDMHGIFFRSLDETTLRPLARLPMLQTLRLQMNFINQAQLGIFRAFPGLRYVDLSDNRISGASELTATMGEADGGEKVWLQPGDLAPAPVDTPSSEDFRPNCSTLNFTLDLSRNNLVTVQPEMFAQLSHLQCLRLSHNCISQAVNGSQFLPLTGLQVLDLSHNKLDLYHEHSFTELPRLEALDLSYNSQPFGMQGVGHNFSFVAHLRTLRHLSLAHNNIHSQVSQQLCSTSLRALDFSGNALGHMWAEGDLYLHFFQGLSGLIWLDLSQNRLHTLLPQTLRNLPKSLQVLRLRDNYLAFFKWWSLHFLPKLEVLDLAGNQLKALTNGSLPAGTRLRRLDVSCNSISFVAPGFFSKAKELRELNLSANALKTVDHSWFGPLASALQILDVSANPLHCACGAAFMDFLLEVQAAVPGLPSRVKCGSPGQLQGLSIFAQDLRLCLDEALSWDCFALSLLAVALGLGVPMLHHLCGWDLWYCFHLCLAWLPWRGRQSGRDEDALPYDAFVVFDKTQSAVADWVYNELRGQLEECRGRWALRLCLEERDWLPGKTLFENLWASVYGSRKTLFVLAHTDRVSGLLRASFLLAQQRLLEDRKDVVVLVILSPDGRRSRYVRLRQRLCRQSVLLWPHQPSGQRSFWAQLGMALTRDNHHFYNRNFCQGPTAE

>*Homo sapiens* TLR10 AAY78485.1

MRLIRNIYIFCSIVMTAEGDAPELPEERELMTNCSNMSLRKVPADLTPATTTLDLSYNILFQLQSSDFHSVSKLRVLILCHNRIQQLDLKTFEFNKELRYLDLSNNRLKSVTWYLLAGLRYLDLSFNDFDTMPICEEAGNMSHLEILGLSGAKIQKSDFQKIAHLHLNTVFLGFRTLPHYEEGSLPILNTTKLHIVLPMDTNFWVLLRDGIKTSKILEMTNIDGKSQFVSYEMQRNLSLENAKTSVLLLNKVDLLWDDLFLILQFVWHTSVEHFQIRNVTFGGKAYLDHNSFDYSNTVMRTIKLEHVHFRVFYIQQDKIYLLLTKMDIENLTISNAQMPHMLFPNYPTKFQYLNFANNILTDELFKRTIQLPHLKTLILNGNKLETLSLVSCFANNTPLEHLDLSQNLLQHKNDENCSWPETVVNMNLSYNKLSDSVFRCLPKSIQILDLNNNQIQTVPKETIHLMALRELNIAFNFLTDLPGCSHFSRLSVLNIEMNFILSPSLDFVQSCQEVKTLNAGRNPFRCTCELKNFIQLETYSEVMMVGWSDSYTCEYPLNLRGTRLKDVHLHELSCNTALLIVTIVVIMLVLGLAVAFCCLHFDLPWYLRMLGQCTQTWHRVRKTTQEQLKRNVRFHAFISYSEHDSLWVKNELIPNLEKEDGSILICLYESYFDPGKSISENIVSFIEKSYKSIFVLSPNFVQNEWCHYEFYFAHHNLFHENSDHIILILLEPIPFYCIPTRYHKLKALLEKKAYLEWPKDRRKCGLFWANLRAAINVNVLATREMYELQTFTELNEESRGSTISLMRTDCL

>*Mus musculus* TLR1 NP_001263374.1

MTKPNSLIFYCIIVLGLTLMKIQLSEECELIIKRPNANLTRVPKDLPLQTTTLDLSQNNISELQTSDILSLSKLRVLIMSYNRLQYLNISVFKFNTELEYLDLSHNELKVILCHPTVSLKHLDLSFNAFDALPICKEFGNMSQLQFLGLSGSRVQSSSVQLIAHLNISKVLLVLGDAYGEKEDPESLRHVSTETLHIVFPSKREFRFLLDVSVSTTIGLELSNIKCVLEDQGCSYFLRALSKLGKNLKLSNLTLNNVETTWNSFINILQIVWHTPVKYFSISNVKLQGQLAFRMFNYSDTSLKALSIHQVVTDVFSFPQSYIYSIFANMNIQNFTMSGTHMVHMLCPSQVSPFLHVDFTDNLLTDMVFKDCRNLVRLKTLSLQKNQLKNLENIILTSAKMTSLQKLDISQNSLRYSDGGIPCAWTQSLLVLNLSSNMLTGSVFRCLPPKVKVLDLHNNRIMSIPKDVTHLQALQELNVASNSLTDLPGCGAFSSLSVLVIDHNSVSHPSEDFFQSCQNIRSLTAGNNPFQCTCELRDFVKNIGWVAREVVEGWPDSYRCDYPESSRGTALRDFHMSPLSCDTVLLTVTIGATMLVLAVTGAFLCLYFDLPWYVRMLCQWTQTRHRARHIPLEELQRNLQFHAFVSYSGHDSAWVKNELLPNLEKDDIQICLHERNFVPGKSIVENIINFIEKSYKSIFVLSPHFIQSEWCHYELYFAHHNLFHEGSDNLILILLAPIPQYSIPTNYHKLKTLMSRRTYLEWPTEKNKHGLFWANLRASINVKLVNQAEGTCYTQQ

>*Mus musculus* TLR2 NP_036035.3

MLRALWLFWILVAITVLFSKRCSAQESLSCDASGVCDGRSRSFTSIPSGLTAAMKSLDLSFNKITYIGHGDLRACANLQVLMLKSSRINTIEGDAFYSLGSLEHLDLSDNHLSSLSSSWFGPLSSLKYLNLMGNPYQTLGVTSLFPNLTNLQTLRIGNVETFSEIRRIDFAGLTSLNELEIKALSLRNYQSQSLKSIRDIHHLTLHLSESAFLLEIFADILSSVRYLELRDTNLARFQFSPLPVDEVSSPMKKLAFRGSVLTDESFNELLKLLRYILELSEVEFDDCTLNGLGDFNPSESDVVSELGKVETVTIRRLHIPQFYLFYDLSTVYSLLEKVKRITVENSKVFLVPCSFSQHLKSLEFLDLSENLMVEEYLKNSACKGAWPSLQTLVLSQNHLRSMQKTGEILLTLKNLTSLDISRNTFHPMPDSCQWPEKMRFLNLSSTGIRVVKTCIPQTLEVLDVSNNNLDSFSLFLPRLQELYISRNKLKTLPDASLFPVLLVMKIRENAVSTFSKDQLGSFPKLETLEAGDNHFVCSCELLSFTMETPALAQILVDWPDSYLCDSPPRLHGHRLQDARPSVLECHQAALVSGVCCALLLLILLVGALCHHFHGLWYLRMMWAWLQAKRKPKKAPCRDVCYDAFVSYSEQDSHWVENLMVQQLENSDPPFKLCLHKRDFVPGKWIIDNIIDSIEKSHKTVFVLSENFVRSEWCKYELDFSHFRLFDENNDAAILVLLEPIERKAIPQRFCKLRKIMNTKTYLEWPLDEGQQEVFWVNLRTAIKS

>*Mus musculus* TLR3 NP_569054.2

MKGCSSYLMYSFGGLLSLWILLVSSTNQCTVRYNVADCSHLKLTHIPDDLPSNITVLNLTHNQLRRLPPTNFTRYSQLAILDAGFNSISKLEPELCQILPLLKVLNLQHNELSQISDQTFVFCTNLTELDLMSNSIHKIKSNPFKNQKNLIKLDLSHNGLSSTKLGTGVQLENLQELLLAKNKILALRSEELEFLGNSSLRKLDLSSNPLKEFSPGCFQTIGKLFALLLNNAQLNPHLTEKLCWELSNTSIQNLSLANNQLLATSESTFSGLKWTNLTQLDLSYNNLHDVGNGSFSYLPSLRYLSLEYNNIQRLSPRSFYGLSNLRYLSLKRAFTKQSVSLASHPNIDDFSFQWLKYLEYLNMDDNNIPSTKSNTFTGLVSLKYLSLSKTFTSLQTLTNETFVSLAHSPLLTLNLTKNHISKIANGTFSWLGQLRILDLGLNEIEQKLSGQEWRGLRNIFEIYLSYNKYLQLSTSSFALVPSLQRLMLRRVALKNVDISPSPFRPLRNLTILDLSNNNIANINEDLLEGLENLEILDFQHNNLARLWKRANPGGPVNFLKGLSHLHILNLESNGLDEIPVGVFKNLFELKSINLGLNNLNKLEPFIFDDQTSLRSLNLQKNLITSVEKDVFGPPFQNLNSLDMRFNPFDCTCESISWFVNWINQTHTNISELSTHYLCNTPHHYYGFPLKLFDTSSCKDSAPFELLFIISTSMLLVFILVVLLIHIEGWRISFYWNVSVHRILGFKEIDTQAEQFEYTAYIIHAHKDRDWVWEHFSPMEEQDQSLKFCLEERDFEAGVLGLEAIVNSIKRSRKIIFVITHHLLKDPLCRRFKVHHAVQQAIEQNLDSIILIFLQNIPDYKLNHALCLRRGMFKSHCILNWPVQKERINAFHHKLQVALGSRNSAH

>*Mus musculus* TLR4 NP_067272.1

MMPPWLLARTLIMALFFSCLTPGSLNPCIEVVPNITYQCMDQKLSKVPDDIPSSTKNIDLSFNPLKILKSYSFSNFSELQWLDLSRCEIETIEDKAWHGLHHLSNLILTGNPIQSFSPGSFSGLTSLENLVAVETKLASLESFPIGQLITLKKLNVAHNFIHSCKLPAYFSNLTNLVHVDLSYNYIQTITVNDLQFLRENPQVNLSLDMSLNPIDFIQDQAFQGIKLHELTLRGNFNSSNIMKTCLQNLAGLHVHRLILGEFKDERNLEIFEPSIMEGLCDVTIDEFRLTYTNDFSDDIVKFHCLANVSAMSLAGVSIKYLEDVPKHFKWQSLSIIRCQLKQFPTLDLPFLKSLTLTMNKGSISFKKVALPSLSYLDLSRNALSFSGCCSYSDLGTNSLRHLDLSFNGAIIMSANFMGLEELQHLDFQHSTLKRVTEFSAFLSLEKLLYLDISYTNTKIDFDGIFLGLTSLNTLKMAGNSFKDNTLSNVFANTTNLTFLDLSKCQLEQISWGVFDTLHRLQLLNMSHNNLLFLDSSHYNQLYSLSTLDCSFNRIETSKGILQHFPKSLAFFNLTNNSVACICEHQKFLQWVKEQKQFLVNVEQMTCATPVEMNTSLVLDFNNSTCYMYKTIISVSVVSVIVVSTVAFLIYHFYFHLILIAGCKKYSRGESIYDAFVIYSSQNEDWVRNELVKNLEEGVPRFHLCLHYRDFIPGVAIAANIIQEGFHKSRKVIVVVSRHFIQSRWCIFEYEIAQTWQFLSSRSGIIFIVLEKVEKSLLRQQVELYRLLSRNTYLEWEDNPLGRHIFWRRLKNALLDGKASNPEQTAEEEQETATWT

>*Mus musculus* TLR5 NP_058624.2

MDAEFPHAPHFSRIMACQLDLLIGVIFMASPVLVISPCSSDGRIAFFRGCNLTQIPWILNTTTERLLLSFNYISMVVATSFPLLERLQLLELGTQYANLTIGPGAFRNLPNLRILDLGQSQIEVLNRDAFQGLPHLLELRLFSCGLSSAVLSDGYFRNLYSLARLDLSGNQIHSLRLHSSFRELNSLSDVNFAFNQIFTICEDELEPLQGKTLSFFGLKLTKLFSRVSVGWETCRNPFRGVRLETLDLSENGWTVDITRNFSNIIQGSQISSLILKHHIMGPGFGFQNIRDPDQSTFASLARSSVLQLDLSHGFIFSLNPRLFGTLKDLKMLNLAFNKINKIGENAFYGLDSLQVLNLSYNLLGELYNSNFYGLPRVAYVDLQRNHIGIIQDQTFRLLKTLQTLDLRDNALKAIGFIPSIQMVLLGGNKLVHLPHIHFTANFLELSENRLENLSDLYFLLRVPQLQFLILNQNRLSSCKAAHTPSENPSLEQLFLTENMLQLAWETGLCWDVFQGLSRLQILYLSNNYLNFLPPGIFNDLVALRMLSLSANKLTVLSPGSLPANLEILDISRNQLFSPDPALFSSLRVLDITHNEFVCNCELSTFISWLNQTNVTLFGSPADVYCMYPNSLLGGSLYNISTEDCDEEEAMRSLKFSLFILCTVTLTLFLVITLVVIKFRGICFLCYKTIQKLVFKDKVWSLEPGAYRYDAYFCFSSKDFEWAQNALLKHLDAHYSSRNRLRLCFEERDFIPGENHISNIQAAVWGSRKTVCLVSRHFLKDGWCLEAFRYAQSRSLSDLKSILIVVVVGSLSQYQLMRHETIRGFLQKQQYLRWPEDLQDVGWFLDKLSGCILKEEKGKKRSSSIQLRTIATIS

>*Mus musculus* TLR6 NP_035734.3

MVKSLWDSLCNMSQDRKPIVGSFHFVCALALIVGSMTPFSNELESMVDYSNRNLTHVPKDLPPRTKALSLSQNSISELRMPDISFLSELRVLRLSHNRIRSLDFHVFLFNQDLEYLDVSHNRLQNISCCPMASLRHLDLSFNDFDVLPVCKEFGNLTKLTFLGLSAAKFRQLDLLPVAHLHLSCILLDLVSYHIKGGETESLQIPNTTVLHLVFHPNSLFSVQVNMSVNALGHLQLSNIKLNDENCQRLMTFLSELTRGPTLLNVTLQHIETTWKCSVKLFQFFWPRPVEYLNIYNLTITERIDREEFTYSETALKSLMIEHVKNQVFLFSKEALYSVFAEMNIKMLSISDTPFIHMVCPPSPSSFTFLNFTQNVFTDSVFQGCSTLKRLQTLILQRNGLKNFFKVALMTKNMSSLETLDVSLNSLNSHAYDRTCAWAESILVLNLSSNMLTGSVFRCLPPKVKVLDLHNNRIMSIPKDVTHLQALQELNVASNSLTDLPGCGAFSSLSVLVIDHNSVSHPSEDFFQSCQNIRSLTAGNNPFQCTCELRDFVKNIGWVAREVVEGWPDSYRCDYPESSKGTALRDFHMSPLSCDTVLLTVTIGATMLVLAVTGAFLCLYFDLPWYVRMLCQWTQTRHRARHIPLEELQRNLQFHAFVSYSEHDSAWVKNELLPNLEKDDIRVCLHERNFVPGKSIVENIINFIEKSYKAIFVLSPHFIQSEWCHYELYFAHHNLFHEGSDNLILILLEPILQNNIPSRYHKLRALMAQRTYLEWPTEKGKRGLFWANLRASFIMKLALVNEDDVKT

>*Mus musculus* TLR7 NP_001277684.1

MVFSMWTRKRQILIFLNMLLVSRVFGFRWFPKTLPCEVKVNIPEAHVIVDCTDKHLTEIPEGIPTNTTNLTLTINHIPSISPDSFRRLNHLEEIDLRCNCVPVLLGSKANVCTKRLQIRPGSFSGLSDLKALYLDGNQLLEIPQDLPSSLHLLSLEANNIFSITKENLTELVNIETLYLGQNCYYRNPCNVSYSIEKDAFLVMRNLKVLSLKDNNVTAVPTTLPPNLLELYLYNNIIKKIQENDFNNLNELQVLDLSGNCPRCYNVPYPCTPCENNSPLQIHDNAFNSLTELKVLRLHSNSLQHVPPTWFKNMRNLQELDLSQNYLAREIEEAKFLHFLPNLVELDFSFNYELQVYHASITLPHSLSSLENLKILRVKGYVFKELKNSSLSVLHKLPRLEVLDLGTNFIKIADLNIFKHFENLKLIDLSVNKISPSEESREVGFCPNAQTSVDRHGPQVLEALHYFRYDEYARSCRFKNKEPPSFLPLNADCHIYGQTLDLSRNNIFFIKPSDFQHLSFLKCLNLSGNTIGQTLNGSELWPLRELRYLDFSNNRLDLLYSTAFEELQSLEVLDLSSNSHYFQAEGITHMLNFTKKLRLLDKLMMNDNDISTSASRTMESDSLRILEFRGNHLDVLWRAGDNRYLDFFKNLFNLEVLDISRNSLNSLPPEVFEGMPPNLKNLSLAKNGLKSFFWDRLQLLKHLEILDLSHNQLTKVPERLANCSKSLTTLILKHNQIRQLTKYFLEDALQLRYLDISSNKIQVIQKTSFPENVLNNLEMLVLHHNRFLCNCDAVWFVWWVNHTDVTIPYLATDVTCVGPGAHKGQSVISLDLYTCELDLTNLILFSVSISSVLFLMVVMTTSHLFFWDMWYIYYFWKAKIKGYQHLQSMESCYDAFIVYDTKNSAVTEWVLQELVAKLEDPREKHFNLCLEERDWLPGQPVLENLSQSIQLSKKTVFVMTQKYAKTESFKMAFYLSHQRLLDEKVDVIILIFLEKPLQKSKFLQLRKRLCRSSVLEWPANPQAHPYFWQCLKNALTTDNHVAYSQMFKETV

>*Mus musculus* TLR8 NP_573475.2

MENMPPQSWILTCFCLLSSGTSAIFHKANYSRSYPCDEIRHNSLVIAECNHRQLHEVPQTIGKYVTNIDLSDNAITHITKESFQKLQNLTKIDLNHNAKQQHPNENKNGMNITEGALLSLRNLTVLLLEDNQLYTIPAGLPESLKELSLIQNNIFQVTKNNTFGLRNLERLYLGWNCYFKCNQTFKVEDGAFKNLIHLKVLSLSFNNLFYVPPKLPSSLRKLFLSNAKIMNITQEDFKGLENLTLLDLSGNCPRCYNAPFPCTPCKENSSIHIHPLAFQSLTQLLYLNLSSTSLRTIPSTWFENLSNLKELHLEFNYLVQEIASGAFLTKLPSLQILDLSFNFQYKEYLQFINISSNFSKLRSLKKLHLRGYVFRELKKKHFEHLQSLPNLATINLGINFIEKIDFKAFQNFSKLDVIYLSGNRIASVLDGTDYSSWRNRLRKPLSTDDDEFDPHVNFYHSTKPLIKPQCTAYGKALDLSLNNIFIIGKSQFEGFQDIACLNLSFNANTQVFNGTEFSSMPHIKYLDLTNNRLDFDDNNAFSDLHDLEVLDLSHNAHYFSIAGVTHRLGFIQNLINLRVLNLSHNGIYTLTEESELKSISLKELVFSGNRLDRLWNANDGKYWSIFKSLQNLIRLDLSYNNLQQIPNGAFLNLPQSLQELLISGNKLRFFNWTLLQYFPHLHLLDLSRNELYFLPNCLSKFAHSLETLLLSHNHFSHLPSGFLSEARNLVHLDLSFNTIKMINKSSLQTKMKTNLSILELHGNYFDCTCDISDFRSWLDENLNITIPKLVNVICSNPGDQKSKSIMSLDLTTCVSDTTAAVLFFLTFLTTSMVMLAALVHHLFYWDVWFIYHMCSAKLKGYRTSSTSQTFYDAYISYDTKDASVTDWVINELRYHLEESEDKSVLLCLEERDWDPGLPIIDNLMQSINQSKKTIFVLTKKYAKSWNFKTAFYLALQRLMDENMDVIIFILLEPVLQYSQYLRLRQRICKSSILQWPNNPKAENLFWQSLKNVVLTENDSRYDDLYIDSIRQY

>*Mus musculus* TLR9 NP_112455.2

MVLRRRTLHPLSLLVQAAVLAETLALGTLPAFLPCELKPHGLVDCNWLFLKSVPRFSAAASCSNITRLSLISNRIHHLHNSDFVHLSNLRQLNLKWNCPPTGLSPLHFSCHMTIEPRTFLAMRTLEELNLSYNGITTVPRLPSSLVNLSLSHTNILVLDANSLAGLYSLRVLFMDGNCYYKNPCTGAVKVTPGALLGLSNLTHLSLKYNNLTKVPRQLPPSLEYLLVSYNLIVKLGPEDLANLTSLRVLDVGGNCRRCDHAPNPCIECGQKSLHLHPETFHHLSHLEGLVLKDSSLHTLNSSWFQGLVNLSVLDLSENFLYESITHTNAFQNLTRLRKLNLSFNYRKKVSFARLHLASSFKNLVSLQELNMNGIFFRLLNKYTLRWLADLPKLHTLHLQMNFINQAQLSIFGTFRALRFVDLSDNRISGPSTLSEATPEEADDAEQEELLSADPHPAPLSTPASKNFMDRCKNFKFTMDLSRNNLVTIKPEMFVNLSRLQCLSLSHNSIAQAVNGSQFLPLTNLQVLDLSHNKLDLYHWKSFSELPQLQALDLSYNSQPFSMKGIGHNFSFVTHLSMLQSLSLAHNDIHTRVSSHLNSNSVRFLDFSGNGMGRMWDEGGLYLHFFQGLSGLLKLDLSQNNLHILRPQNLDNLPKSLKLLSLRDNYLSFFNWTSLSFLPNLEVLDLAGNQLKALTNGTLPNGTLLQKLDVSSNSIVSVVPAFFALAVELKEVNLSHNILKTVDRSWFGPIVMNLTVLDVRSNPLHCACGAAFVDLLLEVQTKVPGLANGVKCGSPGQLQGRSIFAQDLRLCLDEVLSWDCFGLSLLAVAVGMVVPILHHLCGWDVWYCFHLCLAWLPLLARSRRSAQTLPYDAFVVFDKAQSAVADWVYNELRVRLEERRGRRALRLCLEDRDWLPGQTLFENLWASIYGSRKTLFVLAHTDRVSGLLRTSFLLAQQRLLEDRKDVVVLVILRPDAHRSRYVRLRQRLCRQSVLFWPQQPNGQGGFWAQLSTALTRDNRHFYNQNFCRGPTAE

>*Mus musculus* TLR11 NP_991388.2

MLKESMPRMERHQFCSVLLILILLTLVSLTLTGWAWTIPDCIIADSLLFPNLSYYIPFCTSAPGLHLLASCSNVKNLNQTLKRVPRNTEVLCLQGMVPTLPAKAFIRFHSLQLLRLQLRTTSVTSRTFQGLDQLQYLFFDHHAPCCLSLFLSPNCFESLRSLSSLSFQGYCLTYSQSIYLPTSLRHLTLRNSCLTKFQDLQRLFPDLLLSTSSTPNIKPGAPFLETLDLSYNLQLKQAGVRDLYGLTLHSLILDGTPLKALDLTDSGLLHLHFLSLVGTGIEKVPASLTGYSELRALDLGKNQIQNILENGEIPGYKALEFLSLHDNHLQTLPTRFLHTLPQLQKLNLSMNKLGPILELPEGLFSTNLKVLDLSYNQLCDVPHGALSLLSQLQELWLSGNNISSLSNESLQGLRQLRTLDLSWNQIKVLKPGWLSHLPALTTLNLLGTYLEYILGIQLQGPKMLRHLQLGSYPILDIYPPWPPTLLSLEIQAESCIQFMIHSGQPFLFLENLTLETSILLLKPDNITIHFPSLRRLTLRGYSFIFSTSQLQRFFPQQLPLLEHFFIWCENSYAVDLYLFGMPRLRVLELGYLNFFYESSTMKLEMLLKEVPQLQVLALSHLNLRNLSVSSFKSLQDLKLLLFNSERALEMNSNLQEFIPQMPQYVYFSDVTFTCQCEASWLESWATRAPNTFVYGLEKSICIANASDYSKTLLFSFLATNCPHGTEFWGFLTSFILLLLLIILPLISCPKWSWLHHLWTLFHTCWWKLCGHRLRGQFNYDVFISYCEEDQAWVLEELVPVLEKAPPEGEGLRLCLPARDFGIGNDRMESMIASMGKSRATLCVLTGQALASPWCNLELRLATYHLVARPGTTHLLLLFLEPLDRQRLHSYHRLSRWLQKEDYFDLSQGKVEWNSFCEQLKRRLSKAGQERD

>*Mus musculus* TLR12 NP_991392.1

MGRYWLLPGLLLSLPLVTGWSTSNCLVTEGSRLPLVSRYFTFCRHSKLSFLAACLSVSNLTQTLEVVPRTVEGLCLGGTVSTLLPDAFSAFPGLKVLALSLHLTQLLPGALRGLGQLQSLSFFDSPLRRSLFLPPDAFSDLISLQRLHISGPCLDKKAGIRLPPGLQWLGVTLSCIQDVGELAGMFPDLVQGSSSRVSWTLQKLDLSSNWKLKMASPGSLQGLQVEILDLTRTPLDAVWLKGLGLQKLDVLYAQTATAELAAEAVAHFELQGLIVKESKIGSISQEALASCHSLKTLGLSSTGLTKLPPGFLTAMPRLQRLELSGNQLQSAVLCMNETGDVSGLTTLDLSGNRLRILPPAAFSCLPHLRELLLRYNQLLSLEGYLFQELQQLETLKLDGNPLLHLGKNWLAALPALTTLSLLDTQIRMSPEPGFWGAKNLHTLSLKLPALPAPAVLFLPMYLTSLELHIASGTTEHWTLSPAIFPSLETLTISGGGLKLKLGSQNASGVFPALQKLSLLKNSLDAFCSQGTSNLFLWQLPKLQSLRVWGAGNSSRPCLITGLPSLRELKLASLQSITQPRSVQLEELVGDLPQLQALVLSSTGLKSLSAAAFQRLHSLQVLVLEYEKDLMLQDSLREYSPQMPHYIYILESNLACHCANAWMEPWVKRSTKTYIYIRDNRLCPGQDRLSARGSLPSFLWDHCPQTLELKLFLASSALVFMLIALPLLQEARNSWIPYLQALFRVWLQGLRGKGDKGKRFLFDVFVSHCRQDQGWVIEELLPALEGFLPAGLGLRLCLPERDFEPGKDVVDNVVDSMLSSRTTLCVLSGQALCNPRCRLELRLATSLLLAAPSPPVLLLVFLEPISRHQLPGYHRLARLLRRGDYCLWPEEEERKSGFWTWLRSRLG

>*Mus musculus* TLR13 NP_991389.1

MSGLYRILVQLEQSPYVKTVPLNMRRDFFFLVVTWMPKTVKMNGSSFVPSLQLLLMLVGFSLPPVAETYGFNKCTQYEFDIHHVLCIRKKITNLTEAISDIPRYTTHLNLTHNEIQVLPPWSFTNLSALVDLRLEWNSIWKIDEGAFRGLENLTLLNLVENKIQSVNNSFEGLSSLKTLLLSHNQITHIHKDAFTPLIKLKYLSLSRNNISDFSGILEAVQHLPCLERLDLTNNSIMYLDHSPRSLVSLTHLSFEGNKLRELNFSALSLPNLTNLSASRNGNKVIQNVYLKTLPQLKSLNLSGTVIKLENLSAKHLQNLRAMDLSNWELRHGHLDMKTVCHLLGNLPKLETLVFQKNVTNAEGIKQLAKCTRLLFLDLGQNSDLIYLNDSEFNALPSLQKLNLNKCQLSFINNRTWSSLQNLTSLDLSHNKFKSFPDFAFSPLKHLEFLSLSRNPITELNNLAFSGLFALKELNLAACWIVTIDRYSFTQFPNLEVLDLGDNNIRTLNHGTFRPLKKLQSLILSHNCLKILEPNSFSGLTNLRSLDLMYNSLSYFHEHLFSGLEKLLILKLGFNKITYETTRTLQYPPFIKLKSLKQLNLEGQRHGIQVVPSNFFQGLGSLQELLLGKNPSVFLDHHQFDPLINLTKLDISGTKDGDRSLYLNASLFQNLKRLKILRLENNNLESLVPDMFSSLQSLQVFSLRFNNLKVINQSHLKNLKSLMFFDVYGNKLQCTCDNLWFKNWSMNTEEVHIPFLRSYPCQQPGSQSLLIDFDDAMCNFDLGKVYFLCSFSMVLSTMVFSWFSTKMIASLWYGLYICRAWYLTKWHKTEKKFLYDAFVSFSATDEAWVYKELVPALEQGSQTTFKLCLHQRDFEPGIDIFENIQNAINTSRKTLCVVSNHYLHSEWCRLEVQLASMKMFYEHKDVIILIFLEEIPNYKLSSYHRLRKLINKQTFITWPDSVHQQPLFWARIRNALGKETVEKENTHLIVVE

>*Carcinus maenas* Toll CDO91661.1

MAGEHQPQAAPWLWLAVILALVSHFSACPICFKDFNTLYCSESNTPDNETYSLKLVNYKDAKESALTYKCHYKAPQMFLTYHQECNFSTVQYVLFQRCPLPNVSFSEIFTQLGILPEKILEMTFENIGTRKDLKLEPFHLEGLTNLDILQLKQNLFTSLSPNILQATPNLQHFLFSQNSMPTLPETLFAHTPKLVTISLLNNNFESIPDNIFVNISGLAVLRLYGNTLRELSPKLLANIPEVYKLELSVNGITKILPDTFKYLPKLQHLYMKFNELESLPQDIFHNCPNLQTVHLRNNRLVSFPSELFSKSKNITDFDFHNNRIREIHKELLQGQKNLTILIMKGNSLENLPEGAFQDLVNLNKLLLQNNPLKILPPGSFDHQRKMKTLDLSNTSLIHLPDNIFKNCESLEEIDLSNNHLSKLKSTIFPHPATVLRSLKLEKNILSFSNITSEPQASEVGEVVVEQFPLSDQVNLTDLVLNSNRIQNMPHALRNLKKLKKLDLKNNSIEYLDYYDFLYSSDTTTVDPSDDLGIFNANLEQHSLTPAQVVEVELRDNPLICDCNLNKFAQFLQDKMPEEDKVQLNVVDKQYVKCSLPNDKSVQKSVMTLDLSTLVCYKKNCHPSCTCVTIPHDHMFIMECVDQTLQAIPPLKPYLPQGNYSVKLDLRNNSITSLEGLQNPEYSNVVNLTLSYNHLKFINESFLPKRLQALDVSGNALTHFSPSLIEFLSGTNPTLSLGRNPWHCDCQLVDLYNFLRDPLRKLASSPSILCDNLDRLLSVTEEELCPTIQQPMVVSTIASTTVFLFLFFVLGTVSVYKYQQNIKVWLFTHQMCLWIVAKEEADKKKYDAFISYSNNDEEYVNTVLVPGLECGEPKYRVCLHYRDWLPGEYIQNQINQSIEDSHRTIVILSSNFIENVWGQIEFKTAHSKALKEKSKNIIVIVLGQVPPESEMDEELKLYLSTRTYLQSDHPKFWENLRYAMPHPQDFLHKKKTKTKKVEGLQMIHRNGIARP

>*Eriocheir sinensis* Toll1 AGK90305.1

MAPQRRLPAAPWLALFLVGLAIPLSFTCPNCYKSFDTLSCSMNATFAQQYKLELKDDNGTTDSLLSLRCDYRASHLDLAFHKGCNFSSVQHVSLSLCPLFNVSFSKAFAELGILPEKILQVTFDNRGVRTDLKLERWHLDGLTNLEFLDLKNTKFTTIPPDLLQATPKLQHFFFSQANMSTVPETLFAGASHLKSIHLINNNFESLPDNLFSNIFTLTGLTVYGNTLGEISPKLLSSIPKVYQLELSINKIRKITSDTFLNLPKLKTLYLKINELESLPEDIFHNCPDLETVNLQYNKLQALPSQLFSKSKMITDLSFSNNKIIEIPQGLFQGLINLTELSMSANALEKIPEGSFADLISLEKLSLNDNPLKTLLSGTFNSHHKLKTLDLKNASLSDLPSNIFKTCESLEEIDLSDNHLSELRSTFFPHPITVLRILRLGNNNLSFSTIVSKPEAREAGRTDEVLLEQFPLSDQVGLTELTLNSNRIKAIPHAFRNLQNLMQLDLRNNSIEYLDNFDFLWNPDHGNLDRSNASQAERQDLNSDIPKRVVGVKLRDNPLICDCNLYKFARLLQEKNSEQDKNMVHLEVEDSNAVKCSPPNNRSDQKLVMTLDFTKLFCYRKECHPSCSCAIRPHDHMFIMDCASQGLQAIPKLKPYLPKGNYSITLSVTNNSIASLEGLQSPSYNNLVNLTIPSNHLKVINESYLPKTLQVLDVRRNYLTNFCPSVISFLNATNANLGLGGNPWLCDCQLVDLHVFLRDPKRKLADSHSILCSNLNKQLLSLTEEELCPTIQRPMVVATIASTTVLLVVFFVLGTVVVFNFQQEIKVWLFNHRMCLWAVAKEEADANKKYDAFISYSNKDEEYVNTVLVPGLECGEPRYRVCLHYRDWVPGEYIQNQIDQSIEDSHRTIVVLSSNFIENVWGQIEFKTAHSKALREKSKKIIVIVLGQVPPESEMDEELKLYLSTRTYLQSDHPKFWENLRYAMPHPQEFFQKKQNKTKKTERLELVHKNAKPK

>*Eriocheir sinensis* Toll2 AGT21374.1

MMPQMLCCLLLGWAWLVTGAVGCGLCVEAGEGAQSSFTCTGTVDRKVHYSVEVSDEELRLSCSDVVDVDFSLMNGCDFSSVTHVQFLGCPLPTVSYQEVLQKIGVQSGNLISLTASVVAKKGVLTQQHLQGLKELQLLHLAGIHAVANNSFTQTAKLEIIEICNGTLNDLPPYLFHNLTKLETVMLHRNDIVELPPELFSSTPGLLAIHLQENNISSLQEGLFKGLTFLKTLNISKNKIEKLSSGIFSDTSAMETLDLSRNLISEIEANTFVNTMVLKRLNLGYNQLRALPPGTLEACHSLEWLKLNNNNLTHAGLFNAFPEVPSLIYLDLGHNDITIEDMYIFALNNQNKVKHLLLNNNNISVMPDALNFAFINLETLDLSWNVFKYLEFSALIFLTESIKLNLKHNKIKTLDFRWSSLSVEKKLNLALEGNQLGCDCENYGFLRVLQGKPYPLLNDTLHIEVEDIDEVFCQFSDGAMENLMDVTTEDLTCQLAIDDICSFAWRFHDSMLIIDCSYLNLTSVQSLNFTAYQDDEQDFTLILANNSLTSLDSLQNYSSLRNLSMAYNKLSSLNVSHIPPHLKALDVRGNNLTTLPFAVLSHLNTTDMSLKLGSNPWHCDCDLLDLFRFLHVPSRKVSDFTQLQCAEDGEALMNLNENDLCPFFLQPMVIVTIVAILIFLILFAVLGTVSFYKYKQGIKVWLYAHHMCLWAITEDELDADKKYDAFISYSHKDEEFVMKVLVPGLECGEPRYRVCLHYRDWVPGEYIQNQIMQSVEASRRTIVVLSSNFIESVWGQLEFRAAHSQALQDRTNRIIVIVYGKIPSENDLDEKLKLYITTKTYVRWGDNKFWEKLRYIMPHPPDLVQKKHRKRRDTDKLELCKSTSKQEVC

>*Scylla serrata* Toll AGG55849.1

MASERQALVALWLWLAVLTLATTLSSACPICSKNFQSSYCLNTSTPEEQTYSLKLSNYKNAQESALTYKCHYLTEQMYLTYHQGCNFSTVQYVLFQRCPLPNVSFSDIFAQLGIHPEKILEINFENIGTRRDLELETWHLDGLVNLQILQLKHNLFTSLPPDVFKATPNLEHFLFSQNAMLTLPETLFAHTPKLKTINLLNNNFESIPDNLFINLSNLTVLRLYGNNLKEISPKLFANTPMVYKLELSFNGITNVTSDVFKYLPKLQHLFMKFNDIEFLPPDIFHNCPELQSLHMHYNKLKSLPSELFSKSKNISDFDFQRNEIMEIPEELFHGQENLTILMMQENALENIPDGAFKDLTNLEKLLLQNNPIKSLPPGSFDHQRKMKTLNLANTSLTDLPDKIFKNCESLEEIDLSNNQLSELKSTVFPHPATILRILKLSQNNLSLTARTSEPQAQKGGEIIVEQFPFSDQVNLTYLILSSNRIRNIPHALRNLKKLKHLDLKNNSIEYLDYYGFLFSSDTTADPSDAPWNPLELPQALPTQVIKIELKDNPLICDCSLYNFALWLQGKISEGDVQLDVVDNAYIKCSMPDDRGVQKFVMTLDLNTLVCNRKVCHPSCTCVTRPHDNMFFMECVQQRLQGIPALKAYLPQGNYSVTLNLKNNSITSLEGLQRPEYSNVVNLTMANNYLKFINESYLPRRLQALDVSGNGLTHFSKSLIAFLNVTNPTLSLSGNPWLCDCQLVDLYTFLRDPLRKMANSHPIMCDNSEPLLSLTEEQLCPTIQQPMVVVTVASTTVFLFLFFVLGTVSAYKYQQNIKVWFYTHQMCLWVIAKEEADKKKYDAFISYSNKDEEYVNNVLVPGLESGEPKYRVCLHYRDWVAGDYIQSQINQSIEDSHRTIVILSSNFIENVWGQIEFKTAHSKALKEKSKNIIVIVLGQVPPESEMDEELKLYLSTRTYLQSDHPRFWENLRYAMPHPQEFLYKKRAKTKKTEGLQMVQANGKAGS

>*Daphnia pulex* Toll1 EFX84748.1

MVVRSVAELWLATAAYALCSLVPMPSAVRNYTLNTIDVRMLYESGDCACFCKLTREWTVCVGKDCLNVPRTINIFNRRLKVTGTEIATIGPLDFARYSDLLELQLDGNLLTNIENGTFANLSQLVNLSISSNRLASISPDAFRGLVSLRSLRLMKNRFLALSDVVPSLVPLTALRFLSLSDNTLSRVDAADFIPLRHSQLEALDLSNCDLKYIGSEAFMPFKKLQRLILSENTMPEDNLIYLIHTMQETGLKALDLSQLRFAGSPPRTLLEALSRTDVEELNLSKNTLPRLSPKIFPLMPRIRDLDLSACGIISIENGTFSLMPLLMRLNLAQNGLEDIPPAVMILPQLQWLSLSGNSGSAYEYGGGELKLEDGNFALMSNLTYLDLSFNRVGQVTREIFDGLSRLEELNLKNNSLYRLSEGCFHPLVSLKILHLDGNAFGKQNFSRSTFYGLNSLEYLNMDRCKLSFTDQEAIFAGAPRLRHLSMRDNQIVSFGSRNPFADATSLVSVDLFKNRIRGWDTQLFAGSPDLDVLNLAENQISTVSKAMMADIANLSEVDLLGNPIDCDCNLEPLRRYALYHEDTEDSNLLIKADHCSSPDKWRFQPITSFLLELDPDHCYYNIQASIDDQDPDVDADYSSFISRPHVIALYILIPTVCLSMLVGYAIYRSRWVIRYYMFRKRLSQTNLMSSSSMAELEGNFKYDAFVSYSNVDHAFVARMVGMLENAPPHYKLCVYERDFTAGNVLNDCIMQSIATSRKVVLVISENFIQSHWCLWELHLAQHSLLEDKRNGLVLVVVGKLKLNQCPPTLRFLMKTRIYLEWDLDPSKQRVFWERLRDALAPSSLQKSISLPDAG

>*Daphnia pulex* Toll2 EFX64981.1

MSVSPSSPPPPLNCRLPLPLLHHHRGGIVRLLIVLTVTVCSLFRPTTGLVVSLSLSPAADGCQISETNAEGTSSSLHCRFRAFNPDWTSGSLGRGLEQQSTKGLWVECADSTAYPVILPTSAFAAFPHLEWLHLDSCRLSDLPAKSLQGLAKLRQLRIQTRNADWPGTSLTISDQLLNDVRSLESLDLALNDIRSLPRPSLCALDKLVQLNLTGNRLSDLLWTRPEANRDGCLQSLKVLDMSYNRLVTLPARSLANWTQLEELHLQGNGLVSVDDNSLIGLNSLRLINLAGNQLTSLPPGLLSSSAEHLAELYVSANGLTVLAPGLLSGLSKLLVLDLSENQLTASSFDPTTLSGLFRLAVLSLHNNRISRLDSTIFSDLTNLQILRLDGNMLESLPEGIFGSLPHLHTLILSRNRLTRLDGQLMANLNSLSILALDNNLIERIDPEALANTTQLQDLNLSGNNLPSVPVALASLTRLQSLDLGENRLVGFDYVVLNGMKELSSLRLLDNQIGNVSRATFASLPSLRILNLSKNQIAAVEEGAFSQNPLLQAVRLDANELTDLTGLFHSLPNLVWLNVSDNRLAHFDYALIPKSLQWLDMHLNHIPELGNYFQLDDQLSLQTLDASFNRLTELTASMLPDSLQVLSLNDNLISSVQPYTFFRKDNLTRVDLYANHIADLDQNALRISPTSDGRPLPEFYIGGNPFQCDCNMEWLQRINTPDHLRQHPRVMDLEGIYCRLLHSSRPQRSYVPLVEATPSNFLCTYETHCFALCHCCDFDACDCEMTCPTNCTCYHDQSWSANIVDCSGGSHPNLPERIPMDVTELYLDGAQLRALSSHKFIGRKNLRLLFLNSSGVEIIHNRTFNGLRGLYVLHLEDNRIRTLEGFEFSDLESLRELYLHNNAITSIQNRTFSALKHLQVLRLDGNRLVDFPVWNLLSNAPELNALTLNDNPWSCDCLFLAELRTALHTAGPKVSDASQLICGGSNRSSNRSLGLCVSPTPSATTIVQQRVIQDYLPLLVTTLVAFIAVTLIILFVFIYRQPVRVWCHARYGLRLWASGSGSGGSAATPDSKLFDAFLSYSAKDDAFVQQMLATNLEYGSPTYKLCLQHRDCPSGGGAYGLSETISQAVDSSRRTVMIISPNFIKAEWCRFEYKSALHQLFGTSRHCQQQQTKSAKQTKRLIVILIGDVTHKDLDADLKLYLKTNTYLQWGEDGFWDKLRFALPDPVQQPSRAQQQQQQAQHQQTTHKSVRPCGGGPMINTMTAAPMAATSAAMAAHQMHLHQQRSASRPCNVTIPPRTVTLNMSG
