# Supplementary material for: RNAi screening identifies a new Toll from shrimp Litopenaeus vannamei that restricts WSSV infection through activating Dorsal to induce antimicrobial peptides
Source: PLoS Pathog. 2018 Sep 26;14(9):e1007109. doi: 10.1371/journal.ppat.1007109 (PMC6175524; doi:10.1371/journal.ppat.1007109)
Supplement: S4 Data — The putative NF-κB binding sites in their promoter are shadowed, and the transcription start site (G) and the translation initiation site (ATG) are showed. (DOCX) [file ppat.1007109.s008.docx]

Partial promoter regions of ALF1 and LYZ1 are obtained by Genome Walking method. The putative NF-κB binding sites in their promoter are shadowed, and the transcription start site (G) and the translation initiation site (**ATG**) are showed.

>Partial promoter region of ALF1

TGTGTCTGCTTGATTAGCCGATCCCAGACATCCTGCAGCCCAACAACCAAAGCCAGGCACGACGGGCGTGCGGGAGGACAGCCCTGCCTTCAGGATAAGCTCCCGGCGAGGCATGAGCG**CGGAAATGCA**GTCCTTATGCGCAGTCGGCGACGGACAGGCTTCCGAGCAACACCGCTTCCGCATTCG**G**CCTTGACTTCGGGGGGGAAAACACGACG**ATG**CGGGTGCTGGTCAGCTCTGTAG

> Partial promoter region of LYZ1

TTTGCATTAATCCCACATTACTTTTTTCCTTGTACTAAATAATATTTCGAAATACATCAGAGATACTCCTGATTATACAAAGGTGCAACTTTTTGTCATCTGTTTCACATCGCCCTTGTTATTTGTCAATCAGGTTGCATTCGGTACTAAAATGATAAGTTCATACTGCGTAGTCAAAATGCATATCAAAAGTGATAAACTCCGATAAACATGGGTGAATTGGCTCTGATAGA**GGAAAGGCCA**ACTGCTGCTCTATGCATTATCTCACGCTCCTCAAAACAGCTTTCGGGTAAGCGGACAACTGACGTCACGTGGGAAAACAATAATTCTTGTAGATGTATAAATAACATAATCAGCAAGGAAGGGCC**G**CAGACACAGCCAAGCAACTTACACTTCGGAACCAGAAGACATA**ATG**CGCGCATC
